# Variation in the Evolution and Sequences of Proglucagon and the Receptors for Proglucagon-Derived Peptides in Mammals

**DOI:** 10.3389/fendo.2021.700066

**Published:** 2021-07-12

**Authors:** David M. Irwin

**Affiliations:** ^1^ Department of Laboratory Medicine and Pathobiology, University of Toronto, Toronto, ON, Canada; ^2^ Banting and Best Diabetes Centre, University of Toronto, Toronto, ON, Canada

**Keywords:** proglucagon, glucagon, GLP-1, GLP-2, Gcgr, Glp1r, Glp2r, evolution

## Abstract

The mammalian proglucagon gene (*Gcg*) encodes three glucagon like sequences, glucagon, glucagon-like peptide-1 (GLP-1), and glucagon-like peptide-2 that are of similar length and share sequence similarity, with these hormones having cell surface receptors, glucagon receptor (Gcgr), GLP-1 receptor (Glp1r), and GLP-2 receptor (Glp2r), respectively. Gcgr, Glp1r, and Glp2r are all class B1 G protein-coupled receptors (GPCRs). Despite their sequence and structural similarity, analyses of sequences from rodents have found differences in patterns of sequence conservation and evolution. To determine whether these were rodent-specific traits or general features of these genes in mammals I analyzed coding and protein sequences for proglucagon and the receptors for proglucagon-derived peptides from the genomes of 168 mammalian species. Single copy genes for each gene were found in almost all genomes. In addition to glucagon sequences within Hystricognath rodents (e.g., guinea pig), glucagon sequences from a few other groups (e.g., pangolins and some bats) as well as changes in the proteolytic processing of GLP-1 in some bats are suggested to have functional effects. GLP-2 sequences display increased variability but accepted few substitutions that are predicted to have functional consequences. In parallel, Glp2r sequences display the most rapid protein sequence evolution, and show greater variability in amino acids at sites involved in ligand interaction, however most were not predicted to have a functional consequence. These observations suggest that a greater diversity in biological functions for proglucagon-derived peptides might exist in mammals.

## Introduction

The mammalian proglucagon (*Gcg*) gene encodes three glucagon-like sequences, glucagon, glucagon-like peptide-1 (GLP-2), and glucagon-like peptide-2 (GLP-2), which have diverse hormonal roles in the regulation of metabolism (1–4). Mammalian *Gcg* is primarily expressed in the alpha cells of the pancreas, L-cells of the intestine, and some neurons of the nucleus tractus solitarii of the brainstem (1–4). Tissue-specific proteolytic processing of the proglucagon (Gcg) precursor protein by prohormone convertase enzymes (5) results in the production of glucagon as the major product secreted from pancreatic alpha cells, while GLP-1_7-37_ [which mostly circulates as GLP-1_7-36_amide (4)] and GLP-2 are released from intestinal and neuronal cells (1–4). The primary function of glucagon is to act as the counter regulatory hormone to insulin with a major role stimulating the production and release of glucose from the liver when blood glucose levels are low (6). GLP-1_7-37_ is best known as the incretin hormone secreted by L-cells of the intestine in response to food that potentiates the release of insulin from pancreatic beta cells (3, 7). In addition, GLP-1_7-37_ has roles in the regulation of appetite, gut motility, inflammation, apoptosis, learning and memory, and has cardio- and neuro-protective effects among other roles (3, 4, 7). GLP-2 is co-secreted with GLP-1 by the L-cells in the intestine and has multiple important roles in the maintenance of the intestinal tract, including the promotion of cell growth and villi height, and improving digestive, absorptive, and barrier functions (8, 9). In addition to glucagon and the glucagon-like peptides, Gcg is proteolytically processed to produce several other peptides (1, 2, 4), some of which have well established physiological roles (4, 10, 11). Glicentin, also called enteroglucagon (12), and oxyntomodulin (OXM) are two longer peptides that overlap the glucagon sequence (1, 2, 4, 10, 11). A physiological role for glicentin has not clearly been identified (12), but see (13), while OXM is involved in the regulation of gastric acid release and other intestinal secretions and has roles in food intake and energy expenditure (10, 11). Miniglucagon, a C-terminal secondary proteolytically processed form of glucagon (Glucagon_19-29_), is antagonistic to many of the roles of glucagon (14). Other less studied peptides produced by the proteolytic processing of Gcg, including glicentin-related pancreatic polypeptide (GRPP), major proglucagon fragment (MPGF), and intervening peptide-1 and -2 (IP-1 and IP2), may also have roles in human physiology (1–4, 10).

*Gcg* genes have been identified in the genomes of diverse vertebrate species (15). While all currently characterized mammalian *Gcg* genes encode three glucagon like sequences, lineage specific duplications and deletions of exons within this gene have resulted in changes in the number of glucagon-like hormones encoded by this gene in many non-mammalian species (16–20). In cartilaginous fish (e.g., *Callorhinchus milii*, the elephant shark) the *Gcg* gene encodes 4 glucagon-like sequences, including a glucagon-like peptide 3 (GLP-3) (16), while in the frog *Xenopus laevis* (African clawed frog) the GLP-1 encoding exon has been triplicated to result in a coding sequence that could generate 5 glucagon-like sequences (17). Jawless fish (e.g., the lamprey *Petromyzon marinus*) (18) and teleost fish (e.g., zebrafish (*Danio rerio*) and anglerfish (*Lophius americanus*)) (16, 19, 20) are a pair of lineages that have duplicated *Gcg* genes, where in both lineages one of the duplicated genes has lost an exon that encodes a glucagon-like peptide (the GLP-1 encoding exon in lamprey and the GLP-2 encoding exon in teleost fish). In addition to these changes in the structure of the *Gcg* gene in diverse vertebrates, changes affecting the coding sequence that potentially alter hormone function have been identified (15, 21, 22). The glucagon hormones produced by New World rodents (i.e., suborder Hystricomorpha, which includes the guinea pig, *Cavia porcellus*) have reduced biological activity and have sequences with increased numbers of amino acid substitutions (21). The *Gcg* gene of the platypus (*Ornithorhynchus anatinus*), a member of the earliest diverging lineage of mammals (order Monotremata), encodes a GLP-1_7-37_ sequence that has accumulated many amino acid substitutions (15) and was found to be a component of the venom produced by this species (22). Accelerated evolution of the GLP-2 sequence was inferred to have occurred on the early mammalian lineage, however the physiological consequence of this change remains unknown (15).

In addition to *Gcg*, the genomes of vertebrate species also contain other genes that encode glucagon-like sequences. Glucose-dependent insulinotropic peptide (GIP), which has been described as an incretin hormone in some mammals (e.g., human (*Homo sapiens*) and mouse (*Mus musculus*)) (23), is encoded by the *Gip* gene and is found in most vertebrates (24, 25). While a 42 amino-acid-long peptide hormone is predominantly generated from the prohormone precursor in mammals (26), a shorter hormone sequence is likely produced from this gene in species other vertebrate classes due to changes in the site of proteolytic processing (24, 25). The second gene was first identified to encode Exendin, a component of the toxin produced by the Gila monster (*Heloderma horridum*; class Reptilia) (27–29). Subsequently, the homolog of this gene was found in diverse vertebrates and has been called *Exendin-like* (25, 30, 31), *Gcgl* (glucagon-like) (32), and *Gcrp* (glucagon-related peptide) (33). Intriguingly, while this gene is conserved in the genomes of species in most vertebrate classes, it was lost on the lineage leading to mammals (25, 30).

Glucagon, GLP-1, and GLP-2, along with the other glucagon-like sequences, act through cell surface receptors that are expressed in tissue-specific patterns to direct their distinct physiological functions (1–4, 6–9). Receptors for all glucagon-like sequences (glucagon receptor (*Gcgr*), GLP-1 receptor (*Glp1r*), GLP-2 receptor (*Glp2r*), GIP receptor (*Gipr*), and glucagon receptor-like receptor (*Grlr*; receptor for exendin-like peptides)) belong to a closely related subfamily within Class B1 of the 7-transmembrane domain G protein-coupled receptor gene family (34–37). The glucagon receptor-like subfamily of G protein-coupled receptors originated very early in vertebrate evolution with all members being present in the genome of the common ancestor of all extant vertebrates (35–37). Despite an early origin for these genes, some of these receptor genes have been lost on several different vertebrate lineages. *Glp1r* was lost from the genome on the bony fish lineage, *Gipr* was lost in parallel on both the cartilaginous fish and bird lineages, and *Grlr* is not found in mammalian genomes (25, 31–33, 35). The loss of these genes likely contributed to changes in the physiological functions of these glucagon-like hormones. For example, in some teleost fish GLP-1 promotes glucose production instead of acting as an incretin hormone (38). These fish have lost their *Glp1r* gene but have a duplicated glucagon receptor (*Gcgr*) gene (16, 36, 37). In these fish, GLP- binds to a glucagon receptor encoded by one of their duplicated *Gcgr* genes, thus it signals as if it was glucagon (16, 39–41). Despite all the receptors being 7-transmembrane domain receptors, differences in the rates of evolution of these types of receptor genes have also been detected within vertebrates and rodents, with *Glp1r* seen to be evolving slower than *Gcgr* or *Glp2r*, which suggests that different levels of physiological and selective constraint have been imposed on these genes (16, 35, 42).

Changes in diet likely impacts the roles and evolution of *Gcg* and genes for receptors for proglucagon-derived peptides since these peptide hormones and their receptors play essential roles in glucose metabolism, especially the maintenance of blood glucose levels (1–4, 6–9). Mammalian species possess a great variety of diets that yield differing levels of carbohydrates, lipids, and proteins that can be absorbed through the gut. Changes in diet have been linked to changes in the evolution of some mammalian genes involved in metabolism, including glucokinase regulatory protein (*Gckr*) (43), insulin-like peptide 5 (*Insl5*) (44), and potentially insulin (*Ins*) (45). A result study of insulin (*Ins*) genes in diverse mammals revealed unexpected changes in the sequence of the insulin hormone in several lineages (45) raising the possibility that similar changes have occurred in the gene for its counter regulatory hormone. The rapidly increasing numbers of genome sequences has revolutionized biology (46), thus it has become possible to characterize genes from large numbers of species and identify genes that display differences in their evolution patterns that might suggest novel functions. As previously shown for hormones isolated from other vertebrates, such as fish, homologous peptides from other species might have unique properties that are beneficial for human health (47, 48). To determine whether *Gcg*, *Gcgr*, *Glp1r* and *Glp2r* genes in specific mammals have displayed unexpected patterns of evolution, and potentially novel functions, I examined the evolution of these genes from many diverse mammalian genomes.

## Materials and Methods

### Database Searches

Searches for the genomic and coding sequences for the genes encoding proglucagon (*Gcg*), glucagon receptor (*Gcgr*), glucagon-like peptide-1 (GLP-1) receptor (*Glp1r*), and glucagon-like peptide-2 (GLP-2) receptor (*Glp2r*) were conducted in January 2021. Genome sequences for 153 mammalian species were available in the National Center for Biotechnology Information (NCBI) genome data viewer database (www.ncbi.nlm.nih.gov/genome/gdv/) and 15 additional species had genomes in the Ensembl genome database (www.ensembl.org) for a total of 168 mammalian species (see **Supplementary Table 1** for a list of species and their genome assemblies). Searches of these mammalian genomes were conducted, with the tBlastn algorithm (49), for coding sequences that predict proteins with similarity to human (*Homo sapiens*) proglucagon (GCG, NP_002045.1), glucagon receptor (GCGR, NP_000151.1), glucagon-like peptide-1 (GLP-1) receptor (GLP1R, NP_002053.3), and glucagon-like peptide-2 (GLP-2) receptor (GLP2R, NP_004237.1) protein sequences. While the human protein sequences were used my initial searches, for species where an incomplete coding sequence was initially identified, subsequent searches were conducted, using tBastn and Blastn (49), with protein or DNA sequences, identified here, from a species that was expected to be more closely related. In addition, for species that did not have complete *Gcg* coding sequences identified in our initial searches, tBlastn and Blastn searches were conducted on their genome sequences to identify sequences that could potentially encode the missing genes or exons as previously described (15, 35, 42, 45). The Ensembl genome database identifies potential orthologs of genes, where it uses gene order conservation (GOC), which are based on the presence or absence of orthologous neighboring genes, and whole genome alignment (WGA) coverage, based on the alignment of putative orthologs, scores to determine whether a putative ortholog is “high confidence” or not. I obtained the GOC and WGA scores as well as confidence level of each putative ortholog from the Ensembl database when the human genes were used as queries.

### Sequence Alignment and Phylogenetic Analysis

Full-length *Gcg*, *Gcgr*, *Glp1r*, and *Glp2r* coding sequences were aligned using the codon aware MAFFT algorithm (50) implemented on the Guidance2 server (guidance.tau.ac.il/ver2/) (51). To minimize the introduction of errors into the phylogenetic analyses (52), codon-based alignments of the coding sequences generated above were trimmed to remove regions that might not have been reliably aligned with Guidance (51), using a guidance score of 0.93. Phylogenetic relationships of the sequences were then established using maximum likelihood, as implemented in IQ-TREE (version 1.6.12; iqtree.cibiv.univie.ac.at) (53) where ModelFinder (54) was used to identify the best fitting evolutionary models and 1000 Ultrafast bootstrap replications (55) used to assess the confidence of the nodes in the trees. Coding sequences from *Ornithorhynchus anatinus* (platypus, order Monotremata), or from species of the order Metatheria (e.g., opossum; *Monodelphis domestica*) if no monotreme sequence was available, were used as outgroups to root the phylogenetic trees. Divergence times between pairs of species are from the TimeTree database (www.timetree.org) (56). Consensus sequences, displayed as a sequence logo, were generated using WebLogo 3 (weblogo.threeplusone.com/) (57).

### Genomic Neighborhood Analysis

To confirm the orthology of the *Gcg*, *Gcgr*, *Glp1r* and *Glp2r* genes among mammals, genomic neighborhoods surrounding these genes in the annotated mammalian genomes were examined. Genomic neighborhoods, i.e., relative positions and orientations of adjacent genes, was derived from the displays of the genomic information presented in the NCBI and Ensembl databases. Relative positions of annotated genes adjacent to *Gcg*, *Gcgr*, *Glp1r*, and *Glp2r* genes in the genomic sequences identified above were obtained from the NCBI or Ensembl databases as previously described (31, 35, 58). Briefly, the identity and orientation of genes were identified from displays of the genomic sequences in the two databases

### Evolutionary Analysis of Protein Sequences

Protein sequence alignments were generated from the codon based MAFFT alignments of the coding sequences generated above using MEGA X (59). Potential signal peptidase cleavage sites were predicted using SignalP 5.0 (www.cbs.dtu.dk/services/SignalP/) (60) and NeuroPred (neuroproteomics.scs.illinois.edu/neuropred.htm) (61), using default settings, was used to predict the locations of potential prohormone processing sites in the Gcg protein sequences. Amino acid residues in the glucagon (GCGR), GLP-1 (GLP1R), and GLP-2 (GLP2R) receptor protein sequences that interact with the proglucagon-derived peptides are from sites identified in crystal structures and were retrieved from the GCPRdb (gpcrdb.org) (62–66). The potential consequence of amino acid substitutions, compared to the human sequence, in the proglucagon-derived peptide sequences of Gcg, and the ligand interaction sites of Gcgr, Glp1r, and Glp2r were assessed using SIFT (sift.bii.a-star.edu.sg/www/SIFT_seq_submit2.html) (67) and PROVEAN (provean.jcvi.org/seq_submit.php) (68). Co-evolution of sites within or between coding sequences were assessed using BGM (Bayesian Graphical Models) (69) as implemented on the Datamonkey Adaptive Evolution server (datamonkey.org/) (70). These analyses used sequences from 78 species that had intact coding sequences for *Gcgr*, *Glp1r*, and *Glp2r* (**Supplementary Tables 3**–**5**). To identify co-evolution between *Gcg*, and its encoded proglucagon-derived peptides, and *Gcgr*, *Glp1r*, and *Glp2r*, concatenated coding alignments (receptor plus *Gcg* sequences) were used. Here, sequences from 77 species that have intact coding sequences from all three receptors (*Gcgr*, *Glp1r*, and *Glp2r*) and *Gcg* were used. These sequences were the 78 species used for the comparisons among receptors with the exclusion of *Myotis myotis* (greater mouse-eared bat) as its *Gcg* coding sequence was incomplete (**Supplementary Table 2**). Gcgr, Glp1r, and Glp2r protein sequences were visualized as snake plots generated with Protter (wlab.ethz.ch/protter/start/) (71). Uniprot accessions GLR_HUMAN, GLP1R_HUMAN, and GLP2R_HUMAN, for human GCGR, GLP1R, and GLP2R, respectively, were used to generate the snake plots. Amino acid residues in the Gcgr, Glp1r, and Glp2r sequences were numbered based on the human sequences and the Wooten numbering system (72).

### Evolutionary Analysis of Coding Sequences

Comparisons of the relative rates of nonsynonymous (d_N_) and synonymous substitutions (d_S_) can be used to measure the strength of selective pressure on a coding sequence. Sequences experiencing stronger purifying selective pressure, to conserve protein sequence and function, will have lower nonsynonymous to synonymous (d_N_/d_S_) ratios. To determine whether the levels of selective pressure acting upon *Gcgr*, *Glp1r*, and *Glp2r* sequences on different mammalian lineages was intensified or relaxed, average d_N_/d_S_ rate ratios were calculated and the differences in these ratios between lineages were tested using RELAX (73) as implemented on the Datamonkey Adaptive Evolution server (datamonkey.org/) (70). Briefly, an alignment of the *Gcgr*, *Glp1r*, and *Glp2r* coding sequences from 78 mammals that possess complete sequences of all three receptor genes was generated using MAFFT as described above. To test the coding sequences within a mammalian lineage, only those sequences for the three receptor genes of that lineage were used as the input for RELAX. Coding sequences for pairs of genes (i.e., *Gcgr versus Glp1r, Gcgr versus Glp2r*, and *Glp1r versus Glp2r*) were then tested to determine whether the average d_N_/d_S_ ratios for these genes on a lineage differ with RELAX (73).

## Results

### Numbers of *Gcg*, *Gcgr*, *Glp1r*, and *Glp2r* Genes in Mammalian Genomes

Searches of the 168 annotated mammalian genomes available in the NCBI Genome Data Viewer (www.ncbi.nlm.nih.gov/genome/gdv/) and Ensembl (www.ensembl.org/index.html) databases (see **Supplementary Table 1** for list of species and their genome assemblies) resulted the identification of putative *Gcg*, *Gcgr*, *Glp1r*, *Glp2r* genes from 168, 160, 168, and 166 species, respectively (**Table 1**, **Supplementary Tables 2**–**5**, and **Supplementary Files 1**–**4**).

**Table 1:** Numbers of *Gcg*, *Gcgr*, *Glp1r*, and *Glp2r* genes found in annotated mammalian genomes.

	*Gcg*	*Gcgr*	*Glp1r*	*Glp2r*
**Present** ^a^	168	160	168	166
Intact	162^b^	124	105	125
Incomplete	6	36	63	51
**Missing**	0	8	0	2

a Total of complete and incomplete genes.

b Includes 3 genes missing exon 6, which possess only 4 coding bases.

Of the 168 identified *Gcg* coding sequences, 159 were distributed over 5 coding exons in the genome sequences and predict proteins that were approximately 180 amino acids in length (**Table 1**, **Supplementary Table 2**, and **Supplementary File 1**), as seen for the rat and human *Gcg* genes (74, 75), and thus appear intact. Of the *Gcg* sequences from the remaining 9 species, 3 were found to be missing exon 6 that possess only 4 coding bases, but otherwise predict an intact open reading frame. Gaps were found downstream of exon 5 in all three of these genome assemblies, raising the possibility that the exon 6 sequences reside in an assembly gap. These three sequences, together with the 159 complete coding sequences were used in the remaining evolutionary analyses described below. The genomic sequences for the *Gcg* genes in the last 6 species have several gaps resulting in predicted coding sequences with larger missing regions, however, these sequences do not contain inframe stop codons or frame shifting mutations, thus, it is likely that they are incompletely assembled genes rather than pseudogenes.

Smaller numbers of genes with complete intact open reading frames were found for the putative *Gcgr*, *Glp1r*, and *Glp2r* genes (**Table 1**, **Supplementary Tables 3**–**5**, and **Supplementary Files 2**–**4**), which likely reflects the larger sizes of these genes (1350-1600 coding bases and 13 coding exons) compared to the *Gcg* gene (540 coding bases and 5 coding exons). A total of 124, 105, and 125 species were found to have complete intact coding sequences, for *Gcgr*, *Glp1r*, and *Glp2r*, respectively, with 36, 63, and 41 incomplete coding sequences, respectively, also identified (**Table 1** and **Supplementary Tables 3**–**5**). As seen for the *Gcg* genes described above, all of the incomplete *Gcgr*, *Glp1r*, and *Glp2r* genes do not contain inframe stop codons or frame shifting mutations, suggesting that they are incomplete due to gaps in the genome assemblies rather than being pseudogenes. I failed to identify a *Gcgr* or *Glp2r* gene in 8 and 2 species, respectively (**Table 1** and **Supplementary Tables 3** and **5**), but this might not be due to the loss of the gene in these species, but instead due to incomplete gene assemblies. Both *Gcgr* and *Glp2r* are members of the very large gene family of G protein-coupled receptors (34), thus, multiple genomic sequences were found to have similarity in these species to the query sequences, however many of these genomic hits were composed of a single exon that could not reliably be assigned to a specific gene ortholog. No obvious clustering of species with incomplete or missing *Gcg*, *Gcgr*, *Glp1r*, or *Glp2r* genes was observed (**Supplementary Tables 2**–**5**).

### Orthology of Mammalian *Gcg*, *Gcgr*, *Glp1r*, and *Glp2r* Genes

The *Gcg*, *Gcgr*, *Glp1r*, and *Glp2r* genes were identified above by similarity. Next, I examined the genes to assess their orthology, to exclude the possibility that some of the genes might be paralogs that originated through a more ancient gene duplication. Orthology of genes can be assessed using the Ensembl database, with 104 of the 162 mammalian species examined here represented in this database (**Supplementary Table 1**). Using the human *GCG*, *GCGR*, *GLP1R*, and *GLP2R* genes as queries, I identified mammalian species that were reported by Ensembl to have orthologs of these genes, resulting in the identification of 87, 73, 87, and 90 orthologs for each, respectively (**Supplementary Tables 2**–**5**). Genomic neighborhoods are often conserved between species, thus not only is the studied gene orthologous, but genes adjacent to the studied gene also show orthologous relationships between species, especially when the species are relatively closely related such as mammals (76). Previous studies have shown that the gene neighborhoods flanking the *Gcg*, *Gcgr*, *Glp1r*, and *Glp2r* are largely conserved across vertebrates, while paralogous genes (e.g., *Gcgr*, *Glp1r*, and *Glp2r*) generally have unrelated neighboring genes (30, 31, 36). All 87 mammalian species with a reported *Gcg* ortholog in the Ensembl database were reported to be “high confidence” orthologs (**Supplementary Table 2**). “High confidence” is based on sequence similarity (e.g., WGA scores) and conservation of their genomic neighborhoods (GOC scores) that is evaluated by Ensembl. While 87 species were reported to have “high confidence” orthologs, 16 species were not reported to have orthologs of human *GCG* by Ensembl. Of these 16 genes, I found that 13 do have annotated genes in the Ensembl database, while the 3 others have sequences homologous to *GCG* that were not annotated as genes. Thus, Ensembl did not consider these 16 *Gcg* gene sequences to be “high confidence” orthologs of the human *GCG* gene. All these genes were found with complete coding sequences in the NCBI database (**Supplementary Table 2**). Intriguingly, the bat *Myotis lucifugus*, was reported to have more than one *Gcg* gene-like sequence, although only one of them was reported to be a “high confidence” ortholog (**Supplementary Table 2**). Similar analyses for *Gcgr*, *Glp1r*, and *Glp2r* showed that Ensembl do not identify orthologs for these genes in 30, 16, and 13 species, respectively, with 7 of the 73 *Gcgr*, 2 of the 87 *Glp1r*, and 4 of the 90 *Glp2r* identified orthologs, respectively, not considered to be “high confidence” orthologs (**Supplementary Tables 3**–**5**). Like *GCG*, most species without an identified *GCGR*, *GLP1R*, or *GLP2R* ortholog do indeed have annotated genes in the Ensembl database, and were identified in my Blast searches described above, with 18, 13, and 13, found for *Gcgr*, *Glp1r*, and *Glp2r*, respectively (**Supplementary Tables 3**–**5**), however, these identified genes also cannot be identified as “high confidence” orthologs. In addition to the 104 species represented in the Ensembl database, *Gcg*, *Gcgr*, *Glp1r*, and *Glp2r* sequences were identified in 58 additional species with genome assemblies only in the NCBI database (**Supplementary Table 1**), thus the confidence of the orthology of these genes is not available.

**Figure 1:**
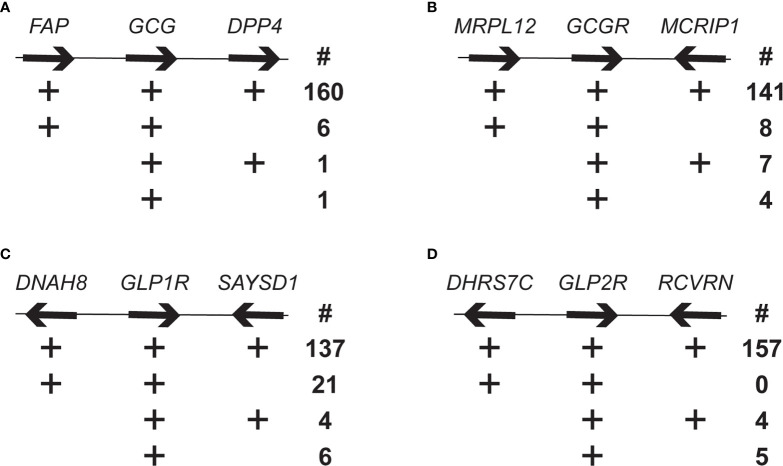
Genomic neighborhood analysis of mammalian *Gcg*, *Gcgr*, *Glp1r*, and *Glp2r* genes. Number of mammalian genomes having genomic neighborhoods for the **(A)** *Gcg*, **(B)** *Gcgr*, **(C)** *Glp1r*, and **(D)** *Glp2r* that are similar tom the human genomic neighborhoods. Organization of selected genes flanking the human *GCG*, *GCGR*, *GLP1R* and *GLP2R* genes, with arrows indicating direction of transcription. Genes and intergenic spaces are not shown to scale. The four rows below each gene are different genomic neighborhood found in the annotated mammalian genomes. The plus symbol (+) indicates presence of the gene and the number (below the # symbol) to the right is the number of species with that type of genomic neighborhood. Genes are: *FAP*, fibroblast activation protein; DPP4, dipeptidyl peptidase 4; *MRLP12*, mitochondrial ribosomal protein L12; *MCRIP1*, MAPK regulated corepressor interacting protein 1; *DNAH8*, dynein axonemal heavy chain 8; *SAYSD1*, SAYSVFN motif domain containing 1 (*SAYSD1*); *DHRS7C*, dehydrogenase/reductase 7C; *RCVRN*, recoverin.

As a next step to assess the orthology of the *Gcg*, *Gcgr*, *Glp1r*, and *Glp2r* genes, especially the “low confidence” (or no confidence) orthologs identified in the Ensembl database and the genes found only in the NCBI database, genomic neighborhoods surrounding these genes in the genomes were examined. The human *GCG* gene is flanking by the fibroblast activation protein (*FAP*) and dipeptidyl peptidase 4 (*DPP4*) genes (**Figure 1A**). Of the 168 mammalian genome assemblies examined, 160 possess this same gene neighborhood, with an additional 6 having *Fap* adjacent to *Gcg*, 1 with *Gcg* adjacent to only *Dpp4*, and 1 with *Gcg* not adjacent to either of these two genes (**Figure 1A** and **Supplementary Table 2**). *Gcg* genes with incomplete genomic neighborhoods (i.e., not adjacent to *Fap* and/or *Dpp4*) were either at an end of a genomic contig, and thus only adjacent to one of the two flanking genes, or on a short genomic contig and not adjacent to either flanking gene. Species with genes in incomplete genomic neighborhoods were not clustered as closely related species, suggesting that the differing genomic neighborhoods were not due to a chromosomal recombination event that would have been shared among related species.

The mitochondrial ribosomal protein L12 (*MRLP12*) and MAPK regulated corepressor interacting protein 1 (*MCRIP1*) genes flank *Gcgr* in the human genome (**Figure 1B**). As with *Gcg*, the vast majority, 141 of 160, of the genome assemblies have *Gcgr* located in the same genomic neighborhood, with only 8 and 7 assemblies having *Gcgr* linked to only *Mrpl12* or *Mcrip1*, respectively, and 4 where *Gcgr* was unlinked to both genes (**Figure 1B** and **Supplementary Table 3**). As with *Gcg*, the *Gcgr* genes in incomplete genomic neighborhoods were located near the end of a genomic contig or on a short contig, which suggests that the genomic neighborhood for *Gcgr* has also been conserved across mammals.

Human *GLP1R* is flanked by the dynein axonemal heavy chain 8 (*DNAH8*) gene and the SAYSVFN motif domain containing 1 (*SAYSD1*) gene (**Figure 1C**). The majority of the annotated genomes, 137 of the 168, share this same gene neighborhood for all three genes (**Table 1** and **Supplementary Table 4**). A small number of genome assemblies had the *Glp1r* gene unlinked to either gene (6) or linked to only the *Saysd1* gene (4). Intriguingly, 21 genomes showed only a linkage between *Glp1r* and *Dnah8*, and not with *Saysd1*, with many (19) of these being from rodents (**Figure 1C** and **Supplementary Table 3**). To determine whether the lack of linkage between *Glp1r* and *Saysd1* in these rodent genomes was due to the loss of the *Saysd1* gene or alternatively, to a genomic rearrangement I identified the genomic locations of *Saysd1* in the well-characterized genomes of two species that did not display this linkage, the mouse (*Mus musculus*) and rat (*Rattus norvegicus*). The genomes of both the mouse and the rat do possess a *Saysd1* gene, however, in both species the *Saysd1* was found to be located on a different chromosome from that for *Glp1r* and *Dnah8*, with *Saysd1* located on chromosomes 14 and 15 in the mouse and rat, respectively, while *Glp1r* (and *Dnah8*) located on chromosomes 17 and 14, respectively.

The human *GLP2R* gene is flanked by the genes for dehydrogenase/reductase 7C (*DHRS7C*) and recoverin (*RCVRN*) (**Figure 1D**). As seen for the other genes described above, almost all, 157 of 166, of the genome assemblies share this same gene neighborhood, with only 4 showing a linkage between *Glp2r* and only *Rcvrn* and 4 with an unlinked *Glp2r* (**Figure 1D** and **Supplementary Table 5**). As with the other genes investigated, incomplete genomic neighborhoods were found for genes that were close to an end of a genomic contig or on short contigs.

As a complementary approach to examine the orthology of the genes, separate phylogenetic analyses of the *Gcg*, *Gcgr*, *Glp1r*, and *Glp2r* coding sequences were conducted. Orthologous sequences should yield a phylogeny that is largely consistent with the accepted species phylogeny, while the presence of paralogous sequences within a dataset should yield relationships that are not expected based on our current understanding (77) of mammalian relationships (e.g., closely related species being suggested to be highly divergent). Here I used the maximum likelihood method, using IQ-TREE (53), to construct phylogenies from codon-based alignments of the intact coding sequences for *Gcg*, *Gcgr*, *Glp1r*, and *Glp2r* that had been objectively trimmed (51, 52) to remove sequences that might be unreliably aligned and interfere with the analysis (see **Methods**). All the phylogenetic analyses (**Supplementary Figures 1** **–** **4** and **Supplementary Files 5**–**8**) yielded trees that were largely consistent with the accepted mammalian phylogeny (77). Similar phylogenies were found if other methods of phylogenetic reconstruction were used (results not shown). These results support the conclusion that the sets of identified *Gcg*, *Gcgr*, *Glp1r*, and *Glp2r* gene sequences identified here are orthologs.

### Variability in Proglucagon-Derived Peptide Sequences

**Figure 2:**
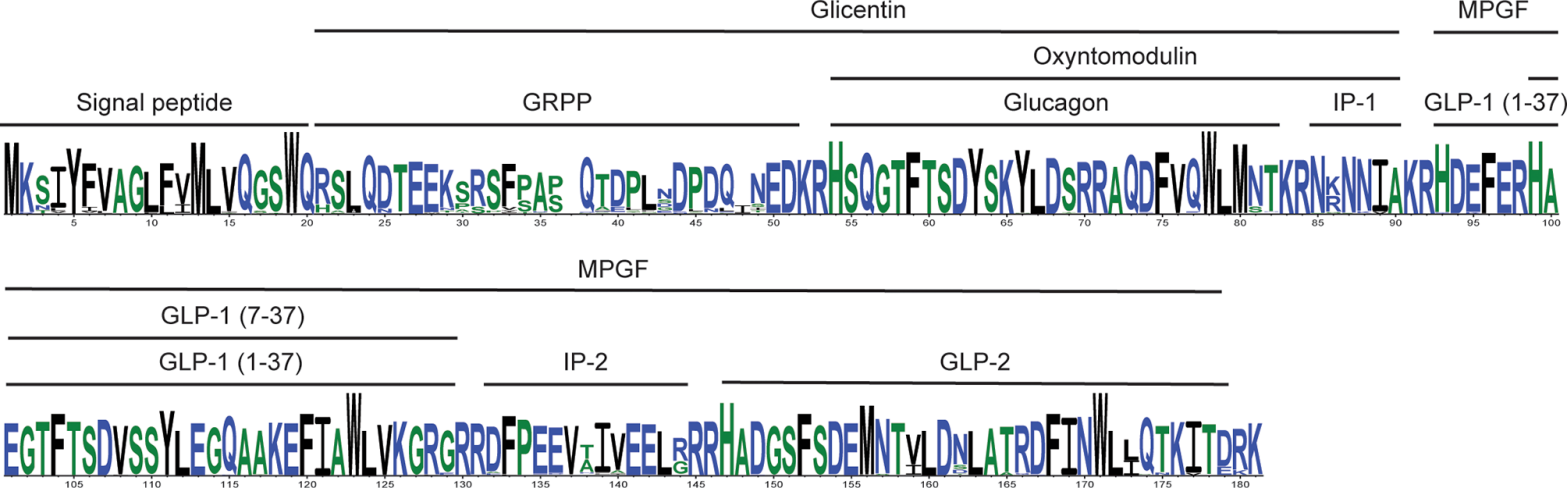
Summary of the alignment of mammalian proglucagon amino acid sequences. Consensus amino acid sequence for proglucagon encoded by mammalian *Gcg* genes. The consensus sequence was generated from aligned predicted protein sequences (see **Supplementary Figure 5**) of mammalian genes with complete open reading frames using WebLogo 3 (57). Hight of the residue is proportional to representation among the compared sequences, while residue width represents proportion of sequences without a gap (thin residues are gaps in many sequences). The locations of the different proglucagon-derived peptides are indicated by the bars above the sequence.

Previous studies had found that the proglucagon-derived peptide GLP-1_7-37_ sequence displays lower levels of variability (i.e., numbers of substitutions at a site and the number of sites accepting substitutions) than glucagon, while GLP-2 was the most variable (15). Here I examined the variability of the mammalian glucagon, miniglucagon, glicentin, OXM, GLP1_1-37_, GLP-1_7-37_, GLP-2, IP-1, IP-2, and MPGF peptide sequences using predicted Gcg protein sequences from a much larger number of mammalian species than used in earlier studies. Peptide sequences from 161 non-human Gcg sequences were compared to the human peptide sequences. When peptides that likely have biological function (1–4, 10) were examined, peptides identical to the human glucagon, miniglucagon, GLP1_1-37_, GLP-1_7-37_, GLP-2, and OXM amino acid sequences were found in 124, 124, 143, 151, 13, and 60 species, respectively (**Figure 2**, **Table 2**, **Supplementary Tables 6** and **7**, and **Supplementary Figure 5**). These results are consistent with the expectations from previous reports where a greater conservation of the glucagon and GLP-1 sequences was found compared to GLP-2 (15). While most glucagon, miniglucagon, and GLP-1_7-37_ sequences in mammals are identical to the human sequences, the vast majority of the GLP-2 and glicentin sequences differ at one or more sites. A larger number of the residues in the GLP-2 and glicentin sequence have also accepted amino acid substitutions, with 20 of the 32 sites accepting at least one amino acid substitution in a mammalian species for GLP-2 and 44 of the 69 sites in glicentin being variable, while glucagon and GLP-1_7-37_ sequences have substitutions at 11 of 29 and 13 of 31 sites, respectively, and miniglucagon (6 of 11) and OXM (17 of 37) display intermediate levels of variability (**Table 2**, **Figure 2**, and **Supplementary Figure 5**). The increased number of sites that have accepted amino acid substitutions and the larger number of different types of substitutions seen in glicentin are consistent with previous observations (12). This pattern remains even when the highly variable Gcg protein sequence from *Ornithorhynchus anatinus* (platypus) (15, 22) is excluded (**Table 2** and **Supplementary Table 7**). On average, the glucagon peptide sequences that differ from the human sequence (37 sequences) have accumulated fewer changes (average 1.68 substitutions) than the GLP-1_7-37_ (10 sequences with an average of 3.60 substitutions) or GLP-2 (148 sequences with an average of 3.32 substitutions) sequences that have accepted amino acid substitutions, even when *Ornithorhynchus anatinus* (platypus) is excluded (**Table 2** and **Supplementary Figure 5**). Intriguingly, most of the amino acid substitutions seen in glucagon fall within the 11 amino acid miniglucagon sequence (**Supplementary Figure 5** and **Supplementary Tables 6** and **7**). A total of 26 different amino acid substitutions were seen in the mammalian glucagon sequences, 18 within GLP-1_7-37_, 32 for GLP-2, 38 for OXM, and 150 for glicentin, with similar numbers found when the *Ornithorhynchus anatinus* (platypus) sequence was excluded (**Figure 2**, **Table 2**, and **Supplementary Figure 5**). The number of species that had peptides identical to GRPP, MPGF, IP-1, and IP-2, peptides that might not have biological functions (1–4, 10), were 6, 10, 64, 35, respectively (**Supplementary Table 7**).

**Table 2:** Variability of the sequences of mammalian proglucagon-derived peptides.

	Glucagon	GLP-1_7-37_	GLP-2	Glicentin	OXM
**All mammalian sequences**					
Peptide length	29	31	33	69	37
Number of species identical to the human sequence	124	151	13	5	60
Range of number of differences^a^	0-7	0-12	0-17	0-24	012
Average number of differences^b^	1.68	3.60	3.32	6.85	1.09
Average number of differences per residue^c^	0.013	0.007	0.008	0.099	0.029
Number of variable amino acid positions^d^	11	13	20	46	17
**Excluding *Ornithorhynchus anatinus***					
Range of number of differences^a^	0-5	0-6	0-7	0-22	0-3
Average number of differences^b^	1.53	2.67	3.23	6.74	1.02
Average number of differences per residue^c^	0.012	0.005	0.006	0.098	0.028
Number of variable amino acid positions^d^	10	10	17	44	15

a Compared to the human sequences. Total 161 non-human proglucagon sequences.

b Average number of substitutions found in sequences that differ from the human sequence.

c Average number of substitutions divided by peptide length.

d Number of sites in the peptide sequence that have accepted an amino acid substitution in the 162 mammalian sequences.

The potential consequences of the amino acid substitutions observed in the Gcg sequences were then examined (**Supplementary Table 8**). SIFT (67) and PROVEAN (68) were used for this analysis as both programs determine whether a tested amino acid substitution is observed in similar protein sequences, with substitutions that are unlikely considered to “Affect function” (SIFT) or be “Deleterious” (PROVEAN). While these tools are designed to assess the impact of a single substitution in a protein sequence, and thus having the remaining sequence being identical, here I used these programs to identify sites that might yield species-specific functional differences. Glucagon, OXM, and glicentin sequences had the largest number of amino acid substitutions (8) that were predicted to be both “Deleterious” [PROVEAN (68)] and “Affect function” [SIFT (67)], with only 1 such site in GLP-1_7-37_ and none in the GLP-2 sequences (**Supplementary Table 8**). All the “Deleterious” or “Affect function” sites in the OXM and glicentin sequences are in the portion of their sequences that overlap with glucagon (**Supplementary Table 8**). Most of the amino acid substitutions in both GLP-1_7-37_ (12 of 18) and GLP-2 (26 of 32), and only a few in glucagon (8 of 26), were predicted to be “Neutral” [PROVEAN (68)] or “Tolerated” [SIFT (67)], while the remaining substitutions (10, 5, and 6 in glucagon, GLP-1_7-37_, and GLP-2, respectively) having differing predicted consequences when tested with these two programs (**Supplementary Table 8**). For OXM and glicentin, most of the substitutions outside the region that overlaps glucagon were predicted to be neutral, with only one substitution in OXM and 22 (with many of these being of low confidence) in glicentin suggested to be either “Deleterious” or “Affect function” by either SIFT (67) or PROVEAN (68) (**Supplementary Table 8**).

Changes in protein sequence may also affect cellular localization and processing of hormone precursors. The variability seen at the N-terminus of the Gcg protein sequences is not suggested to impact secretion as all precursor sequences were predicted to have signal peptides and do not have potentially “Deleterious” or “Affect function” substitutions (**Supplementary Figure 5** and **Supplementary Table 8**). However, substitutions at the C-terminus of the predicted GLP-1 peptides in the *Myotis brandtii* (Brandt’s bat) and *Myotis lucifugus* (little brown bat) are suggested to impact proteolytic processing when tested with NeuroPred (61) (**Supplementary Figure 5**), although neither SIFT (67) nor PROVEAN (68) suggested these were “Deleterious” or “Affect function” substitutions (**Supplementary Table 8**). Intriguingly, except for the highly divergent *Ornithorhynchus anatinus* (platypus) (22) sequence, the GLP-1 peptide sequences from these two bats, along with that from another bat, *Pipistrellus kuhlii* (Kuhl’s pipistrelle), have accumulated the largest number (6) of amino acid substitutions, although none of these substitutions are predicted to “Affect function” or be “Deleterious” (**Supplementary Figure 5** and **Supplementary Tables 6**–**8**). Glucagon can be proteolytically processed by N-arginine dibasic convertase (NRDc) + aminopeptidase-B to yield miniglucagon (Glucagon_19-29_) (14, 78, 79). This processing requires a pair of basic residues (at glucagon residues 17 and 18). Inspection of the Gcg sequences shows that three species, *O. anatinus* (platypus), *M. javanic*a (Malayan pangolin), *M. pentadactyl* (Chinese pangolin), and *C. lanigera* (Long-tailed chinchilla) have substitutions at R18 (to isoleucine (I) in platypus, leucine (L) in the pangolins, and tyrosine (Y) in the chinchilla) that should prevent production of miniglucagon in these species (**Supplementary Figure 5** and **Supplementary Table 8**).

SIFT (67) and PROVEAN (68) predict the effects of a single substitution upon a sequence. The Gcg sequences examined here have had multiple substitutions, thus additional substitutions might have occurred that compensate for the impact of an amino acid substitution. To examine this, I used Bayesian Graphical Models (BGM) (69) to identify potentially co-evolving sites, pairs of sites that accepted substitutions on the same lineage. A total of 23 pairs of sites were identified in the Gcg protein sequence with a posterior probability of at least 50% of having co-evolved substitutions (**Supplementary Table 9**). Of these 23 pairs of sites, 14 include at least one site that was identified by at least one mutation testing method as being “Deleterious” or “Affect function”, of which 5 pairs have both sites containing such a substitution, including one pair that has substitutions at both sites that were identified as both “Deleterious” and “Affect function” (**Supplementary Tables 8** and **9**). Of the potentially co-evolving sites, 3 are within the glucagon and OXM sequences, 4 within glicentin (3 of which overlap with glucagon and OXM), 4 within GLP-1, and 1 within GLP-2 (**Supplementary Table 9**). Major proglucagon fragment (MPGF) had the most pairs, with 9, as expected for the proglucagon-derived peptide with the longest length. Of these 9 pairs of sites within glicentin (including glucagon and OXM), GLP-1, and GLP-2, 5 include a site with an amino acid substitution considered to be “Deleterious” or “Affect function”, with 1 pair [Gcg residues 73 and 81 (glucagon residues 23 and 29)] having both sites identified as “Deleterious” and “Affect function” (**Supplementary Table 9**). This raises the possibility that some substitutions that were identified as “Deleterious” or “Affect function” by PROVEAN (68) or SIFT (67) might be compensated by changes at co-evolving sites.

### Variability in Receptors for Proglucagon-Derived Peptides

Since differences in evolutionary constraints acting upon glucagon, GLP-1, and GLP-2 were seen within mammals, one might also expect differences in the evolutionary constraints acting upon their receptors. Genes encoding the glucagon (*Gcgr*), GLP-1 (*Glp1r*), and GLP2 (*Glp2r*) receptors are all reactively closely related G protein-coupled receptors that originated *via* gene duplication events near the origin of vertebrates (35–37). An early study examining sequences of the glucagon (*Gcgr*), glucagon-like peptide-1 (*Glp1r*), glucagon-like peptide-2 (*Glp2r*), and glucose-dependent insulinotropic peptide (*Gipr*) receptor genes in diverse vertebrates suggested that *Glp1r* had experienced the strongest purifying selection (35). A recent study in rodents concluded that *Gcgr*, *Glp1r*, and *Glp2r* experience significantly different levels of selection pressure (as measured by d_N_/d_S_ ratios), with *Glp1r* experiencing the stronger purifying selection, *Glp2r* the least, and *Gcgr* an intermediate amount (42). To determine whether a similar pattern is observed in other orders of mammalian species I used RELAX (73), which calculates d_N_/d_S_ ratios on specific lineages, to test for intensification or relaxation of selective constrains between sets of receptor coding sequences. For this analysis, I selected only those species that had full length coding sequences for all three genes, and of those, I only compared sequences for mammalian orders that had at least 3 species (see **Supplementary Tables 3**–**5**). A total of 74 species from 7 mammalian orders were used in this analysis, with 21 species from Primates, 21 Rodents, 11 Carnivores, 8 Cetaceans, 7 Artiodactyls, 3 Chiropterans, and 3 Marsupials (**Supplementary Tables 3**–**5**). A trimmed codon-based alignment all 222 receptor coding sequences (74 species, 3 genes from each) was first generated. This alignment was then trimmed to only include sequences from one order of mammals before pairwise testing (i.e., *Gcgr versus Glp1r, Gcgr versus Glp2r*, and *Glp1r versus Glp2r*) with RELAX (73). The average d_N_/d_S_ ratios for the coding sequence in each pair were then tested to see if they were significantly different, to identify if selection was intensified or relaxed (73). When the rodent sequences were examined (**Table 3**), significant differences in the d_N_/d_S_ ratio were seen for each comparison, with *Glp1r* displaying the strongest selective constraint and *Glp2r* the weakest, in agreement with a previously report (24). Significant differences in the d_N_/d_S_ ratio for each receptor gene was also seen in Primates and Artiodactyla (**Table 3**). While the same pattern was seen for Carnivores and Chiroptera, only *Glp1r* was found to be under significantly greater constraint, while the difference between *Gcg*r and *Glp2r* was not significantly different (**Table 3**). In Cetacea all three receptor genes display similar d_N_/d_S_ ratios, with no significant difference between their values, while in Marsupials a different pattern was seen with *Gcgr* displaying significantly greater constraint compared to both *Glp1r* and *Glp2r* (**Table 3**).

**Table 3:** Selective constraints (d_N_/d_S_) acting on *Gcgr*, *Glp1r*, and *Glp2* in different mammalian orders.

Order^a^	*Gcgr*	*Glp1r*	*Glp2r*	P^b^
Rodents (21)				
	0.0972	0.0504		0.0000^c^
	0.0973		0.2463	0.0000^c^
		0.0504	0.2463	0.0000^c^
Primates (21)				
	0.1623	0.0972		0.0014^c^
	0.1621		0.2350	0.0036^c^
		0.0971	0.2341	0.0000^c^
Carnivores (11)				
	0.1267	0.0743		0.0145^c^
	0.1267		0.1705	0.1160
		0.0743	0.1700	0.0001^c^
Cetaceans (8)				
	0.1509	0.1535		0.9831
	0.1507		0.1630	0.1858
		0.1534	0.1629	0.3604
Artiodactyls (7)				
	0.1248	0.0708		0.0241^c^
	0.1235		0.2584	0.0001^c^
		0.0701	0.2582	0.0000^c^
Chiroptera (3)				
	0.2261	0.0873		0.0013^c^
	0.2220		0.2986	0.1012
		0.0864	0.2983	0.0000^c^
Marsupials (3)				
	0.0885	0.2712		0.0001^c^
	0.0885		0.3250	0.0002^c^
		0.2722	0.3284	0.3937

a Mammalian order, with number of species used in each order in brackets.

b Probability that the d_N_/d_S_ ratios are the same for the two tested coding sequences.

c Significantly different, P < 0.05.

**Figure 3:**
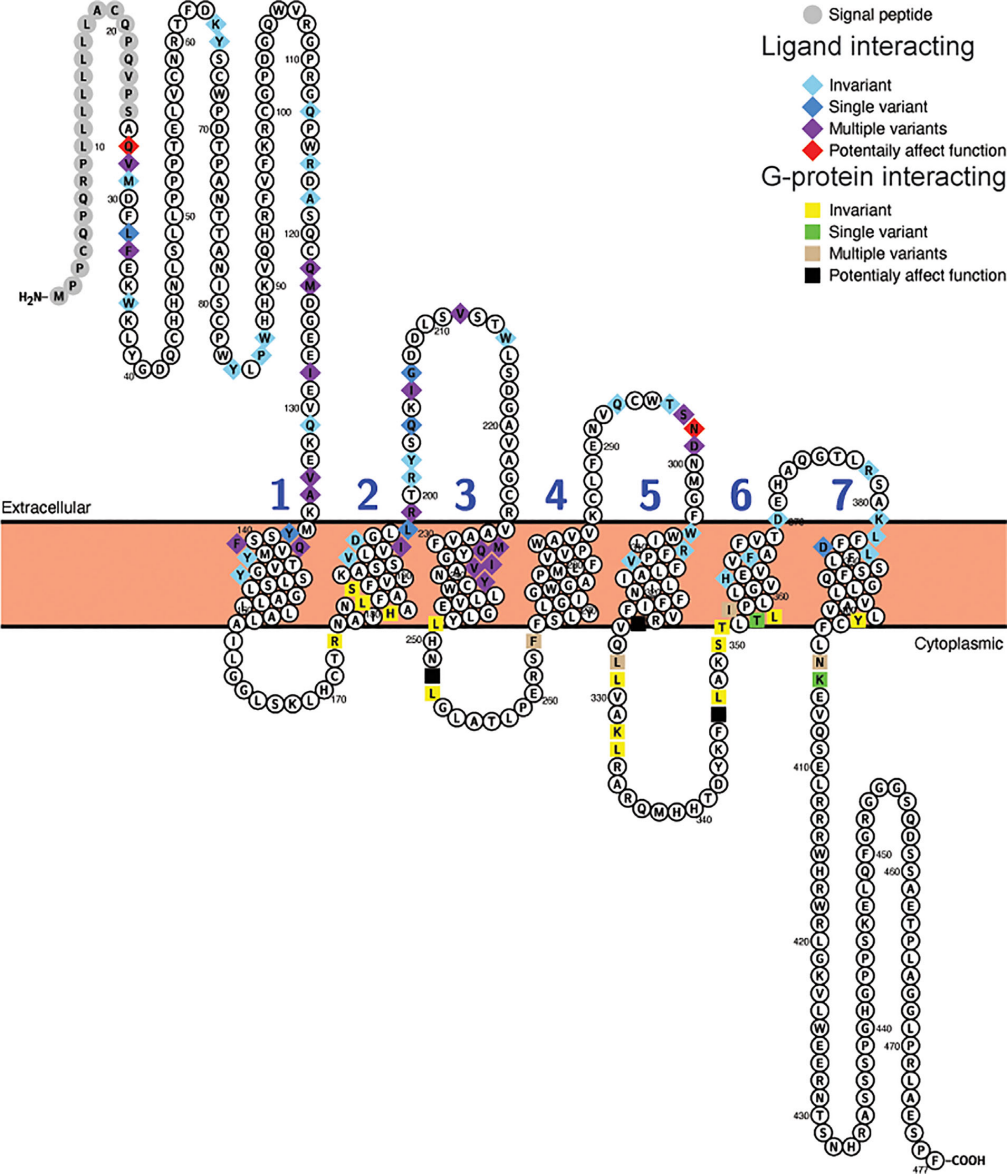
Variability in ligand and G-protein interacting sites in mammalian glucagon receptors (Gcgr). Snake plot of the Gcgr protein sequences (Uniprot accession: GLR_HUMAN) generated with Protter (wlab.ethz.ch/protter/start/) (71). Extracellular regions are shown at the top, cytoplasmic regions at the bottom, and transmembrane domains between the two lines. Signal peptides and transmembrane domains are extracted from the Uniprot accession files for the proteins by Protter. Signal peptide resides are shown in gray. The transmembrane domains are numbered (1-7). Ligand interacting residues are shown as diamonds and G-protein interacting sites as squares. Variability in the ligand and G-protein interacting residues in the sequences from 78 mammalian species are displayed in different colors with light blue diamonds and yellow squares being invariant, dark blue diamonds and green squares only have one species varying, purple diamonds and tan squares representing sites that either vary in multiple species and/or have multiple amino acid states, while red diamonds and black squares identify sites that have a substitution that was predicted to “Affect function” by SIFT (67) and be “Deleterious” by PROVEAN (68).

The differences in selective constraint acting upon sequences for the receptor for proglucagon-derived peptides described above was seen when the complete receptor coding sequences were used for these analyses, however, only a small portion of the receptor protein sequence is involved in hormone interaction with G proteins necessary for downstream signaling. To determine whether the evolutionary constraints acting upon hormone and G protein interacting sites has changed within mammals, I examined the variability of amino acid residues at sites implicated in interacting with hormone ligands and G proteins in the Gcgr, Glp1r, and Glp2r protein sequences based on the alignments of the receptor protein sequences (**Supplementary Figures 6**–**8**). Hormone ligand and G-protein interacting sites were acquired from the GPCRdb (62, 63) with the ligand interacting sites selected from those derived from the structures of the co-crystallization of glucagon with GCGR (PDB code 6LMK) (64), GLP-1 with GLP1R (PDB code 6X18) (65), and glucagon with GLP2R (PDB code 7D68) (66). As no putative G-protein interacting sites for Glp2r are available, I used the sites that were homologous to those present in both GCRG and GLP1R. This analysis was limited to the 78 mammalian species that had complete intact *Gcgr*, *Glp1r*, and *Glp2r* coding sequences (see **Supplementary Tables 3**–**5**) to ensure equal representation of sequences in this analysis. Of the 59 amino acid sites in Gcgr suggested to interact with ligand (64), 28 show variation within mammals, although 7 of them were unique changes seen in only one species (**Figure 3**, **Table 4**, and **Supplementary Table 10**). Similarly, 8 of the 23 sites suggested to interact with G-proteins display variation, with 4 of them having variation in multiple species (**Figure 3**, **Table 4**, and **Supplementary Table 10**). However, far fewer sites, 2 at ligand interacting sites, with one being a unique substitution (N298H in *M. myotis* (greater mouse-eared bat)), and 3 at G-protein interacting sites, 2 being unique (L252V (Wootten numbering: 3.57) in *E. edwardii* (Cape elephant shrew) and R346P (Wootten numbering: 6.37) in *L. vexillifer* (Yangtze River dolphin)) have accepted substitutions that were predicated to “Affect function” by SIFT (67) and be “Deleterious” by PROVEAN (68), with these substitutions occurring on only a few lineages (**Figure 3** and **Supplementary Table 10**). The two remaining amino acid substitutions that were predicted to both “Affect function” and be “Deleterious” were Q27W, a substitution at a ligand interacting site that occurs in 7 species, 5 aquatic carnivores [*C. ursinus* (northern fur seal), *M. leonine* (southern elephant seal), *O. rosmarus* (Pacific walrus), *P. vitulina* (harbor seal), and *Z. californianus* (California sea lion)] and 2 rodents [*C. griseus* (Chinese hamster) and *G. surdaster* (African woodland thicket rat)], indicating that this substitution has occurred at least twice, and the I325V substitution, at a G-protein interacting site, which is present in two species of Afrotheria [*E. edwardii* (Cape elephant shrew) and *O. afer* (aardvark)] (**Figure 3** and **Supplementary Table 10**).

**Table 4:** Variability in ligand and G protein interacting sites in Gcgr, Glp1r, and Glp2r.

	Gcgr	Glp1r	Glp2r
**Ligand interacting**			
Number of sites^a^	59	55	43
Variable sites^b^	28 (7)	29 (10)	25 (3)
Number of sites with “Deleterious” and “Affect function” substitutions^c^	2	3	1
Number of substitutions^d^	58	45	66
Number of “Deleterious” and Affect function” substitutions^d^	2	4	1
**G protein interacting**			
Number of sites^a^	23	27	17
Variable sites^b^	8 (4)	10 (4)	4 (0)
Number of sites with “Deleterious” and “Affect function” substitutions^c^	3	2	0
Number of substitutions^d^	15	12	5
Number of “Deleterious” and “Affect function” substitutions^e^	3	2	0

a Number of sites identified as interaction with ligand or G-protein.

b Number of sites with one or more amino acid substitutions. Number in brackets is the number of sites that vary in only a single species.

c Number of sites with a substitution predicted by both SIFT (67) to “Affect function” and PROVEAN (68) to be “Deleterious”.

d Total number of different types of amino acids substitutions.

e Number of substitutions predicted by both SIFT to “Affect function” and PROVEAN to be “Deleterious”.

**Figure 4:**
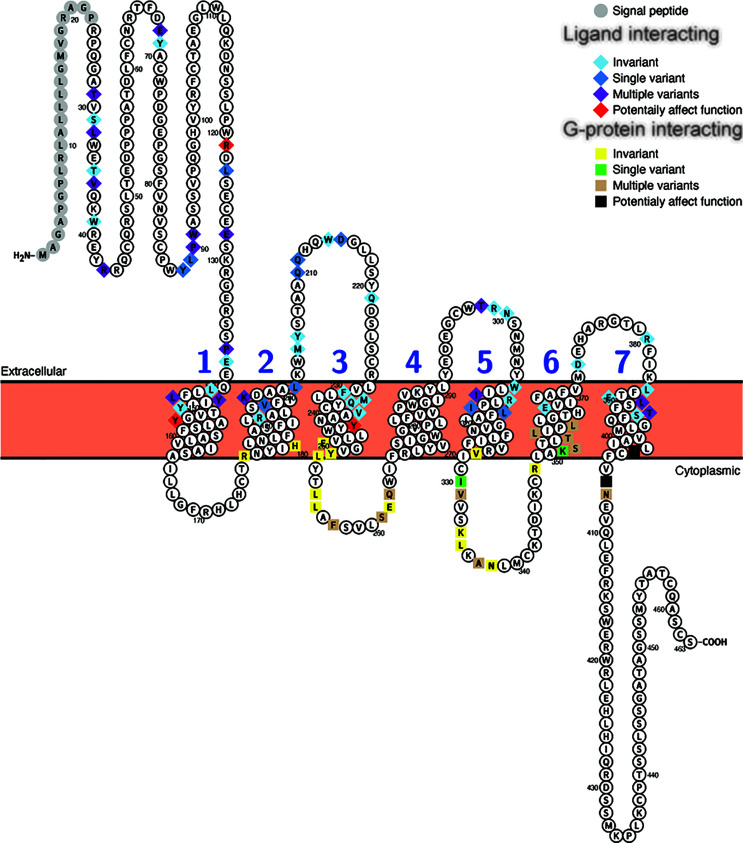
Variability in ligand and G-protein interacting sites in mammalian GLP-1 receptors (Glp1r). Snake plot of the Gcgr protein sequences (Uniprot accession: GLP1R_HUMAN) generated with Protter (wlab.ethz.ch/protter/start/) (71). Extracellular regions are shown at the top, cytoplasmic regions at the bottom, and transmembrane domains between the two lines. Signal peptides and transmembrane domains are extracted from the Uniprot accession files for the proteins by Protter. Signal peptide resides are shown in gray. The transmembrane domains are numbered (1-7). Ligand interacting residues are shown as diamonds and G-protein interacting sites as squares. Variability in the ligand and G-protein interacting residues in the sequences from 78 mammalian species are displayed in different colors with light blue diamonds and yellow squares being invariant, dark blue diamonds and green squares only have one species varying, purple diamonds and tan squares representing sites that either vary in multiple species and/or have multiple amino acid states, while red diamonds and black squares identify sites that have a substitution that was predicted to “Affect function” by SIFT (67) and be “Deleterious” by PROVEAN (68).

Similar results were seen with the Glp1r sequences, where 29 of the 55 putative ligand interacting sites (65) displayed variation, with 10 of these being unique to a single species, and 10 of the 27 G-protein interacting sites, with 4 being unique, being variable (**Figure 4**, **Table 4**, and **Supplementary Table 11**). Only 6 sites in the mammalian Glp1r sequences, 4 at ligand interacting sites (with 2 being unique, R121I and Y152H [Wooten numbering: 1.47) in *M. domestica* (opossum)] and 2 at G-protein interacting sites (with both being unique substitutions (Y402P (Wootten numbering: 7.57) and N406P (Wootten numbering: 8.47) that occur in *U. parryii* (Arctic ground squirrel)) (**Figure 4** and **Supplementary Table 11**) were predicted to both “Affect function” and be “Deleterious”. The two amino acid substitutions that both “Affect function” and be “Deleterious” and are found in multiple species were both at the same residue, Y241F and Y241C (Wootten numbering: 3.44) and were found in the sequences from a pair of artiodactyls [*C. ferus* (wild Bactrian camel) and *S. scrofa* (Pig)] and a pair of carnivores [*P. vitulina* (harbor seal) and *U. thibetanus* (Asiatic black bear)], respectively.

**Figure 5:**
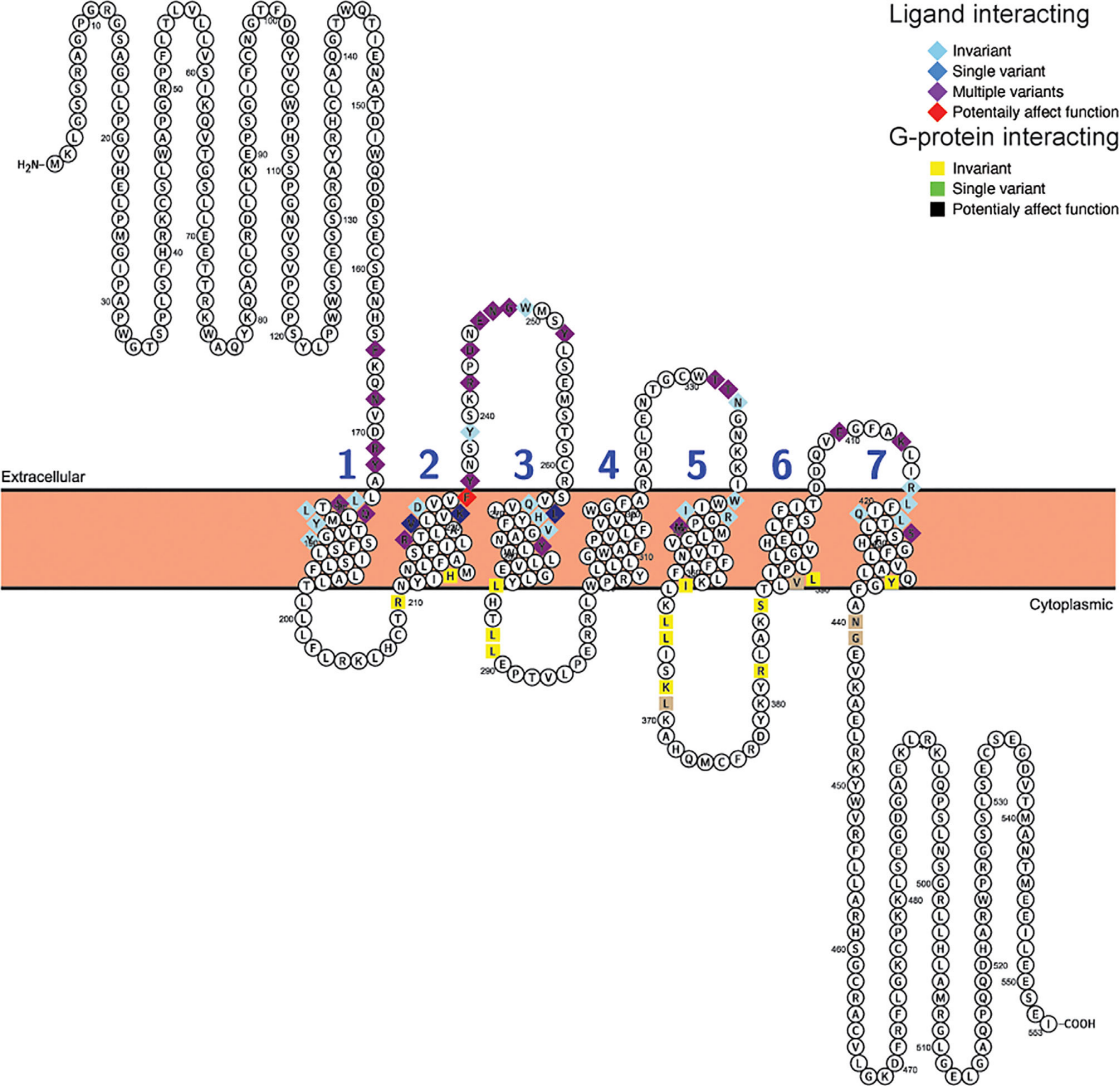
Variability in ligand and G-protein interacting sites in mammalian GLP-2 receptors (Glp2r). Snake plot of the Gcgr protein sequences (Uniprot accession: GLP2R_HUMAN) generated with Protter (wlab.ethz.ch/protter/start/) (71). Extracellular regions are shown at the top, cytoplasmic regions at the bottom, and transmembrane domains between the two lines. Transmembrane domains are extracted from the Uniprot accession files for the proteins by Protter. The transmembrane domains are numbered (1-7). Ligand interacting residues are shown as diamonds and G-protein interacting sites as squares. Variability in the ligand and G-protein interacting residues in the sequences from 78 mammalian species are displayed in different colors with light blue diamonds and yellow squares being invariant, dark blue diamonds only have one species varying, purple diamonds and tan squares representing sites that either vary in multiple species and/or have multiple amino acid states, while red diamonds identify sites that have a substitution that was predicted to “Affect function” by SIFT (67) and be “Deleterious” by PROVEAN (68).

For Glp2r sequences, 25 of the 43 mapped ligand interacting residues (66) were variable, with 3 being unique substitutions (V228I (Wootten numbering: 2.64) and L267V (Wooten numbering: 3.36) in *S. harrisii* (Tasmanian devil), and K231R [Wootten numbering: 2.67) in *L. vexillifer* (Yangtze River dolphin)], while 4 of the 17 putative G-protein interacting sites vary, with none being unique (**Figure 5**, **Table 4**, and **Supplementary Table 12**). Only 1 substitution, K235H (Wootten numbering: 2.67) was predicted to both “Affect function” and be “Deleterious” and was found in 5 species of aquatic carnivores [*C. ursinus* (northern fur seal), *M. leonine* (southern elephant seal), *O. rosmarus* (Pacific walrus), *P. vitulina* (harbor seal), and *Z. californianus* (California sea lion)].

To examine whether substitutions at ligand or G protein interacting sites were more prevalent in the sequences from specific species, the numbers of differences in these regions, compared to the human sequence, was determined for each species (**Supplementary Table 13**). To allow comparison among the three receptors, this analysis was limited to the 25 ligand interacting (see **Supplementary Tables 10**–**12** for sites) and 17 G-protein interacting sites that were shared by the GCGR and GLP1R sequences (64, 65). For Gcgr, a larger number of species were found to have substitutions at ligand-interacting (64) sites than at G protein-interacting sites (22) (**Supplementary Tables 10** and **13**). For G-protein interacting sites, very few (0-2) substitutions were accepted in most species, with only two species (both from Afrotheria, *E. edwardii* (Cape elephant shrew) and *O. afer* (aardvark)) displaying three substitutions (**Supplementary Tables 10** and **13**). Low levels of variation were also seen at the ligand interacting sites in Gcgr, with only *M. myotis*, with 6 substitutions, having more than 4 (**Supplementary Table 13**). When the Glp1r sequences were examined, it was found that the sequences generally have few substitutions than Gcgr sequences, with only 16 varying at ligand interacting and 11 at G-protein interacting sites (**Supplementary Table 13**). A few species, although, have larger numbers of substitutions at the ligand (7 substitution in both *P. cinereus* (koala) and *S. harrasii* (Tasmanian devil) and 6 in *M. domestica* (Gray short-tailed opossum)) and G-protein (3 in *U. parryii* (Arctic ground squirrel)) interacting sites (**Supplementary Tables 10** and **13**). The substitutions seen at the G-protein interacting sites in *U. parrayii* (Arctic ground squirrel) might be due to a sequence error as they are clustered in a region that has a large divergence from all other sequences (see **Supplementary Figure 7**). Only slightly increased numbers of species display variation at ligand interacting (62) and at G-protein interacting (30) sites in the Glp2r compared to Gcgr sequences (**Supplementary Table 13**). However, the number of changes at these sites is increased compared to both the Gcgr and Glp1r sequences. For the ligand interacting sites, 14 species have 5 or more differences compared to the human Glp2r sequence, while only 3 differ by this amount for human Glp1r and only 1 from human Gcgr (**Supplementary Table 13**). This is also seen at G-protein interacting sites, where 3 species (all mice, *M. caroli* (Ryukyu mouse), *M. musculus* (house mouse), and *M. splicilegus* (steppe mouse)), have 4 substitutions, while no species have this amount of change in their Gcgr or Glp1r sequences (**Supplementary Table 13**).

As with the sequences for the proglucagon derived-peptides, compensating substitutions can also occur within the receptor sequences, at both the ligand and G-protein interacting sites. I used BGM (69) to identify potentially co-evolving codons in the *Gcgr*, *Glp1r*, and *Glp2r* sequences (**Supplementary Tables 14**–**16**). BGM identified 68, 33, and 64 pairs of codons that show evidence of co-evolution in *Gcgr*, *Glp1r*, and *Glp2r*, respectively, with a Bayesian probability of at least 50% (**Supplementary Tables 14**–**16**). While some of the identified sites have been implicated in ligand or G-protein interactions, none of the potentially co-evolving pairs of codons in *Gcgr*, *Glp1r* or *Glp2r* involved a pair of sites that interact with ligand or G-proteins (**Supplementary Tables 14**–**16**). Compensating amino acid substitutions can also occur between a ligand and ligand interacting residues in a receptor. To determine whether co-evolving had potentially occurred between codons in *Gcg* and genes for receptors for proglucagon-derived peptides (i.e., *Gcg*r, *Glp1r*, and *Glp2r*), I used BGM (69) with concatenated sequences of Gcg and a receptor from 77 species that had intact sequences for all three receptors (*Gcgr*, *Glp1r*, and *Glp2r*) and *Gcg*. This analysis revealed that 12, 12, and 11 pairs of codons potentially co-evolved, with a Bayesian posterior probability of at least 50%, between *Gcg* and *Gcgr*, *Glp1r*, and *Glp2r*, respectively (**Supplementary Tables 17**–**19**). However, no evidence for co-evolution between a proglucagon-derived peptide and a ligand interacting site in a receptor was found.

## Discussion

### Conservation of *Gcg*, *Gcgr*, *Glp1r*, and *Glp2r* in Mammals

Duplication and loss of genes is a frequent occurrence (80, 81) that leads to differences in the number of copies of a gene even among closely related species. Even genes that have central roles in the regulation of blood glucose levels can vary in number within mammals. For example, some rodents, such as mice (*Mus musculus*) and rats (*Rattus norvegicus*), have two copies of the insulin (*Ins*) gene, while other mammals only have single copy of this gene (21, 42, 45, 82). Variation also occurs in the numbers of copies of genes for other hormones in mammals such as growth hormone (*Gh*), prolactin (*Prl*), relaxin (*Rln*), insulin-like peptides (*Insl*), and resistin-like (*Retnl*) (83–86). Previous work has shown that duplications of *Gcg* and *Gcgr* and deletion of *Glp1r* have occurred on some vertebrate lineages (30, 35, 37, 39, 40). The *Gcg* gene has even shown internal duplication and deletion of exons to result in genes that encode variable numbers of glucagon-like sequences (15–20, 30). To determine whether these types of events occur to the *Gcg*, *Gcgr*, *Glp1r*, and *Glp2r* in mammals, here I searched for and characterized these genes in 168 mammalian genomes.

*Gcg*, *Gcgr*, *Glp1r*, and *Glp2r* genes have been found in almost all mammalian genomes examined to date, with no evidence that any of these genes have been pseudogenized (**Supplementary Tables 2**–**5**). In addition, no changes in the structure of the *Gcg* gene was found, with no evidence for duplication or loss of exons that encode glucagon-like hormone sequences (**Supplementary Table 2** and **Supplementary Figure 5**). Thus, the retention of the exons encoding the three hormones (glucagon, GLP-1, and GLP-2) encoded by *Gcg*, as well as the genes for their receptors (*Gcgr*, *Glp1r*, and *Glp2r*) implies that all three hormones have essential functions in mammals. The failure to identify extra hormone encoding exons in the *Gcg* gene indicates that no new glucagon-like sequence hormone has evolved in mammals. However, it must be recognized that most current genome assemblies are incomplete and are continually being improved (87). In addition, the number of available genomes continues to increase (88). Current genomes are often derived from single individuals, thus may contain rare alleles that might not be biologically significant. In addition, errors exist in most genome assemblies, thus, the identification of a sequence that contains a unique mutation or amino acid substitution must be treated with caution. Many of the unique substitutions reported here may not represent biologically significant variants. The data presented here suggests that intact *Gcg*, *Gcgr*, *Glp1r*, and *Glp2r* genes exist in the genomes of all mammals. It also should be noted that the presence of the gene in a genome does not mean that it is expressed or regulated in the same way in all species, as changes may have occurred in regulatory sequences. I did not examine potential regulatory sequences here.

Conservation of these genes in mammals was also detected when their genomic neighborhoods were examined (76). By identifying genes adjacent to the *Gcg*, *Gcgr*, *Glp1r*, and *Glp2r* genes in diverse mammalian genomes I found no evidence for changes in the genomic neighborhoods for *Gcg*, *Gcgr*, and *Glp2r*, although, evidence for a chromosomal translocation near *Glp1r* was found in some rodents (**Figure 1** and **Supplementary Tables 1**–**4**). As changes in genome structure can lead to changes in expression, and regulation, of genes near these rearrangements (89), it is possible that the regulation of expression of *Glp1r* gene in species with this rearrangement differs from that of other mammals. Importantly, some of the species that have the altered gene neighborhood for *Glp1r* are mouse (*Mus musculus*) and rat (*Rattus norvegicus*), species that are important models for understanding the function of this gene and for the function of GLP-1 (1–4). Despite the general similarity in the gene expression pattern for *Glp1r* in mouse (*Mus musculus*) and human (*Homo sapiens*), the difference in the genomic neighborhood of these genes might result in some differences in the regulation of *Glp1r* between these two species.

Conservation of gene structure and genomic locations for most, if not all, *Gc*g, *Gcgr*, *Glp1r*, and *Glp2r* genes does not necessarily mean that new hormonal functions have not evolved in mammals. Indeed, the glucagon hormone from rodents of the suborder Hystricomorpha [e.g., the guinea pig (*Cavia porcellus*)] have been demonstrated to have reduced biological potency (21) and GLP-1 in the platypus (*Ornithorhynchus anatinus*) has been recruited to a component of its toxin (22). Changes in the sequences of hormones and their receptors can lead to changes in biological activity.

### Variation in the Sequences of Hormones Encoded by the Proglucagon Gene

Change in the function of a sequence is often associated with change in the rate of evolution of a sequence (90). Indeed, the change in the potency of glucagon in rodents of the suborder Hystricomorpha is associated with accelerated evolution of the glucagon amino acid sequence in this lineage (15, 21, 42). Apart from rodents of the suborder Hystricomorpha (e.g., guinea pig (*Cavia porcellus*) and degu (*Octodon degus*) with 4 differences from the human glucagon sequence), three other groups of mammals have glucagon peptide sequences that have accumulated considerable sequence difference from the human glucagon sequence: platypus (*Ornithorhynchus anatinus*) with 7 substitutions, the Malayan and Chinese pangolins (*Manis javanic*a and *Manis pentadactyla*) with 5 substitutions, and Brandt’s bat (*Myotis brandtii*) and little brown bat (*Myotis lucifugus*) with 3 substitutions (**Supplementary Table 6**). The platypus diverged earliest from all other mammals, approximately 180 million years ago (56, 77), thus is expected to have the greatest number of differences. Pangolins, bats, and rodents of the suborder Hystricomorpha all diverged from humans about 90 million years ago (56, 77), a time to that of most other mammals including other rodents, artiodactyls, cetaceans, and carnivores, the Gcg sequences from these three groups of species have accumulated a greater amount of divergence in their glucagon peptide sequences (**Supplementary Table 6** and **Supplementary Figure 5**) raising the possibility that the glucagon hormones in these species might have altered biological properties. For the bats, this is also supported by the observation that 2 of the 4 amino acid substitution (V23E and N28T; **Supplementary Table 8**) were predicted to affect protein function, however, none of the pangolin substitutions were confidently predicted to have an effect. Of the 7 substitutions seen in the platypus glucagon sequence, 2 (D21Q and T29Y; **Supplementary Table 8**) were predicted to affect protein function, thus it might also have altered biological activity, which might be an adaptation to the change in GLP-1 activity in this species (22).

As mentioned above, platypus (*Ornithorhynchus anatinus*) GLP-1_7-37_ is a component of the toxin produced by this species (22) and as previously reported (15, 22) its sequence has accumulated a large number (12) of differences from the human sequence (**Supplementary Table 6** and **Supplementary Figure 5**). The only other mammals to have more than 1 amino acid difference from the human GLP-1_7-37_ sequence are three bats (Brandt’s bat (*Myotis brandtii*), little brown bat (*Myotis lucifugus*), and Kuhl’s pipistrelle (*Pipistrellus kuhlii*)), which each having 6 substitutions (**Supplementary Table 6** and **Supplementary Figure 5**). Surprisingly, none of the substitutions found in these 3 bat GLP-1 sequences were confidently predicted to affect function (**Supplementary Table 8**), however, the Gcg precursor protein sequences from two of these species (Brandt’s bat (*Myotis brandtii*) and little brown bat (*Myotis lucifugus*)) contain amino acid substitutions at proteolytic processing sites (**Supplementary Figure 5**) that likely impair GLP-1_7-37_ production and lead to reduced levels of circulating GLP-1. Intriguingly, the two bats with changes at the proteolytic processing site (Brandt’s bat (*Myotis brandtii*) and little brown bat (*Myotis lucifugus*)) also have glucagon sequences with increased divergence, which might represent a compensation for change GLP-1 activity. Greater variability is seen in the GLP-1_1-37_ sequence (**Supplementary Figure 5** and **Supplementary Tables 6** and **7**). Despite the perfect conservation of the GLP-1_7-37_ sequence, multiple substitutions are observed in the N-terminal extension of the GLP-1_1-37_ sequences from Afrotheria (i.e., *Chrysochloris asiatica* (Cape golden mole), *Echinops telfairi* (Small Madagascar hedgehog), *Elephantulus edwardii* (Cape elephant shrew), *Loxodonta africana* (African elephant), *Ofer afer* (aardvark) and *Trichechus manatus* (Florida manatee)) (**Supplementary Figure 5** and **Supplementary Table 8**). These substitutions include changes of the N-terminal histidine (H) residue of GLP-1_1-37_ to asparagine (N) in *Loxodonta africana* (African elephant) and *Ofer afer* (aardvark) and glutamine in *Elephantulus edwardii* (Cape elephant shrew). Whether these substitutions impact the processing of Gcg to generate GLP-1_7-37_ is unknown. As GLP17-37 has roles in the stimulation of insulin release (3, 7), it is intriguing that amino acid substitutions have been identified in the proinsulin sequences of several species of Afrotheria that might prevent the production of the typical mammalian 2-chain insulin molecule (45). Whether then change in GLP-11-37 are a response to the changes in insulin or vice versa in these species needs further study.

Of the three major proglucagon-derived peptides, mammalian GLP-2 peptide sequences have accumulated a greater number of differences from the human sequence compared to glucagon and GLP-1 with the sequences from most mammals differing from the human sequence (**Table 2**, **Supplementary Table 6**, and **Supplementary Figure 5**). As expected, the platypus (*Ornithorhynchus anatinus*) sequence is most different, with many sequences from other species accumulating 5 or more amino acid substitutions. In agreement with previous analyses (15), mammalian GLP-2 peptide sequences have accumulated the largest numbers of substitution, measured either as substitutions per site or number of sites accepting substitutions (**Table 2**, **Supplementary Figure 5**, and **Supplementary Table 8**). Despite the large number of amino acid substitutions found in the GLP-2 sequences, none were confidently predicted to affect peptide function (**Supplementary Table 8**). These observations suggest that at least from the peptide perspective, there is little evidence to suggest the GLP-2 has acquired a new function in any mammal, except possibly the highly divergent platypus (*Ornithorhynchus anatinus*) sequence.

Comparisons among the three proglucagon-derived peptides showed that GLP-2 is most variable yet shows the least evidence for change in function within mammals (**Table 2**, **Supplementary Figure 5**, and **Supplementary Tables 7** and **8**). This suggests that the roles of GLP-2 in the promotion of cell growth, and improving digestive, absorptive, and barrier functions of cells in the intestine (9) are likely conserved within most mammals, while the roles of glucagon in regulating blood glucose (6) and GLP-1 as incretin hormone (1–4) have tolerated greater changes with mammals adapting to diverging habitats, which includes food sources. Changes in the sequences of several genes involved in metabolism, including glucokinase regulatory protein (*Gckr*) (43), insulin-like peptide 5 (*Insl5*) (44), and insulin (*Ins*) (45) have previously been linked to changes in diet in mammals, thus it should not be surprising that the hormones encoded by the *Gcg* gene might be influenced as well. Change in the hormone is only part of the story. For hormones to function, they need a receptor, and changes in receptors can lead to change in hormone function.

Gcg is processed into additional proglucagon-derived peptides, including glicentin, OXM, GRPP, IP-1, IP-2, and MPGF (see **Figure 2**) (1, 2, 4). The largest number of differences are seen in MPGF (major proglucagon fragment), although this should be expected as this is the longest proglucagon-derived peptide (**Supplementary Tables 6** and **7**). When adjusted for sequence length, the glicentin and GRPP (which is a component of glicentin) show the greatest variability (**Supplementary Table 7**). This might suggest that the GRPP sequences, including those in glicentin, have lower biological importance. IP-1 (intervening peptide-1), which is included in the OXM and glicentin sequences, shows levels or variation like those of glucagon, suggesting that they retain biological function (**Supplementary Table 7**). Miniglucagon, a shorter proteolytic product of glucagon (glucagon_19-29_) (14), displays sequence variation like glucagon (**Supplementary Figure 5** and **Supplementary Tables 6** and **7**). However, three species, *O. anatinus* (platypus), *M. javanic*a (Malayan pangolin), *M. pentadactyl* (Chinese pangolin) and *C. lanigera* (Long-tailed chinchilla, have amino acid substitutions in their Gcg sequences (R69I (platypus), R69L (pangolins), and R69Y (chinchilla)) that likely prevent production of this peptide (**Supplementary Figure 5**). These results parallel a recent study of *GCG* missense mutations found in human populations (91), where fewer missense mutations were found at sites corresponding to the glucagon, GLP-1 and GLP-2 peptides. In this report, which looked at sequences from ~450,000 people, examined mutations that can occur (and may exist in only one individual), while examining diverse mammals identified mutations that likely have been accepted by evolution.

### Variation in the Sequences of Receptors for Hormones Encoded by the Proglucagon Gene

Each of the proglucagon-derived peptides has a cell surface receptor (34), however, the specificity of these receptors can change. Indeed, there is an example of a glucagon receptor acquiring the ability to bind, and be activated by, a different ligand. Teleost fish have lost their *Glp1r* gene (17, 35, 36, 39), yet GLP-1 has a physiological function (38). In these fish GLP-1 and glucagon have overlapping functions and this is due to changes in a glucagon receptor encoded by one of the duplicated *Gcgr* genes in these species that have acquired the ability to bind and be activated by GLP-1 (17, 40, 41). Thus, the presence of single copy *Gcgr*, *Glp1r*, and *Glp2r* genes in likely all mammals, does not necessarily mean that each species has a single receptor for each of the proglucagon-derived peptide hormones.

Previous analyses had suggested that *Glp1r* was under greater selective constraint that *Gcgr* or *Glp2r*, although these conclusions were derived from alignments of receptor coding sequences from a smaller number of vertebrate species (35), or just within rodents (42). Here, by analyzing coding sequences from diverse orders of mammals (**Table 3**) I show that this pattern is not universal. While primates and artiodactyls were like rodents in having *Glp1r* experiencing significantly stronger selection pressure and *Glp2r* have significantly lower selection pressure, in marsupials (e.g., opossum (*Monodelphis domestica*)) *Gcgr* was found to be experiencing the strongest selection pressure and no difference in selection pressure was found for the three genes in Cetacea (e.g., killer whale (*Orca orca*)) (**Table 3**). These results suggest that the selective constraints acting on *Gcgr*, *Glp1r*, and *Glp2r* vary among mammalian orders, which implies that the signaling pathways involving glucagon, GLP-1, and GLP-2 in species of these orders may have differing importance.

Amino acid substitutions can affect the function of receptors in many ways. Here I only examined sites that had previously been implicated in interactions with ligand and G-proteins as they are better defined. It is possible that other substitutions in these sequences can affect the localization and activation, among other properties, of these receptors. When residues shown to be important for ligand and G protein interaction were examined (**Supplementary Tables 10**–**13** and **Supplementary Figures 6**–**8**) very little evidence for lineage-specific amino acid substitutions at these sites were found, although there were some species that exhibited larger numbers of changes at these sites in Gcgr and Glp1r (e.g., *Myotis myotis* (Greater mouse-eared bat), 6 substitutions in Gcgr; *Phascolarctos cinereus* (Koala) and *Sarcophilus harrisii* (Tasmanian devil), 7 substitutions in Glp1r; **Supplementary Table 13**). Greater variability was seen in Glp2r, with 14 of 77 examined species displaying 5 or more amino acid substations in the shared ligand interacting sites, while only 1 and 3 species, for Gcgr and Glp1r, respectively, showed this amount of change (**Supplementary Table 13**). The increased levels of Glp2r variation appears to correlate with the increased levels of GLP-2 sequence variation (**Supplementary Tables 6** and **13**), suggesting that rapidly evolving ligands and receptors co-evolve. Very few amino acid substitutions at ligand or G protein interacting sites were predicted to affect function, which suggests that the ligand binding and signaling functions of these receptors have been conserved within mammals. Comparison among receptor suggested that mammalian Glp2r sequences are more receptive of accepting amino acid substitutions (**Supplementary Tables 6** and **9**).

## Conclusion

Characterization of *Gcg*, *Gcgr*, *Glp1r*, and *Glp2r* genes from mammalian species with annotated genomes demonstrate that they have all been retained in the genomes and have been maintained in conserved genomic neighborhoods in most species. This suggests that the functions of the hormones are essential in these species, and that the expression of these genes is likely conserved in mammals. However, it should be noted, the genomic neighborhood for *Glp1r* in some rodents, including mouse (*Mus musculus*), has experienced a chromosomal translocation that might influence the regulation of this gene. While the peptide sequences encoded by *Gcg* in most mammals are very strongly conserved, some species have accepted greater numbers of substations that might affect function. These include the previously characterized glucagon sequences from rodents of the suborder Hystricomorpha and GLP-1 from the platypus (*Ornithorhynchus anatinus*) (21, 22). In addition, I show that the glucagon sequences from the pangolins (*Manis javanic*a (Malayan pangolin) and *Manis pentadactyla* (Chinese pangolin)) and glucagon and GLP-1 from bats of the genera *Myotis* (*Myotis brandtii* (Brandt’s bat) and *Myotis lucifugu*s (little brown bat)) accumulated greater numbers of substitutions, and thus potentially have altered function. Changes in the Gcg protein sequences from bats of the genera *Myotis* might also affect processing of the precursor protein leading to changes in the relative abundance of some hormones. A parallel increase in number of substitutions at ligand interacting sites in Gcgr from *Myotis myotis* (Greater mouse-eared bat), another bat from the genera *Myotis*, was also seen These observations suggest that the functions of mammalian proglucagon-derived peptides might be more diverse than presently appreciated, and may offer opportunities, as seen in fish, for the discovery of peptides that might have beneficial uses (47, 48).

## Data Availability Statement

The original contributions presented in the study are included in the article/**Supplementary Material**. Further inquiries can be directed to the corresponding author.

## Author Contributions

DI conceived and drafted the manuscript. The author confirms being the sole contributor of this work and has approved it for publication.

## Conflict of Interest

The author declares that the research was conducted in the absence of any commercial or financial relationships that could be construed as a potential conflict of interest.

## Supplementary Material

Supplementary File 1 Fasta formatted proglucagon (*Gcg*) coding sequences.

Supplementary File 2 Fasta formatted glucagon receptor (*Gcgr*) coding sequences.

Supplementary File 3 Fasta formatted glucagon-like peptide-1 (GLP-1) receptor (*Glp1r*) coding sequences.

Supplementary File 4 Fasta formatted glucagon-like peptide-2 (GLP-2) receptor (*Glp2r*) coding sequences.

Supplementary File 5 Treefile for the phylogeny of mammalian proglucagon (*Gcg*) coding sequences.

Supplementary File 6 Treefile for the phylogeny of mammalian glucagon receptor (*Gcgr*) coding sequences.

Supplementary File 7 Treefile for the phylogeny of mammalian glucagon-like peptide-1 (GLP-1) receptor (*Glp1r*) coding sequences.

Supplementary File 8 Treefile for the phylogeny of mammalian glucagon-like peptide-2 (GLP-2) receptor (*Glp2r*) coding sequences.
