# Supplementary material for: Variation in the Evolution and Sequences of Proglucagon and the Receptors for Proglucagon-Derived Peptides in Mammals
Source: Front Endocrinol (Lausanne). 2021 Jul 12;12:700066. doi: 10.3389/fendo.2021.700066 (PMC8312260; doi:10.3389/fendo.2021.700066)
Supplement: Supplementary File 1 — Fasta formatted proglucagon (Gcg) coding sequences. [file DataSheet_1.zip › Supplement/Read me.docx]

Files at this site are Supplementary Files, Figures, and Tables for the manuscript:

Variation in the evolution and sequences of proglucagon and the receptors for proglucagon-derived peptides in mammals

David M. Irwin

Frontiers in Endocrinology (submitted)

Below is a brief description of each file.

**Supplementary Files:**

**Supplementary File 1**. Fasta formatted proglucagon (*Gcg*) coding sequences.

Txt file with fasta formatted proglucagon (*Gcg*) coding sequences

**Supplementary File 2**. Fasta formatted glucagon receptor (*Gcgr*) coding sequences.

Txt file with fasta formatted glucagon receptor (*Gcgr*) coding sequences

**Supplementary File 3**. Fasta formatted glucagon-like peptide-1 (GLP-1) receptor (*Glp1r*) coding sequences.

Txt file with fasta formatted glucagon-like peptide-1 (GLP-1) receptor (*Glp1r*) coding sequences

**Supplementary File 4**. Fasta formatted glucagon-like peptide-2 (GLP-2) receptor (*Glp2r*) coding sequences.

Txt file with fasta formatted glucagon-like peptide-2 (GLP-2) receptor (*Glp2r*) coding sequences

**Supplementary File 5**. Treefile for the phylogeny of mammalian proglucagon (*Gcg*) coding sequences.

Txt tile of the tree file generated by IQtree [1] for mammalian *Gcg* coding sequences. The phylogenetic tree can be viewer using FigTree [2]

**Supplementary File 6**. Treefile for the phylogeny of mammalian glucagon receptor (*Gcgr*) coding sequences.

Txt tile of the tree file generated by IQtree [1] for mammalian *Gcgr* coding sequences. The phylogenetic tree can be viewer using FigTree [2]

**Supplementary File 7**. Treefile for the phylogeny of mammalian glucagon-like peptide-1 (GLP-1) receptor (*Glp1r*) coding sequences.

Txt tile of the tree file generated by IQtree [1] for mammalian *Glp1r* coding sequences. The phylogenetic tree can be viewer using FigTree [2]

**Supplementary File 8**. Treefile for the phylogeny of mammalian glucagon-like peptide-2 (GLP-2) receptor (*Glp2r*) coding sequences.

Txt tile of the tree file generated by IQtree [1] for mammalian *Glp2r* coding sequences. The phylogenetic tree can be viewer using FigTree [2]

**Supplementary Figures:**

**Supplementary Figure 1**. Phylogeny of mammalian proglucagon (*Gcg*) coding sequences.

PDF file of the phylogenetic tree of *Gcg* coding sequences. Treefile is available as Supplementary File 5.

**Supplementary Figure 2**. Phylogeny of mammalian glucagon receptor (*Gcgr*) coding sequences.

PDF file of the phylogenetic tree of *Gcgr* coding sequences. Treefile is available as Supplementary File 6.

**Supplementary Figure 3**. Phylogeny of mammalian glucagon-like peptide-1 (GLP-1) receptor (*Glp1r*) coding sequences.

PDF file of the phylogenetic tree of *Glp1r* coding sequences. Treefile is available as Supplementary File 7.

**Supplementary Figure 4**. Phylogeny of mammalian glucagon-like peptide-2 (GLP-2) receptor (*Glp2r*) coding sequences.

PDF file of the phylogenetic tree of *Glp2r* coding sequences. Treefile is available as Supplementary File 8.

**Supplementary Figure 5**. Alignment of mammalian proglucagon (Gcg) protein sequences.

Word document. Multiple sequence alignment of Gcg protein sequences.

**Supplementary Figure 6**. Alignment of mammalian glucagon receptor (Gcgr) protein sequences.

Word document. Multiple sequence alignment of Gcgr protein sequences.

**Supplementary Figure 7**. Alignment of mammalian glucagon-like peptide-1 (GLP-1) receptor (Glpr1) protein sequences.

Word document. Multiple sequence alignment of Glp1r protein sequences.

**Supplementary Figure 8**. Alignment of mammalian glucagon-like peptide-2 (GLP-2) receptor (Glp2r) protein sequences.

Word document. Multiple sequence alignment of Glp2r protein sequences.

**Supplementary Figures:**

**Supplementary Table 1**. Species and genome assemblies used for searches for proglucagon (*Gcg*), glucagon receptor (*Gcgr*), glucagon-like peptide-1 (GLP-1) receptor (*Glp1r*), and glucagon-like peptide-2 (GLP-2) receptor (*Glp2r*) genes.

Excel file. Contains names of mammalian species examined, their genome assemblies, and abbreviations used in other Supplementary Files, Figures, and Tables.

**Supplementary Table 2**. Genomic locations, accession numbers, and genomic neighborhoods for mammalian proglucagon (*Gcg*) genes.

Excel file. Contains *Gcg* genomic location, accession numbers, genomic neighborhoods, and Ensembl GOC and WGA scores.

**Supplementary Table 3**. Genomic locations, accession numbers, and genomic neighborhoods for mammalian glucagon receptor (*Gcgr*) genes.

Excel file. Contains *Gcgr* genomic location, accession numbers, genomic neighborhoods, and Ensembl GOC and WGA scores.

**Supplementary Table 4**. Genomic locations, accession numbers, and genomic neighborhoods for mammalian glucagon-like peptide-1 (GLP-1) receptor (*Glp1r*) genes.

Excel file. Contains *Glp1r* genomic location, accession numbers, genomic neighborhoods, and Ensembl GOC and WGA scores.

**Supplementary Table 5**. Genomic locations, accession numbers, and genomic neighborhoods for mammalian glucagon-like peptide-2 (GLP-2) receptor (*Glp2r*) genes.

Excel file. Contains *Glp2r* genomic location, accession numbers, genomic neighborhoods, and Ensembl GOC and WGA scores.

**Supplementary Table 6**. Number of differences of mammalian proglucagon-derived peptide sequences from human sequences.

Excel file. Numbers of amino acid substitutions, compared to the human sequences, in proglucagon-derived peptides of each mammalian species examined.

**Supplementary Table 7**. Variation in mammalian proglucagon-derived peptide sequences.

Excel file. Numbers of amino acid substitution in mammalian proglucagon-derived peptides

**Supplementary Table 8**. Predicted consequence of amino acid substitutions in mammalian proglucagon (Gcg) sequences.

Excel file. Predicted consequences of amino acid substitutions in the Gcg protein sequences predicted by SIFT [3] and PROVEAN [4].

**Supplementary Table 9**. Potentially co-evolving sites in proglucagon (Gcg) sequences identified using Bayesian Graphical Models (BGM).

Excel file. Potentially co-evolving sites in Gcg identified using BGM [5]

**Supplementary Table 10**. Predicted consequence of amino acid substitutions in mammalian glucagon receptor (Gcgr) sequences.

Excel file. Predicted consequences of amino acid substitutions in the Gcgr protein sequences predicted by SIFT [3] and PROVEAN [4].

**Supplementary Table 11**. Predicted consequence of amino acid substitutions in mammalian glucagon-like peptide-1 (GLP-1) receptor (Glp1r) sequences.

Excel file. Predicted consequences of amino acid substitutions in the Glp1r protein sequences predicted by SIFT [3] and PROVEAN [4].

**Supplementary Table 12**. Predicted consequence of amino acid substitutions in mammalian glucagon-like peptide-2 (GLP-2) receptor (Glp2r) sequences.

Excel file. Predicted consequences of amino acid substitutions in the Glp2r protein sequences predicted by SIFT [3] and PROVEAN [4].

**Supplementary Table 13**. Number of differences at ligand and G-protein interacting sites shared by glucagon receptor (Gcgr), glucagon-like peptide-1 (GLP-1) receptor (Glp1r), and glucagon-like peptide-2 (Glp2r) receptor in mammalian species.

Excel file. Numbers of amino acid substitutions in 36 ligand and 11 G-protein interacting sites shared by Gcgr, Glp1r, and Glp2r.

**Supplementary Table 14**. Potentially co-evolving sites in glucagon receptor (Gcgr) sequences identified using Bayesian Graphical Models (BGM).

Excel file. Potentially co-evolving sites in Gcgr identified using BGM [5]

**Supplementary Table 15**. Potentially co-evolving sites in glucagon-like peptide-1 (GLP-1) receptor (Glp1r) sequences identified using Bayesian Graphical Models (BGM).

Excel file. Potentially co-evolving sites in Glp1r identified using BGM [5]

**Supplementary Table 16**. Potentially co-evolving sites in glucagon-like peptide-2 (GLP-2) receptor (Glp2r) sequences identified using Bayesian Graphical Models (BGM).

Excel file. Potentially co-evolving sites in Glp2r identified using BGM [5]

**Supplementary Table 17**. Potentially co-evolving sites between proglucagon (Gcg) and glucagon receptor (Gcgr) sequences identified using Bayesian Graphical Models (BGM).

Excel file. Potentially co-evolving sites between Gcg and Gcgr identified using BGM [5]

**Supplementary Table 18**. Potentially co-evolving sites between proglucagon (Gcg) and glucagon-like peptide-1 (GLP-1) receptor (Glp1r) sequences identified using Bayesian Graphical Models (BGM).

Excel file. Potentially co-evolving sites between Gcg and Glp1r identified using BGM [5]

**Supplementary Table 19**. Potentially co-evolving sites between proglucagon (Gcg) and glucagon-like peptide-2 (GLP-2) receptor (Glp2r) sequences identified using Bayesian Graphical Models (BGM).

Excel file. Potentially co-evolving sites between Gcg and Glp2r identified using BGM [5]

**References:**

[1] <http://www.iqtree.org/>

Minh BQ, Schmidt HA, Chernomor O, D. Schrempf D, Woodhams DM, von Haeseler A, Lanfear R. IQ-TREE 2: New models and efficient methods for phylogenetic inference in the genomic era. *Mol Biol Evol* (2020) 37:1,530-4. doi: 10.1093/molbev/msy096.

[2] FigTree 1.4.4 <http://tree.bio.ed.ac.uk/software/figtree/>

[3] <http://provean.jcvi.org/seq_submit.php>

Kumar P, Henikoff S, Ng PC. Predicting the effects of coding non-synonymous variants on protein function using the SIFT algorithm. *Nature Protoc* (2009) 4:1,073-81. doi: 10.1038/nprot.2009.86.

[4] <https://sift.bii.a-star.edu.sg/www/SIFT_seq_submit2.html>

Choi Y, Chan AP. PROVEAN web server: a tool to predict the functional effect of amino acid substitutions and indels. *Bioinformatics* (2015) 31:2,745-27. doi: 10.1093/bioinformatics/btv195.

[5] <http://www.datamonkey.org/bgm>

Poon AF, Lewis FI, Frost SD, Kosakovsky Pond SL. Spidermonkey: rapid detection of co-evolving sites using Bayesian graphical models. *Bioinformatics* (2008) 24:1949-1950. doi: 10.1093/bioinformatics/btn313.
