# Supplementary material for: Variation in the Evolution and Sequences of Proglucagon and the Receptors for Proglucagon-Derived Peptides in Mammals
Source: Front Endocrinol (Lausanne). 2021 Jul 12;12:700066. doi: 10.3389/fendo.2021.700066 (PMC8312260; doi:10.3389/fendo.2021.700066)
Supplement: Supplementary File 1 — Fasta formatted proglucagon (Gcg) coding sequences. [file DataSheet_1.zip › Supplement/Suplementary Figures/Fig S8 Glp2r alignment.docx]

Signal peptide <<<

P P P

Monodelphis_domestica -MLPGVSSVV SGRTTEGPLP YSSAFLMDTS T---FQETKE LHSHKKLCFP VFAQYFLL-- -LILQLSVKP V-TGSLLKET TQKWLLYKKT CLIELQNRP

Phascolarctos_cinereus -....M.... ....A..L.T ....SP..AF I---L....K .....Q.Y.. A...C...-- -....F.... .-........ ....FQ..EK ......K..

Sarcophilus_harrisii -----M...M FE..-..L.L H..SSP..AF .---L..I.. ......IY.. A...C...-- -....FLA.. .-K....Q.. ....FE..QK ..K..EK..

Trichosurus_vulpecula -....M.... ....A..L.S ....SS..AF .---L...QK .......Y.. A...C..V-- -....F.... .-.....E.. ....FQ..EK .........

Vombatus_ursinus -....M.... ....A..L.S ....SP..AF I---P....K F.....FY.. A...C...-- -....F.... .-........ ....FQ..EK ..K...K..

Choloepus_didactylus -.RLEA.RTG ..ARPA.S.. GARGQ.EGIP .---PRG.RP APCPR.RSLQ APGRCV.A-- -.V.LF.IQQ .-........ .R..AQ..E. ..RD.LKE.

Dasypus_novemcinctus -.R.EA.RAR ..GG.G.C.. GVLGLPVGIP A---PRG.GP .PA.R.RPLQ AP.RLI.A-- -.M.LV.I.Q .-........ V...IE..NM .H.D.L.E.

Chrysochloris_asiatica -.RL.P.RAR P..LGAE... EAQRLSRG.P A---PRR.SP ....R..A.Q APVRP..A-- -.VFLV.INQ .-RA...ED. .R..AE..EM ..K..LKK.

Elephantulus_edwardii -.RT.P.GSG PAAPNGR... GAGRLQRS.P A---PRGPRP .P..R..VLQ PPLRP..V-- -.V.LV.I.Q .-..T..E.. ....AQ..EK ..KD.LQE.

Loxodonta_africana -.R..P.RAG P..RRP..P. GARGL.RA.P A---PRG.SP .P.....ALQ APLRP..A-- -.V.LV.I.Q .-.....E.. .R..AQ..EK ..RD.LKEA

Orycteropus_afer -.R..LNRAG P..RRAV... GAHGLR.G.P V---T.R.SP .P.YR..PLQ APVRP..T-- -.L.LV.I.Q .-.....EK. ....AQ..EK ..RD.LKE.

Trichechus_manatus -.R..P.RAG P..RRL.... GARGL.RC.P A---PRG.RP FP.....VLR APVRP..A-- -.V.LV.I.Q .-.....E.. .R..AE..EK ..RD.LKET

Balaenoptera_acutorostrata -.R.EP.RAG PRSRRV.... GAHTLPTGSP A---TRGARP PP.PG.RALW TP.RP..A-- -.L.LV.I.Q .-.....E.. .R..AQ..EK ..RD.LKET

Balaenoptera_musculus -.R.EP.RAG PRSRRV.... GAHTLPTGIP A---TRGARP PP.PG.RALW TP.RP..A-- -.L.LV.I.Q .-.....E.. .R..AQ..EK ..RD.LKET

Delphinapterus_leucas -.R.EPRGAG PRSRRV.... GAHTLSAGIP A---TRGARP PP.PG.RALW TPVRP..A-- -.L.LV.I.Q .-.....E.. .R..AQ..EK ..RD.LKET

Globicephala_melas -.R.EPRRAG PRSRRV.... GAHTVPAGIP A---TRGARP PP.PG.RALW TP.RP..A-- -.L.LV.I.Q .-.....E.. .R..AQ..EK ..RD.LKET

Lagenorhynchus_obliquidens -.R.EPRRAG PRSRRV.... GAHTVPAGVP A---TRGARP PP.PG.RALW TP.RP..A-- -.L.LV.I.Q .-.....E.. .R..AQ..EK ..RD.LKET

Lipotes_vexillifer -.R.EP.WAG PRSRRV.... GAHTLPTGIP A---TRGARP PP.PG.RALW TP.RP..A-- -.L.LV.I.Q .-.....E.. .R..AQ..EK ..RD.LKET

Monodon_monoceros -.R.EPRGAG PRSRRV.... GAHTLSAGIP A---TRGARP PP.PG.RALW TP.RP..A-- -.L.LV.I.Q .-.....E.. .R..AQ..EK ..RD.LKET

Neophocaena_asiaeorientalis -.R.EPRRAG PRSRRV.... GAHTLPAGIP A---TRGARP PP.PG.RALW TP.RP..A-- -.L.LV.I.Q .-.....E.. .R..AQ..EK ..RD.LKET

Orcinus_orca -.R.EPRRAG PRSRRV.... GAHTVPAGIP A---TRGARP PP.PG.RSLW TP.RP..A-- -.L.LV.I.Q .-.....E.. .R..AQ..EK ..RD.LKET

Phocoena_sinus -.R.EPRRAG PRSRRV.... GAHTLPAGIP A---TRGARP PP.PG.RALW TP.RP..A-- -.L.LV.I.Q .-.....E.. .R..AQ..EK ..RD.LKET

Tursiops_truncatus -.R.EPRRAG PRSRRV.... GAHTVPAGIP A---TRGARP PP.PG.RALW TP.RP..A-- -.L.LV.I.Q .-.....E.. .R..AQ..EK ..RD.LKET

Bison_bison -.R..P.WAG .R.RAVS... GAHVL..G.P A---A.GAGP .S.PH..AHW TP.RP..A-- -.L.LV.I.Q .-.....E.. .R..AQ..EK ..RD.LKET

Bos_mutus -.R..P.WAG .R.RAVS... GAHVL..G.P A---A.GAGP .S.PH..AHW TP.RP..A-- -.L.LV.I.Q .-.....E.. .R..AQ..EK ..RD.LKET

Bos_taurus -.R..P.WAG .R.RAVS... AAHVL..G.P A---A.GAGP .S.PR.PAHW TP.RP..A-- -.L.LV.I.Q .-.....E.. .R..AQ..EK ..RD.LKET

Bubalus_bubalis -.R..P.WAG AR.RAVS... GAHVL..G.P A---A.GAGP .S.PREPARW TP.RP..A-- -.L.LV.I.Q .-.....E.. .R..AQ..EK ..RD.LKET

Camelus_dromedarius -.R.PP.RAG P..RRQ.... RAHTLPSGI- P---AARALP SPGRR--ALW TP.RPLMA-- -.L.LV.I.Q .-.....E.. .R..AQ..EK ..RD.LKE.

Camelus_ferus -.R.PP.RAG P..RRQ.... RAHTLPSGI- P---AARALP SPGRR--ALS TP.RPLMA-- -.L.LV.I.Q .-.....E.. .R..AQ..EK ..RD.LKE.

Capra_hircus -.R..P.WAG .R.RAVS... GAHVL.TG.P A---A.GAGP .S.PC..ARW TP.RP..A-- -.L.L..I.Q .-.....Q.. .R..AQ..EK ..RD.LKET

Catagonus_wagneri -.R.DP.LAG P..RRA.LSR EAH.LPTGVP A---ARGARP PP.PG.RALR TPVRP.MA-- -.LFLV.I.Q .-.....E.. .R..AQ..EK ..KD.LKET

Cervus_hanglu -.R.EP.WAG .R.RAVS... GAHVLF...P A---TRGAGP .S.PR.PARW TP.RP..A-- -.LFLV.I.Q .-.....E.. .R..AQ..EK ..RD.LKET

Moschus_moschiferus -.R..P.WAG .Q.RAVS... GDHML..G.P A---A.GSGP .S.PCN.AHW TP.RP..A-- -.L.LV.I.Q .-.....E.. .R..AQ..EK ..RD.LKET

Sus_scrofa -.R.ER.RAG P..KRA.L.. EAHGLPAGIP A---AWGARP .P.PR.RAPR TPVRP.MA-- -.LFLV.I.Q .-.....E.. .R..AQ..EK ..KD.LKET

Vicugna_pacos -.R.PP.RAG P..RRQ.... RAH.LPSGI- P---ATRPLP SPGRR--TLW TPVRPLMA-- -.L.LV.I.Q .-A....E.. .R..AQ..EK ..RD.LKE.

Ailuropoda_melanoleuca ---------- ---------- ------...P A---ARGARP PSPCR.RSP. AL.RPMFVLL L.L.LVPI.Q .-.....E.. .R..AQ..EK ..RD.LKE.

Callorhinus_ursinus ---------- ---------- ------...P A---A.GARP PPRS..RSP. AP.RPV.A-- -.L.LV.I.Q .-...R.E.. .R..AQ..EK ..RD.LKE.

Canis_lupus ---------- ---------- ------...P A---CPGPRP .PPSR.RSP. APTRPV.T-- -.L.LV...Q .-.....E.. .R..AQ..EK ..KD.LKE.

Enhydra_lutris ---------- ---------- ------...P AA--ARGARP PPPSR.RSP. AP.RPV.A-- -.L.LV.I.Q .-.....E.. .R..AQ..EK ..RD.LEE.

Eumetopias_jubatus ---------- ---------- ------...P A---A.GARP PPRS..RSP. AP.RPV.A-- -.L.LV.I.Q .-...R.E.. .R..AQ..EK ..RD.LKE.

Felis_catus ---------- ---------- ------.G.P V---ARGARP .PASG.RSP- ----PV.G-- -.L.LVFI.Q .-.....ED. .R..AQ..EK ..RD.LKE.

Lynx_canadensis ---------- ---------- ------.G.P V---ARGARP .PASG.HSP- ----PV.G-- -.L.LVF..Q .-.....ED. .R..AQ..EK ..RD.LKE.

Mirounga_leonina ---------- ---------- ------...T A---ARGARP PPPS..RSP. AP.RPV.A-- L.L.LV.I.Q .-...R.E.. .R..AQ..EK ..RD.LKE.

Mustela_erminea ---------- ---------- ------...P AT--ARGARP PPPSR.RSP. AP.RPM.A-- -.LVLV.I.Q .-.....E.. .R..AQ..EK ..RD.LKE.

Mustela_putorius ---------- ---------- ------...P AT--ARGARP PPPSR.RSP. AP.RPM.A-- -.LVLV.I.Q .-.....E.. .R..AQ..EK ..RD.LKE.

Neomonachus_schauinslandi ---------- ---------- ------...A A---A.GARP PPPS..RSP. AP.RPV.A-- -.L.LV.I.Q .-...R.E.. .R..AQ..EK ..RD.LKE.

Odobenus_rosmarus ---------- ---------- ------...P A---A.GARP PPRS..RSP. AP.RPV.A-- -.L.LV.I.Q .-...R.E.. .R..AQ..EK ..RD.LKE.

Panthera_pardus ---------- ---------- ------.G.P V---ARGARP .PASG.RSP- ----PV.G-- -.L.LVFI.Q .-.....ED. .R..AQ..EK ..RD.LKE.

Phoca_vitulina ---------- ---------- ------...A A---A.GARP PPPS..RSP. AP.RPV.A-- -.L.LV.I.Q .-...R.E.. .R..AQ..EK ..RD.LKE.

Suricata_suricatta ---------- ---------- ------.G.P V---ARGARP .SASR.RAP- ----PVFA-- -.L.LVCI.Q .-.....E.. .R..AQ..EK ..RD.LKE.

Ursus_thibetanus -VR.APGGAA P..RKA..P. RVH.LP...P A---ARGARP PSPCR.RSPL AP.RPM.A-L L.L.LVPI.Q .-.....E.. .R..AQ..EK ..RD.LKE.

Vulpes_vulpes ---------- ---------- ------...P A---HPGPRP .PPSR.RSL. AP.RPV.T-- -.L.LV...Q .-.....E.. .R..AQ..EK ..KD.LKE.

Zalophus_californianus ---------- ---------- ------...P A---A.GARP PPRS..RSP. AP.RPV.A-- -.L.LV.I.Q .-...R.E.. .R..AQ..EK ..RD.LKE.

Eptesicus_fuscus -.R.EP.RTG P..R.A..W. GVR.LPTHIP A---SPGASS .P.RG.RSLQ SP.RP..A-- -.L.LV...Q .-.....E.. .R..AQ..EK ..RD.LKE.

Miniopterus_natalensis -.RLLEKRNS P.ELGV..-- ------AR.P A---SPGAGP .P.RG.GSLQ PP.RP..A-- -.L.LV...Q .-.....E.. .R..AQ..EK ..RD.LKE.

Myotis_lucifugus -.R.EP.RIG PR.R.A..RR GVR.LPTHSP A---SPGASS .P.RG.RSLQ SP.RP..A-- -.L.LV...Q .-A....E.. .R..AQ.REK ..RD.LKQ.

Myotis_myotis -.R.EP.RIG PR.R.S..R. GVR.LPTHSP A---SPGASS .P.RG.RSLQ SP.RS..A-- -.L.LV...Q .-.....E.. .R..AQ.REK ..RD.LKQ.

Pipistrellus_kuhlii -.RLEP.RRG P..R.A..R. GVHVSPTHVP A---.PGASS .P.LG.RSLQ SP.RP..A-- -.L.LV...Q .-.....E.. .R..AQ.REK ..RD.LKV.

Pteropus_alecto -.RLEP.RAG P..RNV.L.. GVQ.LPTLIP A---AW.ASP SP.RS.CSL. SP.RP..A-- -.L.LV.I.Q .-.....E.. .R..AQ..EK ..RD.LKE.

Rhinolophus_ferrumequinum -.RREP.RAG P..RSV..W. GVRVLPTRIP A---ARGASP .L.RR.CSLQ SP.RPL.A-- -.L.LV.I.Q .-.....E.. .R..AQ..EK ..RD.LKE.

Sturnira_hondurensis ---------- ---------- ---------- ---------- -----MCSLQ SP..P..A-- -.L.LV.I.Q I-S....Q.. .R..AQ..EK ..RD.LKE.

Condylura_cristat --------MG P..RKV.S.. GVHVLP.GIP A--------P EPF.R..SLC APRRS..A-- -.L.LV.I.Q .-.....EK. ....IQ..EQ ..RD.LKK.

Sorex_araneus -.RLEL..SG PV.RKV...S GIY.LS.GSR DARRAWRARP ICF.PS.TVC TPVRSVFT-- -.L.LV...H .-.....EK. ..R.AQ..EK ..RD.HEE.

Talpa_occidentalis ---------- ---------- ------.GVP A---PRGARP DPF.R..SLC .PVRP..T-- -.L.LV.I.Q .-.....EK. ....IQ..EQ ..RD.LKE.

Ceratotherium_simum -.R.EP.RAG P..RRV.... GVHVLPLGIA P---A.GATP PP..R.CSLL AA.RP..A-- -.L.LA.I.Q .-.....Q.. .R..AQ..EK ..RD.LKK.

Equus_caballus -.R.DP.RAG P..QKV.... GIHVPPLGIP P---ARGAGP PA.RR.RSL. AP.RPV.T-- -.L.LV.I.Q .-.....Q.. ....AQ..EK ..RD.LKK.

Manis_javanica -.R.ES.RTA PR.RKG.... GAHMLSTA.P A---ALG.WP .PR.S.HPLW TPVRP.HA-- -.L.LV.I.Q .-.....E.. ....AQ..EK ..KD.RKEA

Manis_pentadactyla -.R.KS.RTA PR.RKGR... GAHMLPTG.P A---ARGAWP .LR.S.HSLW TP.RP.HA-- -.L.LV...Q .-.A...E.. ....AQ..EK ..KD.LKEA

Prolemur_simus -VK.EPDRAR P..-R..... GVAELP.GIP A---PRGDSP VP..R.RSLW TPGKP..T-- -.V.LV.I.Q .-.....E.. .R..A...QE ..RD.LKK.

Tupaia_chinensis -.QR.P..AG P.GGKGR... GVPVQPVGI. A---SRG.SP .P..G.RSLW .PGRP..A-- -.V.LV.I.Q .-.....Q.. .R..NQ..EE ..RN.LKK.

Aotus_nancymaae -.KL.S.RAG P..GSVRL.. GIPELP.GIR A---PWR.SP FPLYT.C-IW APGRPI.A-- -.V.LV.I.Q .-.....E.. .R..AQ..QA ..KD.LKE.

Callithrix_jacchus -.KL.S.RAG P..GSM.L.. GIPELP.GIR A---PWG.SP FPLYT.C-IW ASGRPIVA-- -.V.LV.I.Q .-.....E.. .R..AQ..QA ..RD.LKE.

Carlito_syrichta -.KSAP.RAR P..RRA.L.. GVPPLP.GIP A---PWG.SP .PF.R.CSLW APGRP..T-- -.V.LV.I.Q .-.....E.. .R..AQ..EE ..RG.LKV.

Cebus_capucinus -.KL.S.RAG P..GSV.L.. GIPELP.GIR A---PWG.SP FPLYT.C-IW APGRPI.A-- -.V.LV.I.Q .-.....E.. .R..AQ..QA ..RD.LKE.

Cercocebus_atys -.KL.S.RAG P..GSA.L.. GVPELP.GIP A---PWG.SP .SL.R.CSLW APGRP..T-- -.V.LV.I.Q .-.....E.. .R..AQ..QA ..RD.LKE.

Chlorocebus_sabaeus -.KL.S.RAG P..GSA.L.. GVPELP.GIP A---SWG.SP .SF.R.CSLW APGRP..T-- -.V.LV.I.Q .-.....E.. .R..AQ..QA ..RD.LKE.

Colobus_angolensis -.TL.S.RAG P..GSA.L.. GVPELP.GIP A---PWG.SP .SF.R.CSLW APGRP..T-- -.V.LV.I.Q A-.....E.. .R..AQ..QA ..RD.LKE.

Gorilla_gorilla -.KL.S.RAG P..GSA.L.. GIPELP.GIP A---PWG.SP .SF.R.CSLW APGRP..T-- -.V.LV.I.Q .-.....E.. .R..AQ..QA ..RD.LKE.

Homo_sapiens -.KL.S.RAG P..GSA.L.. GVHELP.GIP A---PWG.SP .SF.R.CSLW APGRP..T-- -.V.LV.I.Q .-.....E.. .R..AQ..QA ..RD.LKE.

Hylobates_moloch -.KL.S.RAG P..GSV.L.. GVPELP.GIP A---PGG.TP .SF.R.CSLW APGRP..T-- -.V.LV.I.Q .-.....E.. .R..TQ..QA ..RD.LQE.

Macaca_fascicularis -.KL.S.RAG P..GSA.L.. GVPELP.GIP A---PWG.SP .SF.R.CSLW APGRP..T-- -.V.LV.I.Q .-.....E.. .R..AQ..QA ..RD.LKE.

Macaca_mulatta -.KL.S.RAG P..GSA.L.. GVPELP.GIP A---PWG.SP .SF.R.CSLW APGRL..T-- -.V.LV.I.Q .-.....E.. .R..AQ..QA ..RD.LKE.

Macaca_nemestrina -.KL.S.RAG P..GSA.L.. GVPELP.GIP A---PWG.SP .SF.R.CSLW APGRP..T-- -.V.LV.I.Q .-.....E.. .R..AQ..QA ..RD.LKE.

Mandrillus_leucophaeus -.KL.S.RAG P..GSA.L.. GVPELP.GIP A---PWG.SP .SL.R.CSLW APGRP..T-- -.V.LV.I.Q .-.....E.. .R..AQ..QA ..RD.LKE.

Microcebus_murinus -.K.EPDGAR PA.-RARA.. GVPQLP.G.P A---PRGDSP GP..R.HSLW TPGRP..T-- -.V.LV.I.Q .-.....E.. .R..A...QQ ..RD.LEK.

Nomascus_leucogenys -.KL.S.RAG P.KGSV.L.. GFPELP.GIP A---PRG.TP .SF.R.CSLW APGRP..T-- -.V.LI.I.Q .-.....E.. .R..TQ..QA ..RD.LKE.

Otolemur_garnettii MTK.EP.R-- --.-RV.... GVPELP..IP A---PGGASP PP.PR.PLLR TPGRP..I-- -.V.LG.I.Q .-S....E.. .R..A...QD ..RD.LKK.

Pan_paniscus -.KL.S.RAG P..GSA.L.. GVPELP.GIP A---PWG.SP .SF.R.CSLW APGRP..T-- -.V.LV.I.Q .-.....E.. .R..AQ..QA ..RD.LKE.

Pan_troglodytes -.KL.S.RAG P..GSA.L.. GVPELP.GIP A---PWG.SP .SF.R.CSLW APGRP..T-- -.V.LV.I.Q .-.....E.. .R..AQ..QA ..RD.LKE.

Piliocolobus_tephrosceles -.TL.S.RAG P..GSA.LQ. GVPQLP.GIP A---PWG.SP .SF.R.CSLW APGRP..T-- -.V.LV.I.Q .-.....E.. .R..AQ..QA ..RD.LKE.

Pongo_abelii -.KL.S.RAG P..GSV.L.. GVPELP.GIP A---RWG.SP .SF.R.CSLW APGRP..T-- -.V.LV.I.Q .-.....E.. .R..AQ..QA ..RD.LKE.

Propithecus_coquereli -.K.EPDRAR P..-RV.... GVPEVP.GIP A---PWRDSP VP..R.RSLW TPGRP..T-- -.V.LV.INQ .-.....E.. .R..A...QE ..RD.LKK.

Rhinopithecus_bieti -.TLES.RAG P..GSA.L.. GVPEL..GIP A---PWG.SP FSF.R.YSLW APGRP..T-- -.V.LV.I.Q A-.....E.. .R..AQ..QA ..RD.LKE.

Rhinopithecus_roxellana -.TLES.RAG P..GSA.L.. GVPEL..GIP A---PWG.SP FSF.R.CSLW APGRP..T-- -.V.LV.I.Q A-.....E.. .R..AQ..QA ..RD.LKE.

Saimiri_boliviensis -.KL.S.RAG P..GSV.L.. GIPELP.GIR A---PWG.SP FPLYT.C-IW ARGRPI.A-- -.V.LV.I.Q .-.....E.. .R..AQ..QA ..RD.LKE.

Sapajus_apella -.KL.S.RAG P..GSV.L.. GIPELP.GIR A---PWG.SP FPLYT.C-IW APGRPI.A-- -.V.LV.I.Q .-.....E.. .R..AQ..QA ..RD.LKE.

Theropithecus_gelada -.KL.S.RAG P..GNA.L.. GVPQLP.GIP A---PWG.SP .SL.R.CSLW APGRP..T-- -.V.LV.I.Q .-.....E.. .R..AQ..QA ..RD.LKE.

Arvicanthis_niloticus ---------- ---------- ---------- ---------- -----MRLLW GPGRP..T-- -.L.LVFI.Q .-.....E.. .R..AQ..EM ..RD.HHE.

Arvicola_amphibius ---------- ---------- ---------- ---------- -----MRLLW .PRRSL.T-- -.L.LT.I.Q .-.....ED. .R..AQ..EK ..RD.RKQ.

Castor_canadensis ---------- ---------- ------.GGR L---AWG.GP HP....RSLW .PGSP.FA-- -.V.LG.I.Q .-.....E.. .R..AQ..EK ..RD.LKE.

Cavia_porcellus -.R.HLHQPR P.PSNK..W. RAWVLC...P A---SWG.RP .CP.RTRLLW APGRS..A-- -.V.LV.I.Q .-.....E.. .R..AD..GK ..QD.LKE.

Cricetulus_griseus ---------- ---------- ---------- ---------- -----MRLLW GPRKP..T-- -.L.LV.I.Q .-.....ED. .R..AQ.REK ..RD.L.E.

Fukomys_damarensis -.RSHLCQPE PRAGSKR.W. RVW.LC..IP A---SRGAGP .C..RTRLLW GPGRP.FA-- -.V.LA.I.Q .-.....E.. .R..AQ..EK ..RD.LKE.

Grammomys_surdaster ---------- ---------- ---------- ---------- -----MCLLW GPGRP.IA-- -.L.LV.I.Q .-.....E.. .R..AQ..EM ..RD.RHE.

Heterocephalus_glaber -.R.HL.QPG PRASSK..W. RVWVLS..LP .---ARGAAP .C..RTRLLW APGRP.FT-- -.V.LA.I.Q .-.....E.. .R..AQ..EK ..RD.LKE.

Ictidomys_tridecemlineatus -.R.QL.RAG P..GSP.S.. G--VLPRNI. A---AGG.GS .L..G.QLLW APGRP..T-- -.V.LVFI.Q A-.....E.. .R..AQ..EK ..KD.LKK.

Jaculus_jaculus -.RLPS.PA. PR.SS....L GVQTR.VG.P E---ALG.TP .P..RMRLLW APGRP..A-- -.V.VA.I.Q .-........ ....AQ..EK ..RD.L.K.

Marmota_flaviventris -.R.QL.RAG P..GSP.S.. GVRVLP.SI. A---AGG.GS .L..G.HLLW APGRP..T-- -.V.LVFI.Q A-.....E.. .R..AQ..EK ..KD.LKK.

Marmota_marmota -.R.QL.RAG P..GSP.S.. GVRVLP.SI. A---VGG.GS .L..G.HLLW APGRP..T-- -.V.LVFI.Q A-.....E.. .R..AQ..EK ..KD.LKK.

Mastomys_coucha ---------- ---------- ---------- ---------- -----MRLLR GPGRP..I-- -.L.LV.INQ .-.....E.. VR..AR..EM ..T..RSE.

Meriones_unguiculatus ---------- ---------- ---------- ---------- -----MRL.W GPSRP..A-- -.L.LV.I.Q .-.....E.. .R..AQ.... ..QD.H.E.

Mesocricetus_auratus -.R.QS.PA. PN.GR..R.. RVR.QTVGIP A---A.R.DL .....MHLLW GPRRP..T-- -.L.LV.I.Q .-.....E.. .R..AQ.REK ..RD.L.E.

Microtus_ochrogaster ---------- ---------- ---------- ---------- -----MRLLW GPRRS..T-- -.L.LA.I.Q .-.....E.. .R..AQ..EK ..RD.RKQ.

Mus_caroli ---------- ---------- ---------- ---------- -----MRRLW GPGTP..A-- -.L.LV.I.Q .T..T..E.. V...AQ..E. ..KN.LKK.

Mus_musculus ---------- ---------- ---------- ---------- -----MRRLW GPGTP..A-- -.L.LV.I.Q .T.....E.. V...AQ..E. ..KD.LEK.

Mus_pahari ---------- ---------- ---------- ---------- -----MRRLW GPGTP..A-- -.L.LV.I.Q .-.....E.. V...AK..E. ..KD.HDNT

Mus_spicilegus ---------- ---------- ---------- ---------- -----MHRLW SPGTP..T-- -.L.LV.I.Q .T.....E.. V...AQ..E. ..KD.LEK.

Nannospalax_galili -.K.QLGRA. P..SR..... VVQ.RP.GIP E---T.G.SP .P..RIHQLW TPGRP..A-- -.V.LA.I.Q .-.....E.. .R..AQ..EK ..RD.L.E.

Octodon_degus -.R.HLHQLG PEASSK..W. RVWVLC..IP A---S.GPGP .CPP.TRLLW APGRP..A-- -.V.LV.I.Q .-.....E.. .R..AQ..DK ..RD.LKE.

Onychomys_torridus -.R.QP.PA. PR..R...V. RMR.QPVGIP A---A.R.GP .QF.GMHLLW GPRRL..T-- -.L.LV.I.Q .-.....E.. .R..TQ.REK ..KD.LKE.

Peromyscus_maniculatus ---------- ---------- -MR.QPVG.P A---A.R.GP .Q..GMRLLW GPRRL..T-- -.L.LV.I.Q .-.....E.. .R..TQ.REK ..RD.L.K.

Rattus_norvegicus -.R.QP.PA. PS.CR.A.V. RVR.QPVGIP E---A.GPVP ...QQMRLLW GPGRP..A-- -.L.LV.I.Q .-........ ....AN..EK ..ED.H..L

Rattus_rattus -.R.QP.PA. PS.CR.A... RVR.QPVGIP E---A.GPVP .R.QRMRLLW GPGRP..A-- -.L.LV.I.Q .-........ ....AN..EK ..ED.H..L

Sciurus_vulgaris -.RTQL.LAG P..SS----- ---QVP.SIP A---AGD.GS .L..G.HLLW .PGRP..T-- -.V.LVFI.Q A-.....E.. .R..AQ..EK ..RD.LKE.

Urocitellus_parryii -.R.QL.RAG P..GSP.S.. G--VLPRSI. A---AGG.GS ?L..G.HLLW APGRP..T-- -.V.LVFI.Q A-.....E.. .R..AQ..EK ..KD.LKK.

>>>

P P PP P P P PP

Monodelphis_domestica -S--GIYCNG TFDNYACWPH SFPGIVSIPC PSYLPWLKK- -----ESPGR VYRNCLAGGT WQTQENSTDI WWYDIECSEN RSFIQNDE-- HHTLLSTLQ

Phascolarctos_cinereus -.--.V.... ...R...... .P..N..V.. ......W.E- -----..... ...H...E.. ...L...... .R..T..... Y..K....-- ..I......

Sarcophilus_harrisii -.--...... ...R...... .P..N..... ......WQE- -----..... I..H...... ...L...... .Q.VT..... F..K....-- RY.......

Trichosurus_vulpecula -.--.V.... ...R...... .P..N..... ......WN.- -----..... ...R...... ...L...... .R..T....K Y.LKE.A.-- .........

Vombatus_ursinus -.--.V.... ...R...... .P..N..... ......W.E- -----..... ...H...E.. ...L...... .R..T..... Y..K....-- .........

Choloepus_didactylus -.--..F... ...K.V.... .S..N..V.. ......WRE- -----..SE. AH.R...Q.. ..MR....G. .QD.S..... H..K..VIH- .QAW.....

Dasypus_novemcinctus -.--..F... .....F.... .S..N..V.. ..F..SSH.- -----..S.. A..R..PQ.. ...K....R. .EDVS..AK. D.SGE.MR-- TLA..LI..

Chrysochloris_asiatica -.--..F... ...K.V.... .S..N..VS. .T....WR.- -----.NSE. A..R...Q.. ...W..T.N. .QD.S..D.. H..RK.V.-- .Q.......

Elephantulus_edwardii -P--.LF.H. ...R...... .S..N..V.. ......WRE- -----..S.. .H.R...Q.I ........NV .QDES..A.D S..KNTV.-- .RA...S..

Loxodonta_africana -.--.VF... ...K.V.... .S..N..V.. ......WSE- -----..S.. A..R...R.. ...R..T.V. .QD.S..A.. H..RK.V.-- .RA......

Orycteropus_afer -.--D.F... ...K.V.... .S..N..V.. ......WRE- -----..S.. A..R...Q.. ..MW..T..V .QDES..A.. H..RK.V.-- .QD......

Trichechus_manatus -.--..F... ...K.V.... .S..N..V.. ......WRE- -----..S.. A..R...Q.. ...R..T.V. .QD.S..A.. H..RK.V.-- .QA......

Balaenoptera_acutorostrata -.--.TV... ...Q.V.... .TA.N..V.. ......WR.- -----DNS.. A..Y..SQ.I ...L...... .QDNS..A.D H..K.KV.-- .RA......

Balaenoptera_musculus -.--.TV... ...Q.V.... .TA.N..V.. ......WR.- -----.NS.. A..Y..SQ.. ...L...... .QDNS..A.D H..K.KV.-- .RA......

Delphinapterus_leucas -.--.TV... ...Q.V.... .TA.N..V.. ......WR.- -----.HS.. A..Y..SQ.. ...L...... .QDNS..A.D H..K.KV.-- .RA......

Globicephala_melas -.--.TV... ...Q.V.... .TA.N..V.. ......WR.- -----.HS.. A..Y..SQ.. ...L...... .QDNS..A.D H..K.KV.-- .RA......

Lagenorhynchus_obliquidens -.--.AV... ...Q.V.... .TA.N..V.. ......WR.- -----.HS.. A..Y..SQ.. ...L...... .QDNS..A.D H..K.KV.-- .RA......

Lipotes_vexillifer -.--.TV... ...Q.V.... .TA.N..V.. ......WR.- -----.HS.. A..Y..SQ.. ...L...... .QDNS..A.D H..K.KV.-- YRA......

Monodon_monoceros -.--.TV... ...Q.V.... .TA.N..V.. ......WR.- -----.HS.. A..Y..SQ.. ...L...... .QDNS..A.D H..K.KV.-- .RA......

Neophocaena_asiaeorientalis -.--.TV... ...Q.V.... .TA.N..V.. ......WR.- -----.HS.. A..Y..SQ.. ...L...... .QDNS..A.D H..K.KV.-- .RA......

Orcinus_orca -.--.TV... ...Q.V.... .TA.N..V.. ......WR.- -----.HS.. A..Y..SQ.. ...L...... .QDNS..A.D H..K.KV.-- .RA......

Phocoena_sinus -.--.TV... ...Q.V.... .TA.N..V.. ......WR.- -----.HS.. A..Y..SQ.. ...L...... .QDNS..A.D H..K.KV.-- .RA......

Tursiops_truncatus -.--.TV... ...Q.V.... .TA.N..V.. ......WR.- -----.HS.. A..Y..SQ.. ...L...... .QDNS..A.D H..K.KV.-- .RA......

Bison_bison -.--.AV... ...K.V.... .T..N..V.. ......WRE- -----.NS.. A..Y..SR.A ...L...... .QDNS..A.D D..K.KV.-- .R.......

Bos_mutus -.--.AV... ...K.V.... .T..N..V.. ......WRE- -----.NS.. A..Y..SR.A ...L...... .QDNS..A.D D..K.KV.-- .R.......

Bos_taurus -.--.AV... ...K.V.... .T..N..V.. ......WRE- -----.NS.. A..Y..SR.A ...L...... .QDNS..A.D D..K.KV.-- .R.......

Bubalus_bubalis -.--.AV... ...K.V.... .T..N..V.. ......WRE- -----.NS.. A..Y..SR.A ...L...... .QDNS..A.D D..K.KV.-- .RS......

Camelus_dromedarius -.--.TV... ...Q.V.... .S..N..V.. ......WRE- -----.NS.. A..Y..SR.. .......... .QD.S..... H..K..V.-- ..A...S..

Camelus_ferus -.--.TV... ...Q.V.... ....N..V.. ......WRE- -----.NS.. A..Y..SR.. .......... .QD.S..... H..K..V.-- ..A...S..

Capra_hircus -.--.TV... ...Q.V.... .T..N..V.. ......WRE- -----.NS.. A..Y..SR.A ...L...... .QDNS..A.D H..K.KVG-- R........

Catagonus_wagneri -.--DTV... ...Q.I.... .SA.N..V.. ......WRE- -----..S.. A..H..PR.S .R.LR...SV .QD.SQ...D HG.QK.A.-- .........

Cervus_hanglu -.--.AV... ...Q.V.... .T..N..V.. ......WRE- -----.NS.. A..Y..SR.A ...L....AV .QDNS..A.D H..K.KV.-- .R....I..

Moschus_moschiferus -.--.AV... ...Q.V.... .T..N..V.. ......WRE- -----.NS.. A..Y..SR.A ...L...... .QDNS..A.D H..K.KV.-- .RA......

Sus_scrofa -.--DTV... ...R.I.... .SA.N..V.. ......WRE- -----.NS.. A..Y..PR.S ...LQ....V .QD.SQ...D H..K..V.H- ..A......

Vicugna_pacos -.--.TV... ...Q.V.... .S..N..V.. ......WRE- -----.NS.. A..Y..SR.. .......... .QD.S..... H..K..V.-- ..A...S..

Ailuropoda_melanoleuca -.--.TF... ...R.V.... .S..N..V.. ......WRE- -----.NS.. A..Y...R.. .R.L...... .QD.S...K. H.SGH.A.-- .RA......

Callorhinus_ursinus -.--.TF... ...R.V.... .S..N..V.. ......WRE- -----.NS.. A..Y...P.. ...L....N. .QD.S..... H.SQY.V.-- ..A.....K

Canis_lupus -.--.TF... ...R.V.... .S..N..V.. ......WSE- -----.NS.. A..Y...R.. ..ML...... .QD.S...KD H.SE..V.-- ..A......

Enhydra_lutris -.--.TF... ...R.V.... .S..N..V.. ......WRE- -----.NS.. A..Y...P.. ...L...... .QD.S...K. H.SDS.A.-- ..A......

Eumetopias_jubatus -.--.TF... ...R.V.... .S..N..V.. ......WRE- -----.NS.. A..Y...P.. ...L....N. .QD.S..... H.SQY.V.-- ..A.....K

Felis_catus -P--.TF... ...R.V.... .S..N..V.. ......WHE- -----.NS.. A..R...Q.. ...L....N. .QD.S...KK H.SE.KV.-- N.A......

Lynx_canadensis -P--.TF... ...R.V.... .S..N..V.. ......WHE- -----.NS.. A..R...Q.. ...L....N. .QD.S...KK H.SE.KV.-- N.A......

Mirounga_leonina -.--.TF... ...R.V.... .S..N..V.. ......WRE- -----.NS.. A..Y...P.. ...L....N. .QD.S..... H.SQY.V.-- ..A.....K

Mustela_erminea -.--.TF... ...R.V.... .S..N..V.. ......WRE- -----.NS.. A..Y...S.. ...L...... .QD.S...K. H.SNS.A.-- ..A......

Mustela_putorius -.--.TF... ...R.V.... .S..N..V.. ......WRE- -----.NS.. A..Y...S.. ...L...... .QD.S...K. H.SNS.A.-- ..A......

Neomonachus_schauinslandi -.--.TF... ...R.V.... .S..N..V.. ......WRE- -----.NS.. A..Y...P.. ...L....N. .QD.S..... H.SQY.V.-- ..A.....K

Odobenus_rosmarus -.--.TF... ...R.V.... .S..N..V.. ......WRE- -----.NS.. A..Y...P.. ...L....N. .QD.S..... H.SQY.V.-- ..A.....K

Panthera_pardus -P--.TF... ...R.V.... .S..N..V.. ......WHE- -----.NS.. A..R...Q.. ...L....N. .QD.S...KK H.SE.KV.-- N.A......

Phoca_vitulina -.--.TF... ...R.V.... .S..N..V.. ......WRE- -----.NS.. A..Y...P.. ...L....N. .QD.S..... H.SQY.V.-- ..A.....K

Suricata_suricatta -P--.TF... ...R.V.... .S..N..V.. ......WRE- -----.NS.. A..R...Q.. .E.L...... .QD.S...QQ H.SE..V.-- ..S......

Ursus_thibetanus -.--.TF... ...R.V.... .S..N..V.. ......WRE- -----.NS.. A..Y...R.. .R.L...... .QD.S...K. H.SRH.A.-- .RA......

Vulpes_vulpes -.--.TF... ...R.V.... .S..N..V.. ......WSE- -----.NS.K A..Y...R.. ..ML...... .QD.S...KD H.SE..V.-- ..A......

Zalophus_californianus -.--.TF... ...R.V.... .S..N..V.. ......WRE- -----.NS.. A..Y...P.. ...L....N. .QD.S..... H.SQY.V.-- ..A.....K

Eptesicus_fuscus -.--..F... ...R.V.... .S..N..V.. ......WSE- -----.N... A..R...R.. ...L..R.-V .QD.S..... H..QA.V.-- ..A......

Miniopterus_natalensis -.--.TF... ...Q.V.... .PA.N..V.. ......WRE- -----.NS.. A..R...R.. ...L...... .QD.S..... H..KE.V.-- ..A......

Myotis_lucifugus -.--.VF... ...R.V.... .S..N..V.. .....GWSE- -----.N... A..R...R.. ...LA.R.-V .QD.S..... HNVQK.V.-- ..A......

Myotis_myotis -.--.VF... ...R.V.... .S..N..V.. .....GWSE- -----.N... A..R...R.A ...LA.R.-V .QD.S..... H.IQK.V.-- ..A......

Pipistrellus_kuhlii -.--..F... ...R.V.... .S..N..V.. ......WSE- -----.N... A..R...R.. ...LV.R.-V .QD.S..... H..QKKV.-- ..A......

Pteropus_alecto -.--..F... ...Q.V.... .S..NI.V.. ......WRE- -----.NS.. A..H...Q.. ...LK..... .QD.S..... H..KE.V.-- S.A......

Rhinolophus_ferrumequinum -.--..F... ...Q.V.... .S..N..V.. ......WRE- -----.NT.. A..H...Q.. ..LLK..... .QD.S..A.. H..KE.V.-- ..A......

Sturnira_hondurensis -.--..F... ...R.V.... .S..N..V.. ......WRE- -----.DS.. A..H..EQ.. .R.L....G. .QD.S..A.. H..KE.VK-- .RG......

Condylura_cristat -.--..F... ...K...... .S..N..... .P....WRE- -----..... .H.H...Q.. ...L...S.. .QDIS..L.D Q..KED-.K- ..D.....K

Sorex_araneus -.--..F... ...Q.V.... .L..N..V.. .F....WNE- -----.RS.M AH.Y.S.Q.. ..MR...... .QD.S....E H..KKKEK-- ..D..LN..

Talpa_occidentalis -.--..F... ...K...... .S..N..V.. ......WRE- -----..S.. .H.H...Q.. ...L...... .QDNS..L.D QR.KE.VSK- .RD......

Ceratotherium_simum -.--.VF... ...Q.V.... .S..N..V.. ......WRE- -----.NS.. A..H...W.. .......... .QD.S..... H..K..V.-- ..A......

Equus_caballus -.--..F... ...R.V.... .L..N..V.. ......WR.- -----.NA.. A..H...R.A ...R.....T .QDVS..... H..K..E.-- ..A......

Manis_javanica -.--..F... ...Q.V.... .S..NI.V.. ......WRE- -----.NS.K A..H.S.Q.A ...L...... .QD.S..... H..K..V.-- ..A......

Manis_pentadactyla -.--..F... ...Q.V.... .S..N..V.. ......WRE- -----.NS.K A..H...Q.A ...L...... .QD.S..... H..K..V.-- ..A......

Prolemur_simus -.--.VF... ...Q.V.... .P..N..V.. ......WNE- -----..S.. AH.Y...R.. ...R..A... .QD.S..... H..K..VD-- .YA......

Tupaia_chinensis -.--..F... ...R.V.... .S..N..V.. .....GWS.- -----..S.. A..H...R.. ...L...... .QD.S...K. H..K..VD-- .YA......

Aotus_nancymaae -P--..F... ...QFV.... .P..N..V.. ......WSE- -----..S.. A..H...Q.. ...I..A... .QD.S..... H..K..VD-- .YA......

Callithrix_jacchus -P--..F... ...QFV.... .P..N..VS. ......WSE- -----..S.. A..H...Q.. ...I..A... .QD.S..... H..K..VD-- .YA......

Carlito_syrichta -.--.EF... ...R.V.... .S..N..V.. ......WS.- -----..S.. A..H...Q.. ...I..T... .QD.S...K. H..NK.VD-- .YP......

Cebus_capucinus -P--..F... ...QFL.... .P..N..V.. ......WSE- -----..S.. A..H...Q.. ...V..A... .QD.S..... H..K..VD-- .YA......

Cercocebus_atys -.--.VF... ...Q.V.... .S..N..V.. ......WSE- -----..S.. A..H...Q.. ...I..A... .QD.S..... H..K..VD-- .YA......

Chlorocebus_sabaeus -.--.VF... ...K.I.... .S..N..V.. ......WSE- -----..S.. A..H...R.. ...I..A... .QD.S..A.. H..K..VD-- .YA......

Colobus_angolensis -.--..F... ...Q.V.... .S..NI.V.. ......WSE- -----..S.. A..H...Q.. ...I..A... .QD.S..... H..K..VD-- .YA......

Gorilla_gorilla -.--..F... ...Q.V.... .S..N..V.. ......WSE- -----..S.. A..H...Q.. ...V..A... .QD.S..... H..K..VD-- RYA......

Homo_sapiens -.--..F... ...Q.V.... .S..N..V.. ......WSE- -----..S.. A..H...Q.. ...I..A... .QD.S..... H..K..VD-- RYA......

Hylobates_moloch -.--..F... ...Q.V.... .S..N..V.. ......WSE- -----..S.. A..H...Q.. ...I..A... .QD.S..... H..K..VD-- .YA......

Macaca_fascicularis -.--..F... ...Q.V.... .S..N..V.. ......WSE- -----..S.. A..H...R.. ...I..A... .QD.S..... H..K..VD-- .YA......

Macaca_mulatta -.--..F... ...Q.V.... .S..N..V.. ......WSE- -----..S.. A..H...R.. ...I..A... .QD.S..... H..K..VD-- .YA......

Macaca_nemestrina -.--..F... ...Q.V.... .S..NI.V.. ......WSE- -----..S.. A..R...R.. ...I..A... .QD.S..... H..K..VD-- .YA......

Mandrillus_leucophaeus -.--.VF... ...Q.V.... .S..N..V.. ......WSE- -----..S.. A..H...R.. ...I..A... .QD.S..... H..K..VD-- .YA......

Microcebus_murinus -.--.VF... ...Q.V.... .S..N..V.. ......WNE- -----..S.. AH.Y...R.. ...R..A... .QD.S..... H..K..VD-- .YA......

Nomascus_leucogenys -.--..F... ...Q.V.... .S..N..V.. ......WSE- -----..S.. A..H...Q.. ...I..A... .QD.S..... H..K..VD-- .YA......

Otolemur_garnettii -.--.VF... ...Q.V.... .S..N..V.. ......WNE- -----..S.. A..Y..VQ.. ...R..A... .QD.S..... H..K..VD-- .YS......

Pan_paniscus -.--..F... ...Q.V.... .S..N..V.. ......WSE- -----..S.. A..H...Q.. ...I..A... .QD.S..... H..K..VD-- RYA......

Pan_troglodytes -.--..F... ...Q.V.... .S..N..V.. ......WSE- -----..S.. A..H...Q.. ...I..A... .QD.S..... H..K..VD-- RYA......

Piliocolobus_tephrosceles -.--..F... ...Q.V.... .S..N..V.. ......WSE- -----..S.. A..H...Q.. ...I..A... .QD.S..... H..K..VD-- .YA......

Pongo_abelii -.--..F... ...Q.V.... .S..N..V.. ......WSE- -----..S.. A..H...Q.. ...I..A... .QD.S..... H..K..VD-- RYA......

Propithecus_coquereli -.--.VF... ...Q.V.... .S..N..V.. ......WNE- -----..S.. AR.Y...R.. ...R..A... .QD.S..... H..K..VD-- .YA......

Rhinopithecus_bieti -.--..F... ...Q.V.... .S..N..V.. ......WSE- -----..S.. A..H...Q.. ...I..A... .QD.S..... H..K..VD-- .YA......

Rhinopithecus_roxellana -.--..F... ...Q.V.... .S..N..V.. ......WSE- -----..S.. A..H...Q.. ...I..A... .QD.S..... H..K..VD-- .YA......

Saimiri_boliviensis -P--..F... ...QFV.... .P..N..V.. ......WTD- -----..S.. A..H...Q.. ...I..A... .QD.S..... H.VK..VD-- .YA......

Sapajus_apella -P--..F... ...QFL.... .P..N..V.. ......WSE- -----..S.. A..H...Q.. ...V..A... .QD.S..... H..K..VD-- .YA......

Theropithecus_gelada -.--.VF... ...Q.V.... .S..N..V.. ......WSE- -----..S.. A..H...Q.. ...I..A... .QD.S..... H..K..VD-- .YA......

Arvicanthis_niloticus -.--.VF... ...Q.V.... .S..N..V.. ......WNE- -----..... A..H...Q.. ..K...T... .QD.S...K. H.VK..MD-- .Y.......

Arvicola_amphibius -.--..F... ...QFV.... .S..N..V.. ..F...WSE- -----...K. A..H...Q.. ...R..A.T. .QD.S...G. Y..K.KV.-- .YA......

Castor_canadensis -.--..F... ...R.V.... .S..N..V.. ......WNE- -----..S.. A..H...R.. ...R....N. .QD.S..... H..KE.VD-- .YA......

Cavia_porcellus -.--.VF... ...R.V.... .P..N..V.. .....GWNE- -----....K A..H...R.. ...L..T... .QD.S....K H..KP.VD-- .YA..A...

Cricetulus_griseus -.--..F... ...Q.V.... .S..N..V.. ......WNE- -----..... A..H...Q.. ...RV.A.A. .QD.S..... H..K..VD-- .YS......

Fukomys_damarensis -.--..F... ...R.V.... .S..N..V.. ......WNEE LCLLS..S.. A..H...R.. ..IL..T... .QD.S..A.. H..K..VD-- .YA......

Grammomys_surdaster -.--.VF... ...Q.V.... .T..N..V.. ......WNE- -----..... A..H...Q.. ..K...T... .QD.S....D H.VK..ID-- .Y.......

Heterocephalus_glaber -.--..F... ...R.V.... .S..N..V.. ......WNE- -----..S.. A..H...R.. ...L..T... .QD.S..A.. H..K..VD-- .YA......

Ictidomys_tridecemlineatus -.--..F... ...Q.V.... .S..N..V.. ......WNE- -----..S.. A..H...R.. ...LV.T... .QD.S..... H..K..V.-- NYA......

Jaculus_jaculus -.--.VF... ...Q.I.... .S..N..V.. ......WSE- -----..S.. A..Y...R.. ...L..A... .QD.S..... H..K..VD-- .YA......

Marmota_flaviventris -.--..F... ...Q.V.... .S..N..V.. ......WNE- -----..S.. A..H...R.. ...LV.T... .QD.S..... H..K..V.-- NYA......

Marmota_marmota -.--..F... ...Q.V.... .S..N..V.. ......WNE- -----..S.. A..H...R.. ...LV.T... .QD.S..... H..K..V.-- NYA......

Mastomys_coucha -.--.VF... ...Q.V.... .S..N..V.. ......WNE- -----..... A..H...Q.. ...K..T... .QD.S...K. Q.SK..VD-- .YP..Y...

Meriones_unguiculatus -.--..F... ...R.V.... .S..N..V.. ......WNE- -----..... A..Y.S.Q.. ..K...A... .QD.S..... H.LK..VD-- .LA......

Mesocricetus_auratus -.--..F... ...Q.V.... .S..N..V.. ......WSE- -----..... A..H...Q.. ...RA.A.A. .QD.S..... H..K..VD-- .YG......

Microtus_ochrogaster -.--..F... ...QFV.... .S..N..V.. ..F...WSE- -----...R. A..H...Q.. ...R..A.T. .QD.S...G. Y..K.KV.-- .YA......

Mus_caroli -.--.VF... ...K.V.... ....N..V.. ......WN.- -----..... A..H...Q.. ..KR.....T .QDES..... H..K..VDHY .Y.......

Mus_musculus -.--.VF... ...K.V.... ....N..V.. ......WN.- -----..... A..H...Q.. ..K......T .QDES..... H..K..VDHY .........

Mus_pahari -.VQ.VF... ...R.V.... .S..N..V.. ......WN.- -----..... A..H...Q.. ..R...T... .QD.S..... H..K..VDRY .Y.......

Mus_spicilegus -.--.VF... ...K.V.... ....N..V.. ......WN.- -----..... A..H...Q.. ..K......T .QDES..... H..K..VDHY .........

Nannospalax_galili -.--..F... ...QFV.... .S..N..V.. ......WNE- -----.GS.. A..H...R.. ...L..A... .QD.S..... H..K..VD-- .YA......

Octodon_degus S.--..F... ...R.V.... .S..N..V.. ......WNE- -----..S.. A..H...R.. .K.R..T... .QD.S..... H..K..VD-- .YA......

Onychomys_torridus -.--.VF... ...KLV.... .S..N..V.. ......WSE- -----..... A..H...Q.. ...R..T.A. .QD.S..A.. H..K..VD-- .YA......

Peromyscus_maniculatus -.--..F... ...KLV.... .S..N..V.. ......WSE- -----..... A..H...Q.. ...R..A.A. .QD.S..A.. H..K..VD-- .YA......

Rattus_norvegicus -.--..F... ...R.V.... .Y..N..V.. ......WNA- -----..... A..H...Q.. ...R..T... .QDES..... H..R..VD-- .YA..Y...

Rattus_rattus -.--..F... ...RFV.... .Y..N..V.. ......WNA- -----..... A..H...Q.. ...R..T... .QDES..... N..KK.VD-- .Y...Y...

Sciurus_vulgaris -.--..F... ...Q.V.... .S..N..V.. ......WNE- -----..S.. A..H...R.. ...LV.A... .QD.S....D H..K..V.-- NYA...S..

Urocitellus_parryii -.--..F... ...Q.V.... .S..N..V.. ......WNE- -----..S.. A..H...R.. ...LV.T... .QD.S..... H..K..V.-- NYA......

1.50 12.50 2.50

TM1 **$** <<< **$** >>> **$** TM2 <<< >>>

P P G G P P P PP PP P PP

Monodelphis_domestica KVYTLGYSLS LISLFLALII LMCLRRVHCT RNYIHMNLFG SFMLRALAVL VKDIIIHNSY SKRPDDENGW ISYLSESL-- -------STC RTAQTLLHY

Phascolarctos_cinereus IM........ .......V.. .L........ .......... .......... ...V...... .......... M......S-- -------... ....V....

Sarcophilus_harrisii II........ .......... .L....I... .......... .......SI. M.....QD.. .......... M..M....-- -------... ....V.V..

Trichosurus_vulpecula IM........ .......... .L........ .......... ........I. .....V.... .......... M......S-- -------.M. ....V....

Vombatus_ursinus IM........ .......V.. .L........ .......... .......... .......... .E........ M......S-- -------... ....V....

Choloepus_didactylus LM..V..... .V..L...SL .L...KL... .......V.A ..I....... ...VVVS... .R........ ......VS-- -------VS. .S..V....

Dasypus_novemcinctus RI..V.H... .A..S..IGL .LF..KL... .....V.V.A A.I.K.VV.. I...NL.S.. ..K.NS.DE. .A.Q..VSGS -------TS. .L.HA....

Chrysochloris_asiatica LM..V..... .V......AL .LS..KL... .........A ..I..G.S.. ...VV.Y... T.K...L... M.FIPQVS-- -------TS. .S..V....

Elephantulus_edwardii LM..V..... F.......AL .L...KL... .........A ..I....... ...V.TY... AQK..SVSD. L..IPQVS-- -------.S. .S..I....

Loxodonta_africana LM..V..... .V......AL .LF..KL... .........A ..I....... ...VVVY... NQK...V... M..M..VS-- -------AS. .S..V....

Orycteropus_afer LM..V...F. .A..LM..AL .LF..KL... .........A ..I....... ...AVVY... TRK..SV... M..M..VS-- -------TS. .S..V....

Trichechus_manatus LM..V..... .V......AL .LF..KL... .........A ..I....... ...VVVY... N.K..NV... M..M..VS-- -------TS. .S..V....

Balaenoptera_acutorostrata LL..V..... IV......TL .LF..KL... .........A ..I....... ...V.FY... ....NS.KE. M..V..VS-- -------TS. .S..V....

Balaenoptera_musculus LL..V..... IV......TL .LF..KL... .........A ..I....... ...V.FY... .....S.KE. M..M..AS-- -------TS. .S..V....

Delphinapterus_leucas LL..V..... IV......TL .LF..KL... .........A ..I....... ...V.FY... .....S.KQ. M..V..VS-- -------TS. .S..V....

Globicephala_melas LL..V..... IV......TL .LF..KL... .........A ..I....... ...V.FY... .....S.KQ. M..V..MS-- -------TS. .S..V....

Lagenorhynchus_obliquidens LL..V..... IV......TL .LF..KL... .........A ..I....... ...V.FY... .....S.KQ. M..V..VS-- -------TS. .S..V....

Lipotes_vexillifer LL..V..... IV......TL .LF..KL... .........A ..I....... .R.S.VY... .....S.RE. M..V..VS-- -------TS. .S..V....

Monodon_monoceros LL..V..... IV......TL .LF..KL... .........A ..I....... ...V.FY... .....S.KQ. M..V..VS-- -------TS. .S..V....

Neophocaena_asiaeorientalis LL..V..... IV......TL .LF..KL... .........A ..I....... ...V.FY... .....S.KQ. M..V..VS-- -------TS. .S..V....

Orcinus_orca LL..V..... IV......TL .LF..KL... .........A ..I....... ...V.FY... .....S.KQ. M..V..VS-- -------TS. .S..V....

Phocoena_sinus LL..V..... IV......TL .LF..KL... .........A ..I....... ...V.FY... .....S.KQ. M..V..VS-- -------TS. .S..V....

Tursiops_truncatus LL..V..... IV......TL .LF..KL... .........A ..I....... ...V.FY... .....S.KQ. M..V..VS-- -------TS. .S..V....

Bison_bison LL..V..... IV......TL .LF..KL... .........A ..I....... ...V.FYK.. ..T.NS.TE. L..V..IA-- -------AS. .S..V....

Bos_mutus LL..V..... IV......TL .LF..KL... .........A ..I....... ...V.FYK.. ..T.NS.TE. L..V..IA-- -------AS. .S..V....

Bos_taurus LL..V..... IV......TL .LF..KL... .........A ..I....... ...V.FYK.. ..T.NS.TE. L..V..IA-- -------AS. .S..V....

Bubalus_bubalis LL..V..... IV......TL .LF..KL... .........A ..I....... ...V.FYK.. ..T.NS.TE. L..V..IA-- -------AS. .S..V....

Camelus_dromedarius LL..V..... IV......TL .LF..KL... .........A ..I....... ...VVFY... .....N.QE. M..V..MA-- -------AS. .S..V....

Camelus_ferus LL..V..... IV......TL .LF..KL... .........A ..I....... ...VVFY... .....N.QE. M..V..MA-- -------AS. .S..V....

Capra_hircus LL..V..... IV......TL .LF..KL... .........A ..I....... ...V.FYK.. ..K.NS.KE. L..M..IS-- -------AS. .S..V....

Catagonus_wagneri LL..V..... VV..L...TL .LF..KL... .........A ..I.K.V... ...V.FY... .R...S.KE. L..V..MS-- -------TS. .A..V....

Cervus_hanglu LL..V..... IV......TL .LF..KL... .........A ..I.K..... ...V.LYK.. ..K.NS.K.. L..M..IS-- -------AS. .S..I....

Moschus_moschiferus LL..V..... IV......TL .LF..KL... .........A ..I....... ...V.FYK.. ..K.TS.RE. L..V..IS-- -------AS. .S..V....

Sus_scrofa LL..V..... TA......TL .L...KL... .........A ..I.K.V... A..V.FDS.. .....S.KA. L..V..MS-- -------PS. ....V....

Vicugna_pacos LL..V..... IV......TL .LF..KL... .........A ..I....... ...V.FY... .....N.QE. M..V..MA-- -------AS. .S..V....

Ailuropoda_melanoleuca LL..V..... I...L...TL .LF..KL... .........A ..I....... ...A.F.... .R..KSDKE. M..V..IS-- -------AS. .S..V....

Callorhinus_ursinus VL..V..... I...L...TL .LF..KL... .........A ..I....... ...A.HS... .R..NNDK.. ...M..VS-- -------AS. .S..V....

Canis_lupus LL..V..... V...L...TL .LF..KL... .........A ..I....... ...A.FY... .R..NSDKE. ...V..IS-- -------AS. .S..V....

Enhydra_lutris LL..V..... IV..L...TL .LF..KL... .........A ..I....... ...A..Y... .R..NSDKE. M..V..IS-- -------AS. .S..V....

Eumetopias_jubatus VL..V..... I...L...TL .LF..KL... .........A ..I....... ...AVHS... .R..NNDK.. ...M..VS-- -------AS. .S..V....

Felis_catus LL..V..... V...L...T. .LF..KL... .........A ..I....... ...A.FY... .R..NSD.K. L.FV..IS-- -------VS. .S..F....

Lynx_canadensis LL..V..... VV..L...T. .LF..KL... .........A ..I....... ...A.FY... .R..NSD.K. L.FV..IS-- -------VS. .S..F....

Mirounga_leonina VL..V..... I...L...TL .LF..KL... .........A ..I....... ...A.HS... .R..NSDK.. ...M..VS-- -------AS. .S..V....

Mustela_erminea LL..V..... IV..L...TL .LF..KL... .........A ..I....... ...A.VY... .R..NSDKE. V..V..IS-- -------AS. .S..V....

Mustela_putorius LL..V..... IV..L...TL .LF..KL... .........A ..I....... ...A.VY... .R..NSDKE. V..V..IS-- -------AS. .S..V....

Neomonachus_schauinslandi VL..V..... I...L...TL .LF..KL... .........A ..I....... ...A.HS... .R..NNDK.. ...M..VS-- -------AS. .S..V....

Odobenus_rosmarus VL..V..... I...L...TL .LF..KL... .........A ..I....... ...A.HS... .R..NNDK.. ...M..VS-- -------AS. .S..V....

Panthera_pardus LL..V..... VV..L...T. .LF..KL... .........A ..I....... ...A.FY... .R..NSD.K. L.FV..IS-- -------VS. .S..F....

Phoca_vitulina VL..V..... I...L...TL .LF..KL... .........A ..I....... ...A.HS... .R..NSDK.. ...M..VS-- -------AS. .S..V....

Suricata_suricatta LL..V..... V...L...TL .LF..KL... .........A ..I....... ...A.FY... .R..TSDKK. M.FV..IS-- -------VS. .S..F....

Ursus_thibetanus LL..V..... I...L...TL .LF..KL... .........A ..I....... ...A.FY... .R..KSDKE. M..V..IS-- -------AS. .S..V....

Vulpes_vulpes LL..V..... V...L...TL .LF..KL... .........A ..I....... ...A.FY... .R..NSDKE. M..V..IS-- -------AS. .S..V....

Zalophus_californianus VL..V..... I...L...TL .LF..KL... .........A ..I....... ...AVHS... .R..NNDK.. ...M..VS-- -------AS. .S..V....

Eptesicus_fuscus LL..V..... TVA.L...TL .LF..KL... .........A ..V.K..... G...VF.... .R...T.KE. V..M..IS-- -------AS. .S..V....

Miniopterus_natalensis LL..V..... IV..L...TL .LF..KL... .........A ..V..G.... ...V.LY... .R...N.KE. V..V.QIS-- -------AS. .S..V....

Myotis_lucifugus LL..V..... AVA.L...TL .LF..KL... .........A ..V.K..... GR..VF.... .R...T.RQ. V..M..IS-- -------TS. .S..V....

Myotis_myotis LL..V..... TVA.L...TL .LF..KL... .........A ..V.K..... G...VF.... .R...T.RE. V..M..IS-- -------TS. .S..V....

Pipistrellus_kuhlii LL..V..... TVT.L...TL .LF..KL... .........A ..I.K..... G...VF.... .R...T.KE. V..M..IS-- -------A.. .S..V....

Pteropus_alecto LL..V..... IV..L...TL .L...KL... .........A ..I....V.. ...V.L.... ....NN.KE. M..V..IS-- -------AS. .S..V....

Rhinolophus_ferrumequinum ML..V..... IV..L...TL .LF..KL... .........A ..I...V... .....LY... ....NN.KE. ...V..MS-- -------AS. .S..V....

Sturnira_hondurensis LL..V..... IAA.L...T. .L...KL... .Y.......A ..I..GV... .....FY... ....NS.KE. M..V..IS-- -------TS. .S..V....

Condylura_cristat LL..V..... .A..C...TL .LF...L... .........A ..I....... ...VV..... ....SN.K.. ...MP.IF-- -------MP. .M..A...F

Sorex_araneus LL..V...V. IA..C...SL .LF..KL... .........A ..I....... ...VV..K.. ....NN.EE. M..M..VS-- -------VS. .SV.I....

Talpa_occidentalis LL..V..... .A..C...TL .LF..KL..K .........A ..I....... ...V...... .N..SS.K.. V..MT.IS-- -------TS. .VV.S...F

Ceratotherium_simum LL..V..... IV......TL .LF..KL... .........A ..I....... ...VVF.... .R..NN.EE. M..V..IS-- -------TS. .S..V....

Equus_caballus LL..V..... IV......TL .LF..KL... .........A ..I....... ...V.FY... .R..NN.KE. M..V..IS-- -------TS. .S..V....

Manis_javanica LL..V..... IV..L...TL .LF..KL... .........A ..I....... ...V.FY... ....NN.KE. M..V..IS-- -------TS. .S..V....

Manis_pentadactyla LL..V..... IV..L...TL .LF..KL... .........A ..I....... ...V.FY... ....NN.KE. M..V..IS-- -------TS. .S..V....

Prolemur_simus LM..V...F. .V..L...TL .LF..KL... .........A ..I...V... ...VVFY... .....N.D.. M.....IS-- -------TS. .SV.V....

Tupaia_chinensis LM..V..... ........TL .LL..KL... .........A ..I....... ...VVFY... .....N.... M.....MS-- -------TS. .SV.I....

Aotus_nancymaae LM..V...F. ........TL .LF..KL... .........A ..I..T.... ...VVFY... .....N.... M.....MS-- -------TS. .SV.V....

Callithrix_jacchus LM..V...F. ........TL .LF..KL... .........A ..I..T.... ...VVFY... .....N.... M.....MS-- -------TS. .SV.V....

Carlito_syrichta LM..V..... ........TL .LF..KL... .........A ..I.K..V.. ...VVS.... ....NN.S.. M..VP.M.-- -------AS. .SVHV....

Cebus_capucinus LM..V...F. ........TL .LF..KL... .........A ..I..T.... ...VVFY... .....N.... M.....MS-- -------TS. .LV.V....

Cercocebus_atys LM..V...V. ........TL .LF..KL... .........A ..I..T.... ...VVFY... .....N.... M.....MS-- -------TS. .SV.V....

Chlorocebus_sabaeus LM..V...V. ........TL .LF..KL... .........A ..I..T.... ...VVFY... .....N.... M.....MS-- -------TS. .SV.V....

Colobus_angolensis LM..V...V. ........TL .LF..KL... .........A ..I..T.... ...VVFY... .....N.... M.....MS-- -------TS. .SV.V....

Gorilla_gorilla LM..V...F. ........TL .LF..KL... .........A ..I..T.... ...VVFY... .....N.... M.....MS-- -------TS. .SV.V....

Homo_sapiens LM..V...F. ........TL .LF..KL... .........A ..I..T.... ...VVFY... .....N.... M.....MS-- -------TS. .SV.V....

Hylobates_moloch LM..V...F. ........TL .LF..KL... .........A ..I..T.... ...VVFY... .....N.... M.....MS-- -------TS. .SV.V....

Macaca_fascicularis LM..V...V. ..F.....TL .LF..KL... .........A ..I..T.... ...VVFY... .....N.... M.....MS-- -------TS. .SV.V....

Macaca_mulatta LM..V...V. ..F.....TL .LF..KL... .........A ..I..T.... ...VVFY... .....N.... M.....MS-- -------TS. .SV.V....

Macaca_nemestrina LM..V...V. ..F.....TL .LF..KL... .........A ..I..T.... ...VVFY... .....N.... M.....MS-- -------TS. .SV.V....

Mandrillus_leucophaeus LM..V...V. .........L .LF..KL... .........A ..I..T.... ...VVFY... .....N.... M.....MS-- -------TS. .SV.V....

Microcebus_murinus LM..V...F. .V......TL .LF..KL... .........A ..I...V... ...VVFY... .....S.A.. M.....IS-- -------TS. .SV.V....

Nomascus_leucogenys LM..V...F. ........TL .LF..KL... .........A ..I..T.... ...VVFY... .....N.... M.....MS-- -------TS. .SV.V....

Otolemur_garnettii LM..V...F. ........AL .L....L... .........A ..I...M... ....VFY... .....N.D.. M.....IS-- -------TS. .SV.V....

Pan_paniscus LM..V...F. ........TL .LF..KL... .........A ..I..T.... ...VVFY... .....N.... M.....MS-- -------TS. .SV.V....

Pan_troglodytes LM..V...F. ........TL .LF..KL... .........A ..I..T.... ...VVFY... .....N.... M.....MS-- -------TS. .SV.V....

Piliocolobus_tephrosceles LM..V...V. ........TL .LF..KL... .........A ..I..T.... ...VVFY... .....N.... M.....MS-- -------TS. .SV.V....

Pongo_abelii LM..V...F. ........TL .LF..KL... .........A ..I..T.... ...VVFY... .....N.... M.....MS-- -------TS. .SV.V....

Propithecus_coquereli LM..V...F. .V......TL .LF..KL... .........A ..I...V... ...VVFY... .....N.D.. M.....IS-- -------TS. .SV.V....

Rhinopithecus_bieti LM..V...V. ........TL .LF..KL... .........A ..I..T.... ...VVFY... .....N.... M.....MS-- -------TS. .SV.V....

Rhinopithecus_roxellana LM..V...V. ........TL .LF..KL... .........A ..I..T.... ...VVFY... .....N.... M.....MS-- -------TS. .SV.V....

Saimiri_boliviensis LM..V...F. ........TL .LF...L... .........A ..I..T.... ...VVFY... .....N.... M.....MS-- -------AS. .SV.V....

Sapajus_apella LM..V...F. ........TL .LF..KL... .........A ..I..T.... ...VVFY... .....N.... M.....MS-- -------TS. .SV.V....

Theropithecus_gelada LM..V...V. ..F.....TL .LF..KL... .........A ..I..T.... ...VVFY... .....N.... M.....MS-- -------TS. .SV.V....

Arvicanthis_niloticus LM..V..... ........AL .LF..KL... .........A ..I....... ...MVFY... .....N.S.. M..I..MS-- -------TS. .SV.V....

Arvicola_amphibius LM..V..... ....L...TL FL...KL... .........A ..I....... ....VFY... ....NS.S.. M.....DSPR FCPCRCLPS. .SV.V....

Castor_canadensis LM..V..... ........TL FLF..KL... .........A ..I....... ....VF.... .....S.K.. M..Q..MS-- -------PS. .LV.V....

Cavia_porcellus LM..V...V. .V......SL .LF..KL... .........A ..I...V... ...VVFSS.. .R.....S.. M.....VS-- -------TS. .SV.V....

Cricetulus_griseus L...V..... ........TL .LF..KL... .........A ..I....... .....FY... .....N.S.. M.....MS-- -------AS. .CV.V....

Fukomys_damarensis LM..V..... ........SL .WF..KL... .........A ..I.K.V... ...VVFS... .......S.. T.....VS-- -------TS. .SV.V....

Grammomys_surdaster LM..V..... ........AL .LF..KL... .........A ..I....... ...MVFY... .....N.S.. M..T..IS-- -------AS. .SV.V....

Heterocephalus_glaber LM..V..... ........SL .LF..KL... .........A ..I...V... ...VVFS... .....S.S.. M.....VS-- -------TS. .SV.V....

Ictidomys_tridecemlineatus LM..V..... ........TL .LF..KL... .........A ..I.K..... ...VVFY... .....S.S.. M.....MS-- -------TS. .SV.VF...

Jaculus_jaculus LM..V..... ........TL .LF..KL... .........A ..I...V... ...VVFY... .R...N.R.. M..V..MS-- -------TS. .SV.V....

Marmota_flaviventris LM..V..... ........TL .LF..KL... .........A ..I.K..... ...VVFY... .....S.S.. M.....MS-- -------TS. .SV.VF...

Marmota_marmota LM..V..... ........TL .LF..KL... .........A ..I.K..... ...VVFY... .....S.S.. M.....MS-- -------TS. .SV.VF...

Mastomys_coucha LM..V..... ....C...TL .LF..KL... .........A ..I....... ...VVF.... .....N.S.. ......IS-- -------TS. .SV.V....

Meriones_unguiculatus LM..V..... ........TL FLF..KL... .........A ..I....... ...AVS.S.. .R...S.S.. L.....MS-- -------AS. .SV.V....

Mesocricetus_auratus LM..V..... ........TL .LF..KL... .........A ..I....... .....LY... .....N.S.. M.....MS-- -------AS. .SV.V....

Microtus_ochrogaster LM..V..... ....L...TL FL...KL... .........A ..I....... ....VFY... ....NS.S.. M.....VS-- -------AS. .SV.V....

Mus_caroli LM..V..... ....S...TL FLF..KL... .........A ..I....T.. ...MVFY... .R...S.S.. M.....VS-- -------AS. .SV.V....

Mus_musculus LM..V..... ........TL FLF..KL... .........A ..I....V.. ...MVFY... .R...S.S.. M.....IS-- -------AS. .SV.V....

Mus_pahari LM..V..... ........AL .LF..KL..I .........A ..I....... ...MVFY... .R...S.S.. M..V..IS-- -------AS. .SV.V....

Mus_spicilegus LM..V..... ........TL FLF..KL... .........A ..I....... ...MVFY... .R...S.S.. M.....IS-- -------AS. .SV.V....

Nannospalax_galili LI..V..... ........TL FLF..KL... .........A ..I.K..V.. ...VVFY... .R...S.S.. M.....VS-- -------TP. .SV.V....

Octodon_degus LM..V..... ........SL .LF..KL... .........A ..I...V... ...VVFS... .....S.... L.....VS-- -------TS. .SV.V....

Onychomys_torridus LM..V..... ........TL FLF..KL... .........A ..I....... .....F.... .....N.S.. M..V..IS-- -------AS. .SI.V....

Peromyscus_maniculatus LM..V..... ........TL FLF..KL... .........A ..I....... .....FY... .....N.S.. M..V..IS-- -------AS. .SV.V....

Rattus_norvegicus LM..V...V. ........TL FLF..KL... .........A ..I.KV.... ...MVS.... .......S.. M.....TS-- -------VS. .SV.V....

Rattus_rattus LM..V...V. ........TL FLF..KL... .........A ..I.KV.... ...MVS.... .......... M.....TS-- -------VS. .SV.V....

Sciurus_vulgaris LM..V..... .V..L...TL .LF..KL... .........A ..I.K..... ....VFD... .....N.S.. M.....MS-- -------TS. .SV.VF...

Urocitellus_parryii LM..V..... ........TL .LF..KL... .........A ..I.K..... ...VVFY... .....N.S.. M.....MS-- -------TS. .SV.VF...

3.50 4.50 45.50 5.50

TM3 **$** <<< >>> TM4 **$** <<< **$** >>> TM5 **$** <<<

P G GG G P P P P G GG G

Monodelphis_domestica FVGANYSWLL VEGIYLHTLL GPLVLSQRRL LLRYIMVGWA FPVLYMIPWG ITRARLENIG CWGTNTNMNI WWIIRGPILL SIIINFFIFL KILKLLISK

Phascolarctos_cinereus .......... .......... .......K.. .P...L.... .....VV..W ....K...T. .....K..K. .......... ..T....... .........

Sarcophilus_harrisii .......... .......... .......K.. .P...LL... .....VV..C ....Q...T. .....K..K. .......M.. ..TV...... ...R.....

Trichosurus_vulpecula .......... .......... .......K.. .P...L.... .....VV..W ....K...T. .....K..K. .......... ..TV...... .........

Vombatus_ursinus .......... .......... .......K.. .P...L.... .....VV..W ....K...T. .....K..K. .......... ..TV...... .........

Choloepus_didactylus .......... ...L...... ..TL.FE... WPT.ML...G Y...FVV..C VA..Q...T. ...I.G.K.. .......M.. CVTV...... .........

Dasypus_novemcinctus ..V..Q.... ...L..Y... K.T...E... WPKFML...G Y...FVV..C VA.VQ...K. ..MK.K..K. .......... CVTV..V... YTV......

Chrysochloris_asiatica C......... ...L...I.. ..TL..E... WPT.LL...G ....SVL... .V..Q...T. .....G.KL. .......V.. C.TV..L... .....I...

Elephantulus_edwardii .......... ...L...A.. ..SL.FE... WPS.LL...G .....VV..S .V..H...K. .....E.KK. .......M.. CVTV...... .........

Loxodonta_africana .......... ...L...... ..TM..E... WP..LL...G ....SVV... .V..Q...T. .....G.KK. .......V.. CVTV...... ......V..

Orycteropus_afer .......... ...L..N... ..TM..E... WP..LL...G ....SVV..S .V..Q...T. .....G.KK. .......V.. C.TV...... .........

Trichechus_manatus .......... ...L...... ..T...E... WP..LL...G ....SVV..S .V..H...T. .....G.K.. .......V.. C.TV...... .........

Balaenoptera_acutorostrata .......... ...L...... E.I...E... WP..LL.... ....FVV..S .A..Q...T. .....R.K.. .......M.. CVTV...... .........

Balaenoptera_musculus .......... ...L...... E.I...E... WP..LL.... ....FVV..S VA..Q...T. .....R.K.. .......M.. CVTV...... .........

Delphinapterus_leucas .......... ...L...... E.I...E... WP..LL.... ....FVV..S .A..Q...T. .....R.K.V .......M.. CVTV...... .........

Globicephala_melas .......... ...L...... E.I...E... WP..LL.... ....FVV..S .A..Q...T. .....R.K.V .......M.. CVTV...... .........

Lagenorhynchus_obliquidens .......... ...L...... E.I...E... WP..LL.... ....FVV..S .A..Q...T. .....R.K.V .......M.. CVTV...... .........

Lipotes_vexillifer .......... ...L...... E.T...E... WP..LL.... ....FVV..S .A..Q...T. .....R.K.V .......L.. CVTV...... .........

Monodon_monoceros .......... ...L...... E.I...E... WP..LL.... ....FVV..S .A..Q...T. .....R.K.V .......M.. CVTV...... .........

Neophocaena_asiaeorientalis .......... ...L...... E.I...E... WP..LL.... ....FVV..S .A..Q...T. .....R.K.V .......M.. CVTV...... .........

Orcinus_orca .......... ...L...... E.I...E... WP..LL.... ....FVV..S .A..Q...T. .....R.K.V .......M.. CVTV...... .........

Phocoena_sinus .......... ...L...... E.I...E... WP..LL.... ....FVV..S .A..Q...T. .....R.K.V .......M.. CVTV...... .........

Tursiops_truncatus .......... ...L...... E.I...E... WP..LL.... ....FVV..S .A..Q...T. .....R.K.V .......M.. CVTV...... .........

Bison_bison ...T...... ...L...... E.IM..E... WP..LL...G ....FVV..S VA..Q...T. ...N.R.K.. .......MM. C.TV...... .........

Bos_mutus ...T...... ...L...... E.IM..E... WP..LL...G ....FVV..S .A..Q...T. ...N.R.K.. .......MM. C.TV...... .........

Bos_taurus ...T...... ...L...... E.IM..E... WP..LL...G ....FVV..S .A..Q...T. ...N.R.K.. .......MM. C.TV...... .........

Bubalus_bubalis ...T...... ...L...... E.IM..E... WP..LL...G ....FIV..S .A..Q...T. ...N.R.K.. .......MM. C.TV...... .........

Camelus_dromedarius .......... ...L..R... E.T...E.W. WP..LL.... ....FVV..S .A..Q...T. .....R.KS. .......M.. CVMV...... .........

Camelus_ferus .......... ...L..R... E.T...E.W. WP..LL.... ....FVV..S .A..Q...T. .....R.KS. .......M.. CVMV...... .........

Capra_hircus .......... ...L..D... E.I.F.E... WP..LL.... ....FVV..S .A..Q...T. .....R.K.. .......M.. C.MV...... .........

Catagonus_wagneri .......... ...L...... E.IA..E... WP..LL.... ....FVV..S VA..Q..DT. .....R.K.. .......MM. CVMV...... .........

Cervus_hanglu ...T...... ...L...... E.I.F.E... WP..LL.... ....FVV..S .V..W...T. ...N.K.KK. .......MM. CVTV...... .........

Moschus_moschiferus .......... ...L...... E.I...EK.. WP..LL.... ....FVV..S .A..Q...T. .....R.K.. .......M.. CVMV...... .........

Sus_scrofa .......... ...L...... E.IA..E... WP..LL.... ....FVV..S VA..K...T. ..VN.R.K.. .......MM. CVMV...... .........

Vicugna_pacos .......... ...L..R... E.IA..E... WP..LL.... ....SVV..S .A..Q...T. ..V..R.KS. .......M.. CVMV...... .........

Ailuropoda_melanoleuca .......... ...L...... E.T...E... WP..ML.... ..L.FVV..S .A..Q...T. ...I.R.KK. .......M.. CVMV...... ..VR.....

Callorhinus_ursinus .....H.... ...L...... E.T...E... WP..ML.... ..L.FVV..S .A..Q...T. .....R.KK. .......M.. YVMV...... ..V......

Canis_lupus .......... ...L...... E.T...E... WP..ML.... ..L.FVV..S ....Q...T. ..V..R.KK. .......M.. CVMV...... ..V......

Enhydra_lutris .......... ...L...... E.T...E... WP..ML.... ....FVV..S MA..Q...T. .....K.KK. .......M.. CVMV...... ..V......

Eumetopias_jubatus .....H.... ...L...... E.T...E... WP..ML.... ..L.FVV..S .A..Q...T. .....R.KK. .......M.. YVMV...... ..V......

Felis_catus .......... ...L...... E.T...E... WP..ML.... ..L.FVV..S .A..Q...T. .....R.KK. .......M.. CVMV...... ..V......

Lynx_canadensis .......... ...L...... E.T...E... WP..ML.... ..L.FVV..S VA..Q...T. .....R.KK. .......M.. CVMV...... ..V......

Mirounga_leonina .....H.... ...L...... E.T...E... WP..ML.... ..L.FVV..S .A..Q...T. .....R.KI. .......M.. YVMV...... ..V......

Mustela_erminea .......... ...L...... E.T...E... WP..ML.... ....FVV..S MA..Q...T. .....KSKK. .......M.. CVMV...... ..V......

Mustela_putorius .......... ...L...... E.T...E... WP..ML.... ....FVV..S MA..Q...T. .....KSKK. .......M.. CVMV...... ..V......

Neomonachus_schauinslandi .....H.... ...L...... E.T...E... WP..ML.... ..L.FVV..S .A..Q...T. .....R.KI. .......M.. YVMV...... ..V......

Odobenus_rosmarus .....H.... ...L...... E.T...E... WP..ML.... ..L.FVV..S .A..Q...T. .....R.KK. .......M.. YVMV...... ..V......

Panthera_pardus .......... ...L...... E.T...E... WP..ML.... ..L.FVV..S .A..Q...T. .....R.KK. .......M.. CVMV...... ..V......

Phoca_vitulina .....H.... ...L...... E.T...E... WP..ML.... ..L.FVV..S .A..Q...T. .....R.KI. .......M.. YVMV...... ..V......

Suricata_suricatta .......... ...L...A.. E.T...EK.. WP..ML.... ..L.FVA..S VA..Q...T. .....R.KK. .......M.. CVMV...... ..V......

Ursus_thibetanus .......... ...L...... E.T...E... WP..ML.... ..L.FVA..S .A..Q...T. ...I.R.KK. .......M.. CVMV...... ..V......

Vulpes_vulpes .......... ...L...... E.T...E... WP..ML.... ..L.FVV..S ....Q...T. ..V..R.KK. .......M.. CVMV...... ..V......

Zalophus_californianus .....H.... ...L...... E.T...E... WP..ML.... ..L.FVV..S .A..Q...T. .....R.KK. .......M.. YVMV...... ..V......

Eptesicus_fuscus .......... ...L..R... E.T...E... WPT.LL.... ..L.FVV..S .A..Q...T. ..V..K.KE. .......M.. CVAV...... .........

Miniopterus_natalensis ...T...... ...L...... E.TM..E... WPT.LL.... ..L.FVV... .A..Q..DT. ...I.R.KK. .......M.. CVAV...... .........

Myotis_lucifugus V......... ...L..R... E.TA..E... WPT.LL...G ..L.FVV... .A..Q...T. ..VN.R.KA. .......... C.AV...... .........

Myotis_myotis V......... ...L..R... E.T...E... WPT.LL...G ..L.FVV... .A..Q...T. ..VN.R.KA. .......M.. CVAV...... .........

Pipistrellus_kuhlii ...T...... ...L..R... E.T...E... WPT.LL.... ..L.FVV..C .A..Q...T. ..V..E.KG. .......M.. CVAV...... .........

Pteropus_alecto .......... ...L..Y... E.T.I.E.W. WP..LL.... ..L.FVV..S .A..Q...T. ...I.G..K. .......M.. CVAV...... .........

Rhinolophus_ferrumequinum ..A....... ...L...... E.I.HPE... WP..LL.... ..L.FVV..S .A..Q...T. .....G.KK. .......M.. CVAV...... .........

Sturnira_hondurensis ........F. ...L...... E.T...G... WP..LL.... S.L.FVV..S VA..Q...T. ...I.N.KP. .......M.. CVAV...F.. .........

Condylura_cristat ...T...... ...L...S.. D.T.F.E... WPT.LLL... ....FVV..V .V.FQ...T. ..RN.K..M. ........MF CVSV...L.M .........

Sorex_araneus ...T...... ...L...W.. E.I.HFE.W. WP..LL.... ....FVA... .V..K...T. ...N.N..K. .......MMF CVAV.....M .........

Talpa_occidentalis .T.T...... ...L...S.. D.T.F.E... WPT.LLL... ....FVV..I .V.FQ...T. ..I-.R..K. .......MMF CVAV...L.M .........

Ceratotherium_simum .......... ...L...... E.T...E... WP..LL.... ....FVV..S .A..Q...T. .....G.KK. .......M.. CVMV...... .........

Equus_caballus .......... ...L...... E.T...E... WP..LL.... ....FVV..S .A..H...T. .....S.KK. .......M.. CVTV...... .........

Manis_javanica .......... ...L...... E.T...E... WP..LL...V ..L.FV...S .A.TQ...T. ..V..R.KKV .......M.. YVMV...... .........

Manis_pentadactyla .......... ...L...... E.T...E... WP..LL...V ..L.FV...S .A.TQ...T. ..V..R.KKV .......M.. YVLV...... .........

Prolemur_simus .....H.... ...L...A.. E.T..PS... WP..LL.... ...FFVV... .A..H...T. ..A..G.KTV ..M....L.. CVTV.....V .........

Tupaia_chinensis .....HL... ...L...A.. E.T.FPE.H. WPK.LLL... ....FVV... FA..H...T. ..A..A.KK. .......M.. CVAV...... .........

Aotus_nancymaae ......L... ...L...A.. E.T..PE... WP..LLL... ....FVV... FA..H...T. ..T..G.KK. .......... CVTV...... .........

Callithrix_jacchus ......L... ...L...... E.T..PE... WP..LLL... ....FVV... FA..H...T. ..T..G.KK. .......... CVTV...... .........

Carlito_syrichta ..ST.HL... ...H...A.. E.T.IPN.W. WP..LL...V C.M.FVV... FA..H...S. ..AQ.K.KE. ........M. C.MV...... ...R.....

Cebus_capucinus ......L... ...L...A.. E.T..PE... WP..LLL... ....FVV... FA..H...T. ..T..G.KK. .......... CVTV...... .........

Cercocebus_atys S.....L... ...L...A.. E.T..PE... WP..LLL... ....FVV... FA..H...T. ..T..G.KK. .......M.. CVTV...... .........

Chlorocebus_sabaeus S.....L... ...L...A.. E.T..PE... WP..LLL... ....FVV... FA..H...T. ..T..G.KK. .......M.. CVTV...... .........

Colobus_angolensis S.....L... ...L...A.. E.T..PE... WP..LLL... ....FVV... FA..H...T. ..T..G.KK. .......M.. CVTV...... .........

Gorilla_gorilla ......L... ...L...... E.T..PE... WP..LLL... ....FVV... FA..H...T. ..T..G.KK. .......MM. CVTV...... .........

Homo_sapiens ......L... ...L...... E.T..PE... WP..LLL... ....FVV... FA..H...T. ..T..G.KK. .......MM. CVTV...... .........

Hylobates_moloch ......L... ...L...... E.T..PE... WP..LLL... ....FVV... FA..H...T. ..T..G.KK. .......... CVTV...... .........

Macaca_fascicularis S.....L... ...L...A.. E.T..PE... WP..LLL... ....FVV... FA..H...T. ..T..G.KK. .......M.. CVTV...... .........

Macaca_mulatta S.....L... ...L...A.. E.T..PE... WP..LLL... ....FVV... FA..H...T. ..T..G.KK. .......M.. CVTV...... .........

Macaca_nemestrina S.....L... ...L...A.. E.T..PE... WP..LLL... ....FVV... FA..H...T. ..T..G.KK. .......M.. CVTV...... .........

Mandrillus_leucophaeus S.....L... ...L...A.. E.T..PE... WP..LLL... ....FVV... FA..H...T. ..T..G.KK. .......M.. CVTV...... .........

Microcebus_murinus .....H.... ...L...A.. E.S..PS... WA..LL.... ....FVV... .A..H...T. ..A..G.KTV .......L.F CVTV.....V .........

Nomascus_leucogenys ......L... ...L...... E.T..PE... WP..LLL... ....FVV... FA..H...T. ..T..G.KK. .......... CVTV...... .........

Otolemur_garnettii .....HL... ...L...A.. E.T..PK... WP..LL.... ....FVV... .A..H...T. ..A..G.KT. .......M.. C.TV...... .........

Pan_paniscus ......L... ...L...... E.T..PE... WP..LLL... ....FVV... FA..H...T. ..T..G.KK. .......MM. CVTV...... .........

Pan_troglodytes ......L... ...L...... E.T..PE... WP..LLL... ....FVV... FA..H...T. ..T..G.KK. .......MM. CVTV...... .........

Piliocolobus_tephrosceles S.....L... ...L...A.. E.T..PE... WP..LLL... ....FVV... FA..H...T. ..T..G.KK. .......M.. CVTV...... .........

Pongo_abelii ......L... ...L...... E.T..PE... WP..LLL... ....FVV... FA..H...T. ..T..G.KK. .......M.. CVTV...... .........

Propithecus_coquereli .....H.... ...L...A.. E.T..PS... WP..LL.... ....FVV... .A..H...T. ..A..G.KTV .......L.. CVTV.....V .........

Rhinopithecus_bieti S.....L... ...L...A.. E.T..PE... WP..LLL... ....FVV... FA..H...T. ..T..G.KK. .......M.. CVTV...... .........

Rhinopithecus_roxellana S.....L... ...L...A.. E.T..PE... WP..LLL... ....FVV... FA..H...T. ..T..G.KK. .......M.. CVTV...... .........

Saimiri_boliviensis ......L... ...L...... E.T..PE... WP..LLL... ....FVG... FA..H...T. ..T..G.KK. .......... CVTV...... .........

Sapajus_apella ......L... ...L...A.. E.T..PE... WP..LLL... ....FVV... FA..H...T. ..T..G.KK. .......... CVTV...... .........

Theropithecus_gelada S.....L... ...L...A.. E.T..PE... WP..LLL... ....FVV... FA..H...T. ..T..G.KK. .......M.. CVTV...... .........

Arvicanthis_niloticus .....HL... ...L...A.. E.T..PE... WPK.LV.... ..M.FV.... FA..H...T. ..AI.G.KA. .......M.. CVTV...... .........

Arvicola_amphibius .....HFG.. ...L...A.. E.T..PE... WP..LV.... ..M.FV.... F...H...T. ..TS.A.KK. .......M.. CVTV...... .........

Castor_canadensis .....HL... ...L...A.. E.T..PE... WP..LV.... ....FVV... .A..H...E. ..AK.E.KE. .......M.. CVTV...... .........

Cavia_porcellus .....HL... ...L...A.. K.T..PE... WP..LV.... ....FGV..A G...L...S. ..A....TK. .......M.. CVTV..L... .........

Cricetulus_griseus .....HL... ...L...A.. E.T..PE... WP..LV.... ..M.FV.... FA..H...T. ..A..G.KK. .......M.. CVTV...... .........

Fukomys_damarensis .....HL... ...L...A.. E.T..PE... WP..LV.... ....FVV... .A..L...T. ..A..G.KI. .......M.. CVMV...... .........

Grammomys_surdaster .....HL... ...L...A.. E.T..PE... WPK.LV.... ..M.FV.... FA..H...K. ..AI.G.KK. .......M.. CVTV...... .........

Heterocephalus_glaber .....HL... ...L...A.. E.T..PE... WP..LV.... ..M.FVV... .A..L...M. ..A..G.KK. .......M.. CVTV...... .........

Ictidomys_tridecemlineatus C....HL... ...L...A.. E.T..PE... WP..LV.... ....FVV... FA..H...T. ..A..G.KK. .......MM. CVMV...... ...R.....

Jaculus_jaculus .....HL... ...L...A.. E.T..PE... WP..LV.... ....FVV... FA..H...T. ..A..G.KK. .......M.. CVTV...... .........

Marmota_flaviventris C....HL... ...L...A.. E.T..PE... WP..LV.... ....FVV... FA..H...T. ..A..G.KK. .......MM. CVMV...... ...R.....

Marmota_marmota C....HL... ...L...A.. E.T..PE... WP..LV.... ....FVV... FA..H...T. ..A..G.KK. .......MM. CVMV...... ...R.....

Mastomys_coucha S.S..NL... ...L...S.. E.T..PE... WPK.LVI... ..M.SV.... FA..H...K. ..AR.E.KK. .......M.. CVTV...... .........

Meriones_unguiculatus .....HL... ...L...A.. A.T..PE... WP..LV.... ..M.FVV..S FA.TH..... ..A..G.KK. .......M.. CVTV...... .........

Mesocricetus_auratus .....HL... ...L...A.. E.T..PE... WP..LV.... ..M.FV.... FA..H...T. ..A..G.KK. .......M.. CVTV...... .........

Microtus_ochrogaster .....HL... ...L...A.. E.T..PE... WP..LV.... ..M.FV.... F...H...T. ..TS.A.KK. .......M.. CVTV...... .........

Mus_caroli .....HL... ...L...A.. E.T..PE.G. WPK.LLL... ..M.FV...V FA..S...T. ..AV.E.KK. .......N.. CVTV..L... .........

Mus_musculus ...T.HL... ...L...A.. E.T..PE... WPK.LV.... ..M.FV...I FV..S...T. ..AV.E.KK. .......... CVTV...... .........

Mus_pahari ......L... ...L...A.. E.T..PE... WPK.LV.... ..M.FV.... FA..S...T. ..AI.E.KK. .......M.. CVTV...... .........

Mus_spicilegus .....HL... ...L...A.. E.T..PE... WPK.LV.... ..M.FV...V FA..S...T. ..AI.E.KK. .......M.. CVTV...... .........

Nannospalax_galili .....HL... ...L...A.. E.T..PE... WP..LV.... ..T.FVV... FA..H..... ..A..G.KK. .......M.. CVTV...... .........

Octodon_degus .....HL... ...L...A.. E.T..PE... WR..LV.... ....FVV... .A..L...T. ..A..G.KK. .......M.. CVTV...... .........

Onychomys_torridus .....HL... ...L...A.. E.T..PE... WP..LV.... ..M.FV.... FA..H..... ..A..G.KK. .......M.. CVTV...... .........

Peromyscus_maniculatus .....HL... ...L...A.. E.T..PE... WP..LV.... ..M.FV.... FA..H...T. ..A..G.KK. .......M.. CVTV...... .........

Rattus_norvegicus ...T.HL... ...L...... E.T.FPE... WPK.LV.... ..M.FV.... FA..H...TR ..A..G.LK. .......M.. CVTV...... .........

Rattus_rattus ...T.HL... ...L...... D.S.FPE... WPK.LV.... ..M.FV.... FA..H...TR ..A..G.LK. .......M.. CVTV...... .........

Sciurus_vulgaris C....HL... ...L...A.. E.T..PE... WP..LV.... ....FVV... FA..H...T. ..A..G.KK. .......M.. CVMV...... ...R.....

Urocitellus_parryii C....HL... ...L...A.. E.T..PE... WP..LV.... ....FVV... FA..H...T. ..A..G.KK. .......MM. CVMV...... ...R.....

6.50 7.50 8.50

>>> TM6 **$** <<< > >> TM7 **$** <<< **$**

G G G GG P P PP G GG

Monodelphis_domestica LKAHQLAFSD YKYRLARSTL VLIPLLGVHE IVFSFIIDEQ VKGSQRHIRL FIQLTMSSFH GLLVAFLYCF ANEEVKAELR KYWIRFLLA- HHISCVGCV

Phascolarctos_cinereus .....MC... .......... .......... .....VT... .Q..PK.... .......... .......... .........Q .H.......- .........

Sarcophilus_harrisii .....MC... .......... ........Y. VIC...T... .EDFS..... .......... .......... .......... .H.......- .........

Trichosurus_vulpecula .....MC... .T........ .......... .....VT.D. ....SK.... .......... .......... .......... .H.......- .........

Vombatus_ursinus .....MC... .T........ .......... .....VT... .Q..PK.... .......... .......... .......... .H.......- .........

Choloepus_didactylus .....MC.R. ......K... .......... .L.L..T... .Q.FPKLV.. .....L.... ......Q... ..G....... RH.L.Y...- .RSG.IAW.

Dasypus_novemcinctus .....MC.R. ......K..F F..S.....D L.YY..T... ...YSK.... Y..MSVG... .....YH... ..G....... RH.PH...D- LCHG.RVW.

Chrysochloris_asiatica ....KMC.R. ..F...K... .......... .L....T... .Q.LSKL... ....AL.... .......... .......... .H....M.V- ..SG.RTWF

Elephantulus_edwardii .....MC.RH ......K... .......... .L....T.D. .E.FA.L... .V..AL.... .......... ..G...S... .H.A.....- .LSG.RAWF

Loxodonta_africana .....MC.R. ......K... .......... .L....T... .Q.FSKL... ....AL.... ......Q... ..G....... .H.V.....- ..SG.RAWF

Orycteropus_afer .....MC.R. ......K... ........Q. .L....T... LQ.FSKL... ....AL.... ......Q... ..G....... .H.V.....- R.SG.RDWF

Trichechus_manatus .....MC.R. ......K... .......... .L....T... .Q.FSKL... ....AL.... ......Q... ..G....... .H.V.....- ..SG.RAWF

Balaenoptera_acutorostrata .....MC.R. ......K... .......... .L....T.D. .E.FP.Y... .....L.... .....LQ... ..G....... .Q.A.....- ..SG.RAW.

Balaenoptera_musculus .....MC.R. ......K... .......... .L....T.D. .E.FP.Y... .....L.... .....LQ... ..G....... .Q.A.....- ..SG.RAW.

Delphinapterus_leucas .....MC.R. ......K... .......... .L....T.D. .E.FP.Y... .....L.... .....LQ... S.G....... .Q.A.....- ..SG.RAW.

Globicephala_melas .....MC.R. ......K... .......... .L....T.D. .E.FP.Y... .....L.... .....LQ... S.G....... .Q.A.....- ..SG.RAW.

Lagenorhynchus_obliquidens .....MC.R. ......K... .......... .L....T.D. .E.FP.Y... .....L.... .....LQ... S.G....... .Q.A.....- ..SG.RAW.

Lipotes_vexillifer .....MC.R. ......K... .......... .L....T.D. .E.FP.Y... .....L.... .....LQ... ..G....... .Q.A.....- ..SG.RAWA

Monodon_monoceros .....MC.R. ......K... .......... .L....T.D. .E.FP.Y... .....L.... .....LQ... S.G....... .Q.A.....- ..SG.RAW.

Neophocaena_asiaeorientalis .....MC.R. ......K... .......... .L....T.D. .E.FP.Y... .....L.... .....LQ... S.G....... .Q.A.....- ..SG.RAW.

Orcinus_orca .....MC.R. ......K... .......... .L....T.D. .E.FP.Y... .....L.... .....LQ... S.G....... .Q.A.....- ..SG.RAW.

Phocoena_sinus .....MC.R. ......K... .......... .L....T.D. .E.FP.Y... .....L.... .....LQ... S.G....... .Q.A.....- ..SG.RAW.

Tursiops_truncatus .....MC.R. ......K... .......... .L....T.D. .E.FP.Y... .....L.... .....LQ... S.G....... .Q.A.....- ..SG.RAW.

Bison_bison .....MC.R. ......K... .........D .L....T.D. .E.FP..... .....LG... .....SQ... ..G....... .Q.A.....- ..SG.RAW.

Bos_mutus .....MC.R. ......K... .........D .L....T.D. .E.FP..... .....LG... .....SQ... ..G....... .Q.A.....- ..SG.RAW.

Bos_taurus .....MC.R. ......K... .........D .L....T.D. .E.FP..... .....LG... .....SQ... ..G....... .Q.A.....- ..SG.RAW.

Bubalus_bubalis .....MC.R. ......K... .........D .L....T.D. .E.FP.L... .....LG... .....SQ... ..G....... .Q.A.....- R.SG.RAW.

Camelus_dromedarius .....MC.R. ......K... .......... .L....T.D. .E.FSKL... .....L.... .....LQ... ..G....... .Q.A.....- ..SG.RAW.

Camelus_ferus .....MC.R. ......K... .......... .L....T.D. .E.FSKL... .....L.... .....LQ... ..G....... .Q.A.....- ..SG.RAW.

Capra_hircus .....MC.R. ......K... .......... .L....T.D. .E.FP.L... .....L.... .....SQ... T.G....... .Q.A.....- ..SG.RAW.

Catagonus_wagneri .....MC.R. ......K... .......... ......T.D. .E.FSKLV.. .....L.... .....LQ... ..G....... .Q.V.....- .SS..RTW.

Cervus_hanglu .R...MC.R. ......K... .......... .L....T.D. .E.FP.L... Y....LG... .....WQ... ..G....... .Q.A.....- ..SG.RAW.

Moschus_moschiferus .....MC.R. ......K... .......... ......T.D. .E.FP.L... .....L.... .....LQ... ..G......Q .H.A.....- ..LG.RAW.

Sus_scrofa .....MC.R. ......K... .......... ...A.VT.D. .E.FAKLV.. .....L.... .....LQ... ..G....... .Q.A.....- .YSG.RAW.

Vicugna_pacos .....MC.R. ......K... .......... .L....T.D. .E.FSKL... .....L.... .....LQ... ..G....... .Q.A.....- ..SG.RAW.

Ailuropoda_melanoleuca .....MC.R. ..F...K... .......... .L....T.D. .E.FSKL... .....L.... ...M.LQ... ..G....... .H.V.....- R.SG.RAW.

Callorhinus_ursinus .....MC.R. ......K... .......... .L....T.D. .E.ISKL... .....L.... ...M.LQ... ..G....... .H.V.....- R.SG.RAW.

Canis_lupus .....MC.R. ......K... .......... .L....T.D. .E.FSKL... .M...L.... .....LQ... ..G....... .H.V.....- R.SG.SAW.

Enhydra_lutris .....MC.R. ......K... .......... .L...VT.D. .E.FSKL... .....L.... .....LQ... ..G....... .H.V.....- R.SG.RAW.

Eumetopias_jubatus .....MC.R. ......K... .......... .L....T.D. .E.ISKL... .....L.... ...M.LQ... ..G....... .H.V.....- R.SG.RAW.

Felis_catus .....MC.R. ......K... .......... .L....T.D. .E.FSKL... .....L.... .....LQ... ..G......H .H.V.....- R.SG.RAW.

Lynx_canadensis .....MC.R. ......K... .......... .L....T.D. .E.FSKL... .....L.... .....LQ... ..G....... .H.V.....- R.SG.RAW.

Mirounga_leonina .....MC.R. ......K... .......... .L....T.D. .E.FSKL... .....LG... ...M.LQ... ..G....... .H.V.....- R.SG.RAW.

Mustela_erminea .....MC.R. ......K... .......... .L...VT.D. .E.FSKL... .....L.... .....LQ... ..G......Q .H.V.....- R.SG.RAW.

Mustela_putorius .....MC.R. ......K... .......... .L...VT.D. .E.FSKL... .....L.... .....LQ... ..G....... .H.V.....- R.LG.RAW.

Neomonachus_schauinslandi .....MC.R. ......K... .......... .L....T.D. .E.FSKL... .....LG... ...M.LQ... ..G....... .H.V.....- R.SG.RAW.

Odobenus_rosmarus .....MC.R. ......K... .......... .L....T.D. .E.ISKL... .....L.... ...M.LQ... ..G....... .H.V.....- R.SG.RAW.

Panthera_pardus .....MC.R. ......K... .......... .L....T.D. .E.FSKL... .....L.... .....LQ... ..G....... .H.V.....- R.SG.RAWA

Phoca_vitulina .....MC.R. ......K... .......... .L....T.D. .E.FSKL... .....LG... ...M.LQ... ..G....... .H.V.....- R.SG.RVW.

Suricata_suricatta .....MC.K. ..C...K... .......... .L....T.D. .E.FSKL... .....L.... .F...LQ... ..G....... .Q.V.....- RSSG.RA..

Ursus_thibetanus .....MC.R. ..F...K... .......... ......T.D. .E.FSKL... .....L.... ...M.LQ... ..G....... .H.V.....- R.SG.RAW.

Vulpes_vulpes .....MC.R. ......K... .......... VL....T.D. .E.FSKL... .M...L.... .....LQ... ..G....... .H.V.....- R.SG.SAW.

Zalophus_californianus .....MC.R. ......K... .......... .L....T.D. .E.ISKL... .....L.... ...M.LQ... ..G....... .H.V.....- R.SG.RAW.

Eptesicus_fuscus .....MC.K. ......K... .......... .L...VT.D. .E.FSKL... .....L.... .....LQ... V.G....... RH.G.....- ..SG.RAWG

Miniopterus_natalensis .....TC.R. ......K... .......... .L....T.D. .Q.FSKL... .V...L.... .....LQ... ..G....... RH.L.....- ..SG.RAWA

Myotis_lucifugus .....MC.K. .R....K... .......... .L...VT.D. .Q.FS.LV.. Y....L.... .....LQ... V.G....... RH.G.....- ..SG.RAWG

Myotis_myotis .....MC.K. .R....K... .......... .L...VT.D. .Q.FS.LV.. .....L.... .....LQ... V.G....... RH.G.....- ..SG.RAWG

Pipistrellus_kuhlii .....MC.K. ......K... .......... .L...VT.D. .Q.FSKLV.. .....L.... .....LQ... V.G....... RH.G.....- ..SG.RAWG

Pteropus_alecto .....MC.R. ......K... .......... .L....T.D. .Q.FSKL... .....L.... .....LQ... ..G....... .H.V.....- R.SG.RAW.

Rhinolophus_ferrumequinum .....MC.R. ......K... .......... VL....T.D. .Q.FSKL... .....L.... .....LQ... ..G....... .H.V.....- .PLG.RAW.

Sturnira_hondurensis .....MC.R. ......K... .......... VI....S.D. .Q.FS.L... .....L.... .....LQ... ..G....... ...V.....- ..L..RAW.

Condylura_cristat .....MC.R. .R....K... .......... L..F.VT.DH .F.FSKLV.. .F...LG.V. .FI..L.... ..G......W .H.F.....- P.SGYRARF

Sorex_araneus ...R.MC.K. ......K... .......... MI....T.DH IT.FS..L.. IF...L.... .F...L.... ..G...T... .H.V.....- RESG.RAW.

Talpa_occidentalis .....MC.R. .R....K... .......... L..F.VT.DH IL.FPKLV.. Y....LG.V. ..I..L.... ..G......W .H.F.....- P.SGYRAWF

Ceratotherium_simum .....MC.R. ......K... .......... .L.T..T.D. .E.FSKL... .....L.... .....LQ... ..G....... .H.V.....- R.SG.RAW.

Equus_caballus .....MC.R. ......K... .......... .L.T.VT.D. .E.FSKL... .....L.... .....LQ... ..G....... .H.V.....- R.SG.RAW.

Manis_javanica .....MC.R. ......K... .......... .L....T.D. .E.FS.I... .....L.... .F...LQ... ..G......Q .H.V.....- ..SG.TAW.

Manis_pentadactyla .....MC.R. ......K... .......... .L....T.D. .E.FS.I... .....L.... .F...LQ... ..G......Q .H.V.....- ..SG.TAW.

Prolemur_simus .....MC.R. ......K... .......... .L....T.D. .Q.FSKLV.. .....L.... .F...LQ.G. ..G....... .H.V.....- R.SG.RA.G

Tupaia_chinensis .....MC.R. ......K.A. .......... VL....T.D. .Q.FSKLV.. .....L.... .F...WQ.G. .SG....... .Q.V.....- D.SG.GAW.

Aotus_nancymaae .....MC.R. ......K... .......... .L....T.D. .E.FAKLV.. .....L.... .F...LQ.G. ..G....... .........- R.SG.RA..

Callithrix_jacchus .....MC.R. ......K... .......... .L....T.D. .E.FAKLV.. .....L.... .F...LQ.G. ..G....... .........- R.SG.RA..

Carlito_syrichta .....MC.R. ......K... .......... .L...LT.D. .E.FP.V... .....LG... .F...L.FG. ..G......W ...V.....- .NLG.RAFF

Cebus_capucinus .....MC.R. ......K... .......... .L....T.D. .E.FAKLV.. .....L.... .F...LQ.G. ..G....... .........- R.SG.RA..

Cercocebus_atys ...N.MC.R. ......K... .......... .L...VT.D. .E.FAKL... .....L.... .F...LQ.G. .SG....... ...V.....- ..AG.RA..

Chlorocebus_sabaeus ...N.MC.R. ......K... .......... .L....T.D. .E.FAKL... .....L.... .F...LQ.G. .SG....... ...V.....- R.AG.RA..

Colobus_angolensis ...N.MC.R. ......K... .......... .L....T.D. .E.FAKL... .....L.... .F...LQ.G. .SG....... ...V.....- R.AG.RA..

Gorilla_gorilla .....MC.R. ......K... .......... .L....T.D. .E.FAKL... .....L.... .F...LQ.G. ..G....... ...V.....- R.SG.RA..

Homo_sapiens .....MC.R. ......K... .......... .L....T.D. .E.FAKL... .....L.... .F...LQ.G. ..G....... ...V.....- R.SG.RA..

Hylobates_moloch .....MC.R. ......K... .......... .L....T.D. .E.FAKL... .....L.... .F...LQ.G. ..G....... ...V.....- R.SG.RA..

Macaca_fascicularis ...N.MC.R. ......K... .......... .L....T.D. .E.FAKL... .....L.... .F...LQ.G. .SG....... ...V.....- R.AG.GA..

Macaca_mulatta ...N.MC.R. ......K... .......... .L....T.D. .E.FAKL... .....L.... .F...LQ.G. .SG....... ...V.....- R.AG.RA..

Macaca_nemestrina ...N.MC.R. ......K... .......... .L....T.D. .E.FAKL... .....L..L. .F...LQ.G. .SG....... ...V.....- R.AG.RA..

Mandrillus_leucophaeus ...N.MC.R. ......K... .......... .L....T.D. .E.FAKL... .....L.... .F...LQ.G. .SG....... ...V.....- R.AG.RA..

Microcebus_murinus .....MC.R. ......K... .......... .L....T.D. .Q.FSKLV.. .....L.... .F...LQ.G. ..G....... .H.V.....- R.SG.RA.G

Nomascus_leucogenys .....MC.R. ......K... .......... .L....T.D. .E.FAKL... .....L.... .F...LQ.G. ..G....... ...V.....- R.SG.RA..

Otolemur_garnettii .....MC.R. ......K... .......... .L....T.D. .Q.FSKL... .....L.... .F...LQ.G. ..G....... .H.V.....- R.SG.RA.G

Pan_paniscus .....MC.R. ......K... .......... .L....T.D. .E.FAKL... .....L.... .F...LQ.G. ..G....... ...V.....- R.SG.RA..

Pan_troglodytes .....MC.R. ......K... .......... .L....T.D. .E.FAKL... .....L.... .F...LQ.G. ..G....... ...V.....- R.SG.RA..

Piliocolobus_tephrosceles ...N.MC.R. ......K... .......... .L....T.D. .E.FAKL... .....L.... .F...LQ.G. .SG....... ...V.....- R.AG.RA..

Pongo_abelii .....MC.R. ......K... .......... .L....T.D. .E.FAKL... .....L.... .F...LQ.G. ..G....... ...V.....- R.SG.RA..

Propithecus_coquereli .....MC.R. ......K... .......... .L....T.D. .Q.FSKLV.. .....L.... .F...LQ.G. ..G....... .H.V.....- R.SG.RA.G

Rhinopithecus_bieti ...N.MC.R. ......K... .......... .L....T.D. .E.FAKL... .....L.... .F...LQ.G. .SG....... ...V.....- R.AG.RA..

Rhinopithecus_roxellana ...N.MC.R. ......K... .......... .L....T.D. .E.FAKL... .....L.... .F...LQ.G. .SG....... ...V.....- R.AG.RA..

Saimiri_boliviensis .....MC.R. ......K... .......... .L....T.D. .E.FAKLV.. .....L.... .F...LQ.G. ..G....... .........- R.SG.RA..

Sapajus_apella .....MC.R. ......K... .......... .L....T.D. .E.FAKLV.. .....L.... .F...LQ.G. ..G....... .........- R.SG.RA..

Theropithecus_gelada ...N.MC.R. ......K... .......... .L....T.D. .E.FAKL... .....L.... .F...LQ.G. .SG....... ...V.....- R.AG.RA..

Arvicanthis_niloticus .....MC.R. ......K... L......... VL.T.FT.D. .Q.FS.L... .....L.... .F...LQ.G. .SG......S .S.G.....- R.WG.RA..

Arvicola_amphibius .....MC.R. ......K... L......... VL....T.D. LE.FSKL... .....L.... .F...LQ.G. ..G....... .F.G.....R R.CG.RA..

Castor_canadensis .....MC.R. ......K... L......... .I....T.D. .Q.HS.RV.. ....AL.... .F...LQ.G. ..G....... .F.G.....- R.SG.LAW.

Cavia_porcellus .....MC.R. ......K... L......... .L....T.D. .Q.LP.R... .....L.... .F...LQ.G. ..G......W .S.V.....- R.SG.RAW.

Cricetulus_griseus .....MC.R. ......K... L......... VL....T.D. .Q.FSKL... .....L.... .F...LQ.G. ..G....... .S.G.....- R.WG.RV..

Fukomys_damarensis .....MC.R. ......K... L......... .L....T.D. .Q.FSKL... .....L.... .F...LQ.G. ..G......W .S.A.....- RNSG.RA..

Grammomys_surdaster .....MC.R. ......K... L......... VL.T.FT.D. .Q.FS.L... .....L.... .F...L..G. GSG......S .S.G.....- R.WG.RA..

Heterocephalus_glaber .....MC.R. ......K... L......... .L....T.D. .Q.FSKL... .....L.... .F...LQ.G. ..G......W .S.A...PV- Q.SG.RAW.

Ictidomys_tridecemlineatus .....MC.R. ......K... .......... .L....T.D. IQ.FSKL... .V...L.... .F...LQ.G. V.G....... .F.A....D- N.S..GAW.

Jaculus_jaculus .....MC.R. ......K... .......... .I....T.D. .Q.FS.L... .....L.... .F...LQ.G. ..G....... .S.G.....- R.WG.RA..

Marmota_flaviventris .....MC.R. ......K... .......... .L....T.D. IQ.FSKL... .V...L.... .F...LQ.G. V.G....... .F.A....D- N.SG.GAW.

Marmota_marmota .....MC.R. ......K... .......... .L....T.D. IQ.FSKL... .V...L.... .F...LQ.G. V.G....... .F.A....D- N.SG.GAW.

Mastomys_coucha .....MC.K. ......K... L......... VL.T.VT.D. .Q.FS..... .....L.... .F...LQ.G. .SG......S .S.G.....- R.WG.RA..

Meriones_unguiculatus .....MC.R. ......K... L......... .L...VT.D. .Q.FSKL... .....L.... .F...LQ.G. ..G....... .S.G.....- R.WG.RA..

Mesocricetus_auratus .....MC.R. ......K... L......... VL....T.D. .Q.FSKL... .....L.... .F...LQ.G. ..G....... .S.G.....- R.WG.RA..

Microtus_ochrogaster .....MC.R. ......K... L......... VL....T.D. LE.FSKL... .....L.... .F...LQ.G. ..G....... .F.G.....R R.CG.RA..

Mus_caroli FR...MC.R. ......K... L..L.M.... FL.T.FT.D. .Q.FS.L... .....L.... .F...LQ.G. .SR....... .T.G.....- R.WG.RA..

Mus_musculus FR...MC.R. ......K... L..L.M.... FL.T.FT.D. .Q.FS.L... .....L.... .F...LQ.G. .SR....... .T.G.....- R.WG.RA..

Mus_pahari .R...MC.R. ......K... L..L...... VL.T.FT.D. .E.FSKL... .....L.... .F...LQ.G. .SR......S .A.D.....- R.WG.RA..

Mus_spicilegus FR...MC.R. ......K... L..L.M.... FL.T.FT.D. .Q.FS.L... .....L.... .F...LQ.G. .SR....... .T.G.....- R.WG.RA..

Nannospalax_galili .....MC.R. ......K... .......... .I....T.D. .Q.YSKL... .....L.... .F...LQ... ..G....... .S.G.....- R.WG.RA..

Octodon_degus .....MC.R. ......K... L......... .L....T.D. .Q.FSKL... .....L.... .F...LQ.G. ..G....... .S.V....P- RCSG.RA..

Onychomys_torridus .....MC.R. ......K... L......... VL....T.D. .Q.FSKL... .....L.... .F...LQ.G. ..G....... .S.G.....- ..WG.RA..

Peromyscus_maniculatus .....MC.R. ......K... L......... VL....T.D. .Q.FSKL... .....L.... .F...LQ.G. ..G....... .S.G.....- R.WR.RA..

Rattus_norvegicus .....MC.R. ......K... L......... VL.T.FP.D. .Q.FSKR... .....L..V. .F...LQ.G. ..G....... .S.G.....- R.WG.RT..

Rattus_rattus .....MC.R. ......K... L......... VL.T.FP.D. .Q.FSKR... .....L..V. .F...LQ.G. ..G....... .S.G.....- R.WG.RA..

Sciurus_vulgaris .....MC.R. ......K... .......... .L....T.D. IQ.FSKL... .....L.... .F...LQ.G. V.G....... .F.V.....- R.SG.GAW.

Urocitellus_parryii .....MC.R. ......K... .......... .L....T.D. IQ.FSKL... .V...L.... .F...LQ.G. V.G....... .F.A....D- N.SG.GAW.

Monodelphis_domestica LEKNIKYFQK HSKNQEIQHF GANQGPGEYR -----PDSG- --GQLPHLAG RDQRTFSSQP HRGKVALL-- RG-SLSESSE GDFTIIDTTE EILEDSEI

Phascolarctos_cinereus ..R.F...G. ...S.AS... RW.R...... -----..IS- --........ ...G...... .Q.....V-- ..-....... .......... ....E...

Sarcophilus_harrisii ..R.F..... ...S.AS... .W......D. -----.G.N- --...S.... ...G...... ........-- G.-....... .......... ....G...

Trichosurus_vulpecula ..R.F...G. ...S.AS... .W.P...... -----...S- --........ .......... .......V-- ..-....... .......... ....E...

Vombatus_ursinus ..R.F...G. ...S.AS.N. RW........ -----...S- --....Q... ...G...... .A.....V-- ..-....I.. .......... ....E...

Choloepus_didactylus ...-FRLLG. YP.KLSEEG- ----SS.TLQ RLQSSSG.-- --.S..Y.SS QG.GELGAR. Y..HA.WP-- ..S....... ....LAN.M. ....E...

Dasypus_novemcinctus ...SFGLLG. YP.KLSDGN- ----SSRTLQ KLQPS.G.-- --R.FLYQ.T WGPGGLGTW. ...HA.WP-- ..S......D ....LAN.M. ....E...

Chrysochloris_asiatica .G..FRFLG. WP.KLSLGD- ----.A.TLQ KLQLSHS..- --E.FL.VTE .GL.ELDTRS ...HT.WP-- ..S....... ....QAN.M. ....E...

Elephantulus_edwardii .G..FRFLG. CP..FPEDN- ----STRMMQ KLQLS.G..- --.K.L...A WGLGKVGTR. .GNRAPWP-- ..SN.....D ....PAN.V. ....E...

Loxodonta_africana .G..FRFLG. CP.KLSEED- ----SA.TLQ KLQLS.G..- --...L..TM .GLGELGTP. ...HT.WP-- ..S....... ....LAN.L. ....E...

Orycteropus_afer ....FRFLG. CP.KLSEED- ----.A.TLQ KLQLS.G..- --...L...T Q--SELGTR. ...NT.WP-- ..T....... ....LAN.L. ....E...

Trichechus_manatus .G..VRFLG. FP.KLSEGD- ----.A.TLQ KLQFS.GG.- --...L...M WGLGELGAP. ...HA.WP-- ..S....... ....LAN.L. ....E...

Balaenoptera_acutorostrata .G..FRFLE. CP.KLAEGT- ----.S.KLQ KARPS.HG-- --.R.L.STA EGLGAAAPL. RQ.HA.WP-- ..S.V..... ....LVH.M. ....E...

Balaenoptera_musculus .G..FRFLE. CP.KLAEGT- ----.S.KLQ KARPS.HG-- --.R.L.STA EGLGAAGAL. RQ.HA.WP-- ..S.V..... ....LVH.M. ....E...

Delphinapterus_leucas .G..FRFLE. CP.KLSEGT- ----.S.KLQ KARPS.HG-- --...L.STE EGL.VAGAL. RQ.HA.WP-- ..S.V..... ....LVH.M. ....E...

Globicephala_melas .G..FRFLE. CP.KLSEGT- ----.SSKLQ KARPS.HG-- --...L.STE EGL.VAGTL. RQ.HA.WP-- ..S.V..... ....LVH.V. ....E...

Lagenorhynchus_obliquidens .G..FRFLE. CP.KLSEGT- ----SSSKLQ KARPS.HG-- --...L.STE EGL.VAGAL. RQ.HA.WP-- ..S.V..... ....LVH.V. ....E...

Lipotes_vexillifer .G..FRFLE. CP.KLSEGT- ----.SSKLQ KARPS.HG-- --...L..TE EVLGVAGAL. RQ.HA.WP-- ..S.V..... ....LVH.M. ....E...

Monodon_monoceros .G..FRFLE. CP.KLSEGT- ----.S.KLQ KARPS.HG-- --...L.STE EGL.VAGAL. RQ.HA.WP-- ..S.V..... ....LVH.M. ....E...

Neophocaena_asiaeorientalis .G..FRFLE. CP.KLSEGT- ----.S.KLQ KARPS.HG-- --...L.STE EGL.VAGAL. RQ.HA.WP-- ..S.V..... ....LVH.M. ....E..M

Orcinus_orca .G..FRFLE. CP.KLSEGT- ----SSSKLQ KARPS.HG-- --...L.STE EGL.VAGAL. RQ.HA.WP-- ..S.V..... ....LVH.V. ....E...

Phocoena_sinus .G..FRFLE. CP.KLSEGT- ----.S.KLQ KARPS.HG-- --...L.STE EGL.VAGAL. RQ.HA.WP-- ..S.V..... ....LVH.M. ....E..M

Tursiops_truncatus .G..FRFLE. CP.KLSEGT- ----.SSKLQ KARPS.HG-- --...L.STE EGL.VAGAL. RQ.HA.WP-- ..S.V..... ....LVH.V. ....E...

Bison_bison .G..FRFLG. CP.KLSEGT- ----NS.SLQ KARPW.GG-- --.R.L..TK EGLGVAGTP. .Q.HA.WP-- ..S....... .Y..LVH.M. ..FKE...

Bos_mutus .G..FRFLG. CP.KLSEGT- ----NS.SLQ KARPW.GG-- --.R.L..TK EGLGVAGTP. .Q.HA.WP-- ..S....... .Y..LVH.M. ..FKE...

Bos_taurus .G..FRFLG. CP.KLSEGT- ----NS.SLQ KARPW.GG-- --.R.L..TK EGLGVAGTP. RQ.HA.WP-- ..S....... .Y..LVH.M. ..FKE...

Bubalus_bubalis .G..FRFLG. CP.KLSEGT- ----NS.SLQ KARPW.GG-- --.R.L..TR EGLGVAGTP. RQ.HA.WP-- ..S....... .Y..LVH.M. ..FKE...

Camelus_dromedarius ....FRFLG. CP.KLSEGT- ----SA.TLG KVQPSSG.-- --R..L..TV EGLGEVGAP. R..HA.WP-- ..S....... ....LAH.M. ....E...

Camelus_ferus ....FRFLG. CP.KLSEGT- ----.A.TLG KVQPSSG.-- --R..L..TV EGLGEVGAP. R..HA.WP-- ..S....... ....LAH.M. ....E...

Capra_hircus .G..FRFLG. CP.KLSEGT- ----NS.SLQ KARPL.GG-- --.R.L.STR ESLGVAGTP. RQ.HA.WP-- ..S....... ....LVH.M. ..F.E...

Catagonus_wagneri ..R.FRLLGR CP.KFSEGT- ----.T.TLQ KALPSSGQ-- --.RALR.TA EGPGEAGAP. R..HP.WP-- ..S....... ....LVH.M. ....E...

Cervus_hanglu .G..FRFLG. CP.KLSEGT- ----NS.SLQ KAQPS.GG-- --.R.L..TR EGLGVVGAL. RQ.H..WP-- ..S....... ....LVH.M. ..F.E...

Moschus_moschiferus .G..FRFLG. CP.KLSEGI- ----DS.SLQ KARPS.GG-- --.RFL...R EGLGVAGTP. .Q.HA.WP-- ..S....... ....LVH.M. ..F.E...

Sus_scrofa ....FRFLG. CP.KVPEGT- ----.T.TLQ KALPSSGR-- --..SL..TA DGLGQAEAL. RH.HP.WP-- ..S....... ....LVH.M. ....E...

Vicugna_pacos ....FRFLG. CP.KLSEGT- ----.A.TLG KAQPSSGG-- --R..L..TV EGLGELGAP. R.CHA.WP-- ..S....... ....LAH.M. ....E...

Ailuropoda_melanoleuca ....FRFLG. CP.KLSQAD- ----.A.TLQ KLQPAAGG-- -DAP.L..SE HGLGGRGARS P..HA.WPRG ..SIV..... .E..LTH.M. ....E...

Callorhinus_ursinus ....FRF.G. YP.KLSQAD- ----.T.TLQ KLQTSCGG-- -N...L.MSV QGLGGRGAG. P..HK.WP-- ..S.V..... ....LTH.M. ....E.K.

Canis_lupus .Q.DFQFLG. CP.KLSQAG- ----RTRTRE KLQPSLGG.N NH...L...M HGLGGLGPR. P..HA.WP-- ..S......D ....LTH.M. ....E...

Enhydra_lutris ....FQFLG. CP.KLSQAD- ----.T.TLQ KLQTSSGG-- -D...L..SV HVLGGRGARA P..HP.WP-- ..S.V..... ....LTH.M. ....E...

Eumetopias_jubatus ....FRF.G. YP.KLSQAD- ----.T.TLQ KLQTSCGG-- -N...L.MSV QGLGGRGAG. P..HT.WP-- ..S.V..... ....LTH.M. ....E.K.

Felis_catus ....FRFLG. CP.KLSQAD- ----.K.TLE KPQPS.GG-- --...L...M QGLGGLGAR. P..HG.WP-- ..S....... ....LTH.M. ....E...

Lynx_canadensis ....FRFLG. CP.KLSQAD- ----.K.TLQ KPQAS.GG-- --...L...M QGLGGLGAR. P..HG.WP-- ..S....... ....LTH.M. ....E...

Mirounga_leonina ....FRFLG. CP.KLSQAD- ----.T.TLQ KLQPSSGG-- -N...L..SV HGLGGQGAR. P..HA.WP-- Q.S.V..... ....LTH.M. ....E...

Mustela_erminea ....FRFLG. CP.KLSQAD- ----.T.TLQ KLQTSSSG-- -D...L..SV HGLGGRDARA P..HA.WP-- ..S.V..... ....LTH.M. ....E...

Mustela_putorius ....FRFLG. CP.KLSQAD- ----.T.TLQ KLQTSSGG-- -D...L..SV HGLGGRDARA P..HA.WP-- ..S.V..... ....LTH.M. ....E...

Neomonachus_schauinslandi ....FRFLG. CP.KLSQAD- ----.T.TLQ KLQPSSGG-- -N...L..SV HGLGGQGAR. P..HA.WP-- ..S.V..... ....LTH.M. ....E...

Odobenus_rosmarus .Q..FRF.G. CP.KLSQAD- ----.T.TLQ KLQPSSGG-- -N...L.MSV QGLGGRGAG. Q..HA.WP-- ..S.V..... ....LTH.M. ....E...

Panthera_pardus ....FRFLG. CP.KLSQAD- ----.K.TLQ KPQPS.GG-- --...L...M QGLGGLGAR. P..HG.WP-- ..S....... ....LTH.M. ....E...

Phoca_vitulina ....FRFLG. CP.KLSQAD- ----.T.TLQ KLQPSSGG-- -N...L..SV HGLGGRGAR. P..HA.WP-- ..S.V..... ....LTH.M. ....E...

Suricata_suricatta ....FRFLG. CP.TLARAD- ----.KRTLQ KLQ--.GG-- --...LP..A QGLGGRGAR. P..HG.WP-- ..G......D ....LTH.M. ....E...

Ursus_thibetanus ....FRFLG. CP.KLSQAD- ----.A.TLQ KLQPVAGG-- -DAP.L...E HGLGGRGAR. P..HA.WPRG ..SIV..... ....LTH.M. ....E...

Vulpes_vulpes .Q.DFRFLG. CP.KLSQAG- ----HTRTRE KLQPSLGG.N NH...L...M HGLGGLGPR. P..HA.WP-- ..S......D ....LTH.M. ....E...

Zalophus_californianus ....FRF.G. YP.KLSQAD- ----.T.TLQ KLQTSCGG-- -N...L.MSV QGLGGRGAG. P..HT.WP-- ..S.V..... ....LTH.M. ....E.K.

Eptesicus_fuscus ....FRFLG. GP.KLSEGD- ----SARALQ KSQSL.G.-- --..PLP.TM QGLGELGTRS .G.HT.WR-- WAR....... ....QAH.M. ....E..M

Miniopterus_natalensis .Q..FRFLG. CP.KLSQGG- ----SARALQ KPLSS.G.-- --...LQ.TV QG.GEQGARS P..HT.WR-- W.G....... ..L.LAH.M. ....E..M

Myotis_lucifugus ....FRFLG. YP.KLSKGD- ----SARALQ KSQSS.G.-- --...LP.TM QGLGEPGTRS .G.HT.WR-- W.R....... ....QAH.M. ....E..M

Myotis_myotis ....FRFLG. YP.KLSKGD- ----SARALQ KSQSS.GG-- --...LP.TT QGLGEPGTRS .G.HT.WR-- W.R....... ....QAH.M. ....E..M

Pipistrellus_kuhlii M...FRFLG. CP.KLSKGD- ----SARALQ KSQSS.G.-- --..PLP.TM QGLGELDTRS .G.HT.WR-- W.R....... ....QAH.M. ....E..M

Pteropus_alecto ....FRFLG. CP.RLSKRD- ----NARMLQ KPQPS.G.-- --...LQ.TV QGPGQPGAR. .Q.HT.WP-- WVS....... ....LAH.M. .S..E...

Rhinolophus_ferrumequinum .....RFLG. CP.KLSKGN- ----STRTLQ KPRPS.G.-- --..ILQ.SV QSLGALGAR. .L.HT.WP-- W.S....... ..L.LAH.M. ....E...

Sturnira_hondurensis ....FRFLRN FP.KFSKGD- ----SARALQ RPRCL.G.-- --...LQ.TA QGPGEPGTRS Q..HA.WS-- Y.R....... ...SLAH.M. ....E..T

Condylura_cristat .-.TFRFLG. CP.KLSEED- ----STRKLQ KLRPSQGR-- --E..RN.SM QGLGQRGPR. -------P-- ..S....... .Y..MAH... ....E...

Sorex_araneus ....FRFLG. YPNKFSEGD- ---------H SLQPL.GR-- --..IL..PL QSPGARGPRT -------P-- ..S.V..... ..I.LTH.M. ....E..M

Talpa_occidentalis .-..FRFLG. CPQKLSEED- ----STRTRP KLRPLAR.-- --E..L..TT QGPGQRGP.. -------P-- ..S....... .YV.MAH... ....E...

Ceratotherium_simum ....FRFLG. GP.KLSEGD- ----SA.TLQ KLRPS.G.-- --...L...V QGLGVLGGG. ...HAGWP-- ..S....... ....PAH.M. ....E...

Equus_caballus ....FRFLG. CP.KLSEGD- ----SA.TL. KLRPS.G.-- --...L...M QGLGELGGR. Q..HAGWP-- ..S....... ....LAH.M. ....E...

Manis_javanica ....FQFLG. CP.KLSKGD- ----RARAL. RLRPP.G.-- --...L..T. QGLEELGARL .G.HADWP-- ..S....... ....LAH.M. ....E...

Manis_pentadactyla ....FQFLG. CP.KLSKGD- ----RARAL. RLRPP.G.-- --...L..T. QGLEELGARL .GCHADWP-- ..S....... ....LAH.M. ....E...

Prolemur_simus PG..LRFLG. SP.KLSEGD- ----.AEKL. KLQPSLN.-- --...L...M .GLGELGAR. .Q.HAPWP-- .AS....Y.. ..V.LAH.M. ....E...

Tupaia_chinensis .G..FRFLG. YP.KLSGRD- ----.AEKLG KRQPSLSN-- --..FL.Q.M .GLGELGTR. ...QT.WP-- ..G....C.. ....LAN.M. ....E...

Aotus_nancymaae .G..FRFLG. CP.KLSEGD- ----.AEKL. QLQPSLN.-- --.R.L...M .GLGELGTR. QQDHARWP-- ..S....Y.. ..V.LAN.M. ....E...

Callithrix_jacchus .G..FRLLG. CP.KLSEGD- ----.TEKL. QLQPSLNR-- --.R.I...M .GLGELGTR. QQDHARWR-- Q.S....Y.. ..V.LAN.M. ....E...

Carlito_syrichta .G..FRFLG. CP.KFSEGD- ----.AEKL. KLQPSLN.-- --E..LR..M .GLGELGTR. .Q.HAPWP-- ..S....C.. ..V.LAN.M. ....E...

Cebus_capucinus .G..FRFLG. GP.KLSEGD- ----.AEKL. QLQPSLN.-- --.R.L...M .GLGELGTR. QQDHARWP-- ..S....Y.. ..V.LAN.V. ....E...

Cercocebus_atys .G..FRFLG. CP.KLSEGD- ----.AEKL. TLQPSLN.-- --.RFL...M .GLGELG.R. QQDHARWP-- ..S....C.. ..V.MAN.M. ....E...

Chlorocebus_sabaeus .G..FRFLG. CP.KLSEGD- ----.AEKL. TLQPSLN.-- --.RFL...M .GLGELGAR. QQDHARWP-- ..S....C.. ..V.MAN.M. ....E...

Colobus_angolensis .G..FRFLG. CP.KLSEGD- ----.AEKL. TLQPSLN.-- --.RFL...M .GLGELGAR. QQDH.RWP-- ..S....C.. ..V.MAN.M. ....E...

Gorilla_gorilla .G..FRFLG. CP.KLSEGD- ----.TEKL. KLQPSLN.-- --.R.L...M .GLGELGA.. QQDHARWP-- ..S....C.. ..V.MAN.M. ....E...

Homo_sapiens .G.DFRFLG. CP.KLSEGD- ----.AEKL. KLQPSLN.-- --.R.L...M .GLGELGA.. QQDHARWP-- ..S....C.. ..V.MAN.M. ....E...

Hylobates_moloch .G..FRFLG. CP.KLSEGD- ----.AEKL. KLQPSLN.-- --.R.L...M .GLGELGA.. QQDHARWP-- ..S....C.. ..V.MAN.M. ....E...

Macaca_fascicularis .G..FRFLG. CP.KLSEGD- ----.AEKL. TLQPSLN.-- --.RFL...M .GLGELG.R. QQDHARWP-- ..S....C.. ..V.MAN.M. ....E...

Macaca_mulatta .G..FRFLG. CP.KLSEGD- ----.AEKL. TLQPSLN.-- --.RFL...M .GLGELG.R. QQDHARWP-- ..S....C.. ..V.MAN.M. ....E...

Macaca_nemestrina .G..FRFLG. CP.KLSEGD- ----.AEKL. TLQPSLN.-- --.RFL...M .GLGELG.R. QQDHARWP-- ..S....C.. ..V.MAN.M. ....E...

Mandrillus_leucophaeus .G..FRFLG. CP.KLSEGD- ----.AEKL. TLQPSLN.-- --.RFL...M .GLGELG.R. QQDHARWP-- ..S....C.. ..V.MAN.M. ....E...

Microcebus_murinus PGR.LRFLG. CP.KLSEGD- ----.AEKLG KLQPSLNR-- --...L...M .GLGELGAR. .Q.HA.WP-- ..G....Y.. ..V.LAN.M. ....E...

Nomascus_leucogenys .G..FRFLG. CP.KLSEGD- ----.TEKL. KLQPSLN.-- --.R.L...M .GLGELGA.. QQDHARWP-- ..S....C.. ..V.MAN.M. ....E...

Otolemur_garnettii SG..FRIPS. CP.KISEGD- ----SAEKLQ KLQPSLN.-- --...L...M .GLGELGAR. .Q.RTPWP-- ..S....C.. ..V.LAN.M. ....E...

Pan_paniscus .G..FRFLG. CP.KLSEGD- ----.AEKL. KLQPSLN.-- --.R.L...M .GLGELGT.. QQDHARWP-- ..S....C.. ..V.MAN.M. ....E...

Pan_troglodytes .G..FRFLG. CP.KLSEGD- ----.TEKL. KLQPSLN.-- --.R.L...M .GLGELGA.. QQDHARWP-- ..S....C.. ..V.MAN.M. ....E...

Piliocolobus_tephrosceles .G..FRFLG. CP.KLSEGD- ----.AEKL. TLQPSLN.-- --.GFL...M .GLGELGTR. QQDHARWP-- ..S....C.. ..V.MVN.M. ....E...

Pongo_abelii .G..FRFLG. CP.KLSEGD- ----.AEKL. KLQPSLN.-- --.R.L...M .GLGELGA.. QQDHARWP-- ..S....C.. ..V.MAN.M. ....E...

Propithecus_coquereli PG..LRFLG. CP.KLSEGD- ----.AEKL. KLQPSLN.-- --...L...I .GLGDLGAR. .Q.HAPWP-- ..S....Y.. ..V.LAN.M. ....E...

Rhinopithecus_bieti .G..FRFLG. CP.KISEGD- ----.AEKL. TLQPSLN.-- --.RFL...M .GLGELGAR. QQDHARWP-- ..S....C.D ..V.MAN.M. ....E...

Rhinopithecus_roxellana .G..FRLLG. CP.KISEGD- ----.AEKL. TLQPSLN.-- --.RFL...M .GLGELGAR. QQDHARWP-- ..S....C.. ..V.MAN.M. ....E...

Saimiri_boliviensis .G..FRFLG. CPTKLSEGD- ----.AEKL. QLQPSLN.-- --.R.L...M .GLGELGTR. QQDHARWP-- ..S....Y.. ..V.LAN.M. ....E...

Sapajus_apella .G..FRFLG. GP.KLSEGD- ----.AEKL. QLQPSLN.-- --.R.L...M .GLGELGTR. QQDHARWP-- ..S....Y.. ..V.LAN.V. ....E...

Theropithecus_gelada .G..FRFLG. CP.KLSEGD- ----.AEKL. TLQPSLN.-- --.RFL...M .GLGELG.R. QQDHARWP-- ..S....C.. ..V.MAN.M. ....E...

Arvicanthis_niloticus .G..FRFLG. C..KLSERN- ----DAETLQ KLQSSAC.-- --S---..TT GTLGDQRA.L ...RG.WP-- P.S......D ....LAN.V. ....E...

Arvicola_amphibius .G..FRFLGQ CP.KLSEGD- ----SAETL. KLQSSTCR-- --S---...S ETLGELGAR. MP.RR.WP-- ..S....... ....LAN.M. ....E...

Castor_canadensis .G..FQFLG. CP.KLSEGD- ----.AETLQ KLQSSS..-- --S---N.ST .SLGELGT.. N..HA.WP-- ..S....... ....LAN.M. ....E...

Cavia_porcellus .RR.FRFLG. CP.KLSEGD- ----.TQTIH KPQASS..-- --S---..TS QALAGLNTR. .Q.HRVWP-- ..I....... ....LAN... ....E...

Cricetulus_griseus .G..FRFLG. C..KLSEGD- ----.AETL. KLQSSTC.-- --S---...S ETLGELGAR. .Q.RG.WP-- ..S....... ....LAN.M. ....E...

Fukomys_damarensis .R..FGLLG. CP.KFSERD- ----.IQII. KPQASV..-- --S---R..L QGL.ELDTW. .QDHR.WP-- ..I...D... ....LAN.M. ....E...

Grammomys_surdaster .G..FRFLG. C..KLSERD- ----.AETLQ KLQSSAC.-- --S---...T GTLGDHRA.L ...RG.WP-- P.S......D ....LAN.V. ....E...

Heterocephalus_glaber .R..FGFLG. CP.KFSERD- ----.TQRIC KPQASA..-- --S---...S QGMGELDTR. .Q.HA.WP-- ..I....... ....LAN.M. ....E...

Ictidomys_tridecemlineatus .G..FRFLG. CP..LSDGD- ----.AETLQ KLKTS.GG-- --S---...T .GRGELGAH. ...HT.WP-- ..S....... ....LAN.M. ....E...

Jaculus_jaculus .G.TFQFLGT CP.KLTEGD- ----.AETH. QLQSS..G-- --S---Q..T GA.GQVGAR. ...HGVWP-- ..S....... ....LAN.M. ....E...

Marmota_flaviventris .G..FRFLG. CP..LSDGD- ----.AETL. KLKTS...-- --S---...T .GRGELGAH. ...HT.WP-- ..S....... ....LAN.M. ....E...

Marmota_marmota .G..FRFLG. CP..LSDGD- ----.AETL. KLKTS..G-- --S---...T QGRGELGAH. ...HT.WP-- ..S....... ....LAN.M. ....E...

Mastomys_coucha .G..FRFLG. C..KLSEGD- ----.AETLQ KLQSSKC.-- --S---RS.T GTLGDHGA.. ...RG.WP-- ..S....... ....LAN.M. ....E...

Meriones_unguiculatus .G..FRFLG. C..KLSEGDG ---A.AETL. KLRSS.C.-- --S---RS.S GTLGDRGA.. ...HGTWP-- ..I......D ....LAN.V. ....E...

Mesocricetus_auratus .G..FWFLG. C..KLSEGD- ----.AETL. KLQSSTC.-- --S---...S ETLGELGAR. Y..RG.WP-- ..S....... ....LAN.M. ....E...

Microtus_ochrogaster .G..FRFLGQ CP.KLSEGD- ----SAEML. KLQSSTC.-- --S---...S ETLGELGAR. VP.RR.WP-- ..S....... ....LAN.M. ....E..M

Mus_caroli .G..FRFLG. C..KLSEGD- ----.AETLQ KLQSSAFG-- --S---..TA GTL.DHGA.. ...RG.WP-- .AS....... ....LAN.M. ....E...

Mus_musculus .G..FRFLG. C..KLSEGD- ----.AETLQ KLQSSGV.-- --S---..TA GNL.DHGA.. ...RG.WP-- .AS....... ....LAN.M. ....E...

Mus_pahari .G..FQFLG. C..KLSEGD- ----.AETHQ KLQSSTC.-- --S---..TT GTL.DHGA.. .K.RG.WP-- .AS....... ...NLAN.M. ....E...

Mus_spicilegus .G..FRFLG. C..KLSEGD- ----.AETLQ KLQSSAF.-- --S---..TA GNL.DHGA.. ...HG.WP-- .AS....... ....LAN.M. ....E...

Nannospalax_galili .G..FRFLG. CP.KLSERE- ----.AETLQ KLQTSSG.-- --S---N.ES EAL.ELGAR. ...HG.WP-- ..S......D ....LAN.M. .V..E...

Octodon_degus .R..FRFLG. CP.KLSDGG- ----.TRTTH KLQVS.G.-- --S---..SS QGLGELDTR. CQ.HT.WP-- .SI....... ....LAN.M. ....E...

Onychomys_torridus .G..FPFLG. C..KLSEGN- ----.AETL. KLQSSTC.-- --S---...S ETLEERGARS ...RG.WP-- ..S....... ....LAN.M. ....E...

Peromyscus_maniculatus .G..FRFLG. C..KLSEGD- ----.AETL. KLQSSAC.-- --S---...S ETRGELGARS ...RG.WP-- ..S....... ....LAN.M. ....E...

Rattus_norvegicus .G..FRFLG. C..KLSEGD- ----.SETLQ KLRFSTC.-- --S---...S ETLGDVGV.. ...RG.WP-- ..S....... ....LAN.M. ....E...

Rattus_rattus .G..FRFLG. C..KLSEGD- ----.SETLQ KLRSSTC.-- --S---...S ETLGDVGV.. ...HG.WP-- ..S....... ....LAN.M. ....E...

Sciurus_vulgaris .G..FRLLG. CP.KLSDGD- ----.AETLQ KLQSS..G-- --C---...T .GLGELGAH. ..NHT.WP-- ..S....... ....LAN.M. ....E...

Urocitellus_parryii .G..FRFLG. CP..LSDGD- ----.GETL. KLKTS.GG-- --S---...T .GRGELGAH. ...HT.WP-- ..S....... ....LAN.M. ....E...

**Supplementary Figure 8. Alignment of mammalian glucagon-like peptide-2 (GLP-2) receptor (Glp2r) protein sequences**.

Predicted glucagon-like peptide-2 receptor (Glp2r) amino acid sequences from 125 mammals are based on the MAFFT [51] aligned coding sequences. Sequences are shown in single letter amino acid code, with identical residues indicated by a period (.) and gaps by dashes (-). The positions of the signal peptide and transmembrane domains (TM1 – TM7) in the human (*Homo sapiens*) sequence are indicated above the sequences, with <<< and >>> indications the extent of these domains. Amino acid sites involved in peptide ligand binding and G-protein binding are indicate by “P” and “G”, respectively, above the sequences and are from the GPCRdb [63,64]. The anchor points for the Wootten numbering system [73] are indicated by $ with the corresponding numbers shown above.
