# Supplementary material for: Variation in the Evolution and Sequences of Proglucagon and the Receptors for Proglucagon-Derived Peptides in Mammals
Source: Front Endocrinol (Lausanne). 2021 Jul 12;12:700066. doi: 10.3389/fendo.2021.700066 (PMC8312260; doi:10.3389/fendo.2021.700066)
Supplement: Supplementary File 1 — Fasta formatted proglucagon (Gcg) coding sequences. [file DataSheet_1.zip › Supplement/Suplementary Figures/Fig S6 Gcgr alignment.docx]

Signal peptide < <<

PP P PP P PP

Monodelphis_domestica --MRLTHPCY -S-H----VL L--L-----L -LWISCQLQA PFAQVMDFLY EKWKSYSDEC RYNLSLLPP- P--TELVCNR TFDKYSCWPD TLPNTTANI

Phascolarctos_cinereus --.L..---- ---.----.S .--.-----. -.VV...... .S.......F .......... H........- .--....... .......... .........

Sarcophilus_harrisii --.L..---- ---.----.. .--.-----. -.L....... AS.......F .......... H........- .--....... .......... .........

Vombatus_ursinus --.L..---- ---.----.S .--.-----. -.VV....K. .S.......F .......... H........- .--....... .......... .........

Echinops_telfairi --.PP.Q.RC -P-.---LL. .--.-----. -.LLVH.P.. .A.......F ....L.G... SH.......- .--....... .......... .......S.

Elephantulus_edwardii --.PT.Q.RC -.-.-----. .--.-----. -.LLAR.P.V .A.......F .T..I.G.Q. TH.......- .--....... .....A.... .PV.....L

Orycteropus_afer --.PPAQ..C -P-.-----. .--.-----. -.LLAR.P.. .A.......F ....L.G.Q. .H.......- .--....... .......... .P.......

Trichechus_manatus --.PI.Q.RH -P-.----L. .--.-----. -.LLAR.P.. .A.......F ....L.G.Q. H........- .--....... .......... .........

Balaenoptera_acutorostrata --.PPIL.HC -P-.-----. .--.-----. -.LLA..P.. .S.......F ....L.G.Q. LH.......- .--....... .......C.A PP.......

Balaenoptera_musculus --.PPIL.RC -P-.-----. .--.-----. -.LLA..P.. .S.......F ....L.G.Q. LH.......- .--....... .......... .P.......

Delphinapterus_leucas --.PPVL.RC -P-.-----. .--.-----. -.LLA..P.. .S.......F ....L.G.Q. LH.......- .--....... .......... .P.......

Globicephala_melas --.PPIL.RC -P-.-----. .--.-----. -.LLA..P.. .S.......F ....L.G.Q. L........- .--....... .......... .P.......

Lagenorhynchus_obliquidens --.PPIL.RC -P-.-----. .--.-----. -.LLA..P.. .S.......F ....L.G.Q. LH.......- .--....... .......... .P.......

Lipotes_vexillifer --.PPIL.RC -P-.-----. .--.-----. -.LLA..P.. .S.......F ....L.G.Q. LH.......- .--....... .......... .P.......

Monodon_monoceros --.PPVL.RC -P-.-----. .--.-----. -.LLA..P.. .S.......F ....L.G.Q. LH.......- .--....... .......... .P.......

Neophocaena_asiaeorientalis --.PPIL.RC -P-.-----. .--.-----. -.LLA..P.. .S.......F ....L.G.Q. LH.......- .--....... .......... .P.......

Orcinus_orca --.PPIL.RC -P-.-----. .--.-----. -.LLA..P.. .S.......F ....L.G.Q. LH.......- .--....... .......... .P.......

Phocoena_sinus --.PPIL.HC -P-.-----. .--.-----. -.LLA..P.. .S.......F ....L.G.Q. LH.......- .--....... .......... .P.......

Physeter_catodon --.PPIL.RF -P-.-----. .--.-----. -.LLA..P.. .S.......F ....L.G.Q. LH.......- .--....... .......... .P.......

Bos_indicus --.PPIQ.H. -P-.-----. F--.-----. -.LLA..PET .S.......F ....L.G.Q. LH......L- .--....... .......... .P.......

Bos_mutus --.PPIQ.H. -P-.-----. F--.-----. -.LLA..PET .S.......F ....L.G.Q. LH......L- .--....... .....F.... .P.......

Bos_taurus --.PPIQ.H. -P-.-----. F--.-----. -.LLA..PET .S.......F ....L.G.Q. LH......L- .--....... .......... .P.......

Bubalus_bubalis --.PPIQ.H. -P-.-----. F--.-----. -.LLA..PET .S.......F ....L.G.Q. LH......L- .--....... .......... .P.......

Camelus_dromedarius --.PRLQ.RC -P-.-----. .--.-----. -.LLAW.P.. .S.......F ....L.G.Q. L........- .--....... .......... .P.......

Camelus_ferus --.PRLQ.RC -P-.-----. .--.-----. -.LLAW.P.. .S.......F ....L.G.Q. L........- .--....... .......... .P.......

Capra_hircus --.PPIQ.R. -P-.-----. SL-.-----. -.LLA..PET .S.......F ....L.G.Q. LH.......- .--....... .......... AP.......

Cervus_hanglu --.PPIQ... -P-.-----. F--.-----. -.LLA..P.T .S.......F ....L.G.Q. LH.......- .--....... .......... .P.......

Moschus_moschiferus --.PPIQ.R. -P-.-----. .--.-----. -.LLA..PET TS.......F ....L.G.Q. LH.......- .--....... .......... .P.......

Odocoileus_virginianus --.PPIQ.R. -P-.-----. F--.-----. -.LLA..P.T .S.......F ....L.G.Q. LH.......- .--....... .......... .P.......

Ovis_aries --.PPIQ.R. -P-.-----. FL-.-----. -.LLA..PET .S.......F ....L.G.Q. LH.......- .--....... .......... AP.......

Sus_scrofa --.PPARLRH -P-.-----. .--.-----. -.LLA..P.. .A..A....F Q...L.G.Q. LR.......- .--....... .......... .P.......

Acinonyx_jubatus --.PP.R.HR -P-.-----F .P-.-----. -.LLA..P.. .S.......F ....L.G.Q. L........- .--....... .......... .P.......

Callorhinus_ursinus --.PP.R.HC -P-Q-----. ---.-----. -.LLA..PW. .S.......F ....L.G.Q. L........- .--....... .......... .P......M

Canis_lupus --.PPAR.RC -P-.-----. .--.------ -.LLA..P.. SS.......F ....L.G.Q. L........- .--....... .......... .P.......

Enhydra_lutris --.CPAP.HC PP-P----L. .--.-----. -.LLA..P.. .S.......F ....L.G.Q. LS.......- .--....... .....A.... .PA.....T

Eumetopias_jubatus --.PP.R.HC -P-Q-----. ---.-----. -.LLA..PW. .S.......F ....L.G.Q. L........- .--....... .......... .P......M

Felis_catus --.PP.R.HR -P-.-----F .PL.-----. -.LLA..P.. .S.......F ....L.G.Q. L........- .--....... .......... .P.......

Halichoerus_grypus --.PP.R.HC -P-Q-----. ---.-----. -.LLA..PW. .S.......F ....L.G.Q. L........- .--....... .......... .P......M

Leptonychotes_weddellii --.PP.R.HC -P-Q-----. ---.-----. -.LLA..PW. .S.......F ....L.G.Q. L........- .--....... .......... .P......M

Lontra_canadensis --.CP.P.HC -P-.----L. .--.-----. -.LLA..P.. .S.......F ....L.G.Q. LS.......- .--....... .....A.... .PA.....T

Lynx_canadensis --.PPAR.HR -P-.-----F .P-.-----. -.LLA..P.. .S.......F ....L.G.Q. L........- .--....... .......... .P.......

Mirounga_leonina --.PP.R.HC -P-Q-----. ---.-----. -.LLA..PW. .S.......F ....L.G.Q. L........- .--....... .......... AP......T

Mustela_erminea --.CPAP.PC -P-.----L. .--.-----. -.LLF..P.. .S.......F ....L.G.Q. LS.......- .--....... .....A.... .PA.....T

Neovison_vison --.CPAP.PC -P-.----L. .--.-----. -.LLF..P.. .S.......F ....L.G.Q. LS.......- .--....... .....A.... .PA.....T

Odobenus_rosmarus --.PP.R.HC -P-Q-----. ---.-----. -.LLA..PW. .S.......F ....L.G.Q. L........- .--....... .......... .P......M

Phoca_vitulina --.HP.R.HC -P-Q-----. ---.-----. -.LLA..PW. .S.......F ....L.G.Q. L........- .--....... .......... .P......M

Suricata_suricatta --.PP.W.HR -A-.-----. .--.-----P -.LLA.RP.. .A.......F ....L.G.Q. L.......A- .--....... .......... .P.......

Ursus_americanus --.PP.R.HR -P-RPRHVL. ---.-----. -.LLA.--.. .S.......F .R..L.G.Q. L........- .--....... .......... .P......T

Ursus_arctos --.PP.R.HR -P-RPRHV-. ---.-----. -.LLA..... .S.......F .R..L.G.Q. L........- .--....... .......... .P......T

Ursus_thibetanus --.PP.R.HR -P-RPCHV-. ---.-----. -.LLA..... .S.......F .R..L.G.Q. L.......AL .CL....... .......... .P......T

Zalophus_californianus --.PP.R.HC -P-Q-----. ---.-----. -.LLA..PW. .S.......F ....L.G.Q. L........- .--....... .......... .P......M

Artibeus_jamaicensis --.PPAQ..P SP-------. .--.-----. -.LLA..P.. .S.......L ....L.G.Q. LH.......- .--....... .......... .P.....R.

Desmodus_rotundus --.PP.W..H SP-------. .--.-----. -.LLA..P.. .S.......L ....L.G.Q. LH.......- .--....... .......... .P.....S.

Hipposideros_armiger --.PPSWLHR -P-.-----. .--.-----. -.LLAF.PE. .S.......F ....L.G.Q. LH.......- .--....... .......... .A.....S.

Miniopterus_natalensis --.PPAL.HR -P-.----F. .P-.-----. -.LL...P.. .S.......F ....L.G.Q. LH.......- .--....... .......... .P.......

Molossus_molossus --.PP.W.HR -P-P----F. ---.-----. -.LLAG.P.. .A...T...F ....L.G.Q. LH.......- .--....... .......... .P.....S.

Myotis_myotis --.PP.R.RP PP-------F .--.-----. -.LLA..... .C..M..SVF ....L.G.Q. LH.......- .--....... .......... .P.....S.

Phyllostomus_discolor --.TP.R.RR GP---PALL. .--.-----. -.LLA..P.. .S.....S.L .R..L.GEQ. LH.......- .--....... .......... .P.....S.

Pipistrellus_kuhlii --.PP.W.RP -P-P----F. .--.-----. -.LLTS.P.. .C.......F .R..L.G.Q. LH.......- .--....... .......... .P.......

Rousettus_aegyptiacus --.PPAQ..R -P-R----L. .--.-----. -.LLAS.P.. .S..A....F ....L.G.Q. LH.......- .--....... .......... .P.....S.

Sturnira_hondurensis --.PP.R..R SL-------. .--.-----. -.LLA..T.. .S.......L ....L.G.Q. LH.......- .--....... .......... .P.....S.

Talpa_occidentalis --.PP.QSHH -P-.-----. .--.-----. -.LLV..PL. .G..A..S.L AR.RL.R.Q. LR.......- .--....... .......... .P..S....

Ceratotherium_simum --.PP.W.RC -P-.------ .L-.-----. -.LLA..P.. .S.......F ....L.G.Q. LH.......- .--S...... .......... .P.......

Equus_caballus --.PP.W.RR -P-.-----. .L-.-----. -.LLA..P.. .S.......F ....L.G.Q. LH.......- .--....... .......... .P.......

Manis_pentadactyla --.LPAL..G -P-.-----. .--.------ -.LLA....V .A.......F ...QL.G.Q. LH.......- .--....... .......... .P.....SM

Galeopterus_variegatus --.SHAR.HC -A-V------ ---.-----. -.LLA..P.. .S.......F ....L...Q. HH.......- .--....... .......... .P.......

Callithrix_jacchus --.P.FQ.RQ -P-L-----. .--.------ -.LLV..P.. .S.......F ....L...Q. HH.......- .--....... .......... .P.......

Cebus_capucinus --.P.RQ.RP -P-V-----. .--.------ -.LLV..P.. SS.......F .R..L.G.Q. HH.......- .--....... .......... .P.......

Cercocebus_atys --.PPCQ.RR -P-L-----. .--.-----. -.LLA..P.. .S.......F ....L.G.Q. HH.......- .--....... .......... .PA......

Chlorocebus_sabaeus --.PPCQ.RR -P-L-----. .--.-----. -.LLA..P.. .S.....V.F ....L.G.Q. HH.......- .--....... .......... .PA......

Colobus_angolensis --.SPCQ.RR -P-L-----. .--.-----. -.LLA..P.. .S.......F ....L.G.Q. HH.......- .--....... .......... .PA......

Gorilla_gorilla --.PPCQ.QR -P-L-----. .--.-----. -.LLA..P.V .S.......F ....L.G.Q. HH.......- .--....... .......... .PA......

Homo_sapiens --.PPCQ.QR -P-L-----. .--.-----. -.LLA..P.V .S.......F ....L.G.Q. HH.......- .--....... .......... .PA......

Hylobates_moloch --.PPCQ.RR -P-L-----. .--.-----. -.LLA..P.. .S.......F ....L.G.Q. HH.......- .--....... .......... .PA......

Macaca_fascicularis --.PPCQ.RR -P-L-----. .--.-----. -.LLA..P.. .S.......F ....L.G.Q. HH.......- .--....... .......... .PA......

Macaca_mulatta --.PPCQ.RR -P-L-----. .--.-----. -.LLA..P.. .S.......F ....L.G.Q. HH.......- .--....... .......... .PA......

Macaca_nemestrina --.PPCQ.RR -P-L-----. .--.-----. -.LLA..P.. .S.......F ....L.G.Q. HH.......- .--....... .......... .PA......

Mandrillus_leucophaeus --.PPCQ.RR -P-L-----. .--.-----. -.LLA..P.. .S.......F ....L.G.Q. HH.......- .--....... .......... .PA......

Microcebus_murinus --.PPAR.HR -P-.-----. .--.-----. -.LLAVRP.. .S.E.....F ....L...Q. HH.......- .--....... .......... .P.......

Nomascus_leucogenys --.PPCQ.RR -P-L-----. .--.-----. -.LLA..P.. .S.......F ....L.G.Q. HH.......- .--....... .......... .PA......

Otolemur_garnettii --.SP.W.RH -R-.----L. .--.-----. -.LLAYR... TS.......F ....L...Q. YH.Q.....- .--....... .......... .P.......

Pan_paniscus --.PACQ.QR -P-L-----. .--.-----. -.LLA..P.V .S.......F ....L.G.Q. HH.......- .--....... .......... .PA......

Pan_troglodytes --.PACQ.QR -P-L-----. .--.-----. -.LLA..P.V .S.......F ....L.G.Q. HH.......- .--....... .......... .PA......

Papio_anubis --.PPCQ.RR -P-L-----. .--.-----. -.LLA..P.. .S.......F ....L.G.Q. HH.......- .--....... .......... .PA......

Piliocolobus_tephrosceles --.PHCQ.RR -P-L-----. .--.-----. -.LLA..P.. .S.......F ....L.G.Q. HH.......- .--....... .......... .PA......

Pongo_abelii --.PPCQ.RR -P-L-----. .--.-----. L.LLA..P.. .S.......F ....L.G.Q. HH.......- .--....... .......... .PA......

Propithecus_coquereli --.PPAR.HR -P-.-----. .--.-----. -.LLV.GP.. .S.......F ....L...Q. HH.......- .--....... .......... .A.......

Rhinopithecus_bieti --.PPCQ.RR -P-L-----. .--.-----. -.LLA..P.. .S.......F .N..L...Q. HH.......- .--....... .......... .PA......

Rhinopithecus_roxellana --.PPCQ.RR -P-L-----. .--.-----. -.LLA..P.. .S.......F .N..L...Q. HH.......- .--....... .......... .PA......

Saimiri_boliviensis --.P.RQ.RQ -A-V-----. .--.------ -.LLVFKP.. SS.......F ....L.G.Q. H........- .--....... .......... .P.......

Sapajus_apella --.P.RP.RP -P-V-----. .--.------ -.LLV..P.. SS.......F .R..L.G.Q. HH.......- .--....... .......... .P.......

Theropithecus_gelada --.PPCQ.RR -P-L-----. .--.-----. -.LLA..P.. .S.......F ....L.G.Q. HH.......- .--....... .......... .PA......

Trachypithecus_francoisi --.PPYQ.RR -P-L-----. .--.-----. -.LLA..P.. .S.......F ....L.G.Q. HH.......- .--....... .......... .PA......

Ochotona_princeps MWVAAAQRRA ---R-----. .A-.-----. -.LLAS.P.V .S.......F ...RL.GEQ. QQ.......- .--.Q..... .......... .......S.

Arvicanthis_niloticus --.P..QFH. -P-.----L. .--.-----. -VVL..LPK. .S.......F ....L...Q. HH...R...- .--....... .......... .P.......

Arvicola_amphibius --.PR.LLHS -P-.------ .--.-----. -.LL..LPK. .S....N..F ....L.G.Q. H........- .--....... .......... .........

Castor_canadensis --.PPAG.RC -L-------. .--.-----. -.LLA....T .S.......F ....L.G.Q. HH.......- .--....... .......... .P.......

Cavia_porcellus --.PP.Q.-H -PL.-----. .--.-----F -.LLV..P.. SS.......F ....L...Q. HR......A- .--....... .......... .P.......

Chinchilla_lanigera --.PP.QL.. -P-C-----. .--.-----. -.LLV..P.. SS.......F ....L...Q. HH......A- .--....... .......... .P.......

Cricetulus_griseus --.P..RLHS -P-Y----P. .--.-----. -.LL..LAK. .S.......F ....L.G.Q. HR.......- .--....... .......... .P.......

Dipodomys_ordii MWGAAAQKHA -PLQ-----A .P-.-----. -.LLA..PP. .S.......F K...L.R.Q. H........- .--....... .....A.... .P......V

Fukomys_damarensis --.PPAQ..H -P-Y-----. .--.-----. -.LLA.WP.G .S.......F ....L...Q. HH......A- .--....... .......... .P.......

Grammomys_surdaster --.P..QLHC -P-.----L. .--M-----. -.VL..LPK. .S.......F ....L...Q. HH...Q...- .--....... .......... .P.......

Heterocephalus_glaber --.PPAQ..H -P-.-----. .--V-----. -.LLA.WP.. .S.......F ....L...Q. HH......T- .--....... .......... .P.......

Ictidomys_tridecemlineatus --.SPVR.WH -P-N-----. .--.-----. -.LLA..P.. LS.......F ....L...Q. HR.M.....- .--.D..... .......... .P.......

Jaculus_jaculus --.PPAQQSC -P-.----L. .--.----L. -.LL...P.. .S.......F ....L.G.Q. HH.......- .--....... .......... .P.......

Marmota_flaviventris --.SPVQ.WR -P-N-----. .--.-----. -.LLA..P.. LS.......F ....L...Q. HR.M.....- .--ND..... .......... .P.......

Marmota_marmota --.SPVR.WR -P-N-----. .--.-----. -.LLA..PP. LS.....LVF ...QL...Q. HR.M.....- .--ND..... .......... .P.......

Mastomys_coucha MWGVAIQRHA -P-.----PA P--.SPPAAA -ALDAILSE. .S.......F ....L...Q. HH.......- .--....... .......... .P.......

Meriones_unguiculatus --.P..RLHC -A-.------ ---------. -.LL.WLAR. .S.....S.F ....F.G.Q. .H.......- .--....... .......... .P.......

Mesocricetus_auratus --.P..QLHS -P-Y----P- .--.-----. -.LL..LAK. .S.......F ....L.G.Q. HH.......- .--....... .......... .........

Microtus_ochrogaster --.PC.PLHS -P-.------ .--.-----. -.LL..LPK. .S.......F ....L.G.Q. HH.......- .--....... .......... .P.......

Mus_caroli --.P..QLHC -P-.----L. .--.-----. -.VL..VPE. .S.......F ....L...Q. HH.......- .--....... .......... .P.......

Mus_musculus --.P..QLHC -P-.----L. .--.-----. -.VL..LPE. .S.......F ....L...Q. HH.......- .--....... .......... .P.......

Mus_pahari --.P..QLHC -P-.-----. .--.-----. -.VL..LPE. .S.......F ....L...Q. HH.......- .--....... .......... .P.......

Mus_spicilegus --.P..QLHC -P-.----L. .--.-----. -.VL..LPE. .S.......F ....L...Q. HH.......- .--....... .......... .P.......

Nannospalax_galili --.PPAQ.RC -T-.------ .--.----L. -.LL...P.. .S.......F ....L.G.Q. HH.......- .--....... .......... .........

Octodon_degus --.PPIQSHH -P-Y-----. .--.-----. -.LLA..P.V SS.......F ....L...Q. HH......A- .--....... .......... .P.......

Onychomys_torridus --.P..QLHS -P-.------ .--.-----. -.LL-.LPKV .S.......F ....L.G.Q. HH.......- .--....... .......... .P.......

Peromyscus_leucopus --.P.RQ.HS -P-.----L. .--.-----. -.LL-.LPR. .S.......F ....L.G.Q. HH.......- .--....... .......... .P.......

Peromyscus_maniculatus --.P..Q.HS -P-.-----. .--.-----. -.LL-.LPR. .S.......F ....L.G.Q. HH.......- .--....... .......... .P.......

Rattus_norvegicus --.L..QLHC -P-Y----L. .--.-----. -VVL..LPK. .S.......F ....L...Q. HH.......- .--....... .......... .P.......

Rattus_rattus --.L..QLHC -P-Y----L. .--.-----. -VVL..LPK. .S.......F ....L...Q. HH.......- .--....... .......... .P.......

Urocitellus_parryii --.SPVR.WH -P-N-----. .--.-----. -.LLA..P.. LS.......F ....L...Q. HR.M.....- .--.D..... .......... .P.......

1.50 12.50 2.50

> >> **$** TM1 <<< **$** >>> **$**

P PP P P P PP P P PP P PP P P G G

Monodelphis_domestica SCPWYLPWHH KVQHRFVFKK CGPDGQWVKG PQGQALRNAS QCEMDINEIW SQNKSAQMYS SFQVMYTVGY SMSLGALLLA LLILLGFSKL HCTRNYIHM

Phascolarctos_cinereus .......... .......... .......... .....W.... ..Q....... ......L... ...M...... ....A..... .......... .........

Sarcophilus_harrisii .......... .......... .......... .....W.... ..Q...S... .......... .......... .L...T.... .......... .........

Vombatus_ursinus .......... .......... .......... ....S..... ..Q...S... .......... .......... .......... .......... .........

Echinops_telfairi .......... .....L.... ........R. ....PW.D.. ..Q..MD.LK D.KEVVK... ...M...... CL........ .T...SL.Q. .........

Elephantulus_edwardii ........Y. .....L...T ........R. ....PW.... ..Q..QD.LE D.KEVVK... .......... CL.....F.. .A....L.Q. .........

Orycteropus_afer ........Y. .....L...Q ........R. .R..PW.... ..Q..AD.LK V.KEVVR..G G......... CL........ .A....L.Q. ........I

Trichechus_manatus .......... .....L.... ........R. ....PW.... ..Q..KE.LE A.KEMVK... ...M...... CL..A..... .A....V.Q. .........

Balaenoptera_acutorostrata .......... .....L...R ........R. .R..PW.... ..Q..DK.LE V.KEV.K... .......... .L........ .A....L... ........V

Balaenoptera_musculus .......... .....L...R ........R. .R..PW.... ..Q..DK.LE V.KEV.K... .......... .L........ .A....L... ........V

Delphinapterus_leucas .......... .....L...R ........R. .R..PW.... ..Q..DK.LE V.KEV.K... .......... .L........ .A....L... ........V

Globicephala_melas .......... .....L...R ........R. .R..PW.... ..Q..DK.LE V.KEV.K... .......... .L........ .A....L... ........V

Lagenorhynchus_obliquidens .......... .....L...R ........R. .R..PW.... ..Q..DK.LE V.KEV.K... .......... .L........ .A....L... ........V

Lipotes_vexillifer .......... .....L...R ........R. .R..PW.... ..Q..DK.LE V.KEV.K... .......... .L........ .A....L... R.......V

Monodon_monoceros .......... .....L...R ........R. .R..PW.... ..Q..DK.LE V.KEV.K... .......... .L........ .A....L... ........V

Neophocaena_asiaeorientalis .......... .....L...R ........R. ....PW.... ..Q..DK.LE V.KEV.K... .......... .L........ .A....L... ........V

Orcinus_orca .......... .....L...R ........R. .R..PW.... ..Q..DK.LE V.KEV.K... .......... .L........ .A....L... ........V

Phocoena_sinus .......... .....L...R ........R. ....PW.... ..Q..DK.LE V.KEV.K... .......... .L........ .A....L... ........V

Physeter_catodon .......... ....HL...R ........R. .R..PW.... ..Q..DK.LE V.KEV.K... .......... .L........ .A....L... ........V

Bos_indicus ........Y. .....L...R ........R. .R..PW.D.. ..Q..DE.LE V.KEV.K... .......... .L........ .TT...L... .........

Bos_mutus ........Y. .....L...R ........H. .R..PW.D.. ..Q..DE.LE V.KEV.K... .......... .L........ .TT...L... .........

Bos_taurus ........Y. .....L...R ........R. .R..PW.D.. ..Q..DE.LE V.KEV.K... .......... .L........ .TT...L... .........

Bubalus_bubalis ........Y. ....HL...R ........R. .R..PW.D.. ..Q..DK.LE V.KEV.K... .......... .L........ .AT...L... .........

Camelus_dromedarius .........R .........R ........R. .R..PW.... ..QV.DE.LA V.KEA..... .......... .L........ .A....L... ........V

Camelus_ferus .........R .........R ........R. .R..PW.... ..QV.DE.LA V.KEA..... .......... .L........ .AV...L... ........V

Capra_hircus ........Y. .....L...R ........R. .R..PW.D.. ..Q..DE.LE V.KEV.K... .......... .L........ .AT...L... .........

Cervus_hanglu ........Y. .....L...R ........R. .R..PW.... ..Q..NE.LE V.KEV.K... .......... .L........ .AT...L... .........

Moschus_moschiferus ........Y. .....L...R ........R. .R..PW.... ..Q..DK.LE V.KEV.K..R .......... .L........ .AT...L... .........

Odocoileus_virginianus ........Y. .....L...R ........R. .R..PW.... ..Q..DE.LE V.KEV.K... .......... .L.....F.. .AT...L... .........

Ovis_aries ........Y. .....L...R ........R. .R..PW.D.. ..Q..DE.LE V.KEV.K... .......... .L........ .AT...L... .........

Sus_scrofa ........Y. .....L...R ........R. .R..PW.... ..QV.DE.LG V.REV.E... ...A...A.. .L..A..... .A....L... ........A

Acinonyx_jubatus .......... .....L.... ........R. .R..PW.... ..Q..DD..E V.KEV.K..N .......... .L........ .AT...L... ........A

Callorhinus_ursinus .......... ...Q.L.... ........R. .R..SW.D.. ..Q..EK..E V.KEV.K... .......... .L........ .A....L... ........V

Canis_lupus .......... .....L.... ........R. .R..SW.... ..QL.GQ..E V.KEV.K... .......... .L........ .VV...L... R.......A

Enhydra_lutris .........P .....L.... ........R. ....SW.... ..Q..EH.LE G.KEA.E... GL........ .L........ .AL...L.Q. R.......A

Eumetopias_jubatus .......... ...Q.L.... ........R. .R..SW.D.. ..Q..EK..E V.KEV.K... .......... .L........ .A....L... ........V

Felis_catus .......... ..R..L.... ........R. .R..PW.... ..Q..DD..E V.KEV.K..N .......... .L........ .AT...L... ........A

Halichoerus_grypus .......... .......... ........R. .R..SW.D.. ..Q..EK..E V.K.A.K... G......... .L........ .A....L... ........V

Leptonychotes_weddellii .......... .....L.... ........R. .R..SW.D.. ..Q..EK..E V.KEA.K... G......... .L........ .A....L... ........V

Lontra_canadensis .........P .....L.... ........R. ....SW.... ..Q..EH.LE G.KEA.E... GL........ .L........ .AL...L.Q. R.......A

Lynx_canadensis .......... .....L.... ........R. .R..PW.... ..Q..DD..E V.KEV.K..N .......... .L........ .AT...L... ........A

Mirounga_leonina .......... .....L..R. ........R. .R..SW.D.. ..Q..EK..E A.KEA.K... G......... .L........ .A....L... ........V

Mustela_erminea .........P .....L.... ........R. .R..SW.... ..Q..EH.LK G.KEV.E... GL........ .L........ .AL...L.Q. R.......A

Neovison_vison .........P .....L.... ........R. .R..SW.... ..Q..ER.LE G.KEA.E... GL........ .L........ .AL...L.Q. R.......A

Odobenus_rosmarus .......... .....L.... ........R. .R..SW.D.. ..Q..EQ..E V.KEV.K... .......... .L........ .A....L... ........V

Phoca_vitulina .......... .......... ........R. .R..SW.D.. ..Q..EK..E V.K.A.K... G......... .L........ .A....L... ........V

Suricata_suricatta .......... .....L.... ........R. .R..PW.... ..Q..ED..E V.KDA.K..G R..A...... .L........ .AT...L... R.......A

Ursus_americanus .......... .....L.... ........R. .G..SW.... ..Q..DH..E V.KEV.KL.. G.R....... .L........ .A....L... ........A

Ursus_arctos .......... .....L.... ........R. .G..SW.... ..Q..DH..E V.KEV.KL.. ..R....... .L........ .A....L... ........A

Ursus_thibetanus .......... .....L.... ........R. .G..SW.... ..Q..DH..E V.KEV.KL.. G.R....... .L........ .A....L... ........A

Zalophus_californianus .......... ...Q.L.... ........R. .R...W.D.. ..Q..EK..E V.KEV.K... .......... .L........ .A....L... ........V

Artibeus_jamaicensis ........Y. ....G..Y.R ........R. ....P..... ..Q..ED.VK V.KEA.KL.G AS........ CL........ .A....LRR. ........A

Desmodus_rotundus ........Y. ....G..Y.R ........R. .R..PW.D.. ..Q..EE.VK V.KEA.KL.G AS........ CL........ .A....LRR. ........A

Hipposideros_armiger ........Y. .......Y.R ...N....R. ....SW.... ..Q..DD..E V.KEV.K... .......... CL........ .A....L... ........A

Miniopterus_natalensis ........Y. .......Y.R ........R. .R..PW.... ..Q..NE..V V.KEV.KL.G .......... CL.....I.. .V....LRR. ........V

Molossus_molossus .......... .......Y.R ........R. .R..PW.... ..QL.ED.LK A.KET.V... T..A...... CL........ .A....LR.. ........V

Myotis_myotis A......... .......Y.A ........R. ....P..... ..R..KE.LE A.EAE.KLLG GSKA...A.. CL........ .A....LR.. ........V

Phyllostomus_discolor P........R ....G..Y.T ........R. .R..PW.... ..Q..EE.VK V.REE.KL.G AS.A...... CL........ .A....LRR. ........A

Pipistrellus_kuhlii ........Y. .......Y.R ........R. .R..PW.... ..Q..EE.VK V..GV.RL.R G......... CL........ .AV...LRR. R.......A

Rousettus_aegyptiacus P.......Y. .....L.Y.R .E.....AR. .R..PW.... ..QLEDD..E V.KEV.K... .......... CL........ .A....L... ........V

Sturnira_hondurensis ........Y. ....G..Y.R ........R. ....PW.... ..Q..EE.VK V.KEV.KL.G A..G...... CL........ .A....LRR. ........A

Talpa_occidentalis .......... .....L...R .......AR. .R..PW.D.. ..Q..EA.ME D.EEE.R.LA ..RA..S... .L..A..... .A....L... R.......A

Ceratotherium_simum ........Y. .....L...R ........R. ....PW.... ..Q..DE..E V.REV.K... .......... .L........ .A....L... ........V

Equus_caballus .......... .....L...R ........R. .R..PW.... ..Q..DE..E V.KEV.K..N .......... .L........ .A....L... .........

Manis_pentadactyla .......... ....H....R ........R. .R..PW.... ..R..DE.LK V.KEA.K... G......... .L........ .V....L... ........A

Galeopterus_variegatus .......... .....L...T ........R. .R..PW.... ..Q..AE..E V.KEV.K... .......... AL........ .A....L... ........V

Callithrix_jacchus .......... .........R ...N....R. .R..PW.... ..QI.GE..E V.KEV.K... ...A...... .L........ .AL.G.L... .....A..A

Cebus_capucinus .......... .....Y...R ........R. .G..PW.... ..QI.GE.TD V.KEV.R... ...A..S... TL........ .AL.G.L... .....A..A

Cercocebus_atys .......... .........R ........R. .R..PW.D.. ..Q..GE.LE V.KEV.K... .......... .L........ .A..G.I... .....A..A

Chlorocebus_sabaeus .......... .........R ...N....R. .R..PW.D.. ..QI.GE.LE V.KEV.K... .......... .L........ .A..G.I... .....A..A

Colobus_angolensis ........Y. .........R ........R. .R...W.D.. ..Q..GE..E V.KEV.K... R......... .L........ .A..G.L... .....A..A

Gorilla_gorilla .......... .........R ........R. .R..PW.D.. ..Q..GE.TE V.KEV.K... .......... .L........ .A..G.L... .....A..T

Homo_sapiens .......... .........R ........R. .R..PW.D.. ..Q..GE..E V.KEV.K... .......... .L........ .A..G.L... .....A..A

Hylobates_moloch .......... .........R ........R. .R..PW.D.. ..Q..GE..E V.KEV.K... .......... .L........ .A..G.L.T. .....A..A

Macaca_fascicularis .......... .........R ........R. .R..PW.D.. ..Q..GE.LE V.KEV.K... .......... .L........ .AV.G.I... .....A..A

Macaca_mulatta .......... .........R ........R. .R..PW.D.. ..Q..GE.LE V.KEV.K... .......... .L........ .AV.G.I... .....A..S

Macaca_nemestrina .......... .........R ........R. .R..PW.D.. ..Q..GE.LE V.KEV.K... .......... .L........ .A..G.I... .....A..A

Mandrillus_leucophaeus .......... .........R ........R. .R..PW.D.. ..Q..GE.LE V.KEV.K... .......... .L........ .A..G.I... .....A..A

Microcebus_murinus .......... .....L...R ........R. ....PW.... ..Q..DE.LQ V.KEV.K... ...E...... .L.....V.. .A........ ........A

Nomascus_leucogenys .......... .........R ........R. .R..PW.D.. ..Q..GE..E V.KEV.K... .......... .L.....I.. .A..G.L.T. .....A..A

Otolemur_garnettii .......... .........R ........Q. .R..PW.... ..QL.DE.LE V.KEVVK... ...GV..... .L........ .AT...L... ......V.A

Pan_paniscus .......... .........R ........R. .R..PW.D.. ..Q..GK..E V.KEV.K... .......... .L........ .A..G.L... .....A..A

Pan_troglodytes .......... .........R ........R. .R..PW.D.. ..Q..GK..E V.KEV.K... .......... .L........ .A..G.L... .....A..A

Papio_anubis .......... .........R ........R. .R..PW.D.. ..Q..GE.LE V.KEV.K... .......... .L........ .A..G.I... .....A..A

Piliocolobus_tephrosceles ........Y. ....G....R ........H. .R..PW.D.. ..Q..GE..E V.KEV.K... R......... .L........ .A..G.L... .....A..A

Pongo_abelii .......... .........R ........R. .R..PW.D.. ..Q..GE..E V.KEV.K... .......... .L........ .A..G.L... .....A..A

Propithecus_coquereli .......... .....L...R ........R. ....PW.... ..Q..DE.LK V.KEV.K... ...E...... .L.....V.. .A....L... ........A

Rhinopithecus_bieti ........Y. .........R ........R. .R..PW.D.. ..Q..GE..E V.KEV.K... .......... .L........ .A..G.L... .....A..A

Rhinopithecus_roxellana ........Y. .........R ........R. .R..PW.D.. ..Q..GE..E V.KEV.K... .......... .L........ .A..G.L... .....A..A

Saimiri_boliviensis .......... .........R ........R. .R..PW.... ..QI.DE.TE V.KEV.KL.. ...AV..... .L........ .AL.G.L... .....A..A

Sapajus_apella .......... .....Y...R ........R. .G..PW.... ..QI.GE.TD V.KEV.R... N..A..S... TL........ .AL.G.L... .....A..A

Theropithecus_gelada .......... .........R ........R. .R..PW.D.. ..Q..GE.LE V.KEV.K... .......... .L........ .A..G.I... .....A..A

Trachypithecus_francoisi ........Y. .........R ........R. .R..PW.D.. ..Q..SE..E V.KEV.K... .......... .L........ .A..G.L... .....A..A

Ochotona_princeps A.......Y. .........R ........R. .R..PW.... ..QL.MD.ME A.KEV..L.. ...AT..... GL........ .AV...C... ........A

Arvicanthis_niloticus ........Y. .....L...R ........R. .R..PW.... ..Q..DE..E V.KGV.KL.. .Y........ .L........ .V....LR.. ........G

Arvicola_amphibius ........Y. .....L...R ........R. .R..PW.... ..Q..DK..E V.KEE.K... RY........ .L........ .V....LR.M ........G

Castor_canadensis ........Y. .....L...R .......ER. .R..PW.... ..Q..NE..E V.KEV.K... .......T.. .L........ .A....L... ........G

Cavia_porcellus ........Y. .....L.... ........R. .H..PW.... ..Q..DQ..E V..EV.K... .......... .L..A..... .A....L... ........L

Chinchilla_lanigera ........Y. .....L.... ........R. .R..PW.... ..Q..DQ..E V.KEV.K... .......... .L........ .A....L... ........L

Cricetulus_griseus ........Y. .....L...R ........R. .R..PW.... ..Q..DE..Q V.KEV.K... .Y........ .L.....I.. .V....LR.. ........G

Dipodomys_ordii ........Y. .....L...R ........P. ....PR.... ..Q..ME..E V.REA.KL.. .......M.. .L........ .A....L... ........G

Fukomys_damarensis ........Y. .....L...E ........R. .R..PW.... ..Q..DQ..E V.KEV.K... R..M...... .L........ .A...AL... ........L

Grammomys_surdaster ........Y. .....L...R ........R. .R..PW.... ..Q..DE..E V..GV.K... .Y........ .L........ .V....LR.. ........G

Heterocephalus_glaber ........Y. .....L...E ...N....R. .R..PW.... ..Q..DQ..E A.KEV.K... .......... .L........ .A....L... ........L

Ictidomys_tridecemlineatus ........Y. .....L.... ........R. .R..PW.... ..Q.EDE.LE G.KEV.K... .......... .L........ .A.M..L... ........V

Jaculus_jaculus ........Y. .....L...R ........R. .R..PW.... ..Q..DE..E V.KEV.K... .......... .L........ .AV..SL... ........G

Marmota_flaviventris ........Y. .....L.... ........R. .R..PW.... ..Q.EDE.LE G.KEV.K... .......... .L........ .A.M..L... ........V

Marmota_marmota ........Y. .....L.... ........R. .R..PW.... ..Q.EDE.LE G.KEV.K... .......... .L........ .A.M..L... ........V

Mastomys_coucha ........Y. .....L...R ........R. .R..PW.... ..Q..DE..E V.KGV.K... .Y........ .L........ .V....LR.. ........G

Meriones_unguiculatus ........Y. .....L...R ........R. .R..PW.... ..QL.EE..E V.KEV.K... NY........ .L..A..... .V....LR.. ........A

Mesocricetus_auratus ........Y. ....GL...R ........R. .R..PS.... ..Q..DE..Q V.KEV.K... .Y........ .L........ .V....LR.. R.......G

Microtus_ochrogaster ........Y. .....L...R ........R. .R..PW.... ..Q..DE..E V.KEV.K... .Y........ .L........ .V....LR.M ........G

Mus_caroli ........Y. .....L...R ........R. .R..PW.... ..QL.DE..E V.KGV.K... .Q........ .L........ .V....LR.. ........G

Mus_musculus ........Y. .....L...R ........R. .R..PW.... ..QL.DE..E V.KGV.K... .Q........ .L........ .V....LR.. ........G

Mus_pahari ........Y. .....L...R ........R. .R..P..... ..QL.DE..E V.KGE.K... .Y........ .L........ .V....LR.. ........G

Mus_spicilegus ........Y. .....L...R ........R. .R..PW.... ..QL.DE..E V.KGV.K... .Q........ .L........ .V....LR.. ........G

Nannospalax_galili ........Y. .....L...R .....K..R. ....PW.... ..Q..DE..E V.KDV.K..N .Y.L...... GL........ .V....LR.. ........G

Octodon_degus ........Y. .....L.... ........R. ....PW.... ..Q..DQ..E I.KEA.K... .......... .L......F. .A....L... ........L

Onychomys_torridus ........Y. .....L...R ........R. .R..PW.... ..Q..DE..K V.KEV.K... .Y........ .L........ .V....LR.. ........G

Peromyscus_leucopus ........Y. .....L...R ........R. ....PW.D.. ..Q..DE..E V.KEV.K... .Y........ .L........ .V....VR.. .....C..G

Peromyscus_maniculatus ........Y. .....L...R ........R. ....PW.... ..Q..DE..E V.KEV.K... .Y........ .L........ .V....VR.. .....C..G

Rattus_norvegicus ........Y. .....L...R ........R. .R..SW.D.. ..Q..DD..E V.KGV.K... .Y........ .L........ .V....LR.. ........G

Rattus_rattus ........Y. .....L...R ........R. .R..SW.D.. ..Q..DD..E V.KGV.K... .Y........ .L........ .V....LR.. ........G

Urocitellus_parryii ........Y. .....L.... ........R. .R..PW.... ..Q.EDK.LE G.KEV.K... .......... .L........ .A.M..L... ........V

3.50 4.50

TM2 <<< >>> TM3 **$** <<< >>> TM4 **$**

G G P PP P P PP P PPP P P PP PP P G GG G

Monodelphis_domestica NLFVSFILKA SSVLVIDALL KTRYSQKIGD DLSMSIWLSD EAVAGCRVAT VFMQYGIVAN YCWLLVEGVY LHNLLVL-AV FSEKSYFNLY LGIGWGAPL

Phascolarctos_cinereus ...A...... .......... .......... ...V...... .......... .......... .......... .......-.. .......... .V......I

Sarcophilus_harrisii .......... .......... .......... ...V.V...N .......... .......... .......... .......-.. .......... ........I

Vombatus_ursinus ...A...... .......... .......... ...V...... .......... .......... .......... .......-.. ........F. .V......I

Echinops_telfairi ...A..V... ....AM.... Q.....Q... ...V.V.... R........A ......V... .......... ..S..G.-.T .P.R.F.PI. ........I

Elephantulus_edwardii ...A..V... ....AT.... Q......V.. ...V...... Q...S....V .......... ........L. ..SV.HR-.A .Q.DGF.P.. V........

Orycteropus_afer ...A..V... G...AM.... Q......... ...V.V.... R........A .......... .......... ..S..G.-.A .P.RRL.P.. ........M

Trichechus_manatus ...A..V... ....AT.... Q........N ...V...... G........A ......V... .......... ..S..GQ-.A .T.RRF.T.. ........M

Balaenoptera_acutorostrata ...A..V... .......T.. ......R... ...V...... G........A ......V... .......... ..S..GF-.T IP.R.C.P.. ........M

Balaenoptera_musculus ...A..V... .......T.. ......R... ...V...... G........A ......V... .......... ..S..GF-.T IP.R.C.P.. ........M

Delphinapterus_leucas ...A..V... .......T.. ......R... .F.V.V.... G........A ......V... .......... ..S..SF-.T IP.R.C.P.. ........M

Globicephala_melas ...A..V... .......T.. ......R... .F.V.V.... G........A ......V... .......... ..S..SF-.T IP.R.C.P.. ........M

Lagenorhynchus_obliquidens ...A..V... .......T.. ......R... .F.V.V.... G........A ......V... .......... ..S..SF-.T IP.R.C.P.. ........M

Lipotes_vexillifer ......V... .......T.. ......R... .F.V.V.... G........A ......V... .......... ..S..SF-.T IP.R.C.P.. ........V

Monodon_monoceros ...A..V... .......T.. ......R... .F.V.V.... G........A ......V... .......... ..S..SF-.T IP.R.C.P.. ........M

Neophocaena_asiaeorientalis ...A..V... .......T.. ......R... .F.V.V.... G........A ......V... .......... ..S..SF-.T IP.R.C.P.. ........M

Orcinus_orca ...A..V... .......T.. ......R... .F.V.V.... G........A ......V... .......... ..S..SF-.T IP.R.C.P.. ........M

Phocoena_sinus ...A..V... .......T.. ......R... .F.V.V.... G........A ......V... .......... ..S..SF-.T IP.R.C.P.. ........M

Physeter_catodon ...A..V... .......T.. ......R... .F.V.V.... G........A ......V... .......... ..S..GF-.T IP.R.C.P.. ........M

Bos_indicus ......M... .......T.. ......R... .I.V.V.... G........A ......V... .......... ..S..S.-.A IP.R.C.P.. ........M

Bos_mutus ......M... .......T.. ......R... .I.V.V.... G........A ......V... .......... ..S..S.-.A VP.R.C.P.. ........M

Bos_taurus ......M... .......T.. ......R... .I.V.V.... G........A ......V... .......... ..S..S.-.A IP.R.C.P.. ........M

Bubalus_bubalis ......M... .......T.. ......R... .I.V.V.... G........A ......V... .......... ..S..S.-.A VP.R.C.P.. ........M

Camelus_dromedarius ...T..V... ....A..... ......R... ...V.V.... G........A ......V... ........L. ..G..G.-.A APGR.C.G.. ........M

Camelus_ferus ...T..V... ....A..... ......R... ...V.V.... G........A ......V... ........L. ..G..G.-.A APGR.C.G.. ........M

Capra_hircus ......M... .......T.. ......R... .I.V.V.... G......... ......V... .......... ..S..S.-.T VP.R.C.P.. ........M

Cervus_hanglu ......M... .......T.. ......R... .I.V.V.... G........A .......... .......... ..S..S.-.A VP.R.C.P.. ........M

Moschus_moschiferus ......M... .......T.. ......R... .I.V.V.... G........A ......V... .......... ..S..S.-.A VP.R.C.P.. ........M

Odocoileus_virginianus ......M... .......T.. ......R... .I.V.V.... .........A .......... .......... ..S..S.-.A VP.R.C.P.. ........M

Ovis_aries ......M... .......T.. ......R... .I.V.V.... G........A ......V... .......... ..S..S.-.A VP.R.C.P.. ........M

Sus_scrofa ..LA..V.R. ....AL.... ......RL.. ...V...... .........A ......V... .......... ..S..RQ-.T IP.R.C.P.. .A......M

Acinonyx_jubatus ...A..V... .......T.. .......... ...V.V.... G........A .......... .......... ..S..G.-.A .P.R.F.A.. ..L.....M

Callorhinus_ursinus ...A..V... .......T.. R......... ...V.V.... G........A ......V... .......... ..G..G.-.A .P.R.FLA.. ........M

Canis_lupus ...A..V... G......... .......... ...V.V...N G........A ......V... .......... ..S..GR-.T .P.R.F.P.. .AV.....V

Enhydra_lutris ...A..V... .......... .......... ..RV...... G........A ......V... .S........ ..S..G.-.A LPDR.F.A.. ........M

Eumetopias_jubatus ...A..V... .......T.. RM........ ...V.V.... G........A ......V... .......... ..G..G.-.A .P.R.FLA.. ........M

Felis_catus ...A..V... .......T.. .......... ...V.V.... G........A .......... .......... ..S..G.-.A .P.R.F.A.. ..L.....M

Halichoerus_grypus ...A..V... G......T.. R......... ...V...... G........A ......V... .......... ..G..G.-.A .P.R.F.A.. ........M

Leptonychotes_weddellii ...A..V... G......T.. R......... ...V...... G........A ......V... .......... ..G..G.-.A .P.R.F.V.. ........M

Lontra_canadensis ...A..V... .......... .......... ..RV...... R........A ......V... .S........ ..S..G.-.A LPDR.F.A.. ........M

Lynx_canadensis ...A..V... .......T.. .......... ...V.V.... G........A .......... .......... ..S..G.-.A .P.R.F.A.. ..L.....M

Mirounga_leonina ...A..V... G......T.. R......... ...V...... G........A ......V... .......... ..G..G.-.A .P.R.F.A.. ........M

Mustela_erminea ...A..V... .......... E......... ..RV.V.... G........A ......V... .S........ ..S..G.-.A LPDR.F.A.. ........M

Neovison_vison ...A..V... .......... .......... ..RV.V.... G........A ......V... .S........ ..S..G.-.A LPDR.F.A.. ........M

Odobenus_rosmarus ...A..V... G......T.. R......... ...V...... G........A ......V... .......... ..G..G.-.A .P.R.F.A.. ........M

Phoca_vitulina ...A..V... G......T.. R......... ...V...... G........A ......V... .......... ..G..G.-.A .P.R.F.A.. ........M

Suricata_suricatta ...A..V... .......... R......... ...V.V.... G........A ...H...... .....A.... ..G..G.-.S .PDR.C.A.. ........M

Ursus_americanus ...A..V.R. .......V.. E......V.. .....V.... G........A ......V... .......... ..G..G.-.A .P.R.C.A.. VA......V

Ursus_arctos ...A..V.R. .......V.. E......V.. .....V.... G........A ......V... .......... ..G..G.-.A .P.R.C.A.. VA......V

Ursus_thibetanus ...A..V.R. .......V.. E......V.. .....V.... G........A ......V... .......... ..G..G.-.A .P.R.C.A.. VA......V

Zalophus_californianus ...A..V... .......T.. R......... ...V.V.... G........A ......V... .......... ..G..G.-.A .P.R.FLA.. ........M

Artibeus_jamaicensis ......V.R. ....AT.... R.....Q... ...VRS...G G.L......A ......V... ........L. ..S..G.-.A GP...L.A.. V.V...T.M

Desmodus_rotundus ......A.R. ....A..... R.....E... ...V.G.... G.L.S..A.A ......V... .......... ..S..G.-.A GP.R.S.A.. ........M

Hipposideros_armiger ...A..V... .......T.. .......... ..GV.V.... G........A ......V... .......... ..S..G.-.T .P.R.F.T.. ........M

Miniopterus_natalensis ...A..V... .......... .......V.. ...V.G.... G........A ......V... .......... ..S..G.-.A CP.R.F.T.. ........M

Molossus_molossus ...A..V.R. .......T.. R......V.. ...L.G.... G........A .V....V... .......... ..S..S.-.A CRDR.LST.. ........V

Myotis_myotis ...A..V... G....T.L.. ......EF.. ..RVRG.M.. ..L......A .......... A.......L. ..R..R.-SA GP.R.V.AF. ........M

Phyllostomus_discolor ...L..V.R. ....AV.... R.....N... ...V.S...G G.L......A ......V... .....A.... ..S..G.-.A GP.R.F.S.. ........M

Pipistrellus_kuhlii ...A..V... G...A..T.. Q...G.Q... ...VRG.... G......... ......V... A......... .LS..G.-ST GP.R...A.. ........M

Rousettus_aegyptiacus ...A..V... ....A..... .......M.. ...V.V...N G.L......A ......V... .......... ..S..GP-.T ...RDF.TY. ........M

Sturnira_hondurensis ......V.R. ....AM.... R......... ...V.S...G G.L......A ......V... .......... ..S..G.-.A GP.R.F.T.. ..V.....M

Talpa_occidentalis H..L..V... G...AT.... Q...G.Q... ...V.V.... G........A ......V... ........L. ..G..GR--P VPRR.C.P.. ........M

Ceratotherium_simum ......V... .......T.. E......... ...V...... .........A .......M.. .......... ..S..G.-.T .P.R.F.A.. ........M

Equus_caballus ......V... .......T.. E......... ...V...... .........A .......... .......... ..S..G.-.T .P.R.F.T.. .S......M

Manis_pentadactyla ...A..V... ....A..... ....N..... ...V.....G G......A.. ......VM.. ........M. ..S..G.-.T AP.R.F.T.. .TV.....M

Galeopterus_variegatus ...L..V.R. .......R.. .......... ...V.V...S G........A ......V... .......... ..S..G.-.T LP.R.F.A.. M.......M

Callithrix_jacchus ...A..V... G......G.. R......... ...V...... G........A ...H...... ........L. ..S..G.-.T LPKR.F.R.. ........M

Cebus_capucinus ...A..V... G......G.. R.......E. ...V...... G......... ...H...... ........L. ..S..G.-.T LP.R.F.R.. ........M

Cercocebus_atys ......V... .......G.. R......... ...V...... G........A ......V... ........L. .....G.-.T LP.R.F.S.. ........M

Chlorocebus_sabaeus ......V... .......G.. R......... ...V.T.... G.M......A ......V... ........L. .....G.-.T LP.R.F.S.. ........M

Colobus_angolensis ......V... .......G.. R......... ...VRT.... G.L......A ......V... ........L. .....G.-.T LP.R.F.S.. ........M

Gorilla_gorilla ...A..V... .......G.. R......... ...V...... G........A .......... ........L. .....G.-.T LP.R.F.S.. ........M

Homo_sapiens ...A..V... .......G.. R......... ...V.T.... G........A .......... ........L. .....G.-.T LP.R.F.S.. ........M

Hylobates_moloch ...A..V... .......G.. R......... ...V...... G........A .......... ........L. .....G.-.T LP.R.F.S.. ........M

Macaca_fascicularis ......V... .......G.. R......... ...V...... G........A ......V... ........L. .....G.-.T LP.R.F.S.. ........M

Macaca_mulatta ......V... .......G.. R......... ...V...... G........A ......V... ........L. .....G.-.T LP.R.F.S.. ........M

Macaca_nemestrina ......V... .......G.. R......... ...V...... G........A ......V... ........L. .....G.-.T LP.R.F.S.. ........M

Mandrillus_leucophaeus ......V... .......G.. R......... ...V...... G........A ......V... ........L. .....G.-.T LP.R.F.S.. ........M

Microcebus_murinus ...A..V.R. .......Q.. .......... ..--.L.... G........A ......V... .......... ..S..D.-.T .P.R.F.T.. ........M

Nomascus_leucogenys ...A..V... .......G.. R......... ...V...... G........A .......... ........L. .....G.-.T LP.R.F.S.. ........M

Otolemur_garnettii ...A..M... C......R.I .......... ...V...... G........S ......V... .........H ..S..GR-.T LP.R.R.T.. ........M

Pan_paniscus ...A..V... .......G.. R......... ...V.T.... G........A .......... ........L. .....G.-.T LP.R.F.S.. ........M

Pan_troglodytes ...A..V... .......G.. R......... ...V.T.... G........A .......... ........L. .....G.-.T LP.R.F.S.. ........M

Papio_anubis ......V... .......G.. R......... ...V...... G........A ......V... ........L. .....G.-.T LP.R.F.S.. ........M

Piliocolobus_tephrosceles ......V.R. .....V.G.. R...NR.... ...VRT.... GEL......A ......V... ........L. .....G.-.A LP.R.F.S.. ........M

Pongo_abelii ...A..V... .......G.. R......... ...V...... G........A .......... ........L. .....G.-.T LP.R.F.S.. ........M

Propithecus_coquereli ...A..V... .......Q.. ....N..... ...V...... G......M.A ......V... ........L. ..S..D.-.T .P.R.F.T.. ........M

Rhinopithecus_bieti ......V... .......G.. R......... ...VRS.... G........A ......V... ........L. .....G.-.T LP.R.F.S.. ........M

Rhinopithecus_roxellana ......V... .......G.. R......... ...VRS.... G........A ......V... ........L. .....G.-.T LP.R.F.S.. ........M

Saimiri_boliviensis ...A..V... G......G.. R......... ..TV...... G........A ...H...M.. ........L. ..S..G.-.T LP.R.F.R.. ........M

Sapajus_apella ...A..V... G......G.. R.......E. ...V...... G......... ...H...... ........L. ..S..G.-.T LP.R.F.R.. ........M

Theropithecus_gelada ......V... .......G.. R......... ...V...... G........A ......V... ........L. .....G.-.T LP.R.F.S.. ........M

Trachypithecus_francoisi ...A..V... .......G.. R......... ...VRT.... G........A ......V... ........L. ....MG.-.T LP.R.F.S.. ........M

Ochotona_princeps ...A..V... AA..AT.R.. Q...G.QL.. ...V.L.... G......... ......V... .......... ..S..G.-PT .P.R.R.S.. .........

Arvicanthis_niloticus ...A..V... G......W.. .......... ...V.V.... G......... .I.....I.. .......... .YR..S.-.T ...R.F.S.. .A.......

Arvicola_amphibius ...A..V... G....T.W.. ......T... V..V...... G......... .IT....I.. .......... .YS..S.-.T .Q...F.S.. .........

Castor_canadensis ...A..V... G......Q.. .......... ..RV.V.... G..T.....A .V........ .......... .....S.-.T .P.R.F.A.. .........

Cavia_porcellus ...A..V... G......R.. E......... ...V.V.... G........A .I...AV... .......... ..R..S.-.A .P.QGC.A.. .AT......

Chinchilla_lanigera ...A..M... G......W.. .......... ...V.V.... G........A .I....V... .......... ..S..S.-.T .P.R.F.M.. .A.......

Cricetulus_griseus ...A..V... G...A..W.. .......... ...V.V.... G......... .I.....I.. .........F ..S..S.-.T .Q...F.S.. .........

Dipodomys_ordii ...A..V... G...I..Q.. .M........ ...VG....S G........A .V........ .......... ..S..SR-.T .P.R.F.AV. ......T..

Fukomys_damarensis ...A..V... G......W.. .I........ ...V.V.... G......M.A .I....V... ........M. ..S..S.-.T .P.R.F.T.. .........

Grammomys_surdaster ...A..V... G......W.. .......... ...V...... G......... .I.....I.. .......... .YS..S.-.T ...R.F.S.. .........

Heterocephalus_glaber ...A..V... G......Q.. .......... ...V.V...N G........A .I....V... .......... ..S..S.-.T .P.R.F.S.. .........

Ictidomys_tridecemlineatus ...A..V... .......W.. .......... ...V...... G........A .I....V... .......... ..G..G.-SS .P.R.L.T.. .........

Jaculus_jaculus ...A..V... G......Q.. .......... ...V...... G........A .I........ .......... ..S..S.-.T .P.R.F.A.. .........

Marmota_flaviventris ...A..V... .......W.. .......... ...V...... G........A .I....V... .......... ..G..G.-.S .P.R.F.T.. .........

Marmota_marmota ...A..V... .......W.. .......... ...V...... G........A .I....V... .......... ..G..G.-.S .P.R.F.T.. .........

Mastomys_coucha ...A..V... G......W.. .......... ...V.V.... G.M....... .I.....I.. .......... .YS..S.-.T ...R.F.S.. .........

Meriones_unguiculatus ...A..V... G......W.. .......... ...V.V..R. .......... .I.....I.. .......... ..S..S.-.T .....F.S.. .........

Mesocricetus_auratus ...A..V... G......W.. .......... ...V.V.... G......... .V.....I.. .......... .YS..S.-.A .Q...FLF.. .C.......

Microtus_ochrogaster ...A..V... G....V.W.. .......... ...V.V.... G......... .IT....I.. .......... .YS..R.-.T .Q...F.S.. .........

Mus_caroli ...A..V... G......W.. .......... ...V.V.... G.M....... .I.....I.. .......... .YS..S.-.T ...R.F.S.. .........

Mus_musculus ...A..V... G......W.. .......... ...V.V.... G.M....... .I.....I.. .......... .YS..S.-.T ...R.F.S.. .........

Mus_pahari ...A..V... G......W.. .......... ...V.V.... G.M....... .I.....I.. .......... .YS..S.-.T ...R.F.Y.. .........

Mus_spicilegus ...A..V... G......W.. .......... ...V.V.... G.M....... .I.....I.. .......... .YS..S.A.A T..RRF.S.. .........

Nannospalax_galili ...T..V... G......Q.. .......... ...V....G. G........A .I........ .......... .YS..S.-.T .P.R.F.A.. .S.......

Octodon_degus ...A..V... G......K.. .A....N.R. ...V.V.... RT.....A.A .I........ .......... .YS..S.-.T .P.R.F.M.. .A.......

Onychomys_torridus ...A..V... G......W.. .......... ...V...... G......... .I.....I.. .......... .YS..S.-.T .P...F.S.. .........

Peromyscus_leucopus ...A..V... G......W.. .......... ...V.V.... G......... .I.....I.. .......... .YS..S.-.T .P...F.S.. .........

Peromyscus_maniculatus ...A..V... G......W.. .......... ...V.V.... G......... .I.....I.. .......... .YS..S.-.T .P...F.S.. .........

Rattus_norvegicus ...A..V... G......W.. .......... ...V.V.... G......... .I.....I.. .......... .YS..SI-TT .....F.S.. .C....S..

Rattus_rattus ...A..V... G......W.. .......... ...V.V.... G......... .I.....I.. .......... .YS..S.-.T .....F.S.. ......S..

Urocitellus_parryii ...A..V... .......W.. .......... ...V...... G........A .I....V... .......... ..G..G.-.S .P.R.F.T.. .........

45.50 5.50 6.50

<<< **$** >>> TM5 **$** << < >>> TM6 **$** <<<

P P PPP P P P G GG GG G G GG GGG P P P

Monodelphis_domestica LFVIPWVVVK FFFENIQCWT SNDNMGFWWI LRFPVFLAIL INFFIFIRII QILVSKLQAH QMRYTDYKFR LAKSTLTLIP LLGIHEVVFA FVTDEHAQG

Phascolarctos_cinereus .......... ..Y....... .......... .......... .......... ...I...... .......... .......... .......... .........

Sarcophilus_harrisii .......... ..Y....... .......... .......... .......... ...I...... .......... .......... .......... .........

Vombatus_ursinus ......I... ..Y....... .......... ..L....... .......... ...I...... .......... .......... .......... .........

Echinops_telfairi ...V..A... CL...V.... .......... ..V....... ..S...MHTV HL..A..R.. .......... .......... ...V...... .........

Elephantulus_edwardii ......A... CL........ .......... .......... ...L...HTV H..L...R.. ..HCI..... ........V. ...V...... .........

Orycteropus_afer ...T..A.L. CL...V.... ..G......V .......... ......VHT. H...A..R.. .......... ......S.V. ...V...L.. L....Q...

Trichechus_manatus ......A... GL........ ..N....... P......... .......HT. H..MA..R.. .V........ ........V. ...V...... .........

Balaenoptera_acutorostrata ...T...... CL........ .......... .......... .........L HL..V..R.. .......... .......... ...V...... .........

Balaenoptera_musculus ...T...... CL........ .......... .......... .........L HL..A..R.. .......... .......... ...V...... .........

Delphinapterus_leucas ...T...... CL........ .......... .......... ......V..L HL.AA..... ......C... .......... ...V...... .........

Globicephala_melas ...T...... CL........ .......... .......... .........L HL..A..... ..H....... .......... ...V...... .........

Lagenorhynchus_obliquidens ...T...... CL........ .......... .......... .........L HL..A..... .......... .......... ...V...... .........

Lipotes_vexillifer ...T...... CL........ .......... .......... ......T..L HL.AA..... .T....CRCP .......... ...V...... .........

Monodon_monoceros ...T...... CL........ .......... .......... ......V..L HL.AA..... ......C... .......... ...V...... .........

Neophocaena_asiaeorientalis ...T...... CL........ .......... .......... ......V..L HL.AA..... .......... .......... ...V...... .........

Orcinus_orca ...T...... CL........ .......... .......... .........L HL..A..... .......... .......... ...V...... .........

Phocoena_sinus ...T...... CL........ .......... .......... ......V..L HL.AA..... .......... .......... ...V...... .........

Physeter_catodon ...T...... CL........ .......... .......... .........L HL..A..R.. .......... .......... ...V...... .........

Bos_indicus ......A... CL........ .....A.... .......... .........L HL..A..R.. .......... .......... ...V...... .........

Bos_mutus ......A... CL........ .....A.... .......... .........L HL..A..R.. .......... .......... ...V...... .........

Bos_taurus ......A... CL........ .....A.... .......... .........L HL..A..R.. .......... .......... ...V...... .........

Bubalus_bubalis ......A... CL........ .....A.... .......... .........L HL..A..R.. .......... .......... ...V...... .........

Camelus_dromedarius ......A... CL........ .....A.... .......... .........L HV..A..R.. ........L. .......... ...V...... .........

Camelus_ferus ......A... CL........ .....A.... .......... .........L HV..A..R.. ........L. .......... ...V...... .........

Capra_hircus ......A... CL........ .....A.... .........V .........L HL..A..R.. .......... .......... ...V...... .........

Cervus_hanglu ......A... CL........ .....A.... .......... .........L HL..A..R.. .......... .......... ...V...... .........

Moschus_moschiferus ......A... CL........ .....A.... .......... .........L HL..A..R.. .......... .......... ...V...... .........

Odocoileus_virginianus ......A... CL........ .....A.... .......... .........L HL..A..R.. .......... .......... ...V...... .........

Ovis_aries ......A... CL........ .....A.... .........V .........L HL..A..R.. .......... .......... ...V...... .........

Sus_scrofa ......A... CL........ .......... .......... ...S....VL HV..A..R.. ...C...... ..R....... ...V...... .........

Acinonyx_jubatus ......A... CL........ .......... .......... ..LL..T.VL RV..A..R.R .......... .......... ...V...... .........

Callorhinus_ursinus ......A... CL........ .......... .......... .........L L..MA....R .......... .......... ...V...... .........

Canis_lupus ...V..A... CL........ .......... ..L....... ........VL L..MA..R.R ....S..... .......... ...V...... .........

Enhydra_lutris ......A... CL........ R....A.... .......... .........L L..TA..R.R .......... .......... ...V...... .........

Eumetopias_jubatus ......A... CL........ .......... .......... .........L L..MA....R .......... .......... ...V...... .........

Felis_catus ......A... CL........ .......... .......... ..LL..T.VL RV..A..R.R .......... .......... ...V...... .........

Halichoerus_grypus ......A... CL........ .......... .......... ........VL L..MA....R .......... .......... ...V...... .........

Leptonychotes_weddellii ......A... CL........ ..N....... .......... ........VL L..MA....R .......... .......... ...V...... .........

Lontra_canadensis ......A... CL........ R....T.... .......... ..S......L L..TA..R.R .......... .......... ...V...... .........

Lynx_canadensis ......A... CL........ .......... .......... ..LL..T.VL RV..A..R.R .......... .......... ...V...... .........

Mirounga_leonina ......A... CL........ ..N....... .......... ........VL L..MA....R .......... .......... ...V...... .........

Mustela_erminea ......A... CL........ R....A.... .......... .........L L..TA..R.R ...H..S... .......... ...V...... .........

Neovison_vison ......A... CL........ R....A.... .......... .........L L..TA..R.R ...H..S... .......... ...V...... .........

Odobenus_rosmarus ......A... CL........ .......... .......... .........L L..MA....R .......... .......... ...V...... .........

Phoca_vitulina ......A... CL........ .......... .......... ........VL L..MA....R .......... .......... ...V...... .........

Suricata_suricatta ...V..A... CL........ .......... ..L....... .........L F...A..R.R ........L. .......... ...V...... .........

Ursus_americanus F.....A... CL........ .......... .......... ...C....VL L..AA..R.R .......... .......... ...V...A.. .........

Ursus_arctos F.....A... CL........ .......... .......... ...C....VL L..AA..R.R .......... .......... ...V...A.. .........

Ursus_thibetanus F.....A... CL........ .......... .......... ...C....VL L..AA..R.R .......... .......... ...V...A.. .........

Zalophus_californianus ......A... CL........ .......... .......... .........L L..MA....R .......... .......... ...V...... .........

Artibeus_jamaicensis ...V..A... CL...V.... .....A.... P.L....... .........L H..LA..R.. ...RN..... .......... ...V...... .........

Desmodus_rotundus ...V..A... CL...V.... ..N..AV... P.L..V..V. .........L H...A..R.. ...H...... .......... ...V...... .........

Hipposideros_armiger ......A... SL........ .......... P......... ...V.....L H..MA..R.. .......... .......... ...V...... .........

Miniopterus_natalensis ......A... CL........ .......... .......... ....V...VL H...A..R.R ...Q...... .......... ...V...... .........

Molossus_molossus ...V..A... CLY....... ......V... .......... .........L H...A..R.R ...H...... .......... ...V...... .........

Myotis_myotis ......A... CV........ .HG....... ........T. ..CC.....L H...A..R.. ..HH...... .......... ...V...... .....N...

Phyllostomus_discolor ...V..A... SV...V.... ..N..A.... ..L....... .........L H..LA..R.R ...HS..... .......... ...V...... .....Q...

Pipistrellus_kuhlii ......A... CL........ .......... .......TLV ......V..L H...A..R.. ...Q...... .......... ...V...... .........

Rousettus_aegyptiacus ......A... SR...V.... .......... .......... .........L H..LA..R.. ....S...L. .......... ...V...... .........

Sturnira_hondurensis ...V..A... CL...V.... R....A.... P.L......V .........L H..LA..R.. ...H...... .......... ...V...... .....Q...

Talpa_occidentalis ......A... CL........ R......... ..A....... ...GV.V.TL R..LA..R.R ...HS...L. .......... ...V...... .........

Ceratotherium_simum ......A... CL........ .......... .......... ......L..V H...A..R.. ........L. .......... ...V...... .........

Equus_caballus ......A... CL........ .......... .......... .......... H..LA..R.. .......... .......... ...V...... .........

Manis_pentadactyla ..I...A... CL........ ..G....... .......... ...C...H.L H...A..R.. ........L. .......... ...V...... .........

Galeopterus_variegatus ...V..A... CL...V.... ..N....... .......... ......V..L LL..A..R.. ..H.....L. .......... ...V...I.. .........

Callithrix_jacchus ......A... CL........ .......... .......... ..L.....VV HL..A..R.R ..HH...... .......... ...V...... .......H.

Cebus_capucinus ......A... CL........ .......... .......... ..L.....VV HL..A..R.R ..HH...... .......... ...V...... .........

Cercocebus_atys ..I......R CL........ ..N..D.... .......... .........V HL..A..R.R ..HH...... .......... ...V...... .........

Chlorocebus_sabaeus ..I...A..R CL...V.... R......... .......... ......V..V HL..A..R.R ..HH...... .......... ...V...... .........

Colobus_angolensis ..I....... CL........ ..K....... P......... .........V HL..A..R.R ..HH...... .......... ...V...... .........

Gorilla_gorilla ...V..A... CL...V.... .......... .......... ......V... HL..A..R.R ..HH...... .......... ...V...... .........

Homo_sapiens ...V..A... CL...V.... .......... .......... ......V..V .L..A..R.R ..HH...... .......... ...V...... .........

Hylobates_moloch ..I...A... CL...V.... .......... .......... ......V..V HL..A..R.R ..HH....L. .......... ...V...... .........

Macaca_fascicularis ..I......R CL........ .......... .......... .........V HL..A..R.R E.HH...... .......... ...V...... .........

Macaca_mulatta ..I......R CL........ .......... .......... .........V HL..A..R.R E.HH...... .......... ...V...... .........

Macaca_nemestrina ..I......R CL........ .......... .......... .........V HL..A..R.R E.HH..C... .......... ...V...I.. .........

Mandrillus_leucophaeus ..I......R CL........ ..N....... .......... .........V HL..A..R.R ..HH...... .......... ...V...... .........

Microcebus_murinus .......... CL...V.... R......... .......... ......V... RL..A..HSR ..H.A..... .......... ...V...... .........

Nomascus_leucogenys ..I...A... CL........ .......... .......... ......V..V HL..A..R.R ..HH....L. .......... ...V...... .........

Otolemur_garnettii ......A..R CL........ .......... .......... ...V..V... HL..A....R ..H....... .......... ...V...... .........

Pan_paniscus ...V..A... CL...V.... .......... .......... ......V..V HL..A..R.R ..HH...... .......... ...V...... .........

Pan_troglodytes ...V..A... CL...V.... .......... .......... ......V..V HL..A..R.R ..HH...... .......... ...V...... .........

Papio_anubis ..I......R CL........ ..N..D.... .......... .........V HL..A..R.R ..HH...... .......... ...V...... .........

Piliocolobus_tephrosceles ..I......R CL...F.... ..R....... P......... .........V HL..A..R.R ..HH...... .......... ...V...I.. .........

Pongo_abelii ...V..A... CL...V.... .......... P......... ......V..V HL..A..R.R ..HH...... .......... ...V...... .........

Propithecus_coquereli ......A... CL...V.... .......... .....I..V. ......V... RL..A..RSR ..H.A..... .......... ...V...... .........

Rhinopithecus_bieti ..I....... CL...N.... ..K....... P.S....... ......V..V HL..A..R.Q ..HH...... .......... ...V...... .........

Rhinopithecus_roxellana ..I....... CL...N.... ..K....... P.S....... ......V..V HL..A..R.Q ..HH...... .......... ...V...... .........

Saimiri_boliviensis ......A... CL........ .......... .......... ..L.....VV HL..A..R.R ..HH...... .......... ...V...... .........

Sapajus_apella ......A... CL........ .......... .......... ..L.....VV HL..A..R.R ..HH...... .......... ...V...... .........

Theropithecus_gelada ..I......R CL........ ..N..D.... .......... .........V HL..A..R.R ..HH...... .......... ...V...... .........

Trachypithecus_francoisi ..I....... CL...N.... ..K....... P......... ......V..V HL.MA..R.R ..HH...... .......... ...V...... .........

Ochotona_princeps ......A... CL...V.... N......... ..V..I.... ......V.VV CL..A..R.. ..HH...... .......... ...V...... .....Q...

Arvicanthis_niloticus .......... CL...V.... .......... ..I....... ......VH.. HL..A..G.. ..HP...... ..R....... ...V...... .........

Arvicola_amphibius .......... CL...V.... .......... ..I....... ......V..V HL..A..R.R ..H.A..... ..R....... ...V...... .........

Castor_canadensis ......AT.. CL...V.... .......... ..I....... ...C..V... LL..V..R.R ..H.A..... .......... ...V...... .....Q...

Cavia_porcellus .......... CL........ ..N....... ..V....... ...C..V..V HL..A..R.R ..H.A..... ..R....... ...V...... .....Q...

Chinchilla_lanigera .......... CL........ .......... ..V....... ...Y..VH.. HL..A..R.R ..H.A..... .......... ...V...... .....Q...

Cricetulus_griseus ...V...... CL...V.... .......... ..I....... ......V... HL..A..R.R ..H.A..... ..R....... ...V...... .........

Dipodomys_ordii .......... GL...V.... .......... ..A....... ...C..V..V HL..A..R.R ..H.G..... ..R.....V. ...V..M... ..P..Q...

Fukomys_damarensis .......... CL........ ..N....... ..V....... ...C..V..V HL..A..R.R ..H.A..... ..R....... ...V...... .....Q...

Grammomys_surdaster .......... CL...V.... .......... ..I....... ......VH.. HL..A..R.. ..H.A..... ..R....... ...V...... .........

Heterocephalus_glaber .......... CL........ .......... ..V....... ...C..V... HL..A..R.R ..H....... ..R....... ...V...... .....Q...

Ictidomys_tridecemlineatus ..I...A... CL........ ..T...V... ..A....... ..S...V.VV HL.IT....R ..H.A..... ..R....... ...V...... .....Q...

Jaculus_jaculus ...V...... CL...V.... .......... ..I....... ......V.V. HL..A..R.. ..H.A..... ..R....... ...V...... .....Q...

Marmota_flaviventris ..I...A.A. CL........ ..T...V... ..A....... ..S...V.VV HL.IT....R ..H.A..... ..R....... ...V...... .....Q...

Marmota_marmota ..I...A... CL........ ..T...V... ..A....... ..S...V.VV HL.IT....R ..H.A..... ..R....... ...V...... .....Q...

Mastomys_coucha ...T...... CL...V.... .......... ..I....... ......V... HL..A..R.. L.H.A..... ..R....... ...V...... .........

Meriones_unguiculatus .......... CL........ .......... ..I....... ......V..L HL..A....R ..H.G...C. ..R....... ...V...... .........

Mesocricetus_auratus ...V...... CL...V.... T......... ..I....... ......VHV. HL..A..R.R ..H.A..... ..R....... ...V...... .........

Microtus_ochrogaster .......... CL...V.... .......... ..I....... ......VH.. HL..A..R.R ..H.A..... ..R....... ...V..M... .........

Mus_caroli .......... CR...V.... .......... ..I.....L. ......VH.. HL..A..R.. ..H.A..... ..R....... ...V...... .........

Mus_musculus .......... CL...V.... .......... ..I.....L. ......VH.. HL..A..R.. ..H.A..... ..R....... ...V...... .........

Mus_pahari .......... CL...V.... .......... ..I..L.... ......VH.. HL..T..R.R ..H.A..... ..R....... ...V...... .........

Mus_spicilegus .......... CL...V.... .......... ..I.....L. ......VH.. HL..A..R.. ..H.A..... ..R....... ...V...... .........

Nannospalax_galili .......... CL...V.... .......... ..V....... ......V... RL..T..R.. ..H.A..... ..R....... ...V...... .........

Octodon_degus .......... CL........ T......... ..V....... ...T..V... HL..A..R.R ..H.A..... .......... ...V...... .....Q.K.

Onychomys_torridus .......... CL...V.... .......... ..I....... ......V... NL..A..R.. ..H.A..... ..R....... ...V...... L........

Peromyscus_leucopus .......... CL...V.... .......... .......... V.....V... HL..A..R.. ..H.A..... ..R....... ...V...... .........

Peromyscus_maniculatus .......A.R CL...V.... .......... ..I....... ......V... HL..A..R.. ..H.A..... ..R....... ...V...... .........

Rattus_norvegicus .......... CL...V.... .......... ..I..L.... ......V... HL..A..R.. ..H.A..... ..R....... ...V...... .........

Rattus_rattus .......... CL...V.... .......... ..I..L.... ......V... HL..A..R.. ..H.A..... ..R....... ...V...I.. .........

Urocitellus_parryii ..I...A... CL........ ..T...V... ..A....... ..SVV.V.VV HL.IT....R .VH.A..... ..R....... ...V...... .....Q...

7.50 8.50

>>> TM7 **$** <<< **$**

P PP P P G GG

Monodelphis_domestica TLRSIKLFFD LFLSSFQGML VAILYCFVNK EVQGELLKSW HRWQLGRALE EEHRH---TC SHMSTGRPG- STCPGEKRQL VSCSSTSNGA GCSQANALN

Phascolarctos_cinereus ....V..... .......... ..V....... ...A..M... .......... .....---.. ..L......- .A........ .......... .S......T

Sarcophilus_harrisii .......... .......... .......... ...A....A. R......... .....---.. ....S....- .......... .......... .G......T

Vombatus_ursinus ....V..... .......... .......... ...A..M... .......... .....---M. ..L......- .......... .......... .S..S...T

Echinops_telfairi ....A..... ........L. .......L.. ...A...RR. Q..RV.KV.Q ....S----G ..TAP--.TA RGP.S..LL. PRS.G-N..T S--------

Elephantulus_edwardii A...T..... ..F..I..L. ..V....L.. ...S...RR. ...RVDTG.R ..L.L----G .RSAP--.S- HGP.S.E.L. T.TC--.E.P R--------

Orycteropus_afer A...A..... .L......L. ..V....L.. ...S..MRR. ...RV.K..R ..RQP----G ..TAP--.-A HEP.R..LL. .RGGG-.D.G .--------

Trichechus_manatus ...F...... ........L. ..V....L.. ...A..MRR. Y..RVDK..Q K..QL----G ..AAQ--.-G HGS.S..LL. RRSGG-.D.. .--------

Balaenoptera_acutorostrata ....A..... ........L. ..V....L.. ...S...RR. ...RE.K..Q ..RHV----G ..TA--..T- GGP.S..LL. SRGGG-..RT S--------

Balaenoptera_musculus ....A..... ..F.....L. ..V....L.. ...S...RR. ...RE.K..Q ..RHV----S ..TA--..T- GGP.S..LL. SRGGG-..RT S--------

Delphinapterus_leucas ....A..... ........L. ..V....L.. ...S...RR. ...RE.K..Q ..CHV----G ..TA--..T- GGP.S..LL. SRGGG-..RT S--------

Globicephala_melas ....A..... ........L. ..V....L.. ...S...RR. ...RE.K..Q ..CHV----G ..TA--..T- GGP.S..LL. SRG.G-..RT S--------

Lagenorhynchus_obliquidens ....A..... ........L. ..V....L.. ...S...RR. ...RE.K..Q ..CHV----G ..TA--..T- GGP.S..LL. SRG.G-..RT S--------

Lipotes_vexillifer ....A..... ........L. ..V....L.. ...S...RR. ...RE.K..Q ..C.V----G ..TA--..T- GGP.S..LL. SRGGG-..RT S--------

Monodon_monoceros ....A..... ........L. ..V....L.. ...S...RR. ...RE.K..Q ..CHV----G ..TA--..T- GGP.S..LL. SRGGG-..RT S--------

Neophocaena_asiaeorientalis ....A..... ........L. ..V....L.. ...S...RR. ...RE.K..Q ..CHV----G ..TA--..T- GGP.S..LL. SRGGG-..RT S--------

Orcinus_orca ....A..... ........L. ..V....L.. ...S...RR. ...RE.K..Q ..CHV----G ..TA--..T- GGP.S..LL. SRG.G-..RT S--------

Phocoena_sinus ....A..... ........L. ..V....L.. ...S...RR. ...RE.K..Q ..CHV----G ..TA--..T- GGP.S..LL. SRGGG-..RT S--------

Physeter_catodon ....A..... ........L. .......L.. ...S...RR. ...RE.K..Q ..RHV----R ..TA--..T- GGP....LL. SRGGG-..RT S--------

Bos_indicus ....A..... ........L. .......L.. ...S...RH. ...RE.K..Q ..RHT----G ..TT--..L- RGP.SK.LL. ARG..-...T .--------

Bos_mutus ....A..... ........L. .......L.. ...S...RR. ...RE.K..Q ..RHA----G ..TT--..L- RGP.SK.LL. ARG..-...T .--------

Bos_taurus ....A..... ........L. .......L.. ...S...RH. ...RE.K..Q ..RHT----G ..TT--..L- RGP.SK.LL. ARG..-...T .--------

Bubalus_bubalis ....A..... ........L. .......L.. ...S...RR. ...RE.K..Q ..RHA----G ..TT--..L- RGP.SK.LL. ARG..-...T .--------

Camelus_dromedarius A...T..... ........L. ..V....L.. ...S...RR. ...HE.K..H N.RHMG---G G..A--Q.A- .GP.SQ.LL. SRGGGS...T S--------

Camelus_ferus ....T..... ........L. ..V....L.. ...S...RR. ...RE.K..H N.RHMG---G G..A--Q.A- RGP.SQ.LL. SRGGGS...T S--------

Capra_hircus ....A..... ........L. .......L.. ...S...RR. ...RE.K..Q ..RHA----G ..TT--..L- RGP.S..LL. ARG.G-...T S--------

Cervus_hanglu ....A..... ........L. ..V....L.. ...S...RR. ...RE.K..Q ..RHA----G ...T--..L- RGP.S..LL. SRG.G-...T S--------

Moschus_moschiferus ....A..... ........L. ..V....L.. ...S...RR. .H.RE.K..Q ..RNT----G ..TT--..L- RGPSSK.LL. SRG.G-...T R--------

Odocoileus_virginianus ....A..... ........L. ..V....L.. ...S...RR. ...RE.K..Q ..RHA----G ..TT--..L- RGPLS..LLH SRG.G-.... S--------

Ovis_aries ....A..... ........L. .......L.. ...S...RR. ...RE.K..Q ..RHA----G ...T--..L- GGP.S..LL. ARG.G-...T S--------

Sus_scrofa A...A..... ........L. ..V....L.. ...A...RR. ...RE.K..Q KA..V----G ..SA--..P- .GP.S..LL. STGG.-...T S--------

Acinonyx_jubatus ....A..... ........L. ..V....L.. ...S..RRR. ...HV.NS.R .QRHV----H RPPASA..R- GRP.SA.LL. SGDGGG.... .--------

Callorhinus_ursinus ....A..... ........L. ..V....L.. ...S..RRC. ...RA.TS.R .QRHV----S RPVASA..P- GGP.SKQPL. SGGGGS.... .--------

Canis_lupus ....A..... ........L. ..V....L.. ...S...RR. Q..RM.KR.R ..RPP----S R.PDP...A- GGA.SK.LL. SRGGD---.. A--------

Enhydra_lutris ....A..... ........L. ..V....L.. ...S...RR. N..RA..S.Q ..RHT----G RLGPPV..T- GDP.S..PL. SRSGGS...T .--------

Eumetopias_jubatus ....A..... ........L. ..V....L.. ...S..RRC. ...RA.TS.R .QRHV----S RPAASA..P- GGP.S.QPL. S-------.. .--------

Felis_catus ....A..... ........L. ..V....L.. ...S...RR. ...HV.NS.R .QRHV----H RPPASA..R- GRP.SA.LL. SGGGGG.... .--------

Halichoerus_grypus ....A..... ........L. ..V....L.. ...S..RR.. ..RRA.AS.R .RRHA----- ---------- .GP.S..PL. CGAGG-.... .--------

Leptonychotes_weddellii ....A..... ........L. ..V....L.. ...S..RRC. ...RA.AS.R .RRPV----S GP------P- GGP....PL. CGGGG-.... A--------

Lontra_canadensis ....A..... ........L. ..V....L.. ...S...RR. N..RA..S.Q .ARHT----S RLGPPV.TT- GDP.S..PL. SRSRGS...T .--------

Lynx_canadensis ....A..... ........L. ..V....L.. ...S...RR. ...HV.NS.R .QRHV----H RPPASA..R- GRP.SA.LL. SGGGGG.... .--------

Mirounga_leonina ....A..... ........L. ..V....L.. ...S..RRC. ...RA.AS.R .RRPV----- ---------- GGP....PL. CGGRG-.... A--------

Mustela_erminea ....A..... ........L. ..V....L.. ...S...RR. N..RA..S.R ..RHP----S RLGPPA..T- GDP.S..PL. SRSGGS..ET .--------

Neovison_vison ....A..... ........L. ..V....L.. ...S...RR. N..RA..L.Q ..RHS----S RLGPPA..T- GDP.S..PL. SRSGGS...T .--------

Odobenus_rosmarus I...V..... ........L. ..V....L.. ...S..RRC. ...HA.TS.R .Q.HV----S RPAASA.QP- GGP.S..PL. SGGGGGG... C--------

Phoca_vitulina ....A..... ........L. ..V....L.. ...S..RRC. ..RRA.AS.R .RRHA----- ---------- .GP.S..PL. CGAGG-.... .--------

Suricata_suricatta ....A..... ........L. ..L....L.. ...S.V.RR. ...HV.NS.R VQRHV----R GPSASA...- GAP.S..PL. SRAEGG.... S--------

Ursus_americanus ....A..... ........L. ..V....L.. ...S...R.. R..RA.NS.Q .GR.T----S G.TASA..T- GGP.SQ.LL. SRGGGS.... .--------

Ursus_arctos ....A..... ........L. ..V....L.. ...S...R.. R..RA.NS.Q .GR.T----S G.TASA..T- GGP.SQ.LL. SRGGGS.... .--------

Ursus_thibetanus ....A..... ........L. ..V....L.. ...S...R.. R..RA.NL.Q .GR.T----S G.TASA..T- .GP.SQ.LL. SRGGGS.... .--------

Zalophus_californianus ....A..... ........L. ..V....L.. ...S..RRC. ...RA.TS.R .QRHV----S RPAASA..P- GGP.S.QPL. SGGGGG.... .--------

Artibeus_jamaicensis A..FA..... ........L. ..V....L.. ...A...R.. R..RV.K..R ..Q.G----G GRPASA..T- .GGATQ.LLF SRGRA-.S.. S--------

Desmodus_rotundus ...FA..... ........L. ..V....L.. ...A...R.. ..RH..E.RW ...QGS---S ..PASA..T- .GAAT..LL. SRGRA-.S.. S--------

Hipposideros_armiger ...FA..... ........L. ..V....L.. ...A..RRC. ...RV.K..Q VGRHV----S ..RASAL.T- DGP.S..LL. SRG.G-.... S--------

Miniopterus_natalensis ...FA..... ........L. ..V....L.. ...A...R.. ...RMDEV.R ...QG----S ..RALD..T- .----..LL. SRGHD-.... S--------

Molossus_molossus ....A..... ........L. ..V....L.. ...A...RN. ...RG.E..Q DGRQG----G .RSALAQTT- .GA.T..LL. SRGRD-.... S--------

Myotis_myotis ...FV..... ........L. ..V....L.. ...T...R.. ...R..EL.R ..RQV----S G.PALA.ST- .GA.T..LL. SGGCD-...G SH-------

Phyllostomus_discolor A..FA..... ........L. ..V....L.. ...A...R.. R..RV.E..R G.TQG----G G.PASA..T- .GAAT..LL. SRGRA-GS.T S--------

Pipistrellus_kuhlii ...FA..... ........L. ..V....L.. ...A...R.. R..RA.EG.R ..RPG----S .RPAPA..S- .GA.T..LL. TRGPD-.... P--------

Rousettus_aegyptiacus ....A..... ........L. ..V....L.. ...A..RRR. L..RM....R .RQ-----AG .YAA--..P- RGPSS..LP. S-GG.----- ---------

Sturnira_hondurensis A..FV..... ........L. ..V....L.. ...A...R.. R..HV.T..R ...QG----G R.PASA.AT- .GAAT.RPL. SRGRA-GS.. S--------

Talpa_occidentalis A...A..... ........L. .......L.. ...S...RR. ..RR.....R ..RPV----- .LPASA..P- GSP..K.LL. SGGGG--Q.. R--------

Ceratotherium_simum ....A..... ........L. ..V....L.. ...S...RR. ...RT.K..Q ..CHI----G ..T.SA..A- GGT.S..LL. SRGG.-..R. S--------

Equus_caballus ....A..... ........L. ..V....L.. ...S...RC. ...RV.K..Q ..CHM----G ..TTAA..A- GST.R..LL. SRGGG-.... S--------

Manis_pentadactyla ....A..... ........L. ..V....L.. ...S...RC. .H.RG.K..Q K-----CPVG ..AASAQ.T- GGPCS.RLL. SIGGA-..R. S--------

Galeopterus_variegatus ....A..... ........L. ..V....L.. ...T...RC. ...RV.K..R ..QLA----S ..AAPAG..- HGP.S..L.. .RGG.-.... .--------

Callithrix_jacchus ....A..... ........L. ..V....L.. ...S..WRR. .H.R..KV.R ..QNT----S ..KASSA.S- FSS.SK.LRF G.GRG-.H.. .--------

Cebus_capucinus ....A..... ........L. ..V....L.. ...S..RQR. ...R..K..Q ..QNT----S ..KASSA..- LSS.SR.LRF G.SGG-.H.. .--------

Cercocebus_atys ...FA..... ........L. ..V....L.. ...S..RRH. ...R..KV.Q ..RGT----S N.KAPSA..- QGL..K.L.S GRGGG-.--- ---------

Chlorocebus_sabaeus ...FA..... ........L. ..V....L.. ...S..RRH. ...R..KE.Q ..RGT----S N.KAPSA..- RGL..K.L.S GRGGG-.--- ---------

Colobus_angolensis ...FA....E ........L. ..V....L.. ...S..RRH. ...R..KV.Q ..RST----S N.KAPSA..- RGL..K.L.S GRGGG-.--- ---------

Gorilla_gorilla ....A..... ........L. ..V....L.. ...S..RRR. ...R..KV.W ..RNT----S N.KASSS..- RGL..KEL.F GRGGG-.--- ---------

Homo_sapiens ....A..... ........L. ..V....L.. ...S..RRR. ...R..KV.W ..RNT----S N.RASSS..- HGP.SKEL.F GRGGG-.--- ---------

Hylobates_moloch ...TA..... ........L. ..V....L.. ...S..RRR. .H.R..KV.R ..RST----S N.KASSSS.- RGL.SKEL.F GRGDG-.--- ---------

Macaca_fascicularis ...FA..... ........L. ..V....L.. ...S..RRH. ...R..KV.Q ..RGT----S N.KAPSA..- QGL..K.L.S GRDGG-.--- ---------

Macaca_mulatta ...FA..... ........L. ..V....L.. ...S..RRH. ...R..KV.Q ..RGT----S N.KAPSA..- QGLS.K.L.S GRDGG-.--- ---------

Macaca_nemestrina ...FA..... ........L. ..V....L.. ...S..RRH. ...R..KV.Q ..RGT----S N.KAPSA..- QGL..K.L.S GRGGG-.--- ---------

Mandrillus_leucophaeus ...FA..... ........L. ..V....L.. ...S..RQH. ...R..KV.Q ..RGT----S N.KAPSA..- QGL..K.L.S GRGGG-.--- ---------

Microcebus_murinus ....A..... ........L. ..V....L.. ...L..QRR. ...R..K..P G.R.T----G ..SASAG..- HGN.T.NLR. .TGG.-...V .--------

Nomascus_leucogenys ...TA..... ........L. ..V....L.. ...S..RRR. .H.R..KV.R ..RST----S N.KASSSS.- RGL.SKEL.F GRGDG-.--- ---------

Otolemur_garnettii ....A..... ........L. ..V....L.. ...S..RRY. R..L..K..R Q.L.T----S ..VASTG.S- HGI.C..L.. .RGG.-.... .--------

Pan_paniscus ....A..... ........L. ..V....L.. ...S..RRR. ...R..KV.W ..QNS----S N.KASSS..- RGP.SKEL.F GRGGG-.--- ---------

Pan_troglodytes ....A..... ........L. ..V....L.. ...S..RRR. ...R..KV.W ..QNS----S N.KASSL..- RGP.SKEL.F GRGGG-.--- ---------

Papio_anubis ...FA..... ........L. ..V....L.. ...S..RRH. ...R..KV.Q ..RGT----S N.KAPSA..- QGL..K.L.S GRGGG-.--- ---------

Piliocolobus_tephrosceles ...FA..... ........L. ..V....L.. ...S..RRH. ...R..KV.Q ..RST----S N.KAPSA..- RGL..K.L.S GRGGG-.--- ---------

Pongo_abelii ....A..... ........L. ..V....L.. ...S..RRR. ...R..KV.Q ..RNT----S N.KASSS..- RSL.SKEL.F GRAGG-.--- ---------

Propithecus_coquereli ....A..L.. ........L. ..V....L.. ...S..QRR. ...R..K..R R.Q.T----G ..TASAG.A- -----KNL.. .RDG.-...I .--------

Rhinopithecus_bieti ...FA..... ........L. ..V....L.. ...S..RRH. ...R..KV.Q ..RST----S N.KAPSA..- RGL..K.L.S GRGGG-.--- ---------

Rhinopithecus_roxellana ...FA..... ........L. ..V....L.. ...S..RRH. ...R..KV.Q ..RST----S N.KAPSA..- RGL..K.L.S GRGGG-.--- ---------

Saimiri_boliviensis ....A..... ........L. ..V....L.. ...S..RRR. ...R..KV.Q ..QNT----S G.RASSV..- LSP.SK.LRF G.GGG-.H.. .--------

Sapajus_apella ....A..... ........L. ..V....L.. ...S..RRR. ...R..K..Q ..QNT----S ..KASSA..- LSS.SR.LRF G.SGG-.H.. .--------

Theropithecus_gelada ...FA..... ........L. ..V....L.. ...S..RRH. ...R..KV.Q ..RGT----S N.KAPSA..- QGL..K.L.S GRGGG-.--- ---------

Trachypithecus_francoisi ...FA..... ........L. ..V....L.. ...S..RRH. ...R..KV.Q ..RST----S N.KAPSA..- RGL..K.L.S GRGGG-.--- ---------

Ochotona_princeps A...T..... ........L. ..V....L.. ...L...RR. R..H..K..R ..RSA----T M.IPSAG.-- -----..L.. AQAGG-.SR. .--------

Arvicanthis_niloticus ....T..... ........L. ..L....L.. ...A...RR. RL..E.K..P ..RMASG-HG ...APAG.C- HGD.C..L.. M.AG.-.R.T .--------

Arvicola_amphibius A......... ..F.....L. ..V....L.. ...A...RR. R..RE.K..K ..QMASC-RG N.TAPAGSS- HGE.S..L.. ..A..-C..- ---------

Castor_canadensis ....T..... ........L. ..V....L.. ...A...RR. RH.RE....W ..QLT----N N..APAG.S- RGVHCQ.L.. MRSG.-.R-. .--------

Cavia_porcellus ...LA..... ........L. ..V....L.. ...A..WRR. QC.YQ.K..L D.RIT----G ..LAPTG.S- HSPLC..L.. .KGA.-.... .--------

Chinchilla_lanigera ....A..... ........L. ..V....L.. ...A..WQR. QH.RM.K..L D.RVT----G ..SAPTGLT- RGPLS..L.. .KGV.-.S.. S--------

Cricetulus_griseus ....T..... ........L. ..V....L.. ...A.V.GR. R...E.K..K ..QKASS-HG ..VAPAGSS- HDD.C..L.. ..AG.-...T .--------

Dipodomys_ordii ....T..... ........L. ..V....LS. ...A..RRR. R.RRE..V.R ..RLS----G ..A.------ ---.CQ.L.. LRDG.-GL.. .--------

Fukomys_damarensis ....A..... ........L. ..V....L.. ...A..WRR. W..RE.K..L D.RIT----G ..SAPT..S- HSPLC..L.. .KEG.-.K.. S--------

Grammomys_surdaster ....T..... ........L. ..V....L.. ...A...RR. RQ..E.K..Q ..RMASS-HG ...ARAG.C- YGD.C..L.. M.AG.-.R.T .--------

Heterocephalus_glaber ....A..... ........L. ..V....L.. ...A..WRR. R..HE.K..L D.RIT----G ..SAPTG.S- HSPLCKNL.. LK-GD-.... S--------

Ictidomys_tridecemlineatus A...A..... ........L. ..V....LS. ...A...QR. R..HE....Q DPR.S----S .PSAPA..C- RGPSF.RLR. .KGG.-.... .--------

Jaculus_jaculus ....A..... ........L. ..V....L.. ...A...RR. R..RE.K..R .VRLSGS-HG ..LAPSGLS- QSA.C.SL.. .GKG.-...T .--------

Marmota_flaviventris A...A..... ........L. ..V....LS. ...A...QR. R..HE....Q DPR.S----S .PSAPA..C- RGPSC..LR. .KGG.-.... .--------

Marmota_marmota A...A..... ........L. ..V....LS. ...A...QR. R..HE....Q DPR.S----S .PSAPA..C- RGPSC..LR. .KGG.-.... .--------

Mastomys_coucha ....T..... ........L. ..V....L.. ...A...RR. RQ..E.K..Q ..RMASS-HG R..APAGSC- HGD.C..F.. M.AG.-.S.T .--------

Meriones_unguiculatus ....T..... ........L. ..V....L.. ...A...RR. R..RT.K..Q ..QLASS-HG ...APAG.R- RGD.C.RL.. ..AGG-..DT .--------

Mesocricetus_auratus ....A..... ........L. ..V....L.R ...A...RR. R..RE.K..K ..QMASS-HG ..VAPAGSR- HGD.C..L.. ..AG.-...T .--------

Microtus_ochrogaster A...T..... ..F.....L. ..V....L.. ...A...RR. R..RE.K..K ..QMASC-RG N.TAPAGSS- HGE....L.. .TAG.-C..T ---------

Mus_caroli ....T..... ........L. ..V....L.. ...A..MRR. RQ..E.K..Q ..RLASS-HG ..TAPAG.C- HGD.C..L.. M.AG.-.S.T .--------

Mus_musculus ....T..... ........L. ..V....L.. ...A..MRR. RQ..E.K..Q ..RLASS-HG ...APAG.C- HGD.C..L.. M.AG.-.S.T .--------

Mus_pahari ....T..... ........L. ..V....L.. ...A...RR. RQ..E.K..Q ..RLASS-HG ...APAG.C- HGD.C..LR. T.AG.-.S.T .--------

Mus_spicilegus ....T..... ........L. ..V....L.. ...A..MRR. RQ..E.K..Q ..RLASS-HG ...APAG.C- HGD.C..L.. M.AG.-.S.T .--------

Nannospalax_galili ....A..... ........L. ..V....L.. ...A...RR. SL.RE.K..R ..QLASS-QG ..NAPAGAS- RGA.C..L.F ..MG.-...T .--------

Octodon_degus ....V..... ........L. ..V....L.. ...A..WRH. R..RE.K..L D.RIT----S ..SAPTG.S- RGPLC..L.. .KGG.-.... S--------

Onychomys_torridus ....T..... ........L. ..V....L.. ...A...RR. R..RE.K..K D.QMAIS-HG ...APAGSS- PGD.S..LE. ..AG.-...T .--------

Peromyscus_leucopus ....T..... ........L. ..V....L.. ...A...RR. R..RE.K..K D.QMAIS-HG ...APAGSSR RDD....L.. ..AG.-...T .--------

Peromyscus_maniculatus ....T..... ........L. ..V....L.. ...A...RR. R..RE.K..K D.QTAIS-HG ...APAGSSR RDD....L.. ..AG.-...T .--------

Rattus_norvegicus ....T..... ..F.....L. ..V....L.. ...A...RR. R...E.K..Q ..RMASS-HG ...APAGTC- HGD.C..L.. M.AG.-.S.T .--------

Rattus_rattus ....T..... ..F.....L. ..V....L.. ...A...RR. R...E.K..Q ..RMASS-HG ...APAGTC- HGD.CQ.L.. M.AG.-.S.T .--------

Urocitellus_parryii A...A..... ........L. ..V....LS. ...A...QR. R..HE....Q DPR.S----S .PSAPA..C- RGPSF..L.. .KGG.-.... .--------

Monodelphis_domestica RNSYLEKTEA SPGDPGTEGE GTHCYTFPGV AESNF----- ---------- ---------- ---------- ---------- ---------- --

Phascolarctos_cinereus QS......GP ..S..D...K ---------- .....----- ---------- ---------- ---------- ---------- ---------- --

Sarcophilus_harrisii QS......G. .SC..D...K ..---....L .....----- ---------- ---------- ---------- ---------- ---------- --

Vombatus_ursinus QS......G. ..S......K .I.R...S.. .....----- ---------- ---------- ---------- ---------- ---------- --

Echinops_telfairi ---------- --P..SS.TP SA.--GL.AL ...P.----- ---------- ---------- ---------- ---------- ---------- --

Elephantulus_edwardii ---------- --L..SL.TP SA.--SL..- P..P.WSLGT PL-------- ---------- ---------- ------G--- ---------- --

Orycteropus_afer ---------- --P..SS.TP SA.--GLC-- ---------- ---------- ---------- ---------- ---------- ---------- --

Trichechus_manatus ---------- --P..PS.TP SA.--GL.RL ..RP.----- ---------- ---------- ---------- ---------- ---------- --

Balaenoptera_acutorostrata ---------- --Q..SA.TH LAG--SL..L ..NP.----- ---------- ---------- ---------- ---------- ---------- --

Balaenoptera_musculus ---------- --Q..SA.TH LAG--GL..L ..NP.----- ---------- ---------- ---------- ---------- ---------- --

Delphinapterus_leucas ---------- --Q..S..TH LAG--GL..L ..NP.----- ---------- ---------- ---------- ---------- ---------- --

Globicephala_melas ---------- --Q..S..TH LAG--GL..L ..NP.----- ---------- ---------- ---------- ---------- ---------- --

Lagenorhynchus_obliquidens ---------- --Q..S..TH LAG--GL..L ..NP.----- ---------- ---------- ---------- ---------- ---------- --

Lipotes_vexillifer ---------- --Q..SA.TH LAG--GL..L ..NP.----- ---------- ---------- ---------- ---------- ---------- --

Monodon_monoceros ---------- --Q..S..TH LAG--GL..L ..NP.----- ---------- ---------- ---------- ---------- ---------- --

Neophocaena_asiaeorientalis ---------- --Q..S..TH LAG--SL..L ..NP.----- ---------- ---------- ---------- ---------- ---------- --

Orcinus_orca ---------- --Q..S..TH LAG--GL..L ..NP.----- ---------- ---------- ---------- ---------- ---------- --

Phocoena_sinus ---------- --Q..S..TH LAG--SL..L ..NP.----- ---------- ---------- ---------- ---------- ---------- --

Physeter_catodon ---------- --Q..CA.TH WAG--GL..L ..DP.----- ---------- ---------- ---------- ---------- ---------- --

Bos_indicus ---------- --Q.ASA.T? LAG--GL..S ..NPQ----- ---------- ---------- ---------- ---------- ---------- --

Bos_mutus ---------- --Q.ASA.TR LAG--GLA.S ..NPQ----- ---------- ---------- ---------- ---------- ---------- --

Bos_taurus ---------- --Q.ASA.TC LAG--GL..S ..NPQ----- ---------- ---------- ---------- ---------- ---------- --

Bubalus_bubalis ---------- --Q..SA.TH LAG--GL..S ..NPQ----- ---------- ---------- ---------- ---------- ---------- --

Camelus_dromedarius ---------- --Q..SA.TC LAG--GL..L ...P.----- ---------- ---------- ---------- ---------- ---------- --

Camelus_ferus ---------- --Q..SA.TC LAG--GL..L ...P.----- ---------- ---------- ---------- ---------- ---------- --

Capra_hircus ---------- --Q..S..TR LAG--GL..S ..NPQ----- ---------- ---------- ---------- ---------- ---------- --

Cervus_hanglu ---------- --Q..SA.TR LAG--GL..S ..NPQ----- ---------- ---------- ---------- ---------- ---------- --

Moschus_moschiferus ---------- --Q..S..TR LAG--GL..S ..NPK----- ---------- ---------- ---------- ---------- ---------- --

Odocoileus_virginianus ---------- --Q..SA.TR LAG--GL..L ..NPQ----- ---------- ---------- ---------- ---------- ---------- --

Ovis_aries ---------- --Q..S..TH LAG--GL..S ..NPQ----- ---------- ---------- ---------- ---------- ---------- --

Sus_scrofa ---------- --QE.SA.TH LAS--GL... ..NP.----- ---------- ---------- ---------- ---------- ---------- --

Acinonyx_jubatus ---------- --R..SA.TC RVS--SL..L ...P.----- ---------- ---------- ---------- ---------- ---------- --

Callorhinus_ursinus ---------- --RG.SADPS LLG--SL.AL ...P.----- ---------- ---------- ---------- ---------- ---------- --

Canis_lupus ---------- --R..SADTG LVG--IP... .A.P.ESPQE PP-------- ---------- ---------- GAPVGAGLRC ---------- --

Enhydra_lutris ---------- --RV.SADPR LVS--SL..L ...P.----- ---------- ---------- ---------- ---------- ---------- --

Eumetopias_jubatus ---------- --RG.SADPS LLG--SL.AL ...P.----- ---------- ---------- ---------- ---------- ---------- --

Felis_catus ---------- --R..SA.TC RVS--SL..L ...P.----- ---------- ---------- ---------- ---------- ---------- --

Halichoerus_grypus ---------- --RG.PAHPS PVG--SR.AL ...P.----- ---------- ---------- ---------- ---------- ---------- --

Leptonychotes_weddellii ---------- --RG.SAHPN LVG--SL.AL ..NP.----- ---------- ---------- ---------- ---------- ---------- --

Lontra_canadensis ---------- --R..SADPC LVS--SL..L ...P.----- ---------- ---------- ---------- ---------- ---------- --

Lynx_canadensis ---------- --R..SA.TC RVS--SL..L ...P.----- ---------- ---------- ---------- ---------- ---------- --

Mirounga_leonina ---------- --RG.SAHPT LVG--SL.AL ..NP.----- ---------- ---------- ---------- ---------- ---------- --

Mustela_erminea ---------- --R..SADPC LVS--SL..L ...P.----- ---------- ---------- ---------- ---------- ---------- --

Neovison_vison ---------- --R..CADPC LVS--SL..L ...P.----- ---------- ---------- ---------- ---------- ---------- --

Odobenus_rosmarus ---------- --R..SADPS LLG--SL.AL ...P.----- ---------- ---------- ---------- ---------- ---------- --

Phoca_vitulina ---------- --RG.PAHPS PVG--SR.AL ...P.----- ---------- ---------- ---------- ---------- ---------- --

Suricata_suricatta ---------- --Q..SA.TC LVS--SL.AS ...PSKGPWA PSRGRTQH-- --------LP --DNPEPGPE GREPDSGPTA PLP----PSV LV

Ursus_americanus ---------- --R..SADSC LVG--SL..L ...P.----- ---------- ---------- ---------- ---------- ---------- --

Ursus_arctos ---------- --R..SADSC LVG--SL..L ...P.----- ---------- ---------- ---------- ---------- ---------- --

Ursus_thibetanus ---------- --R..SGDSC LVG--SL..L ...P.----- ---------- ---------- ---------- ---------- ---------- --

Zalophus_californianus ---------- --RG.SADPS LLG--SL.AL ...P.----- ---------- ---------- ---------- ---------- ---------- --

Artibeus_jamaicensis ---------- --Q..SAGTH AAI--SL..L .D.P.----- ---------- ---------- ---------- ---------- ---------- --

Desmodus_rotundus ---------- --Q..SAGTH AAS--SV..L ...P.----- ---------- ---------- ---------- ---------- ---------- --

Hipposideros_armiger ---------- --Q..LA.TR VAG--DLS.L ..CPL----- ---------- ---------- ---------- ---------- ---------- --

Miniopterus_natalensis ---------- --H..SA.TH VAG--SL..L .Q.SL----- ---------- ---------- ---------- ---------- ---------- --

Molossus_molossus ---------- --QG.SV.TC VAG--NL..L .Q.CL----- ---------- ---------- ---------- ---------- ---------- --

Myotis_myotis ---------- -GPS.SG.PP AAG--RP..W .Q.AL----- ---------- ---------- ---------- ---------- ---------- --

Phyllostomus_discolor ---------- --QG.SVGPL TAG--SL..P .D.P.----- ---------- ---------- ---------- ---------- ---------- --

Pipistrellus_kuhlii ---------- --H..AG.PP EAA--RL..W .Q.AL----- ---------- ---------- ---------- ---------- ---------- --

Rousettus_aegyptiacus ---------- --QN.S---P LDN--PE.QP LS.PA-SHVS PS-------- ---------- ---------- ------GAD- ---------- --

Sturnira_hondurensis ---------- --Q..SAGNS A.S--SL..L .D.P.----- ---------- ---------- ---------- ---------- ---------- --

Talpa_occidentalis ---------- --R..SSDTS PAG--HL..S ..CP.----- ---------- ---------- ---------- ---------- ---------- --

Ceratotherium_simum ---------- --Q..SA.TR LAS--GL..L ..RP.----- ---------- ---------- ---------- ---------- ---------- --

Equus_caballus ---------- --Q..SA.TH LAG--GL..L ..RP.----- ---------- ---------- ---------- ---------- ---------- --

Manis_pentadactyla ---------- --Q.RSW.TR LAD--SL..L D..PV----- ---------- ---------- ---------- ---------- ---------- --

Galeopterus_variegatus ---------- --Q.HAVKTP SAG--GL..L ...PS----- ---------- ---------- ---------- ---------- ---------- --

Callithrix_jacchus ---------- --Q.SSVATP LAG--GLSRL ...P.----- ---------- ---------- ---------- ---------- ---------- --

Cebus_capucinus ---------- --Q.SSVATP LAG--GLSRL ...P.----- ---------- ---------- ---------- ---------- ---------- --

Cercocebus_atys ---------- --Q.SSA.IP LAG--GL.RL ...P.STLLG PQLGL----- ---------- ---------- ----DSGT-- ---------- --

Chlorocebus_sabaeus ---------- --Q.TSA.IP LAG--DL.RL ...P.STPLG PQLGL----- ---------- ---------- ----NSGT-- ---------- --

Colobus_angolensis ---------- --Q.SSA.IP LAG--GL..L ..NPSEPCWD PS-------- ---------- ---------- ---------- ---------- --

Gorilla_gorilla ---------- --Q.SSA.TP LAG--GL.RL ...P.----- ---------- ---------- ---------- ---------- ---------- --

Homo_sapiens ---------- --Q.SSA.TP LAG--GL.RL ...P.----- ---------- ---------- ---------- ---------- ---------- --

Hylobates_moloch ---------- --Q.SSA.TP LAG--DL.RL ...P.----- ---------- ---------- ---------- ---------- ---------- --

Macaca_fascicularis ---------- --Q.SSA.IP LAG--GL.RL ...P.STLLG PQLGL----- ---------- ---------- ----DSGT-- ---------- --

Macaca_mulatta ---------- --Q.SSA.IP LAG--GL.RL ...P.STLLG PQLGL----- ---------- ---------- ----DSGT-- ---------- --

Macaca_nemestrina ---------- --Q.SSA.IP LAG--GL.RL ...P.STLLG PQLGL----- ---------- ---------- ----DSGT-- ---------- --

Mandrillus_leucophaeus ---------- --Q.SSA.IP LAG--GL.RL ...P.STLLG PQLGL----- ---------- ---------- ----DSGT-- ---------- --

Microcebus_murinus ---------- --R..SA.TP SAS--SL..L ..RPC----- ---------- ---------- ---------- ---------- ---------- --

Nomascus_leucogenys ---------- --Q.SSA.TP LAG--DL.RL ...P.----- ---------- ---------- ---------- ---------- ---------- --

Otolemur_garnettii ---------- --QN.SA.PP LAG--GL..L ...PSVSLAG AGLRFPERAS LDSPELDTLP RLEDPAVHLS HSVVVCGGSC PSPHLPCP-- --

Pan_paniscus ---------- --Q.SSA.TP LAG--GL..L ...P.----- ---------- ---------- ---------- ---------- ---------- --

Pan_troglodytes ---------- --Q.SSA.TP LAG--GL..L ...P.----- ---------- ---------- ---------- ---------- ---------- --

Papio_anubis ---------- --Q.SSA.IP LAG--GL.RL ...P.STLLG PQLGL----- ---------- ---------- ----DSGT-- ---------- --

Piliocolobus_tephrosceles ---------- --Q.SSA.IP LAG--GLSRL ..NPSEPCWD PS-------- ---------- ---------- ---------- ---------- --

Pongo_abelii ---------- --Q.SSA.TP LAG--GL.RL ...P.----- ---------- ---------- ---------- ---------- ---------- --

Propithecus_coquereli ---------- --Q..SA.TP LAS--GL..L .D.P.----- ---------- ---------- ---------- ---------- ---------- --

Rhinopithecus_bieti ---------- --Q.SSA.IP LAG--GLSRL T.NPSEPCWD PS-------- ---------- ---------- ---------- ---------- --

Rhinopithecus_roxellana ---------- --Q.SSA.IP LAG--GLSRL T.NPSEPCWD PS-------- ---------- ---------- ---------- ---------- --

Saimiri_boliviensis ---------- --Q.SSVATP LAG--GLSRL ...P.----- ---------- ---------- ---------- ---------- ---------- --

Sapajus_apella ---------- --Q.SSVATP LAG--GLSSL ...P.----- ---------- ---------- ---------- ---------- ---------- --

Theropithecus_gelada ---------- --Q.SSA.IP LAG--GL.RL ...P.STLLE PQLGL----- ---------- ---------- ----DSGT-- ---------- --

Trachypithecus_francoisi ---------- --Q.SSA.IP LAG--GLSRL T.NPSEPCWD PS-------- ---------- ---------- ---------- ---------- --

Ochotona_princeps ---------- --W.RSAKAL LAG--DL..L ..RP.----- ---------- ---------- ---------- ---------- ---------- --

Arvicanthis_niloticus ---------- --RE.SVGTS LAS--SL.RL .D.PT----- ---------- ---------- ---------- ---------- ---------- --

Arvicola_amphibius ---------- --------TS FAS--SLS.M TD.PT----- ---------- ---------- ---------- ---------- ---------- --

Castor_canadensis ---------- --Q.SSVQTP LAS--G...L ...PS----- ---------- ---------- ---------- ---------- ---------- --

Cavia_porcellus ---------- --Q..SA.TP LAS--GL..L .K.P.----- ---------- ---------- ---------- ---------- ---------- --

Chinchilla_lanigera ---------- --Q..SA.TP LAS--GLL-- ---------- ---------- ---------- ---------- ---------- ---------- --

Cricetulus_griseus ---------- -HCT.SV.TS LAS--SLS.L .D.PT----- ---------- ---------- ---------- ---------- ---------- --

Dipodomys_ordii ---------- --R..SV.TP LAG--G--.L S..PT----- ---------- ---------- ---------- ---------- ---------- --

Fukomys_damarensis ---------- --Q..CA.TP LAN--GL..L .K.PL----- ---------- ---------- ---------- ---------- ---------- --

Grammomys_surdaster ---------- --RE.SVGTS LAS--SL.RL .DIPTRIPTG AQPGWIQ--- ---------- --KGPHRTTQ NQMPGQG--- ---------- --

Heterocephalus_glaber ---------- --Q..SA.TP LAN--GL..L ...PL----- ---------- ---------- ---------- ---------- ---------- --

Ictidomys_tridecemlineatus ---------- --Q..SA.TS LAC--GL.EL ..RP.----- ---------- ---------- ---------- ---------- ---------- --

Jaculus_jaculus ---------- --HE.S.QTP LAS--GL.SL .--------- ---------- ---------- ---------- ---------- ---------- --

Marmota_flaviventris ---------- --Q..SA.TS LAC--GL.EL DG.P.----- ---------- ---------- ---------- ---------- ---------- --

Marmota_marmota ---------- --Q..SA.TS LAC--GL.EL ...P.----- ---------- ---------- ---------- ---------- ---------- --

Mastomys_coucha ---------- --CE.SV.TS LAS--SL.RL .DNPT----- ---------- ---------- ---------- ---------- ---------- --

Meriones_unguiculatus ---------- --RE.SVGTS LAS--SL..L .D.PT----- ---------- ---------- ---------- ---------- ---------- --

Mesocricetus_auratus ---------- -LCA.SM.TS LAC--QSLR. G--------- ---------- ---------- ---------- ---------- ---------- --

Microtus_ochrogaster ---------- ---K.SV.TS IAS--SLS.L TD.PT----- ---------- ---------- ---------- ---------- ---------- --

Mus_caroli ---------- --CE.SM.TS LAS--SL.RL .D.PT----- ---------- ---------- ---------- ---------- ---------- --

Mus_musculus ---------- --CV.SM.TS LAS--SL.RL .D.PT----- ---------- ---------- ---------- ---------- ---------- --

Mus_pahari ---------- --CE.SK.TS LAS--SL.RL .D.PT----- ---------- ---------- ---------- ---------- ---------- --

Mus_spicilegus ---------- --CV.SM.TS LAS--SL.RL .D.PT----- ---------- ---------- ---------- ---------- ---------- --

Nannospalax_galili ---------- --H..SA.TF LAS--SL.D- ---------- ---------- ---------- ---------- ---------- ---------- --

Octodon_degus ---------- --HE.SA.TP LVN--DL.-- ---------- ---------- ---------- ---------- ---------- ---------- --

Onychomys_torridus ---------- -HHEASV.IS FAS--SLS.L .D.HT----- ---------- ---------- ---------- ---------- ---------- --

Peromyscus_leucopus ---------- -HCE.SV.IS FVS--S.S.L .H.HI----- ---------- ---------- ---------- ---------- ---------- --

Peromyscus_maniculatus ---------- -HRE.SV.IS FVS--S.S.L .H.HI----- ---------- ---------- ---------- ---------- ---------- --

Rattus_norvegicus ---------- --CE.SAKTS LAS--SL.RL .D.PT----- ---------- ---------- ---------- ---------- ---------- --

Rattus_rattus ---------- --CE.SA.TS LAS--SL.RL .D.PT----- ---------- ---------- ---------- ---------- ---------- --

Urocitellus_parryii ---------- --Q..SA.TS LAC--GL.EL ..RP.----- ---------- ---------- ---------- ---------- ---------- --

**Supplementary Figure 6. Alignment of mammalian glucagon receptor (Gcgr) protein sequences**.

Predicted glucagon receptor (Gcgr) amino acid sequences from 124 mammals are based on the MAFFT [51] aligned coding sequences. Sequences are shown in single letter amino acid code, with identical residues indicated by a period (.) and gaps by dashes (-). The positions of the signal peptide and transmembrane domains (TM1 – TM7) in the human (*Homo sapiens*) sequence are indicated above the sequences, with <<< and >>> indications the extent of these domains. Amino acid sites involved in peptide ligand binding and G-protein binding are indicate by “P” and “G”, respectively, above the sequences and are from the GGPCRdb [63,64]. The anchor points for the Wootten numbering system [73] are indicated by $ with the corresponding numbers shown above.
