# Supplementary material for: Variation in the Evolution and Sequences of Proglucagon and the Receptors for Proglucagon-Derived Peptides in Mammals
Source: Front Endocrinol (Lausanne). 2021 Jul 12;12:700066. doi: 10.3389/fendo.2021.700066 (PMC8312260; doi:10.3389/fendo.2021.700066)
Supplement: Supplementary File 1 — Fasta formatted proglucagon (Gcg) coding sequences. [file DataSheet_1.zip › Supplement/Suplementary Figures/Fig S5 Gcg alignment.docx]

Signal Peptide << >> GRPP << >> Glucagon << IP-1 ## N

Ornithorhynchus_anatinus MKSFYFVAGLFVMLVQGSWQ RSLQDTEERARSLTAS-QMEPLDDVNQKTEE **KR** HSQGTFTSDYSKHLDAIRAKQFVDWLMNY **KR** GGGPIT **KR** HDEFA**R**

Monodelphis_domestica ...I.......I........ N.......KP.PY.D.-.T.....-.LMN.D .. ............Y..SR..QD..Q...ST .. N.NKVA .. ....E.

Notamacropus_eugenii ..NI..L....I....S... H.......KP.PY.D.-.T.....-.LMN.D .. ............Y..SR..QD..Q....T .. N.NKVA .. ....E.

Phascolarctos_cinereus ..NI..L....I....S... H.......KP.PY.D.-.T.....-.LMN.D .. ............Y..SR..QD..Q....T .. N.NKVA .. ....E.

Sarcophilus_harrisii ..NI..L....I....S.C. H.......KP.PY.D.-.T.....-.LMN.D .. ............Y..SR..QD..Q...ST .. N.NKVA .. ....E.

Trichosurus_vulpecula ..NI..L....I....S... HP......KP.PY.D.-.T.....-.LMN.D .. ............Y..SR..QD..Q....T .. N.NKVA .. ....E.

Vombatus_ursinus ..NI..L....I....N... H.......KP.PY.D.-.T.....-.LMN.D .. ............Y..SR..QD..Q....T .. N.NKVA .. ....E.

Choloepus_didactylus ...I................ ........KS..FPT.-.TD..S.PD.MM.D .. ............Y..SR..QD..Q...ST .. NRNN.A .. ....E.

Dasypus_novemcinctus ...I................ ...K....K...FP..-..D..S.PD.MV.D .. ............Y..SR..QD..Q....T .. NRNN.A .. ....E.

Chrysochloris_asiatica .R...V.............. H.......K...VS..-.TD..N.PD.MV.D .. ............Y..SR..QD..Q....T .. NKNN.A .. ..K.E.

Echinops_telfairi .RNI.IA....L..L.S... .A......KP..IS..-LSD..N.PD.M..D .. ............Y..SR..QD..Q...ST .. N.NN.A .. .N..E.

Elephantulus_edwardii ...I.V.....I........ YA......KS..VP..-PTDL.N.SDEMI.D .. ............Y..SR..LD..Q...DT .. NKNN.A .. ..K.E.

Loxodonta_africana .R.I.V.....I..I..... ........KP..FS..-.TD.PN.PD.MI.D .. ............Y..SR..QD..Q....T .. NRNN.A .. Q.K.E.

Orycteropus_afer ...I.I.............. ........KY..IP..-.TD..N.PD.M..D .. ............Y..SR..QD..Q....T .. NRNN.A .. N.K.E.

Trichechus_manatus .R.I.V........I..... H.......TSS.FS..-.TD..S.PD.MI.D .. ............Y..SR..QD..Q....T .. NRNN.A .. N.K.E.

Balaenoptera_acutorostrata ...I......L......... ........KS..FP.P-.TD..S.PD.MN.D .. ............Y..SR..QD..Q....T .. NKNN.A .. ....E.

Balaenoptera_musculus ...I......L......... ........KS..FP.P-.TD..N.PD.MN.D .. ............Y..SR..QD..Q....T .. NKNN.A .. ....E.

Delphinapterus_leucas ...I......L......... ........KS..FP.P-.TD..N.PD.MN.D .. ............Y..SR..QD..Q....T .. NKNN.A .. ....E.

Globicephala_melas ...I......L......... ........KS..FP.P-.TD..N.PD.MS.D .. ............Y..SR..QD..Q....T .. NKNN.A .. ....E.

Lagenorhynchus_obliquidens ...I......L......... ........KS..FP.P-.TD..N.PD.MS.D .. ............Y..SR..QD..Q....T .. NKNN.A .. ....E.

Lipotes_vexillifer ...I......L......... ........KS..FP.P-.TD..N.PD.MN.D .. ............Y..SR..QD..Q....T .. NKNN.A .. ....E.

Monodon_monoceros ...I......L......... ........KS..FP.P-.TD..N.PD.MN.D .. ............Y..SR..QD..Q....T .. NKNN.A .. ....E.

Neophocaena_asiaeorientalis ...I......L......... ........KS..FP.P-.TD..N.PD.MN.D .. ............Y..SR..QD..Q....T .. NKNN.A .. ....E.

Orcinus_orca ...I......L......... ........KS..FP.P-.TD..N.PD.MS.D .. ............Y..SR..QD..Q....T .. NKNN.A .. ....E.

Phocoena_sinus ...I......L......... ........KS..FP.P-.TD..N.PD.MN.D .. ............Y..SR..QD..Q....T .. NKNN.A .. ....E.

Physeter_catodon ..NI......L......... ........KS..FP.P-.TD..N.PD.MN.D .. ............Y..SR..QD..Q....T .. NKNN.A .. ....E.

Tursiops_truncatus ...I......L......... ........KS..FP.P-.TD..N.PD.MS.D .. ............Y..SR..QD..Q....T .. NKNN.A .. ....E.

Bison_bison ...L................ ....N...KSS.FP.P-.TD..G.PD.IN.D .. ............Y..SR..QD..Q....T .. NKNN.A .. ....E.

Bos_indicus ...L................ ....N...KSS.FP.P-.TD..G.PD.IN.D .. ............Y..SR..QD..Q....T .. NKNN.A .. ....E.

Bos_mutus ...L................ ....N...KSS.FP.P-.TN..G.PD.IN.D .. ............Y..SR..QD..Q....T .. NKNN.A .. ....E.

Bos_taurus ...L................ ....N...KSS.FP.P-.TD..G.PD.IN.D .. ............Y..SR..QD..Q....T .. NKNN.A .. ....E.

Bubalus_bubalis ...L................ .P..N...KSS.FP.P-.TD..G.PD.IN.D .. ............Y..SR..QD..Q....T .. NKNN.A .. ....E.

Camelus_bactrianus ...I................ ........KS..FP.P-.TDT.N.LDLMN.D .. ............Y..SR..QD..Q....T .. NKNN.A .. ....E.

Camelus_dromedarius ...I................ ........KS..FP.P-.TDT.N.LDLMN.D .. ............Y..SR..QD..Q....T .. NKNN.A .. ....E.

Camelus_ferus ...I................ ........KS..FP.P-.TDT.N.LDLMN.D .. ............Y..SR..QD..Q....T .. NKNN.A .. ....E.

Capra_hircus ...L......L...A..... ....N...KSS.FP.P-.TD..G.PD.MS.D .. ............Y..SR..QD..Q....T .. NKNN.A .. ....E.

Catagonus_wagneri ..TI................ ....N...KS..FP.P-.SD..G.PD.M..D .. ............Y..SR..QD..Q....T .. NKNN.A .. ....E.

Cervus_hanglu_yarkandensis ...L................ ........KSS.FP.P-.TDS.S.PD.IN.D .. ............Y..SR..QD..Q....T .. NKNN.A .. ....E.

Moschus_moschiferus ...L................ H...N...KSS.FP.P-.TD..S.PD.IN.D .. ............Y..SR..QD..H....T .. NKNN.A .. ....E.

Odocoileus_virginianus ...L................ ........KSS.FP.P-.TGS.S.PD.IN.D .. ............Y..SR..QD..Q....T .. NKNN.A .. ....E.

Ovis_aries ...L......L...A..... H...N...KSS.FP.P-.TD..G.PD.IS.D .. ............Y..SR..QD..Q....T .. NKNN.A .. ....E.

Sus_scrofa ..TI................ ....N...KS..FP.P-.TD....PD.M..D .. ............Y..SR..QD..Q....T .. NKNN.A .. ....E.

Vicugna_pacos ...I................ ........KS..FP.P-.TD..N.SDLMN.D .. ............Y..SR..QD..Q....T .. NKNN.A .. ....E.

Acinonyx_jubatus ...I................ ........KS..FP.P-.TD..N.PD.MI.D .. ............Y..SR..QD..Q....T .. NKNN.A .. ....E.

Ailuropoda_melanoleuca ...I................ ........KS...P.P-.TD..N.PD.MN.D .. ............Y..SR..QD..Q...ST .. NKNN.A .. ....E.

Callorhinus_ursinus ...I................ ........KS..FP.P-.TD..N.PD.MN.D .. ............Y..SR..QD..Q...ST .. NKNN.A .. ....E.

Canis_lupus ...I................ ........KS..FS.P-.T...N.LD.MN.D .. ............Y..SR..QD..Q....T .. NKNN.A .. ....E.

Enhydra_lutris ...I................ ........KS..FP.P-.TD..N.PD.MS.D .. ............Y..SR..QD..Q...ST .. NKNN.A .. ....E.

Eumetopias_jubatus ...I................ ........KS..FP.P-.TD..N.PD.MN.D .. ............Y..SR..QD..Q...ST .. NKNN.A .. ....E.

Felis_catus ...I................ ........KS..FP.P-.TD..N.PD.MI.D .. ............Y..SR..QD..Q....T .. NKNN.A .. ....E.

Halichoerus_grypus ...I................ ........KS..FP.P-.TD..N.PD.MN.D .. ............Y..SR..QD..Q...ST .. NKNN.A .. ....E.

Leptonychotes_weddellii ...I................ ........KS..FP.P-.TDL.N.PD.MN.D .. ............Y..SR..QD..Q...ST .. NKNN.A .. ....E.

Lontra_canadensis ...I............S... ........KS..FP.P-.TD..N.PD.MS.D .. ............Y..SR..QD..Q...ST .. NKNN.A .. ....E.

Lynx_canadensis ...I................ ........KS..FP.P-.TD..N.PD.MI.D .. ............Y..SR..QD..Q....T .. NKNN.A .. ....E.

Mirounga_leonina ...I................ ........KS..FP.P-.TD..N.PD.MN.D .. ............Y..SR..QD..Q...ST .. NKNN.A .. ....E.

Mustela_erminea ...I................ ...H....KS..FP.P-.TD..S.PD.MS.D .. ............Y..SR..QD..Q...ST .. NKNN.A .. ....E.

Mustela_putorius ...I................ L.......KS..FP.P-.TDL.S.PD.MS.D .. ............Y..SR..QD..Q...ST .. NKNN.A .. ....E.

Neomonachus_schauinslandi ...I................ ........KS..FP.P-.TD..N.PD.MN.D .. ............Y..SR..QD..Q...ST .. NKNN.A .. ....E.

Neovison_vison ...I................ ....E...KS..FP.P-.TD..S.PD.MS.D .. ............Y..SR..QD..Q...ST .. NKNN.A .. ....E.

Odobenus_rosmarus ...I................ ........KS..FP.P-.TD..N.PD.MN.D .. ............Y..SR..QD..Q...ST .. NKNN.A .. ....E.

Panthera_leo ...I................ ........KS..FP.P-.TD..N.PD.MI.D .. ............Y..SR..QD..Q....T .. NKNN.A .. ....E.

Panthera_pardus ...I................ ........KS..FP.P-.TD..N.PD.MI.D .. ............Y..SR..QD..Q....T .. NKNN.A .. ....E.

Panthera_tigris ...I................ ........KS..FP.P-.TD..N.PD.MI.D .. ............Y..SR..QD..Q....T .. NKNN.A .. ....E.

Phoca_vitulina ...I................ ........KS..FP.P-.TD..N.PD.MN.D .. ............Y..SR..QD..Q...ST .. NKNN.A .. ....E.

Puma_concolor ...I................ ........KS..FP.P-.TD....PD.MI.D .. ............Y..SR..QD..Q....T .. NKNN.A .. ....E.

Suricata_suricatta ...I................ ........KS..FP.P-.TD.PN.PD.MN.D .. ............Y..SR..QD..Q....T .. NKNN.A .. ....E.

Ursus_americanus ...I................ ........KS..FP.P-.TD..N.PD.MN.D .. ............Y..SR..QD..Q...ST .. NKNN.A .. ....E.

Ursus_arctos ...I................ ........KS..FP.P-.TD..N.PD.MN.D .. ............Y..SR..QD..Q...ST .. NKNN.A .. ....E.

Ursus_maritimus ...I................ ........KS..FP.P-.TD..N.PD.MN.D .. ............Y..SR..QD..Q...ST .. NKNN.A .. ....E.

Ursus_thibetanus ...I................ ........KS..FP.P-.TD..N.PD.MN.D .. ............Y..SR..QD..Q...ST .. NKNN.A .. ....E.

Vulpes_vulpes ...I................ ........KS..FS.P-.T...N.LD.MN.D .. ............Y..SR..QD..Q....T .. NKNN.A .. ....E.

Zalophus_californianus ...I................ ........KS..FP.P-.TD..N.PD.MN.D .. ............Y..SR..QD..Q...ST .. NKNN.A .. ....E.

Artibeus_jamaicensis ...I................ ........TSS.FP.P-.RD..N.P..MN.D .. ............Y..SR..QD..Q....T .. NKNN.A .. ....E.

Desmodus_rotundus ...I....A........... ........TSS.FP.P-.RD..N.P..MN.D .. ............Y..SR..QD..Q....T .. NKNN.A .. ....E.

Hipposideros_armiger ...I................ .....S..KSS.FP.P-.TD.FN.PG.MS.D .. ............Y..SR..QD..Q....T .. NKNN.A .. ....E.

Miniopterus_natalensis .R.I.L....L......... ........K.S.FP.P-.TD..NNPD.MN.D .. ............Y..SR..QD..Q....T .. NKNN.A .. Q.K.E.

Molossus_molossus ...I.......A........ ........K.S.FP.P-.TD..N.PD.MN.D .. ............Y..SR..QD..Q....T .. NKNN.A .. ....E.

Myotis_brandtii ...I................ ........KSS.FP.P-.ADS.N.PD.IN.. .. ............Y..TR..QD..E...TS .. NKNN.A .. ....E.

Myotis_lucifugus ...I................ ........KSS.FP.P-.ADS.N.PD.IN.D .. ............Y..TR..QD..E...TS .. NKNN.A .. ....E.

Phyllostomus_discolor ...I................ ........TSS.FP.P-.RD..N.P..MN.D .. ............Y..SR..QD..Q....T .. NKNN.A .. ....E.

Pipistrellus_kuhlii ...I................ ........K.S.FP..-.AD..N.PD.MN.D .. ............Y..SR..QD..E....T .. NKNN.A .. ....E.

Pteropus_alecto ...I..L............. .......MKSS.FP.P-.TD.FN.PD.MN.D .. ............Y..SR..QD..Q....T .. NKNN.A .. ....E.

Pteropus_vampyrus ...I..L............. .......MKSS.FP.P-.TD.FS.PD.MN.D .. ............Y..SR..QD..Q....T .. NKNN.A .. ....E.

Rhinolophus_ferrumequinum ...I............S... .....S..KSS.FP.P-.TD.FN.PE.MN.D .. ............Y..SR..QD..Q....T .. NKNN.A .. ....E.

Rousettus_aegyptiacus ...I..L............. .......MKSS.FP.P-.TD.FN.PD.MN.D .. ............Y..SR..QD..Q....T .. NKNN.A .. ....E.

Sturnira_hondurensis ...I............A... ........TSS.FP.P-.RD..N.P..MN.D .. ............Y..SR..QD..Q....T .. NKNN.A .. ....E.

Condylura_cristata ..TI.......-........ ....E...K...FP.P-.TD..S.AD.MN.D .. ............Y..SR..QD..Q....T .. NKNN.A .. ....E.

Erinaceus_europaeus ..TI................ .....V..KL.PFP.P-.TD..G.PDLMN.D .. ............Y..SR..QD..Q....T .. NKNN.A .. ....E.

Sorex_araneus ..TI................ H.......KSS.FP.P-.TD..NNPD.IN.D .. ............Y..SR..QD..R....T .. NKNN.A .. ....E.

Talpa_occidentalis ..II.......-........ ........K...FP.P-.TD..N.PD.MN.D .. ............Y..SR..QD..Q....T .. NKNN.A .. ....E.

Ceratotherium_simum ...I................ ........TS..FP.P-.TD..N.LD.MN.D .. ............Y..SR..QD..Q....T .. NKNN.A .. ....E.

Equus_asinus ...I............S... ........TS..FP.P-.TD..N.PD.MN.D .. ............Y..SR..QD..Q....T .. NKNN.A .. ....E.

Equus_caballus ...I............S... ........TS..FP.P-.TD..S.PD.MN.D .. ............Y..SR..QD..Q....T .. NKNN.A .. ....E.

Equus_przewalskii ...I............S... ........TS..FP.P-.TD..S.PD.MN.D .. ............Y..SR..QD..Q....T .. NKNN.A .. ....E.

Manis_javanica ...I...L..L......... .....R.DQ.G.FP.L-RSDQ.T.LQ.MDKD .. ..........T.Y...L..QE..Q....N .. NKSN.A .. ....E.

Manis_pentadactyla ...I..AV..L......... .....R.DK...FP.L-RSDQ.T.LE.MDKD .. ..........T.Y...L..QE..Q....N .. NRSN.A .. ....E.

Galeopterus_variegatus ...I................ ........KS.PFS..-.TD.F..PD.MN.G .. ............Y..SR..QD..Q....T .. NRNN.A .. ....E.

Prolemur_simus ...I.............T.. ....N...KS..FS..-.TD...EPDPMN.D .. ............Y..SR..QD..Q....T .. NRNN.A .. ....E.

Tupaia_chinensis ...I................ .....A..KS.AFS..-.TD..T.PD.M..D .. ............Y..SR..QD..Q....T .. NRNN.A .. ....E.

Aotus_nancymaae ...I................ ........KS..FS..-PID..S.PD.MN.D .. ............Y..SR..QD..Q....T .. NRNN.A .. ....E.

Callithrix_jacchus ...I................ ........KS..FS..-.TD..S.PD.MN.D .. ............Y..SR..QD..Q....T .. NRNN.A .. ....E.

Carlito_syrichta ...I................ ........QS..FS..-.TD...NPDHMN.D .. ............Y..SR..QD..Q....T .. NRNN.A .. ....E.

Cebus_capucinus ...I................ ........KS..FS..-.TD..S.PD.MN.D .. ............Y..SR..QD..Q....T .. NRNN.A .. ....E.

Cercocebus_atys ...I................ ........KS..VS..-.TD..G.PD.MN.D .. ............Y..SR..QD..Q....T .. NRNN.A .. ....E.

Chlorocebus_sabaeus ...I....V........... ........KP..VS..-.TD..G.PD.MN.D .. ............Y..SR..QD..Q....T .. NRNN.A .. ....E.

Colobus_angolensis ...I................ ........KS..FS..-.AD..G.PD.MN.D .. ............Y..TR..QD..Q....T .. NKNN.A .. ....E.

Gorilla_gorilla ...I................ ........KS..FS..-.AD..S.PD.MN.D .. ............Y..SR..QD..Q....N .. NRNN.A .. ....E.

Homo_sapiens ...I................ ........KS..FS..-.AD..S.PD.MN.D .. ............Y..SR..QD..Q....T .. NRNN.A .. ....E.

Hylobates_moloch ...I................ ........KS..FS..-.AD..S.PD.MN.D .. ............Y..SR..QD..Q....T .. NRNN.A .. ....E.

Macaca_fascicularis ...I................ ........KS..VS..-.TD..G.PD.MN.D .. ............Y..SR..QD..Q....T .. NRNN.A .. ....E.

Macaca_mulatta ...I................ ........KS..VS..-.TD..G.PD.MN.D .. ............Y..SR..QD..Q....T .. NRNN.A .. ....E.

Macaca_nemestrina ...I................ ........KS..VS..-.TD..G.PD.MN.D .. ............Y..SR..QD..Q....T .. NRNN.A .. ....E.

Mandrillus_leucophaeus ...I................ ........KS..VS..-.TD..G.PD.MN.D .. ............Y..SR..QD..Q....T .. NRNN.A .. ....E.

Microcebus_murinus ...M..........I..T.. ....N...KS..FS..-.TD...EPDPMN.D .. ............Y..SR..QD..Q....T .. NRNN.A .. ....E.

Nomascus_leucogenys ...I................ ........KS..FS..-.AD..S.PD.MN.D .. ............Y..SR..QD..Q....T .. NRNN.A .. ....E.

Otolemur_garnettii ...L.............T.. ....N...KS.LFS..-.TD..VEPG.MN.D .. ............Y..SR..QD..Q....T .. NRNN.A .. ....E.

Pan_paniscus ...I................ ........KS..FS..-.AD..S.PD.MN.D .. ............Y..SR..QD..Q....T .. NRNN.A .. ....E.

Pan_troglodytes ...I................ ........KS..FS..-.AD..S.PD.MN.D .. ............Y..SR..QD..Q....T .. NRNN.A .. ....E.

Papio_anubis ...I................ ........KS..VS..-.TD..G.PD.MN.D .. ............Y..SR..QD..Q....T .. NRNN.A .. ....E.

Piliocolobus_tephrosceles ...I................ ........KS..FS..-.AD..G.PD.MN.D .. ............Y..SR..QD..Q....T .. NRNN.A .. ....E.

Pongo_abelii ...I................ ........KS..FS..-.AD..S.PD.MN.D .. ............Y..SR..QD..Q....T .. NRNN.A .. ....E.

Propithecus_coquereli ...I.............T.. ....N...KS..FS..-.TD...EPDPMN.D .. ............Y..SR..QD..Q....T .. NRNN.A .. ....E.

Rhinopithecus_bieti ...I................ ........KS..FS..-.AD..G.PD.MN.D .. ............Y..SR..QD..Q....T .. NRNN.A .. ....E.

Rhinopithecus_roxellana ...I................ ........KS..FS..-.AD..G.PD.MN.D .. ............Y..SR..QD..Q....T .. NRNN.A .. ....E.

Saimiri_boliviensis ...I................ ........KS..FS..-.TD..S.PD.MN.D .. ............Y..SR..QD..Q....T .. NRNN.A .. ....E.

Sapajus_apella ...I................ ........KS..FS..-.TG..S.PD.MN.D .. ............Y..SR..QD..Q....T .. NRNN.A .. ....E.

Theropithecus_gelada ...I................ ........KS..VS..-.TD..G.PD.MN.D .. ............Y..SR..QD..Q....T .. NRNN.A .. ....E.

Trachypithecus_francoisi ...I................ ........KS..FS..-.AD..G.PD.MN.D .. ............Y..SR..QD..Q....T .. NRNN.A .. ....E.

Ochotona_princeps ...I......LI...H.... ....E...KI..FS.P-.TNLP..TDLM..D .. ............Y..SR..QD..Q....T .. NRNN.A .. ....E.

Oryctolagus_cuniculus ...I................ ........KS..FP..-.TDL.G.AD.M..D .. ............Y..SR..QD..Q....T .. NRNN.A .. ....E.

Arvicanthis_niloticus ..TI.I....LI..I..... H.......NP..FP.P-.T...E.PD.MN.D .. ............Y..SR..QD..Q....T .. NRNN.A .. ....E.

Arvicola_amphibius ..NI.I...F.I........ H..R....KP..FP..-.TD..Q.PD.IN.D .. ............Y..SR..QD..Q....T .. NRNN.A .. ....E.

Castor_canadensis ..NI.Y.....I........ H.......KP..FS..-.TD..S.PD.M..D .. ............Y..SR..QD..Q....T .. NRNN.A .. ....E.

Cavia_porcellus ...V.......I..A..... ........KP..VS..-.TDM...PD.MN.D .. ............Y..SR..Q..LK..L.V .. NRNN.A .. ....E.

Chinchilla_lanigera ...I.......I........ ........NP..FS..-RADV...PD.MN.D .. ...............SRY.QE..Q....T .. NRNN.A .. ....E.

Cricetulus_griseus ..NI.I.............. H...N...KP..FPV.-.TD..E.SD.IN.D .. ............Y..SR..QD..Q....T .. NRNN.A .. ...YE.

Dipodomys_ordii ...I.......I........ ............FP..-.TS..E.P.EMN.D .. ............Y..SR..QD..Q....T .. NRNN.A .. ....E.

Fukomys_damarensis ...I................ H.......KP..FS..-.T.LP..PD.MN.D .. ............Y..SR..QD..Q....T .. NRNN.A .. ....E.

Grammomys_surdaster ..TI.I....LI........ H.......NP..FP..-.T.L.E.PD.MN.D .. ............Y..SR..QD..Q....T .. NRNN.A .. ....E.

Heterocephalus_glaber ...I...V...I........ ........KH..FS..-.TDL...PD.MN.D .. ............Y..SR..QD..Q....T .. NRNN.A .. ....E.

Ictidomys_tridecemlineatus ..NI.......I........ ........KT..FP..-.TD..N.PD.MN.D .. ............Y..SR..QD..Q....T .. NRNN.A .. ....E.

Jaculus_jaculus ..NI.......I........ ........KP.Y.LLKF.TT....PD.M..D .. ............Y..SR..QD..Q....A .. .RNN.A .. ....E.

Marmota_flaviventris ..NI.......I........ ........KT..FP..-.TD..N.PD.MN.D .. ............Y..SR..QD..Q....T .. NRNN.A .. ....E.

Marmota_marmota ..NI.......I........ ........KT..FP..-.TD..N.PD.MN.D .. ............Y..SR..QD..Q....T .. NRNN.A .. ....E.

Mastomys_coucha ..TV.L....LL........ HT......NP..FP..-.T..R.NAD.MN.D .. ............Y..SR..QD..Q....T .. NWNN.A .. ....E.

Meriones_unguiculatus ...V.V....LI........ HPI.....KP..IP..-.T...E.PD.MN.D .. ............Y..SR..QD..Q....T .. NRNN.A .. ....E.

Mesocricetus_auratus ..NI.I...F.......... H.......KS..FP..-.TD..E.PD.IN.D .. ............Y..SR..QD..Q....T .. NRNN.A .. ....E.

Microtus_ochrogaster ..NI.I...F.I........ H.......KP..FP..-.T...Q.PD.IN.D .. ............Y..SR..QD..Q....T .. NRNN.A .. ....E.

Mus_caroli ..TV.I....LI........ HA......NP..FP..-.I.AHE.PD.MN.D .. ............Y..SR..QD..Q....T .. NRNN.A .. ....E.

Mus_musculus ..TI......LI........ HA......NP..FP..-.T.AHE.PDEMN.D .. ............Y..SR..QD..Q....T .. NRNN.A .. ....E.

Mus_pahari ..TI.I....LI....S... HA...P..NP..FP..-.I..HE.PD.MN.D .. ............Y..SR..QD..Q....T .. NRNNVA .. ....E.

Mus_spicilegus ..TI......LI........ HA......NP..FP..-.I.AHE.PDEMN.D .. ............Y..SR..QD..Q....T .. NRNN.A .. ....E.

Nannospalax_galili ...V.......I........ ........KP..FS..-.TD....PD.MN.D .. ............Y..SR..QD..Q....T .. NRNN.A .. ....E.

Octodon_degus ...I................ HP......KP..FST.-.TDL...PD.MN.D .. ............F..TR..QD.L...K.T .. NRNE.A .. ....E.

Onychomys_torridus ..TI.I...I.I........ H.......KP..FS.T-.TD..E.PD.IS.D .. ............Y..SR..QD..Q....T .. NRNN.A .. ....E.

Peromyscus_leucopus ..NI.I...F.I........ H.......KP..FS..-.TDL.E.PD.IS.D .. ............Y..SR..QD..Q....T .. NRNN.A .. ....E.

Peromyscus_maniculatus ..NI.I...F.I........ H.......KP..FS..-.TDL.E.PD.IS.D .. ............Y..SR..QD..Q....T .. NRNN.A .. ....E.

Rattus_norvegicus ..TV.I.............. HAP.....N...FP..-.T...E.P..IN.D .. ............Y..SR..QD..Q....T .. NRNN.A .. ....E.

Rattus_rattus ..TV.I.............. HAP.....N...FP..-.T...E.P..IN.D .. ............Y..SR..QD..Q....T .. NRNN.A .. ....E.

Sciurus_vulgaris ..NI.......I........ ........KT.FFPT.-.TD..N.PD.MI.D .. ............Y..SR..QD..Q....T .. NRNN.A .. ....E.

Spermophilus_dauricus ..NI.......I........ L.......KT..FP..-.TD..N.PD.MN.D .. ............Y..SR..QD..Q....T .. NRNN.A .. ....E.

Urocitellus_parryii ..NI.......I........ ........KT..FP..-.TD..N.PD.MN.D .. ............Y..SR..QD..Q....T .. NRNN.A .. ....E.

^^ GLP-1 << IP-2 >> GLP-2 2 <<

Ornithorhynchus_anatinus HSEGTFTNDVTRLLEEKATSEFIAWLLKGLE **KR** T-SREIAESEELW **RR** HADGTFSHELSGVLDQLATKDFLNWLLLPEVEE **RK**

Monodelphis_domestica .A.....S..SSY..GQ.AK......V..RG R. AFPE.VTIV...G .. ....S..D.MNT...T...R..I....QNK.TD ..

Notamacropus_eugenii .A.....S..SSY..GQ.AK......V..RG R. AFPE.VTIV...G .. ....S..D.MNT...N...R..I....QTK.TD ..

Phascolarctos_cinereus .A.....S..SSY..GQ.AK......V..RG R. AFPE.VTIV...G .. ....S..D.MNT...N...R..I....QTK.TD ..

Sarcophilus_harrisii .A.....S..SSY..GQ.AK......V..RG R. AFPE.VTIV...G .. ....S..D.MNT...NI..R..I....QTKIID .N

Trichosurus_vulpecula .A.....S..SSY..GQ.AK......V..RG R. AFPE.V.IV...R .. ....S..D.MNT...N...R..I....QTK.TD ..

Vombatus_ursinus .A.....S..SSY..GQ.AK......V..RG R. AFPE.VTIV...G .. ....S..D.MNT...N...R..I....QTK.TD ..

Choloepus_didactylus .A.....S..SSY..GQ.AK......V..RG R. DFPE.VTIV...R .. ....S..D.MNT...N...R..I....QTKITD ??

Dasypus_novemcinctus .A.....S..SSY..GQ.AK......V..RG R. DFPE.VTIV...R .. ....S..D.MNT...N...R..I....QTKITD ..

Chrysochloris_asiatica .A.....S..SSY..GQ.AK......V..RG R. DFPE.V.IV...R .. ....S..D.MNT...N...R..I....QTKITD ..

Echinops_telfairi .A.....S..SSY..GQ.AK......V..RG R. DFPK.DSFA..-R .. ....S..D.MNTI..N..AR..I....QTKITD ..

Elephantulus_edwardii .A.....S..SSY..GQ.AK......V..RG R. DLPD.V.IV...S .. ....S..D.MNT...N...R..I....QTKITD ..

Loxodonta_africana .A.....S..SSY..GQ.AK......V..RG R. DFPE.V-IV...R .. ....S..D.MNT...N...R..I....QTKITD ..

Orycteropus_afer .A.....S..SSY..GQ.AK......V..RG R. DFPE.VTIV...R .. ....S..D.MNT...N...RE.I....QTKITD ..

Trichechus_manatus .A.....S..SSY..GQ.AK......V..RG R. DFPE.VTIV...R .. ....S..D.MNT...N...R..I....QTKITD ..

Balaenoptera_acutorostrata .A.....S..SSY..GQ.AK......V..RG R. DFPE.VTIV...R .. ....S..D.MNT...N...R..I....QTKITD ..

Balaenoptera_musculus .A.....S..SSY..GQ.AK......V..RG R. DFPE.VTIV...R .. ....S..D.MNT...N...R..I....QTKITD ..

Delphinapterus_leucas .A.....S..SSY..GQ.AK......V..RG R. DFPE.VTIV...R .. ....S..D.MNT...N...R..I....QTKITD ..

Globicephala_melas .A.....S..SSY..GQ.AK......V..RG R. DFPE.VTIV...R .. ....S..D.MNT...N...R..I....QTKITD ..

Lagenorhynchus_obliquidens .A.....S..SSY..GQ.AK......V..RG R. DFPE.VTIV...H .. ....S..D.MNT...N...R..I....QTKITD ..

Lipotes_vexillifer .A.....S..SSY..GQ.AK......V..RG R. DFPE.VTIV...R .. ....S..D.MNT...N...R..I....QTKITD ..

Monodon_monoceros .A.....S..SSY..GQ.AK......V..RG R. DFPE.VTIV...R .. ....S..D.MNT...N...R..I....QTKITD ..

Neophocaena_asiaeorientalis .A.....S..SSY..GQ.AK......V..RG R. DFPE.VTIV...R .. ....S..D.MNT...S...R..I....QTK.TD ..

Orcinus_orca .A.....S..SSY..GQ.AK......V..RG R. DFPE.VTIV...R .. ....S..D.MNT...N...R..I....QTKITD ..

Phocoena_sinus .A.....S..SSY..GQ.AK......V..RG R. DFPE.VTIV...R .. ....S..D.MNT...N...R..I....QTK.TD ..

Physeter_catodon .A.....S..SSY..GQ.AK......V..RG R. DFP-.VTIV...R .. ....S..D.MNT...S...R..I....QTKITD ..

Tursiops_truncatus .A.....S..SSY..GQ.AK......V..RG R. DFPE.VTIV...R .. ....S..D.MNT...N...R..I....QTKITD ..

Bison_bison .A.....S..SSY..GQ.AK......V..RG R. DFPE.VNIV...R .. ....S..D.MNT...S...R..I....QTKITD ..

Bos_indicus .A.....S..SSY..GQ.AK......V..RG R. DFPE.VNIV...R .. ....S..D.MNT...S...R..I....QTKITD ..

Bos_mutus .A.....S..SSY..GQ.AK......V..RG R. DFPE.VNIV...R .. ....S..D.MNT...S...R..I....QTKITD ..

Bos_taurus .A.....S..SSY..GQ.AK......V..RG R. DFPE.VNIV...R .. ....S..D.MNT...S...R..I....QTKITD ..

Bubalus_bubalis .A.....S..SSY..GQ.AK......V..RG R. DFPE.VNIV...R .. ....S..D.MNTI..S...R..I....QTKITD ..

Camelus_bactrianus .A.....S..SSY..GQ.AK......V..RG R. DFPE.VTIV...R .. ....S..D.MNTI..N...Q..I....QTKITD ..

Camelus_dromedarius .A.....S..SSY..GQ.AK......V..RG R. DFPE.VTIV...R .. ....S..D.MNTI..N...Q..I....QTKITD ..

Camelus_ferus .A.....S..SSY..GQ.AK......V..RG R. DFPE.VTIV...R .. ....S..D.MNTI..N...Q..I....QTKITD ..

Capra_hircus .A.....S..SSY..GQ.AK......V..RG R. DFPE.VNIV...R .. ....S..D.MNT...S...R..I....QTKITD ..

Catagonus_wagneri .A.....S..SSY..GQ.AK......V..RG R. EFPE.VTFV...R .. ....S..D.MNT...N...R..I....HTKITD ..

Cervus_hanglu_yarkandensis .A.....S..SSY..GQ.AK......V..RG R. DFPE.VSLV...R .. ....S..D.M.T...S...R..I....QTKITD ..

Moschus_moschiferus .A.....S..SSY..GQ.AK......V..RG R. DFPE.VSLV...R .. ....S..E.MNT...S...R..I....QTKITD ..

Odocoileus_virginianus .A.....S..SSY..GQ.AK......V..RG R. DFPE.VNIV...R .. ....S..D.MNT...S...R..I....QTKITD ..

Ovis_aries .A.....S..SSY..GQ.AK......V..RG R. DFPE.VNIV...R .. ....S..D.MNT...S...R..I....QTKITD ..

Sus_scrofa .A.....S..SSY..GQ.AK......V..RG R. DFPE.VTIV...R .. ....S..D.MNT...N...R..I....HTKITD SL

Vicugna_pacos .A.....S..SSY..GQ.AK......V..RG R. DFPE.VTIV...R .. ....S..D.MNTL..N...Q..I....QTKITD ..

Acinonyx_jubatus .A.....S..SSY..GQ.AK......V..RG R. DFPE.VTIV...R .. ....S..D.MNT...N...R..I....QTKIT. ..

Ailuropoda_melanoleuca .A.....S..SSY..GQ.AK......V..RG R. DFPE.V.IV...R .. ....S..D.MNT...D...R..I....QTKIT. ..

Callorhinus_ursinus .A.....S..SSY..GQ.AK......V..RG R. EFPE.V.IV...R .. ....S..D.MNT...N...R..I....QTKIT. ..

Canis_lupus .A.....S..SSY..GQ.AK......V..RG R. DFPE.V.IV..FR .. ....S..D.MNT...T...R..I....QTKITD ..

Enhydra_lutris .A.....S..SSY..GQ.AK......V..RG R. DFPE.V.IV...R .. ....S..D.MNT...D...R..I....QTKIT. ..

Eumetopias_jubatus .A.....S..SSY..GQ.AK......V..RG R. EFPE.V.IV...R .. ....S..D.MNT...N...R..I....QTKIT. ..

Felis_catus .A.....S..SSY..GQ.AK......V..RG R. DFPE.VTIV...R .. ....S..D.MNT...N...R..I....QTKIT. ..

Halichoerus_grypus .A.....S..SSY..GQ.AK......V..RG R. DFPE.V.IV...R .. ....S..D.MNT...D...R..I....QTKIT. ..

Leptonychotes_weddellii .A.....S..SSY..GQ.AK......V..RG R. DFPE.V.IV...R .. ....S..D.MNT...D...R..I....QTKIT. ..

Lontra_canadensis .A.....S..SSY..GQ.AK......V..RG R. DFPE.V.IV...R .. ....S..D.MNT...D...R..I....QTKIT. ..

Lynx_canadensis .A.....S..SSY..GQ.AK......V..RG R. DFPE.VTIV...R .. ....S..D.MNT...N...R..I....QTKIT. ..

Mirounga_leonina .A.....S..SSY..GQ.AK......V..RG R. DFPE.V.VV...R .. ....S..D.MNT...D...R..I....QTKIT. ..

Mustela_erminea .A.....S..SSY..GQ.AK......V..RG R. DFPE.V.IV...R .. ....S..D.MNT...D...R..I....QTKIT. ..

Mustela_putorius .A.....S..SSY..GQ.AK......V..RG R. DFPE.V.IV...R .. ....S..D.MNT...D...R..I....QTKIT. ..

Neomonachus_schauinslandi .A.....S..SSY..GQ.AK......V..RG R. DFPE.V.IV...R .. ....S..D.MNT...D...R..I....QTKIT. ..

Neovison_vison .A.....S..SSY..GQ.AK......V..RG R. DFPE.V.IV...R .. ....S..D.MNTI..D...R..I....QTKIT. ..

Odobenus_rosmarus .A.....S..SSY..GQ.AK......V..RG R. DFPE.V.IV...R .. ....S..D.MNT...N...R..I....QTKIT. ..

Panthera_leo .A.....S..SSY..GQ.AK......V..RG R. DFPE.VTIV...R .. ....S..D.MNT...N...R..I....QTKIT. ..

Panthera_pardus .A.....S..SSY..GQ.AK......V..RG R. DFPE.VTIV...R .. ....S..D.MNT...N...R..I....QTKIT. ..

Panthera_tigris .A.....S..SSY..GQ.AK......V..RG R. DFPE.VTIV...R .. ....S..D.MNT...N...R..I....QTKIT. ..

Phoca_vitulina .A.....S..SSY..GQ.AK......V..RG R. DFPE.V.IV...R .. ....S..D.MNT...D...R..I....QTKIT. ..

Puma_concolor .A.....S..SSY..GQ.AK......V..RG R. DFPE.VTIV...R .. ....S..D.MNT...N...R..I....QTKIT. ..

Suricata_suricatta .A.....S..SSY..GQ.AK......V..RG R. DFPE.VTIV...R .. ....S..D.MNT...S...R..I....QTKIT. ..

Ursus_americanus .A.....S..SSY..GQ.AK......V..RG R. DFPE.V.IV...R .. ....S..D.MNT...D...R..I....QTKIT. ..

Ursus_arctos .A.....S..SSY..GQ.AK......V..RG R. DFPE.V.IV...R .. ....S..D.MNT...D...R..I....QTKIT. ..

Ursus_maritimus .A.....S..SSY..GQ.AK......V..RG R. DFPE.V.IV...R .. ....S..D.MNT...D...R..I....QTKIT. ..

Ursus_thibetanus .A.....S..SSY..GQ.AK......V..RG R. DFPE.V.IV...R .. ....S..D.MNT...D...R..I....QTKIT. ..

Vulpes_vulpes .A.....S..SSY..GQ.AK......V..RG R. DFPE.V.IV...R .. ....S..D.MNT...T...R..I....QTKITD ..

Zalophus_californianus .A.....S..SSY..GQ.AK......V..RG R. EFPE.V.IV...R .. ....S..D.MNT...N...R..I....QTKIT. ..

Artibeus_jamaicensis .A.....S..SSY..GQ.AK......V..RG R. DFPE.VTIV...R .. ....S..D.MNT...N...R..I...IQTKITD ..

Desmodus_rotundus .A.....S..SSY..GQ.AK......V..RG R. DFPE.VTIV...R .. ....S..D.MNT...N...R..I....QTKITD ..

Hipposideros_armiger .A.....S..SSY..GQ.AK......V..RG R. DFPE.VTII...R .. ....S..D.MNT...N...R..I....QTKITD ..

Miniopterus_natalensis .A.....S..SSY..GQ.AK......V..RG R. DFPD.VTIV...R .. ....S..D.MNT...N...R..I...IQTKITD ..

Molossus_molossus .A.....S..SSY..GQ.AK......V..RG R. DFPE.VTIV...R .. ....S..E..NT...N...R..I....QTKITD ..

Myotis_brandtii .......S.LSSY..G...K......V.... R**Q** DLPEA--IP.A.R .. ..E.S..D.MNT...N...Q..I....QNKITY ..

Myotis_lucifugus .......S.LSSY..G...K......V.... R**Q** DLPEA--IP.A.R .. ..E.S..D.MNT...N...Q..I....QNKITD ..

Phyllostomus_discolor .A.....S..SSY..GQ.AK......V..RG R. DFPE.VTIV...R .. ....S..D.MNT...N...R..I....QTKITD ..

Pipistrellus_kuhlii .A.....S.LSTY..DQ.VR......V..RD RK DFPEG-TIA...R .. ..E.S..D.MNT...N...R..I....QNKITD ..

Pteropus_alecto .A.....S..SSY..GQ.AK......V..RG R. DFPE.VTII...R .. ....S..D.MNT...N...R..I....QTKITD ..

Pteropus_vampyrus .A.....S..SSY..GQ.AK......V..RG R. DFPE.VTII...R .. ....S..D.MNT...N...R..I....QTKITD ..

Rhinolophus_ferrumequinum .A.....S..SSY..GQ.AK......V..RG R. DFPE.VTII...G .. ....S..D.MNT...N...R..I....QTKITD ..

Rousettus_aegyptiacus .A.....S..SSY..GQ.AK......V..RG R. DFPE.VTII...R .. ....S..D.MNT...N...R..I....QTKITD ..

Sturnira_hondurensis .A.....S..SSY..GQ.AK......V..RG R. DFPE.VTIV...R .. ....S..D.MNT...N...R..I...IQTKITD ??

Condylura_cristata .A.....S..SSY..GQ.AK......V..RG R. DFPE.V.IV...N .. ....S..D.MNT...N...R..I....QTKITD ..

Erinaceus_europaeus .A.....S..SSY..GQ.AK......V..RG R. DFPE.VTIV...R .. ....S..D.MNT...N...R..IK...QNKIND ..

Sorex_araneus .A.....S..SSY..GQ.AK......V..RG R. DFPE.V.IV...G .. ....S..D.MNT...N...R..I....QTKITD ..

Talpa_occidentalis .A.....S..SSY..GQ.AK......V..RG R. DFPE.V.MV...N .. ....S..D.MNT...N...R..I....QTKITD ..

Ceratotherium_simum .A.....S..SSY..GQ.AK......V..RG R. DFPE.VTIV...R .. ....S..D.MNT...N...R..I....QTKITD .M

Equus_asinus .A.....S..SSY..GQ.AK......V..RG R. DFPE.VTIV...R .. ....S..D.MNT...N...R..I....QTKITD ..

Equus_caballus .A.....S..SSY..GQ.AK......V..RG R. DFPE.VTIV...R .. ....S..D.MNT...N...R..I....QTKITD ..

Equus_przewalskii .A.....S..SSY..GQ.AK......V..RG R. DFPE.VTIV...R .. ....S..D.MNT...N...R..I....QTKITD ..

Manis_javanica .A.....S..SSY..GQ.AK......V..RG R. DFPE.V.IV...R .. ....S..D.MNT...N...R..I....QTKIT. ..

Manis_pentadactyla .A.....S..SSY..GQ.AK......V..RG R. DFPE.V.IV...R .. ....S..D.MNT...N...R..I....QTKITD ..

Galeopterus_variegatus .A.....S..SSY..GQ.AK......V..RG R. DFPE.V.FV...R .. ....S..D.MNTI..N...R..I...IQTKITD ..

Prolemur_simus .A.....S..SSY..GQ.AK......V..RG R. DFPE.VTIV...G .. ....S..D.MNT...N...R..I...IKNKITD ..

Tupaia_chinensis .A.....S..SSY..GQ.AK......V..RG R. DFPD.V.IV...R .. ....S..D.MNT...N...R..I...IQTKITD ..

Aotus_nancymaae .A.....S..SSY..GQ.AK......V..RG R. DFPE.V.IV...G .. ....S..D.MNTI..N..AR..I...IQTKITD ..

Callithrix_jacchus .A.....S..SSY..GQ.AK......V..RG R. DFPE.V.IV...G .. ....S..D.MNTI..N..AR..I...IQTKITD ..

Carlito_syrichta .A.....S..SSY..GQ.AK......V..RG R. DFPE.VTIV...G .. ....S..D.MNT...H...R..I....QTKITD ..

Cebus_capucinus .A.....S..SSY..GQ.AK......V..RG R. DFPE.V.IV...G .. ....S..D.MNTI..N..AR..I...IQTKITD ..

Cercocebus_atys .A.....S..SSY..GQ.AK......V..RG R. DFPE.V.IV...G .. ....S..D.MNT...N...R..I...IQTKITD ..

Chlorocebus_sabaeus .A.....S..SSY..GQ.AK......V..RG R. DFPE.V.IV...G .. ....S..D.MNT...N...R..I...IQTKITD ..

Colobus_angolensis .A.....S..SSY..GQ.AK......V..RG R. DFPEDV.IV...G .. ....S..D.MNT...N...R..I...IQTKITD ..

Gorilla_gorilla .A.....S..SSY..GQ.AK......V..RG R. DFPE.V.IV...G .. ....S..D.MNTI..N..AR..I...IQTKITD ..

Homo_sapiens .A.....S..SSY..GQ.AK......V..RG R. DFPE.V.IV...G .. ....S..D.MNTI..N..AR..I...IQTKITD ..

Hylobates_moloch .A.....S..SSY..GQ.AK......V..RG R. DFPE.V.IV...G .. ....S..D.MNTI..N..AR..I...IQTKITD ..

Macaca_fascicularis .A.....S..SSY..GQ.AK......V..RG R. DFPE.V.IV...G .. ....S..D.MNT...N...R..I...IQTKITD ..

Macaca_mulatta .A.....S..SSY..GQ.AK......V..RG R. DFPE.V.IV...G .. ....S..D.MNT...N...R..I...IQTKITD ..

Macaca_nemestrina .A.....S..SSY..GQ.AK......V..RG R. DFPE.V.IV...G .. ....S..D.MNT...N...R..I...IQTKITD ..

Mandrillus_leucophaeus .A.....S..SSY..GQ.AK......V..RG R. DFPE.V.IV...G .. ....S..D.MNT...N...R..I...IQTKITD ..

Microcebus_murinus .A.....S..SSY..GQ.AK......V..RG R. DFPE.VTIV...G .. ....S..D.MNT...N...RE.I...IQNKITD ..

Nomascus_leucogenys .A.....S..SSY..GQ.AK......V..RG R. DFPE.V.IV...G .. ....S..D.MNTI..N...R..I...IQTKITD ..

Otolemur_garnettii .A.....S..SSY..GQ.AK......V..RG R. EFPE.E.IV...G .. ....S..D.MNT...N...RE.I...RQNKITD ..

Pan_paniscus .A.....S..SSY..GQ.AK......V..RG R. DFPE.V.IV...G .. ....S..D.MNTI..N..AR..I...IQTKITD ..

Pan_troglodytes .A.....S..SSY..GQ.AK......V..RG R. DFPE.V.IV...G .. ....S..D.MNTI..N..AR..I...IQTKITD ..

Papio_anubis .A.....S..SSY..GQ.AK......V..RG R. DFPE.V.IV...G .. ....S..D.MNT...N...R..I...IQTKITD ..

Piliocolobus_tephrosceles .A.....S..SSY..GQ.AK......V..RG R. DFPEDV.IV...G .. ....S..D.MNT...N...R..I...IQTKITD ..

Pongo_abelii .A.....S..SSY..GQ.AK......V..RG R. DFPE.V.IV...G .. ....S..D.MNTI..N..AR..I...IQTKITD ..

Propithecus_coquereli .A.....S..SSY..GQ.AK......V..RG R. DFPE.VTIV...G .. ....S..D.MNT...N...R..I...IQNKITD ..

Rhinopithecus_bieti .A.....S..SSY..GQ.AK......V..RG R. DFPEDV.IV...G .. ....S..D.MNT...N...R..I...IQTKITD ..

Rhinopithecus_roxellana .A.....S..SSY..GQ.AK......V..RG R. DFPEDV.IV...G .. ....S..D.MNT...N...R..I...IQTKITD ..

Saimiri_boliviensis .A.....S..SSY..GQ.AK......V..RG R. DFPE.V.IV...G .. ....S..D.MNTI..N..AR..I...IQTKITD ..

Sapajus_apella .A.....S..SSY..GQ.AK......V..RG R. DFPE.V.IV...G .. ....S..D.MNTI..N...R..I...IQTKITD ..

Theropithecus_gelada .A.....S..SSY..GQ.AK......V..RG R. DFPE.V.IV...G .. ....S..D.MNT...N...R..I...IQTKITD ..

Trachypithecus_francoisi .A.....S..SSY..GQ.AK......V..RG R. DFPEDV.IV...G .. ....S..D.MNT...N...R..I...IQTKITD ..

Ochotona_princeps .A.....S..SSY..GQ.AK......V..RG R. DFPE.VTIV...R .. ....S..D.MNAI..S...R..I....QTKITD ..

Oryctolagus_cuniculus .A.....S..SSY..GQ.AK......V..RG R. DFPE.VTIV...R .. ....S..D.MNT...S...R..I....QTKISD ..

Arvicanthis_niloticus .A.....S..SSY..GQ.AK......V..RG R. DFPE.VVLA...G .. ....S..D.MNTI..N...R..I...IQTKITD K.

Arvicola_amphibius .A.....S..SSY..GQ.AK......V..RG R. DFPE.VTIV...G .. ....S..D.MNTI..N...R..I...IQTKITD ??

Castor_canadensis .A.....S..SSY..GQ.AK......V..RG R. DFPE.VTIV...R .. ....S..D.MNT...N...R..I...IQTKITD ..

Cavia_porcellus .A.....S..SSY..GQ.AK......V..RG R. DFPE.V.IV...G .. ....S..D.MNTI..N...R..I...IQTKITD ..

Chinchilla_lanigera .A.....S..SSY..GQ.AK......V..RG R. DFPE.VTIV...R .. ....S..D.MNT...N...R..I...IQTKITD ..

Cricetulus_griseus .A.....S..SSY..GQ.AK......V..RG R. DFPE.VTIV...G .. ....S..D.MNTI..S...R..I...IQTKITD K.

Dipodomys_ordii .A.....S..SSY..GQ.AK......V..RG R. DFPE.VTIV...R .. ....S..D.M.T...S...R..I...IQTKIT. ..

Fukomys_damarensis .A.....S..SSY..GQ.AK......V..RG R. DFPE.VTIV...R .. ....S..D.MNT...T...R..I...IQTKIT. ..

Grammomys_surdaster .A.....S..SSY..GQ.AK......V..RG R. DFPE.VVIA...G .. ....S..D.MNTI..N...R..I...IQTKITD K.

Heterocephalus_glaber .A.....S..SSY..GQ.AK......V..RG R. DFPE.VTIV...R .. ....S..D.MNT...T...R..I...IQTKITD ..

Ictidomys_tridecemlineatus .A.....S..SSY..GQ.AK......V..R. R. DFPE.VTIV...R .. ....S..D.MNT...N...R..I...IQTKITD ..

Jaculus_jaculus .A.....S..SSY..GQ.AK......V..RG R. EFPE.V.IV...R .. ....S..D.MNT...N...R..I...IQTKITD ..

Marmota_flaviventris .A.....S..SSY..GQ.AK......V..R. R. DFPE.VTIV...R .. ....S..D.MNT...N...R..I...IQTKITD ..

Marmota_marmota .A.....S..SSY..GQ.AK......V..R. R. DFPE.VTIV...R .. ....S..D.MNT...N...R..I...IQTKITD ..

Mastomys_coucha .A.....S..SSY..GQ.AK......V..RG R. DFPE.V.IA...G .. ....S..D.MNTI..N...R..I...IQTKITD K.

Meriones_unguiculatus .A.....S..SSY..GQ.AK......V..RG R. DFPE--VIA...G .. ....S..D.MNTI..S...R..I...IQTKITD K.

Mesocricetus_auratus .A.....S..SSY..GQ.AK......V..RG R. DFPE.VTIV...G .. ....S..D.MNTI..S...R..I...IQTKITD K.

Microtus_ochrogaster .A.....S..SSY..GQ.AK......V..RG R. DFPE.VTIV...G .. ....S..D.MNTI..N...R..I...IQTKITD K.

Mus_caroli .A.....S..SSY..GQ.AK......V..RG R. DFPE.V.IA...G .. ....S..D.MNTI..N...R..I...IQTKITD K.

Mus_musculus .A.....S..SSY..GQ.AK......V..RG R. DFPE.V.IA...G .. ....S..D.M.TI..N...R..I...IQTKITD K.

Mus_pahari .A.....S..SSY..GQ.AK......V..RG R. DFPE.V.IA...G .. ....S..D.MNTM..N...R..I...IQTKITD K.

Mus_spicilegus .A.....S..SSY..GQ.AK......V..RG R. DFPE.V.IA...G .. ....S..D.M.TI..N...R..I...IQTKITD K.

Nannospalax_galili .A.....S..SSY..GQ.AK......V..RG R. DFPE.VTIV...G .. ....S..D.MNTI..N...R..I...IQTKITD K.

Octodon_degus .A.....S..SSY..GQ.AK......V..RG R. DFPE.VTIV...R .. ....S..D.MNT...H......I...IQTKITD ..

Onychomys_torridus .A.....S..SSY..GQ.AK......V..RG R. DFPE.VKIV..-G .. ....S..D.MNTI..N...R..I...IQTKITD K.

Peromyscus_leucopus .A.....S..SSY..GQ.AK......V..RG R. DFPE.VKIV..-G .. ....S..D.MNTI..N...R..I...IQTKITD K.

Peromyscus_maniculatus .A.....S..SSY..GQ.AK......V..RG R. DFPE.VKIV..-G .. ....S..D.MNTI..N...R..I...IQTKITD K.

Rattus_norvegicus .A.....S..SSY..GQ.AK......V..RG R. DFPE.V.IA...G .. ....S..D.MNTI..N...R..I...IQTKITD K.

Rattus_rattus .A.....S..SSY..GQ.AK......V..RG R. DFPE.V.IA...G .. ....S..D.MNTI..N...R..I...IQTKITD K.

Sciurus_vulgaris .A.....S..SSY..GQ.AK......V..R. R. DFPE.VTIV...R .. ....S..D.MNTI..N...R..I...IQTKITD ..

Spermophilus_dauricus .A.....S..SSY..GQ.AK......V..R. R. DFPE.VTIV...R .. ....S..D.MNT...N...R..I...IQTKITD ..

Urocitellus_parryii .A.....S..SSY..GQ.AK......V..R. R. DFPE.VTIV...R .. ....S..D.MNT...N...R..I...IQTKITD ..

**Supplementary Figure 5. Alignment of mammalian proglucagon (Gcg) protein sequences**.

Predicted proglucagon (Gcg) amino acid sequences from 162 mammals are based on the MAFFT [51] aligned coding sequences. Sequences are shown in single letter amino acid code, with identical residues indicated by a period (.) and gaps by dashes (-). Residues that are unknown, due to the missing exon 6 sequence (in *Choloepus didactylus*, *Sturnira hondurensis*, and *Arvicola amphibius*) are indicated by ? The predicted signal peptide, GRPP (glicentin-related polypeptide), glucagon, intervening peptide-1 (IP-1), glucagon-like peptide-1 (GLP-1), IP-2, and GLP-2 sequences are indicated above the sequences, with the extent of the mature glucagon, GLP-1, and GLP-1 hormone sequences indicated by << and >>. ## indicates the N-terminus of the extended 37 residue-long precursor GLP-1 (labeled as N above the sequence), while ^^ indicates the N-terminus of the 31 residue long active form. Signal peptides for each precursor predicted by SignalP 5.0 [61] while prohormone processing sites were predicted using NeuroPred [62] and are indicted in bold in the *Ornithorhynchus anatinus* sequence. The substitutions at the C-terminal GLP-1 prohormone processing sites in the *Myotis brtandtii* and *Myotis lucifugus* sequences that might alter processing are in red and bold.
