# Supplementary material for: Variation in the Evolution and Sequences of Proglucagon and the Receptors for Proglucagon-Derived Peptides in Mammals
Source: Front Endocrinol (Lausanne). 2021 Jul 12;12:700066. doi: 10.3389/fendo.2021.700066 (PMC8312260; doi:10.3389/fendo.2021.700066)
Supplement: Supplementary File 1 — Fasta formatted proglucagon (Gcg) coding sequences. [file DataSheet_1.zip › Supplement/Suplementary Figures/Fig S7 Glp1r alignment.docx]

Signal peptide <<<

P PP PP P P PP

Ornithorhynchus_anatinus --------MP LPPQPLLFLL LLLLLLLLGT LGRAGPRSQG PPVSLSATLH KWREYRQQCL RLLWETPSPA AGVTVCNRTF DDYACWPDGV PGTFVNVTC

Monodelphis_domestica --------.A S..V..RLT- ----.....A V......P.. AYM...E.VQ .....Q.E.Q ..IQ.I.L.T T.G.F..... .S.......L ...Y...S.

Phascolarctos_cinereus --------.A S..A..RLT- ----...... V......P.. ATG...E.VY ......LK.E QFIQ...L.. T.DIF..... .N.T.....L ...Y...S.

Sarcophilus_harrisii --------.A S..A..R.T- ----...... V.....HP.- -VQ.F.E.IY ......LK.E QSIQ...L.. T.GIF..... .S.......L ...Y...S.

Trichosurus_vulpecula MTDGLDKG.- ---------- ---------- --------E. ATI.F.E.VY ......LK.E QF.Q...L.. T.DVF..... .N.T.....L ...Y...S.

Choloepus_didactylus --------.A GA.G..RLA- ----.....A V......P.. AT....E.VQ ......R..Q .F.S.A.P.. S.-LF..... .........P ..S....S.

Elephantulus_edwardii --------.A GA.G..RLA- ----.....A V......P.. AA....E.VQ .....QR..Q HF.S.A.P.. T.-LY..... .........P ..S....S.

Orycteropus_afer --------.A VA.G..RLA- ----.....A V......P.. AT....E.VQ ......R..Q .F.N.A.P.. T.-LF..... .........P ..S....S.

Balaenoptera_musculus --------.A GA.G..RLA- ----.....A V......P.. AT....E.VE ......R..Q .F.T.A.P.. T.-LF..... .........P ..S....S.

Delphinapterus_leucas --------.A GA.G..RLA- ----.....A V......P.. AT....E.VE ......R..Q .F.T.A.A.. T.-LF..... .........P ..S....S.

Globicephala_melas --------.A GA.G..RLA- ----.....A V......P.. AT....E.VE .....QR..Q .F.T.A.P.. T.-LF..... .........P ..S....S.

Lagenorhynchus_obliquidens --------.A GA.G..RLA- ----.....A V......P.. AT....E.VE .....QR..Q .F.T.A.P.. T.-LF..... .........P ..S....S.

Lipotes_vexillifer --------.A GA.G..RLA- ----.....A V......P.. AT....E.VE ......R..Q .F.T.A.P.. T.-LF..... .........P ..S....S.

Monodon_monoceros --------.A GA.G..RLA- ----.....A V......P.. AT....E.VE ......R..Q .F.T.A.P.. T.-LF..... .........P ..S....S.

Orcinus_orca --------.A GA.G..RLA- ----.....A V......P.. AT....E.VE .....QR..Q .F.T.A.P.. T.-LF..... .........P ..S....S.

Phocoena_sinus --------.A GA.G..RLA- ----.....A V......P.. AT....E.VE ......R..Q .F.T.A.P.. T.-LF..... .........P ..S....S.

Physeter_catodon --------.A GA.R..RLA- ----.....A V...C..P.. AT....E.VE ......R..Q .F.T.A.A.. T.-LF..... .........P ..S....S.

Bos_taurus --------.A GA.G..RLA- ----.....A V......P.. AT....E.VQ ......R..Q .F.T.A.P.. .D-LF..... .........S ..S....S.

Bubalus_bubalis --------.A GA.G..RLA- ----.....A V......P.. AT....E.VQ ......R..Q .F.T.A.P.. .D-LF..... .........S ..S....S.

Camelus_ferus --------.A GA.W..RLA- ----.....A V......P.. AT....E.VQ ......R..Q .F.S.A.L.. T.-LF..... .........P ..S....S.

Capra_hircus --------.A GA.G..RLA- ----.....A V......P.. AT....E.VQ ......R..Q .F.T.A.P.. .D-LF..... .........S ..S....S.

Catagonus_wagneri --------.A SA.G..RLA- ----.....A V......P.. AT....E.VQ ......R..Q .F.A.A.P.. T.-LF..... .........P ..S....S.

Cervus_hanglu --------.A GV.G..RLA- ----.....A V......P.. AT....E.VR ......R..Q HF.T...P.. TD-LF..... .........S ..S....S.

Moschus_moschiferus --------.A GA.G..RLA- ----.....A V......P.. A.....E.VQ ......R..Q .F.T.A.P.. .D-LF..... .........S ..S....S.

Sus_scrofa --------.A GA.G..RLA- ----.....A V......P.. TT....E.VQ ......R..Q .F.T.A.P.. T.-LF..... .........P ..S....S.

Ailuropoda_melanoleuca --------.A RA.S..CLA- ----.....A V......P.. AT....E.VQ ......H..Q .F.T.A.P.. TA-LF..... .E.......L ..S....S.

Callorhinus_ursinus --------.A RA.S..CLA- ----.....A V......P.. A.....E.VQ ......H..Q .F.T.A.P.. T.-LF..... .E.......L ..S....S.

Canis_lupus --------.A RA.S..CLA- ---......A .RT....P.. AT....E.VQ ......H..Q .F.T...P.. T.-LF..... .E.......L ..S....S.

Enhydra_lutris --------.A RA.S..CLA- ----.....A V..V...P.. AT....E.VQ ......H..Q .F.T.A.T.. T.-LF..... .E.......L ..S....S.

Felis_catus --------.A GA.S..CLA- ----.....A V......P.. AT....E.VQ ......H..Q .F.T.A.P.. T.-LF..... .E.......L ..S....S.

Halichoerus_grypus --------.A RA.S..CLA- ----.....A V......P.. A.....E.VQ ......H..Q .F.T.A.P.. T.-LF..... .E.......L ..S....S.

Lontra_canadensis --------.A RA.S..CLA- ----.....A V..V...P.. AT....E.VQ ......H..Q .F.T.A.T.. T.-LF..... .E.......L ..S....S.

Mirounga_leonina --------.A RA.S..CLA- ----.....A V......P.. A.....E.VQ ......H..Q .F.T.A.P.. T.-LF..... .E.......L ..S....S.

Mustela_erminea --------.A RA.S..CLA- ----.....A V..V...P.. AT....E.VQ ......H..Q .F.T.A.P.. T.-LF..... .E.......L ..S....S.

Neomonachus_schauinslandi --------.A RA.S..CLA- ----.....A V......P.. A.....E.VQ ......H..Q .F.T.A.P.. T.-LF..... .E.......L ..S....S.

Neovison_vison --------.A RA.S..CLA- ----.....A V..V...P.. AT....E.VQ ......H..Q .F.T.A.P.. T.-LF..... .E.......L ..S....S.

Odobenus_rosmarus --------.A RA.S..CLA- ----.....A V......P.. A.....E.VQ ......H..Q .F.T.A.P.. T.-LF..... .E.......L ..S....S.

Panthera_leo --------.A GA.S..CLA- ----.....A V......P.. AT....E.VQ ......H..Q .F.T.A.P.. T.-LF..... .E.......L ..S....S.

Panthera_pardus --------.A GA.S..CLA- ----.....A V......P.. AT....E.VQ ......H..Q .F.T.A.P.. T.-LF..... .E.......L ..S....S.

Phoca_vitulina --------.A RA.S..CLA- ----.....A V......P.. A.....E.VQ ......H..Q .F.T.A.P.. T.-LF..... .E.......L ..S....S.

Suricata_suricatta --------.A GA.S..SLA- ----.....A V......P.. AT....E.VQ ......H..Q .F.T.A.P.. T.-LF..... .E.......L ..S....S.

Ursus_thibetanus --------.A RA.S..CLA- ----.....A V......P.. AT....E.VQ ......H..Q .F.T.A.P.. T.-LF..... .E.......L ..S...---

Zalophus_californianus --------.A RA.S..CLA- ----.....A V......P.. A.....E.VQ ......H..Q .F.T.A.P.. T.-LF..... .E.......L ..S....S.

Artibeus_jamaicensis --------.A RA.G..RLA- ----...... V.M....P.. AT....K.VQ ......R..Q .F.I...P.. T.-LF..... .........S ..S....S.

Molossus_molossus --------.A GA.G..RLA- ----.....V V......P.. AI....E.VQ ..Q..Q...Q .F.E.V.A.. T.-LF..... .G.......P .DS....S.

Myotis_myotis --------.A GA.G..RLA- ----.....A V......P.. AT....E.VQ ......H..Q .F.T.A.P.. TD-LF..... .........P ..S....S.

Phyllostomus_discolor --------.A RA.G..RLA- ----...... V......P.. AT....E.VQ ......R..Q .F.I.A.P.. T.-LF..... .........P ..S...IS.

Pipistrellus_kuhlii --------.A GA.GL.RLA- ----....AA V......P.. AT....E.VQ ......H..Q .F.T.A.P.. TD-LF..... .........P ..S....S.

Pteropus_vampyrus --------.A GA.G..RLA- ----.....A V......P.. AT....E.VQ ......H..Q LF.T.A.P.. T.-LF..... .........L ..S....S.

Rhinolophus_ferrumequinum --------.A GA.G..RLA- ----.....A V......P.. AT....E.VQ ......R..Q .F.T.A.P.. T.-LF..... .........L ..S....S.

Rousettus_aegyptiacus --------.A GA.A-.RLA- ----.....? V......P.. AT....E.VQ ......R..Q .F.T.A.P.. T.-LF..... .........L ..S....S.

Sturnira_hondurensis --------.A RA.G..RLA- ----...... V.M....P.. AT....E.VQ ......H..Q ...I...P.. T.-LF..G.. .........L .DS...IS.

Equus_asinus --------.A GA.G..RLA- ----.....A V......P.. AT..F.E..Q ..W...R..Q .F.M...P.. T.-LF..... .E.......L ..SL...S.

Equus_caballus --------.A GA.G..RLA- ----.....A V......P.. AT..F.E..Q ..W...R..Q .F.T...P.. T.-LF..... .E.......L ..SL...S.

Manis_pentadactyla --------.A GA.G..PLA- ----.....A V......P.. AS....E.VQ ......R..Q .F.T.A.P.. T.-LF..... ...V.....L ..S....S.

Prolemur_simus --------.T GA.G..RLA- ----.....A V......P.. AT....E.VQ ......R..Q .F.T.A.P.. T.-LF..... .........P ..S....S.

Cercocebus_atys --------.A GT.G..RLA- ----.....V V......P.. AT...WE.VQ ......R..Q .S.T.D.P.. TD-LF..... .E.......E ..S....S.

Chlorocebus_sabaeus --------.A GT.G..RLA- ----.....V V......P.. AT...WE.VQ ......R..Q .S.T.D.P.T TD-LF..... .E.......E ..S....S.

Colobus_angolensis --------.A GT.G..RLA- ----.....V V......P.. AT...WE.VQ ......R..Q .S.T.D.P.. TD-LF..... .E.......E ..S....S.

Gorilla_gorilla --------.A GA.G..RLA- ----.....M M......P.. AT...WE.VQ ......R..Q .S.T.D.P.. TD-LF..... .E.......E ..S....S.

Homo_sapiens --------.A GA.G..RLA- ----.....M V......P.. AT...WE.VQ ......R..Q .S.T.D.P.. TD-LF..... .E.......E ..S....S.

Hylobates_moloch --------.A GA.G...LA- ----.....M V......P.. AA...WE.VQ ......R..Q .S.T.D.P.. TD-LF..... .E.......E ..S....S.

Macaca_fascicularis --------.A GT.G..RLA- ----.....V V......P.. AT...WE.VQ ......R..Q .S.T.D.P.. TD-LF..... .E.......E ..S....S.

Macaca_mulatta --------.A GT.G..RLA- ----.....V V......P.. AT...WE.VQ ......R..Q .S.T.D.P.. TD-LF..... .E.......E ..S....S.

Macaca_nemestrina --------.A GT.G..RLA- ----.....V V......P.. AT...WE.VQ ......R..Q .S.T.D.P.. TD-LF..... .E.......E ..S....S.

Mandrillus_leucophaeus --------.A GT.G..RLA- ----.....V V......P.. AT...WE.VQ ......R..Q .S.T.D.P.. TD-LF..... .E.......E ..S....S.

Microcebus_murinus --------.A GA.G..RLA- ----.....A V......P.. AT....E.VQ ......R..Q .F.S.A.P.. T.-LF..... .........P ..S....S.

Nomascus_leucogenys --------.A GA.G...LA- ----.....M V......P.. AT...WE.VQ ......R..Q .S.T.D.P.. TD-LF..... .E.......E ..SL...S.

Otolemur_garnettii --------.A GT.G..RLA- ----.....A V......P.. AT....E.VQ ......R..Q LF.T...P.T T.-LF..... .........P A.S....S.

Pan_paniscus --------.A GA.G..RLA- ----.....M V......P.. AT...WE.VQ ......R..Q .S.T.D.P.. TD-LF..... .E.......E ..S....S.

Pan_troglodytes --------.A GA.G..RLA- ----.....M V......P.. AT...WE.VQ ......R..Q .S.T.D.P.. TD-LF..... .E.......E ..S....S.

Papio_anubis --------.A GT.G..RLA- ----.....V V......P.. AT...WE.VQ ......R..Q .S.T.D.P.. TD-LF..... .E.......E ..S....S.

Piliocolobus_tephrosceles --------.A GT.G..RLA- ----F....V V......P.. AT...WE.VQ ......RE.Q .S.T.D.P.T TD-LF..... .E.......E ..S....S.

Pongo_abelii --------.A GA.G..RLA- ----.....M V......P.. AT...WE.VQ ......R..Q .S.T.V.P.. TD-LF..... .E.......E ..S....S.

Propithecus_coquereli --------.A RA.G..RLA- ----.....A .......P.. AT....E.VQ ......R..Q .F.A.A.P.. P.-LF..... .........P ..S....S.

Rhinopithecus_bieti --------.A GT.G..RLA- ----.....V V......P.. AT...WE.VQ ......R..Q .S.T.D.P.T TD-LF..... .E.......E ..S....S.

Rhinopithecus_roxellana --------.A GT.G..RLA- ----.....V V......P.. AT...WE.VQ ......R..Q .S.T.D.P.. TD-LF..... .E.......E ..S....S.

Sapajus_apella --------.A GA.G..RLA- ----.....A V......P.. A....LE.MQ ..E..QR..Q .N.S.A.P.. T.-LF...S. .G.......P .SS....S.

Theropithecus_gelada --------.A GT.G..RLA- ----.....V V......P.. AT...WE.VQ ......R..Q .S.T.D.P.. TD-LF..... .E.......E ..S....S.

Trachypithecus_francoisi --------.A GT.G..RLA- ----.....V V......P.. AT...WE.VQ ......R..Q .S.T.D.P.. TD-LF..... .E.......E ..S....S.

Oryctolagus_cuniculus --------.A GA.S..RLA- ----.....A V......P.. AT....E.VQ ......R..Q HF.T.A.PL. T.-LF..... .........A ..S....S.

Arvicanthis_niloticus --------.A RT.SL.RLA- ----.....A V......P.. AR....E.VQ ......R..Q .F.T.A.LL. T.-LF..... .........P ..S....S.

Arvicola_amphibius --------.A GA.SL.RLA- ----.....A V......P.. AT....E.VQ ......R..Q .F.I.A.PL. T.-LF..... .........P ..S....S.

Castor_canadensis --------.A GA.SA.RLA- ----.....A V......P.. AT....E.VQ ......R..Q .F.I.A.P.. T.-LF..... .........S ..S....S.

Chinchilla_lanigera --------.A GA.S..RLA- ----.....A V......P.. AT....E.VQ ......R..Q .N.T.M.P.. T.-LF..... .........P ..S....S.

Cricetulus_griseus --------.A GA.SL.RLA- ----.....A V......P.. AT....E.VQ ......R..Q .F.T...PL. T.-LF..... .........P ..S....S.

Grammomys_surdaster --------.A RT.SL.RLA- ----.....A V......P.. AR....E.VQ ......R..Q .F.T.A.LL. T.-LF..... .........P ..S....S.

Heterocephalus_glaber --------.A GA.S..RLA- ----.....A V......P.. AT....E.VQ ......R... .N.T...P.. T.-LF..... .........P ..S....S.

Jaculus_jaculus --------.A RA.S..RLA- ----...I.A V......P.. AA....E.VQ ......R..Q .F.T.A.PL. T.-VF..... .........P ..S....S.

Marmota_flaviventris --------.A GA.S..RLA- ----.....A V......P.. AT....E.VQ ......R..Q .F.T.A.P.. T.-LF..... .........P ..S....S.

Mastomys_coucha --------.A GT.SL.RLA- ----.....A V......P.. TT....E.VQ ......R..Q .F.T.A.LL. T.-LF..... .........P ..S....S.

Meriones_unguiculatus --------.A GI.SL.RLA- ----.....A VS.....P.. AT....E.VQ ......R..Q .F.T...LM. T.-LF..... .........P ..S....S.

Mesocricetus_auratus --------.A GA.SL.RLA- ----.....A V......P.. AT....E.VQ ......R..Q .F.T.A.PL. T.-LF..... .........P ..S....S.

Microtus_ochrogaster --------.A GA.SL.RLA- ----.....A V......P.. AT....E.VQ ......R..Q .F.T.A.PL. T.-LF..... .........P ..S....S.

Mus_caroli --------.A ST.SL.RLA- ----.....A V......P.. TT....E.VQ ......R..Q .F.T.A.LL. T.-LF..... .........P ..S....S.

Mus_musculus --------.A ST.SL.RLA- ----.....A V......P.. TT....E.VQ ......R..Q .F.T.A.LL. T.-LF..... .........P ..S....S.

Mus_pahari --------.A ST.SL.HLA- ----.....A V......P.. TT....E.VQ ......R..Q .F.T.A.LL. T.-LF..... .........P ..S....S.

Mus_spicilegus --------.A ST.SL.RLA- ----.....A V......P.. TT....E.VQ ......R..Q .F.T.A.LL. T.-LF..... .........P ..S....S.

Nannospalax_galili --------.A GA.S..HLA- ----.....A V......P.. AT....E.VQ ......R..Q .F.T...PL. ..-LF..... .........P ..S....S.

Onychomys_torridus --------.A GA.SL.RLA- ----.....A V.K....P.. AT....E.VQ ......R..Q .F.T.A.PL. T.-LF..... .........P ..S....S.

Peromyscus_leucopus --------.A GA.SL.RLA- ----.....A V.K....P.. AT....E.VQ ......R..Q .F.A.A.PL. ..-LF..... .........P ..S....S.

Rattus_norvegicus --------.A VT.SL.RLA- ----.....A V......P.. AT....E.VQ ......H..Q .F.T.A.LL. T.-LF..... .........P ..S....S.

Rattus_rattus --------.A VT.SL.RLA- ----.....A V......P.. AT....E.VQ ......H..Q .F.T.A.LL. T.-LF..... .........P ..S....S.

Sciurus_vulgaris --------.A GA.S..RLA- ----.....A V......P.. AT....E.VQ ......R..Q .F.T.A.P.. T.-LF..... .........P .--------

Urocitellus_parryii --------.A GA.S..RLA- ----.....A V......P.. AT....E.VQ ......R..Q .F.T.A.P.. T.-LF..... .........P ..S....S.

1.50 12.50 2.50

>>> TM1 **$** <<< **$** > >> **$**

PPPP P P P PP P PP P P G G

Ornithorhynchus_anatinus PWYLPWASRV SHGQVYRFCT LEGTWLQEKN ATLTWRNLTE CEDAEKGQWS PPEEQFLFLS IIYTVGYALS FSALIVATAI LLQFRHLHCT RNYIHLNLF

Monodelphis_domestica ........S. HQ.H...... T..R..Y... SS.P.IDVS. ..ENKT.ER. H....L.SF. .V....H... ....VI.... ..R....... .........

Phascolarctos_cinereus ........S. .Q.H...... S..I...D.. SSMP..D.S. .-ENKS-EE. H....L.SFT V......... ....VI.... ..R....... .........

Sarcophilus_harrisii ........S. .Q.H...... T.....HDD. SS.P..D.S. .-ENKS.NK. H....L.SFT .......... ....LI.... ..R....... .........

Trichosurus_vulpecula ........S. .Q.H...... T..L...D.. SSMP..D.S. .-EV.S-EE. H....L.SFT V......... ....VI.... ..R....... .........

Choloepus_didactylus ........S. LQ.H...... A..L..HKD. SS.P..D.S. ...SKR.ER. .....L.S.Y .......... ....VI.S.. ..G....... .........

Elephantulus_edwardii ........S. LQ.H...... A..L..HKD. SS.P..D.S. ..ESKR.ER. .L...L.S.Y V......... ....VI.S.. ..G....... .........

Orycteropus_afer ........S. LQ.H...... A..L..HKD. SS.P..D.S. ..ESKR.ERN .L...L.S.Y .......... ....VI.S.. ..G....... .........

Balaenoptera_musculus ........S. LQ.H...... TD.L..HKD. SS.P..D.S. ..ESKR.DRN S..Q.L.S.Y .......... ....VI.S.. ..G....... .........

Delphinapterus_leucas ........S. LQ.H...... TD.L..HKD. SS.P..D.S. ..ESKR.DQN S..Q.L.S.Y .......... ....VI.S.. ..G....... .........

Globicephala_melas ........S. LQ.H...... TD.L..HKD. SS.P..D.S. ..ESKR.DQN S..Q.L.S.Y V......... ....VI.S.. ..G....... .........

Lagenorhynchus_obliquidens ........S. LQ.R...... TD.L..HKG. SS.P..D.S. ..ESKR.DQN S..Q.L.S.Y V......... ....VI.S.. ..G....... .........

Lipotes_vexillifer ......S.S. LQ.H...... TD.L..HKD. SS.P..D.S. ..ESKR.DQ. S..Q.L.S.Y .......... ....VI.S.. ..G....... .........

Monodon_monoceros ........S. LQ.H...... TD.L..H.D. SS.P..D.S. ..ESKR.DQN S..Q.L.S.Y .......T.. ....VI.S.. ..G....... .........

Orcinus_orca ........S. LQ.H...... TD.L..HKD. SS.P..D.S. ..ESKR.DQN S..Q.L.S.Y V......... ....VI.S.. ..G....... .........

Phocoena_sinus ........S. LQ.H...... TD.L..HKD. SS.P..D.S. ..ESKR.DQN S..Q.L.S.Y .......... ....VI.S.. ..G....... .........

Physeter_catodon ........S. LQ.H...... TD.L..HKD. SS.P..D.S. ..ESKR.DRN S..Q.L.S.Y .......... ....VI.S.. ..G....... .........

Bos_taurus ........S. LQ.H...... AD.L..HKD. SS.P..D.S. ...SKR.DR. S..Q.L.S.Y V......... ....VI.S.. ..G....... .........

Bubalus_bubalis ........S. LQ.H...... AD.L..HKE. SS.P..D.S. ...SKR.DR. S..Q.L.S.Y V......... ....VI.S.. ..G....... .........

Camelus_ferus ........S. LQ.H...... AD.L..HKD. SSRP..D.S. ..ESKR.DR. S...HL.S.Y V......... ....VI.S.. ..G....... .........

Capra_hircus ........S. LQ.H...... AD.L..HKD. SS.P..D.S. ...SKR.DR. S..R.L.S.Y VV........ ....VI.S.. ..G....... .........

Catagonus_wagneri .....----- LQ.H...... AD.L..H.D. SS.P..D.S. ..ESKR.DR. S....L.S.Y V......... ....VI.S.. ..G....... .........

Cervus_hanglu .......NS. LQ.H...... AD.L..HKD. SS.P..D.S. ...SKR.DR. S..R.L.S.Y V......... ....V..S.. ..G....... .........

Moschus_moschiferus ....------ LQ.H...... AD.L..HKD. SS.P..D.S. ...SKR.DR. S..R.L.S.Y A......... ....VI.S.. ..G....... .........

Sus_scrofa ........S. LQ.H...... AD.L..HKD. SS.P..D.S. ..ESKH.DR. S....L.S.Y V......... ....VI.S.. ..G....... .........

Ailuropoda_melanoleuca ........S. LQ.R...... A..L..RQD. SSAP....S. ..ESKQ.ER. S..Q.L.SF. .......T.. ....VI.S.. ..S....... .........

Callorhinus_ursinus ........S. LQ.R...... A..L..RQE. SSSP....S. ..ESKR.ERH S....L.SF. .......T.. ....VI.S.. ..S....Y.. .........

Canis_lupus ........S. LQ.H...... A..L..RQH. SS.P....S. ..ESKR.ER. S....L.SF. .......T.. ....VI.S.. ..S....... .........

Enhydra_lutris ........S. LQ.R...... A..L..RQD. SSSP....S. ..ESKR.ER. S....L.SF. .......T.. ....VI.S.. ..S....... .........

Felis_catus ........S. LQ.H...... A..L..RQD. SS.P....S. ..ESKR.ER. S....L.SF. .......T.. ....VI.S.. ..S....... .........

Halichoerus_grypus ........S. LQ.RA..... A..L.RRQD. SSSP....S. ..ESKR.ER. S....L.SF. .......T.. ....VI.S.. ..S....Y.. .........

Lontra_canadensis ........S. LQ.R...... A..L..RQD. SSSP....S. ..ESKP.ER. S....L.SF. .......T.. ....VI.S.. ..S....... .........

Mirounga_leonina ........S. LQ.R...... A..L..RQD. SSSP....S. ..ESKR.ER. S....L.SF. V......T.. ....VI.S.. ..S....Y.. .........

Mustela_erminea ........S. LQ.R...... A..L..RQD. SSSP....S. ..ESKR.ER. S....L.SF. .......T.. ....VI.S.. ..S....... .........

Neomonachus_schauinslandi ........S. LQ.R...... A..L..RQD. SSSP....S. ..ESKR.ER. S....L.SF. .......T.. ....VI.S.. ..S....Y.. .........

Neovison_vison ---------L LQ.R...... A..L..RQD. SSSP....S. ..ESKR.ER. S....L.SF. .......T.. ....VI.S.. ..S....... .........

Odobenus_rosmarus ........S. LQ.R...... A..L..RQE. SSSP....S. ..ESKR.ARR S....L.SF. .......T.. ....VI.S.. ..S....Y.. .........

Panthera_leo ..-------- LQ.H...... A..L..RQD. SS.P....S. ..ESKR.ER. S....L.SF. .......T.. ....VI.S.. ..S....... .........

Panthera_pardus ........S. LQ.H...... A..L..RQD. SS.P....S. ..ESKR.ER. S....L.SF. .......T.. ....VI.S.. ..S....... .........

Phoca_vitulina ........S. LQ.RA..... A..L.RRQD. SSSP....S. ..ESKR.ER. S....L.SF. .......T.. ....VI.S.. ..S....Y.. .........

Suricata_suricatta ........S. LQ.H...... A..L..RQD. SS.P....S. ..ESKR.ER. S....L.SF. .......T.. ....VI.S.. ..S....... .........

Ursus_thibetanus ---------. LQ.R...... A..L..RQD. SSAP....S. ..ESKQ.ER. S..Q.L.SF. .......T.. ....VI.S.. ..S....... .........

Zalophus_californianus ........S. LQ.R...... A..L..RQE. SSSP....S. ..ESKR.ERH S....L.SF. .......T.. ....VI.S.. ..S....Y.. .........

Artibeus_jamaicensis .......N.. LQ.RT..... AD.L..HKD. SS.P..D.S. ..EPKRRER. SL.D.L.A.Y .......... ....VI.S.. ..G....... .........

Molossus_molossus .......DS. LQ.H..Q... AD.L..HKD. SS.P..D.S. .AESKG.DR. YL.D.H.S.Y .......... ....VI.S.. ..R....... .........

Myotis_myotis .......NS. LQ.H...... AD.L..HQD. SS.P..D.S. ..ESKR.DR. S..D.L.S.Y .V........ ....VI.S.. ..G....... .........

Phyllostomus_discolor .......DS. LQ.H...... AD.L..HKD. SS.P..DVS. ..ESKR.ER. .L.D.L.S.Y .......... ....VI.S.. ..G....... .........

Pipistrellus_kuhlii .......NS. LQ.H...... AD.L..HQV. SS.P..D.S. ..ESKR.DR. S..D.L.S.Y .V........ ....VI.ST. ..G....... .........

Pteropus_vampyrus .......NS. LQ.H...... VD.L..YKD. SS.P..D... ..ESKR.ER. S..D.L.S.Y .......... ....VI.S.. ..S....... .........

Rhinolophus_ferrumequinum .......NS. LQ.H...... AD.L..YKE. SS.P..D.S. ..ESKR.ER. S..D.L.S.Y .......... ....VI.S.. ..G....... .........

Rousettus_aegyptiacus .......NS. LQ.H...... AD.L..YKD. SS.P..D.S. ..ESKR.ER. S..D.L.S.Y V......... ....VI.S.. ..S....... .........

Sturnira_hondurensis .......N.. LQ.RM..... AD.L..HKD. SSQP..D.S. ..EPKRRER. SM.D.L.S.Y .......... ....VI.S.. ..G....... .........

Equus_asinus ........N. LQ.H...... AD.L..HVD. SS.P....S. .D.SKG.ER. F..K.L.S.Y F......... ....VI.S.. ..G....... .........

Equus_caballus ........N. LQ.H...... AD.L..HVD. SS.P....S. .D.SKG.ER. F..K.L.S.Y F......... ....VI.S.. ..G....... .........

Manis_pentadactyla ........S. LQ.H...... AD.L..HQD. SSMP....S. ..ESKR.ER. F....L.S.. V......... ....VI.S.. ..S....R.. .........

Prolemur_simus ........S. LQ.H...... A..L..RKD. SS.P..D.S. ..ESKR.ER. S....L.S.Y .......S.. .C..VI.S.. ..G....R.. .........

Cercocebus_atys ........S. PQ.H...... A..L...KD. SS.P..D.S. ..ESKR.ER. .....L.S.Y .......... ....VI.S.. ..G....... .........

Chlorocebus_sabaeus ........S. PQ.H...... A..L...KD. SS.P..D.S. ..ESKR.ER. S....L.S.Y .......... ....VI.S.. ..G....... .........

Colobus_angolensis ........S. PQ.H...... A..L...KD. SS.P..D.S. ..ESKR.ER. S....L.S.Y .......... ....VI.S.. ..G....... .........

Gorilla_gorilla ........S. PQ.H...... A..L...KD. SS.P..D.S. ..ESKR.ER. S....L...Y .......... ....VI.SV. ..G....... .........

Homo_sapiens ........S. PQ.H...... A..L...KD. SS.P..D.S. ..ESKR.ER. S....L...Y .......... ....VI.S.. ..G....... .........

Hylobates_moloch ........S. PQ.H...... A..L...KD. SS.P..D.S. ..ESKR.ER. S....L.S.Y .......... ....VI.S.V ..G....... .........

Macaca_fascicularis ........S. PQ.H...... A..L...KD. SS.P..D.S. ..ESKR.ERN S....L.S.Y .......... ....VI.S.. ..G....... .........

Macaca_mulatta ........S. PQ.H...... A..L...KD. SS.P..D.S. ..ESKR.ERN S....L.S.Y .......... ....VI.S.. ..G....... .........

Macaca_nemestrina ........S. PQ.H...... A..L...KD. SS.P..D.S. ..ESKR.ERN S....L.S.Y .......... ....VI.S.. ..G....... .........

Mandrillus_leucophaeus ........S. PQ.H...... A..L...KD. SS.P..D.S. ..ESKR.ER. .....L.S.Y .......... ....VI.S.. ..G....... .........

Microcebus_murinus ........S. LQ.H...... A..L..HKD. SS.P..D.S. ..ESKR.ER. S....L.S.Y .......... .C..V..S.. ..G....R.. .........

Nomascus_leucogenys ........S. PQ.H...... A..L...KD. SS.P..D.S. ..ESKR.ER. S....L.S.Y .......... ....VI.S.. ..G....... .........

Otolemur_garnettii ........S. LQ.H...... A..L..RKD. SSIP..D.S. ..ESKR.ER. S....L.S.Y .......... ....VI.S.. ..G....R.. .........

Pan_paniscus ........S. PQ.H...... A..L...KD. SS.P..D.S. ..ESKR.ER. S....L...Y .......... ....VI.S.. ..G....... .........

Pan_troglodytes ........S. PQ.H...... A..L...KD. SS.P..D.S. ..ESKR.ER. S....L...Y .......... ....VI.S.. ..G....... .........

Papio_anubis ........S. PQ.H...... A..L...KD. SS.P..D.S. ..ESKR.ER. .....L.S.Y .......... ....VI.S.. ..G....... .........

Piliocolobus_tephrosceles ........S. PQ.H...... A..L...KD. SS.P..D.S. ..ESKR.ER. S....L.S.Y .......... ....VI.SV. ..G....... .........

Pongo_abelii ........S. PQ.H...... A..L...KD. SS.P..D.S. ..ESKR.ERN S....L...Y .......... ....VI.SS. ..G....... .........

Propithecus_coquereli ........S. LQ.H...... A..L..HKD. SS.P..D.S. ..ESKR.ER. S....L.S.Y .V........ .C..VI.S.. ..G....R.. .........

Rhinopithecus_bieti ........S. PQ.H...... A..L...KD. SS.P..D.S. ..ESKR.ER. S....L.S.Y .......... ....VI.S.. ..G....... .........

Rhinopithecus_roxellana ........S. PQ.H...... A..L...KD. SS.P..D.S. ..ESKR.ER. S....L.S.Y .......... ....VI.S.. ..G....... .........

Sapajus_apella ........S. LQ.H...... A..L..HKD. SS.P..D.S. .LESKR.ER. S..V.L.S.Y .......... ....VI.S.. ..G....... .........

Theropithecus_gelada ........S. PQ.H...... A..L...KD. SS.P..D.S. ..ESKR.ER. .....L.S.Y .......... ....VI.S.. ..G....... .........

Trachypithecus_francoisi ........S. LQ.H...... A..L...KD. SS.P..D.S. ..ESKR.ER. S....L.S.Y .......... .T..VI.S.. ..G....... .........

Oryctolagus_cuniculus ........N. LQ.H...... T..H..HKD. SS.P..D.S. ..ESRR.EK. S....L.S.Y .......... ....VI.S.. ..G....... .........

Arvicanthis_niloticus ........S. LQ.H...... A..L..HKD. SS.P..D.S. ..ESKR.ERN S....L.S.Y .......... ....VI.S.. .VG....... .........

Arvicola_amphibius ........S. LQ.H...... A..L..HKD. SS.P..D.S. ..ETKR.ERN S....L.S.Y .......... ....VI.S.. .VG....... .........

Castor_canadensis ........S. LQ.H...... A..L..HKD. SS.P..D.S. ..ESKR.EK. SL...L.S.Y V......... ....VI.S.. ..S....... .........

Chinchilla_lanigera ........S. LQ.H...... P..L..HRD. SS.P..D.S. ..ESKR.EK. S....L.S.Y V......... ....VI.S.. ..S....... .........

Cricetulus_griseus ........S. LQ.H...... A..L..HKD. SS.P..D.S. ..ESKR.ERN S....L.S.Y .......... ....VI.S.. .VG....... .........

Grammomys_surdaster .......NS. LQ.H...... A..L..HKD. SS.P..D.S. ..ESKR.ERN S....L.S.Y .......... ....VI.S.. .VG....... .........

Heterocephalus_glaber ........S. LQ.H...... P..L..HRD. SS.P..D.S. ..ESQR.EK. S....L.S.Y .......... ..T.VI.S.. ..S....... .........

Jaculus_jaculus ........S. LQ.H...... AD.L..HKD. SS.P..D.S. ..ESKR.EKN S....L.S.Y .......... ....VI.S.. ..G....... .........

Marmota_flaviventris ........S. PQ.H...... A..L..HKD. SSQP..D.S. ..ESKR.EK. S....L.S.Y V......... ....VI.S.. ..S....... .........

Mastomys_coucha ........S. LQ.H...... A..L..HKD. SS.P..D.S. ...SKR.ERN F....L.S.Y .......... ....VI.S.. .VG....... .........

Meriones_unguiculatus ........S. LQ.HA..... A..L..HKD. SS.P..D.S. ..ESKR.ER. S....L.S.Y V......... ....LI.S.. .VG....... .........

Mesocricetus_auratus ........S. LQ.H...... A..L..HKE. SS.P..D.S. ..ESKR.ER. S....L.S.Y .......... ....VI.S.. .VG....... .........

Microtus_ochrogaster ........S. LQ.H...... A..L..HKD. SS.P..D.S. ..ESKR.ERN S....L.S.Y .......... ....VI.S.. .VG....... .........

Mus_caroli ........S. LQ.H...... A..L..HKD. SS.P..D.S. ..ESKR.ERN F....L.S.Y .......... ....VI.S.. .VG....... ..H......

Mus_musculus ........S. LQ.H...... A..L..HKD. SS.P..D.S. ..ESKR.ERN F....L.S.Y .......... ....VI.S.. .VG....... .........

Mus_pahari ........S. LQ.H...... A..L..HKD. SS.P..D.S. ..ESKR.ERN F....L.S.Y .......... ....VI.S.. .VG....... .........

Mus_spicilegus ........S. LQ.H...... A..L..HKD. SS.P..D.S. ..ESKR.ERN F....L.S.Y .......... ....VI.S.. .VG....... .........

Nannospalax_galili ........T. LQ.H...... A..L..HKD. SSMP..D.S. ..ESKR.ER. S....L.S.Y .......... ....VI.S.. ..G....... .........

Onychomys_torridus ........S. LQ.H...... A..L...KD. SS.P..D.S. ..ESKR.ERN S....L.S.Y .......... ....VI.S.. .VG....... .........

Peromyscus_leucopus ........S. LQ.H...... A..L...KD. SS.P..D.S. ..ESKR.ERN S....L.S.Y .......... ....VI.S.. .VG....... .........

Rattus_norvegicus ........S. LQ.H...... A..I..HKD. SS.P..D.S. ..ESKQ.ERN S....L.S.Y .......... ....VI.S.. .VS....... .........

Rattus_rattus ........S. LQ.H...... A..I..HKD. SS.P..D.S. ..ESKQ.ERN S....L.S.Y .......... ....VI.S.. .VG....... .........

Sciurus_vulgaris ---------. LQ.H...... A..L..HKN. SS.P..D.S. ..ESKR.EKG S....L.S.Y .......... .C..VI.S.. ..S....... .........

Urocitellus_parryii ........S. PQ.H...... A..L..HKD. SSQP..D.S. ..ESKR.EKD S....L.S.Y .......... ....VI.S.. ..S....... .........

3.50 4.50

TM2 <<< >>> TM3 **$** <<< >> > TM4 **$**

P P P P P P PP P P P P PP P P G GG G G G GGG

Ornithorhynchus_anatinus TSFILRALSV FIKDTVLKWM YNTATQQHQW EGFLAYQESL GCRLVFLMMQ YCVAANYYWL LVEGVYLYTL LVLSVFSEQR IFRIYLCIGW GVPMMFVIP

Monodelphis_domestica M......M.I ....D..... .S...RS... ..L.S..... S......... .......... ....I..... ........R. V..L.V.... .I.LL....

Phascolarctos_cinereus M......... .VR.A..... .G...RH... D.L.S..... S.....I... .......... ....I..... .......... V..L.IF... ...LL....

Sarcophilus_harrisii M......... ..R.A.M... .G...RRY.. D.L.S..... S.....I... .......... ....I..... .......... V..L.VF... ...LL....

Trichosurus_vulpecula M......... ..R.A.M... .G....H... D.L.S..... S.....I... .......... ....I..... .......... V..L.VF... ...LL....

Choloepus_didactylus V......... ....AA.... .S..AP.... D.L.S..D.. .......L.. .......... .......... .A.P...... ...L..SM.. ...LL....

Elephantulus_edwardii A......... ....AA.... .S..A..... D.L.S..D.. .......L.. ...V...... ........K. .AF....... V..L..S... ...LL....

Orycteropus_afer A......... ....AA.... .S..A..... D.L.S..D.V .......L.. .......... .......... .AF....... V..L..T... ...LL....

Balaenoptera_musculus A......... .V..AA.... .S..A..... D.L.S..D.. .......L.. .......... .......... .A........ ...L..S... ...LL....

Delphinapterus_leucas A......... ....AA.... .S..A..... D.L.S..D.. ....A..L.. .......... .......... .AFA...... ...L..S... ...LL....

Globicephala_melas A......... ....AA.... .S..A..... D.L.S..D.. ....A..L.. .......... .......... .AFA...... ...L..S... ...LL....

Lagenorhynchus_obliquidens A......... ....AA.... .S..A..... D.L.S..D.. ...VA..L.. .......... .......... .AFA...... ...L..S... ...LL....

Lipotes_vexillifer A......... ....AA.... .S..A..... D.L.S..D.. ....A..L.. .......... .......... .A........ ...L..S... ...LL....

Monodon_monoceros A......... ....AA.... .S......R. D.L.S..D.. ....A..L.. .......... .......... .AFA...... ...L..S... ...LL....

Orcinus_orca A......... ....AA.... .S..A..... D.L.S..D.. ....A..L.. .......... .......... .AFA...... ...L..S... ...LL....

Phocoena_sinus A......... ....AA.... .S..A..... D.L.S..D.. ....A..L.. .......... .......... .AFA...... ...L..S... ...LL....

Physeter_catodon A......... ....AA.... .S..A..... D.L.S..D.. .......L.. .......... .......... .A........ ...L..S... ...LL....

Bos_taurus A......... .......... .S..A..... D.L.S..... .......L.. .......... ........S. .A........ V..L..S... ...LL....

Bubalus_bubalis A......... .......... .G..A..... D.L.S..... .......L.. .......... ........S. .A........ V..L..S... ...LL....

Camelus_ferus A......... ....AA.... .S..A..... D.L.S..D.. .......L.. .......F.. .......... .A........ V..L..S... ...LL....

Capra_hircus A......... ....A..... .S..A..... D.L.S..... .......L.. .......... .......... .A........ V..L..S... ...LL....

Catagonus_wagneri A......... ....AA.... .S..A..... D.L.S..D.. .......L.. .......F.. ....A..... .A........ ...L..S... ...LL....

Cervus_hanglu A......... ....A..... .S..A..... D.L.S..... .......L.. .......... .......... .A........ A..L..SV.. ...LL....

Moschus_moschiferus A......... ....A..... .S..A..... D.L.S..... .......L.. .......... .......... .A........ V..L..S... ...LL....

Sus_scrofa A......... ....AA.... .S..A..... D.L.S..D.. .......L.. .......F.. .......... .A........ ...L..S... ...LL....

Ailuropoda_melanoleuca A......... ..R.A..... .S..P..... D.L.S..D.. .......L.. ......H... ........A. .AF....... V..L..GL.. ...LL....

Callorhinus_ursinus A......... ..R.A..... .S..P..... D.L.S..D.. .......L.. .......... .......... .AF....... ...L..S... ...LL....

Canis_lupus A......... ..R.A..... .S..P..... D.L.S..D.. .......L.. .......... ....M..... .AF...C... M.QL..SV.. ...LL....

Enhydra_lutris A......... ..R.A..... .S..P..... D.L.S..D.. .......L.. .......... .......... .AF....... ...L..S... ...LL....

Felis_catus A......... ..R.A..... .S..P..... D.L.S..D.. .......L.. .......... .......... .AF....... ...L..S... ...LL....

Halichoerus_grypus A......... ..R.A..... .S..P..... D.L.S..D.. .......L.. .......... .......C.. .AF....... ...L..S... ...LL....

Lontra_canadensis A......... ..R.A..... .S..P..... D.L.S..D.. .......L.. .......... .......... .AF....... ...L..S... ...LL....

Mirounga_leonina A......... ..R.A..... .S..P..... D.L.S..D.. .......L.. .......... ........M. .AF....... ...L..S... ...LL....

Mustela_erminea A......... ..R.A..... .S..P..... D.L.S..D.. .......L.. .......... .......... .AF....... ...L..S... ...LL....

Neomonachus_schauinslandi A......... ..R.A..... .S..P..... D.L.S..D.. .......L.. .......... .......... .AF....... ...L..S... ...LL....

Neovison_vison A......... ..R.A..... .S..P..... D.L.S..D.. .......L.. .......... .......... .AF....... ...L..S... ...LL..T.

Odobenus_rosmarus A......... ..R.A..... .S..P..... D.L.S..D.. .......L.. .......... .......... .AF....... ...L..S... ...LL....

Panthera_leo A......... ..R.A..... .S..P..... D.L.S..D.. .......L.. .......... .......... .AF....... ...L..S... ...LL....

Panthera_pardus A......... ..R.A..... .S..P..... D.L.S..D.. .......L.. .......... .......... .AF....... ...L..S... ...LL....

Phoca_vitulina A......... ..R.A..... .S..P..... D.L.S..D.. .......L.. .......C.. .......... .AF....... ...L..S... ...LL....

Suricata_suricatta A......... ..R.A..... .S..P..... D.L.S..D.. .......L.. .......... .......... .AF....... V..L..S... ...LL....

Ursus_thibetanus A......... ..R.A..... .S..P..... D.L.S..D.. .......L.. .......C.. ........A. .AF....... V..L..GL.. ...LL....

Zalophus_californianus A......... ..R.A..... .S..P..... D.L.S..D.. .......L.. .......... .......... .AF....... ...L..S... ...LL....

Artibeus_jamaicensis A......... .V..AA.... .S..A..... D.L.S..D.. .......L.. ...V...... .......... .A......R. V..L..S... ...LL....

Molossus_molossus A......... .V..AS.... .S..A...R. DVL.S..D.. .......L.H ....T..... .......... .A........ L..L..S... ...LL....

Myotis_myotis A......... .V..AA.... .S..A...E. D.L.S..D.. S......L.. .......... .......... .A........ ...L..S... ...LL....

Phyllostomus_discolor A......... .V..AA.... .S..A..... D.L.S..D.. .......L.. .......... .......... .AI....... V..L..S... ...LL....

Pipistrellus_kuhlii A......... .V..AA.... .S..AP..A. D.L.S..D.. S......L.. .......... .......... .A........ V..L..S... .I.LL....

Pteropus_vampyrus A......... .....A.... .S..A..... D.L.S..D.. .......L.. .......... .......... .A........ V..L..T... ...LL....

Rhinolophus_ferrumequinum A......... .V...A.... .S..A..... D.L.S..D.. .......L.. .......... .......... .AF....... ...L..S... ...LL....

Rousettus_aegyptiacus A......... .V...A.... .S..A..... D.L.S..N.. .......L.. .......... .......... .A........ V..L..T... ...LL....

Sturnira_hondurensis A......... .V..AA.... .S..A..... D.L.S..D.. .......L.. ...V...... ........S. .A......RK V..L..S... ...LL....

Equus_asinus A......... ....A..... ....P..... D.L.S..N.. .......L.. .......... .......... .AF.A..... ...L..S... ...LL....

Equus_caballus A......... ....A..... ....P..... D.L.S..N.. .......L.. .......... .......... .AF.A..... ...L..S... ...LL....

Manis_pentadactyla L......... ....AA.... .S..A..... D.L.S..D.. .......L.. .......... .......... .A........ V..R..S... ...LL..V.

Prolemur_simus A......... ....AA.... .S..A..... D.L.S..D.. .......L.. .......... .......... .A........ V..L.VT... ...LL..V.

Cercocebus_atys A......... ....AA.... .S..A..... D.L.S..D.. ...V...L.. .......... .......... .AF....... ...L.VSV.. ...LL..V.

Chlorocebus_sabaeus A......... ....AA.... .S..A..... D.L.S..D.. ...V...L.. .......... .......... .AF....... ...L.VSV.. ...LL..V.

Colobus_angolensis A......... ....AA.... .S..A..... D.L.S..D.. ...V...L.. .......... .......... .AF....... ...L.VSV.. ...LL..V.

Gorilla_gorilla A......... .....A.... .S..A..... D.L.S..D.. S......L.. .......... .......... .AF....... ...L.VS... ...LL..V.

Homo_sapiens A......... ....AA.... .S..A..... D.L.S..D.. S......L.. .......... .......... .AF..L...W ...L.VS... ...LL..V.

Hylobates_moloch A......... ....AA.... .S..A..... D.L.S..D.. .......L.. .......... .......... .AF....... ...L.VS... ...LL..V.

Macaca_fascicularis A......... ....AA.... .S..A..... D.L.S..D.. ...V...L.. .......... .......... .AF....... ...L.VSV.. ...LL..V.

Macaca_mulatta A......... ....AA.... .S..A..... D.L.S..D.. ...V...L.. .......... .......... .AF....... ...L.VSV.. ...LL..V.

Macaca_nemestrina A......... ....AA.... .S..A..... D.L.S..D.. ...V...L.. .......... .......... .AF....... ...L.VSV.. ...LL..V.

Mandrillus_leucophaeus A......... ....AA.... .S..A..... D.L.S..D.. ...V...L.. .......... .......... .AF....... ...L.VS... ...LL..V.

Microcebus_murinus A......... ....AA.... .S..A..... D.L.S..D.. .......L.. .......... .......... .A........ V..L.VS... ...LL..V.

Nomascus_leucogenys A......... ....AA.... .S..A..... D.L.S..D.. .......L.. .......... .......... .AF....... ...L.VS... ...LL..V.

Otolemur_garnettii A......... ....AA.... .S..A..... D.L.S..D.. .......L.. .......... .......... .AF....... V..L.VS... ...LL....

Pan_paniscus A......... ....AA.... .S..A..... D.L.S..D.. S......L.. .......... .......... .AF....... ...L.VS... ...LL..V.

Pan_troglodytes A......... ....AA.... .S..A..... D.L.S..D.. S......L.. .......... .......... .AF....... ...L.VS... ...LL..V.

Papio_anubis A......... ....AA.... .S..A..... D.L.S..D.. ...V...L.. .......... .......... .AF....... ...L.VSV.. ...LL..V.

Piliocolobus_tephrosceles A......... ....AA.... .S..A..... D.L.S..D.. ...V...L.. .......... .......... .AF....... ...L.VSV.. ...LL..V.

Pongo_abelii A......... ....AA.... .S..A..... D.L.S..N.. .......L.. .......... .......... .AF....... ...L.VS... ...LL..V.

Propithecus_coquereli A......... .V..AA.... .S..A..... D.L.S..D.. .......L.. .......... .......... .A.A...... VL.L.VS... ...LL..V.

Rhinopithecus_bieti A......... ....AA.... .S..A..... D.L.S..D.. ...V...L.. .......... .......... .AF....... ...L.VSV.. ...LL..V.

Rhinopithecus_roxellana A......... ....AA.... .S..A..... D.L.S..D.. ...V...L.. .......... .......... .AF....... ...L.VSV.. ...LL..V.

Sapajus_apella A......... ....AT.... .S..AR.... DEL.S..D.. .......L.. .......... .......... .AF....... ...L.VS... ...LL..V.

Theropithecus_gelada A......... ....AA.... .S..A..... D.L.S..D.. ...V...L.. .......... .......... .AF....... ...L.VSV.. ...LL..V.

Trachypithecus_francoisi A......... ....AA.... .S..A..... D.L.S..D.. ...V...L.. .......... .......... .AF....... ...L.VSV.. ...LL..V.

Oryctolagus_cuniculus A......... ....AA.... .S..A..... D.L.S..D.. .......L.. .......... ....A..... .AFA...... ..KL..S... ...LL....

Arvicanthis_niloticus A......... ....AA.... .S..A..... D.L.S..D.. .......L.. .......... .......... .AF...L... V.KL..S... .A.LL....

Arvicola_amphibius A......... ....AA.... .S..A..... D.L.S..D.. S......L.. .......... .......... .AF......C V.KL..S... ...LL....

Castor_canadensis A......... ....A..... .S..A..... D.L.S..D.. .......L.. .......... .......... .AF....... ...L..S... ...LL....

Chinchilla_lanigera A......... ....AA.... ....A..... D.L.S..D.. .......L.. .......... .......... .AF.A..... V..L..ST.. ...LL....

Cricetulus_griseus A......... ....AA.... .S..A..... D.L.S..D.. S......I.. ...V...... .......... .AF....... L.KL..S... ...LL....

Grammomys_surdaster A......... ....AA.... .S..A..... D.L.S..D.. .......L.. .......... .......... .AF...L... ..KL..S... ...LL....

Heterocephalus_glaber A......... ....AA.... .S..A...R. D.L.S..D.. .......L.. .......... .......... .AF....... V..L..G... ...LL....

Jaculus_jaculus S......... ....AA.... .S..A..... D.L.S..D.. S......L.. .......... .......... .AFA...... V..L..G... ...LL..V.

Marmota_flaviventris A......... ....AA.... .S..A..... D.L.S..D.. .......L.. .......... .......... .AF....... ..KV..SV.. ...LL....

Mastomys_coucha A......... .V..AA.... .S..A..... D.L.S..D.. .......L.. .......... .......... .AF....... ..KL..T... ...LL....

Meriones_unguiculatus A......... ....AA.... .S..A..... D.L.S..D.. S......L.. ...V...... ....I..... .AF....... L.VL..SL.. ...LL....

Mesocricetus_auratus A......... ....AA.... .S..A..... D.L.S..D.. S......L.. .......... .......... .AF....... ...L..S... ...LL....

Microtus_ochrogaster A......... ....AA.... .S..A..... D.L.S..D.. S......L.. .......... .......... .AF......C V.KL..S... ...LL....

Mus_caroli A......... ....AA.... .S..A..... D.L.S..D.. .......L.. .......... .......... .AF....... ..KL..S... ...LL....

Mus_musculus A......... ....AA.... .S..A..... D.L.S..D.. .......L.. .......... .......... .AF....... ..KL..S... ...LL....

Mus_pahari A......... ....AA.... .S..A..... D.L.S..D.. .......L.. .......... .......... .AF....... ..KL..S... ...LL....

Mus_spicilegus A......... ....AA.... .S..A..... D.L.S..D.. .......L.. .......... .......... .AF....... ..KL..S... ...LL....

Nannospalax_galili A......... ....AA.... ....A..... D.L.S..D.. .......L.. .......... .......... .AF....... ..KL..S... ...LL....

Onychomys_torridus A......... ....AA.... .S..A..... D.L.S..D.. S......L.. .......... .......... .AF....... ..KL..S... ...LL....

Peromyscus_leucopus A......... ....AA.... .S..A..... D.L.S..D.. S......L.. .......... .......... .AF....... ..KL..S... ...LL....

Rattus_norvegicus A......... ....AA.... .S..A..... D.L.S..D.. .......L.. .......... .......... .AF....... ..KL..S... ...LL....

Rattus_rattus A......... ....AA.... .S..A..... D.L.S..D.. .......L.. .......... .......... .AF....... ..KL..S... ...LL....

Sciurus_vulgaris A......... ....AA.... .S..A..... D.L.S..D.. .......L.. .......... .......... .AF....... V..L..S... ...LL....

Urocitellus_parryii A......... ....AA.... .S..A..... D.L.S..D.. .......L.. .......... .......... .AF....... ...V..SV.. ...LL....

45.50 5.50 6.50

<<< **$** >> > TM5 **$** <<< >>> TM6 **$** << <

PPP P PP P P G GG GG GG G GG GG G P P P

Ornithorhynchus_anatinus WGIVKYLYED EGCWTRNTNM NYWLIVRLPI LISIGVNFLI FIRVICIIIS KLQANLMCKA DTKCRLAKST LTLIPLLGTH EVIFAFVVDE HARGTLRYV

Monodelphis_domestica .......... ....S..S.. .....I.... .MA.....F. .......... ..K......T .......... .......... .......M.. ........I

Phascolarctos_cinereus .......... ....N..A.. .....I.... ITA..L.... .......... ..K......T .......... .........Q .I.....I.. ........I

Sarcophilus_harrisii .......... ....N..A.. .....I.... .MA..L.... .......... ..K.....RT .......... .......... .I.....I.. ........I

Trichosurus_vulpecula .S........ ....N..A.. .....I.... IMA..L.... .......... ..K......T .......... .........Q .I.....I.. ..Q.....I

Choloepus_didactylus .......... .......S.. .....I.... .FA....... .......VV. ..K......T .I........ .......... .......M.. .......FI

Elephantulus_edwardii ...I...... .......S.. .....I...V .FA....... .V.....VVA ..K......T .I........ .......... .......M.. .......FI

Orycteropus_afer ...I...... .......S.. .F...I.... .FA....... .V.....VVA ..K......T .I........ .......... .......M.. .......FI

Balaenoptera_musculus .......... .......S.. .....I.... .FA....... .V.....VV. ..K......T .......... .......... .......M.. ....M..FI

Delphinapterus_leucas .......... .......S.. .......... .FA....... .V.....VV. ..KT.....T .......... .......... .......M.. ....M..FI

Globicephala_melas .......... .......S.. .....I.... .FA....... .V.....VV. ..KT.....T .......... .......... .......M.. ....I..FI

Lagenorhynchus_obliquidens .......... .......S.. .....I.... .FA....... .V.....VV. ..KT.....T .......... .......... .......M.. ....M..FI

Lipotes_vexillifer ..V....... .......S.. .....I.... .FAV.A.... .V.....VV. ..KT.....T .......... .......... .......M.. ....M..FI

Monodon_monoceros .......... .......S.. .......... .FA....... .V.....VV. ..KT.....T .......... .......... .......M.. ....M..FI

Orcinus_orca .......... .......S.. .....I.... .FA....... .V.....VV. ..KT.....T .......... .......... .......M.. ....M..FI

Phocoena_sinus .......... .......S.. .....I.... .FA....... .V.....VV. ..KT.....T .......... .......... .......M.. ....M..FI

Physeter_catodon .......... .......S.. .....I.... .FA....... .V.....VV. ..K....... .......... .......... .......M.. ....M..FI

Bos_taurus .......... .......S.. .....I.... .FA....... .V.....VV. .........T .I........ .......... .......M.. ....V..F.

Bubalus_bubalis .......... .......S.. .....I.... .FA....... .V.....VV. .........T .I........ .......... .......M.. ....L..F.

Camelus_ferus .......... .......S.. .....I.... .FA....... .V.....VV. ..K......T .......... .......... .......M.. ....M..F.

Capra_hircus .......... .......S.. .....I.... .FA....... .V.....VV. .........T .......... .......... .......M.. ....V..F.

Catagonus_wagneri .......... .......S.. .....I.... .FA....... .V.....VV. ..K......T .......... .......... .......M.. ....M..FI

Cervus_hanglu .......... .......S.. .....I.... .FA....... .V....LVV. ..K......T .A........ .......... .......M.. ....V..F.

Moschus_moschiferus ....R..... .......S.. .....I.... .FA....... .V.....VV. ..K.....RT .......... .......... .......M.. ....A..FI

Sus_scrofa .......F.. .......S.. .....I.... .FA....... .V.....VV. ..K......T .......... .......... .......M.. ....M..FI

Ailuropoda_melanoleuca .......... .......S.. .....I.... .FA....... .......VV. ..K..V...T .I........ .......... ..V....M.. .......FI

Callorhinus_ursinus .....H.... .......S.. .....I.... .FA....... .......VV. ..K......T .I........ .......... .......M.. .......FI

Canis_lupus .......... .......S.. .....I.... .FA....... .......VV. ..K......T .I........ .......... .......M.. ....M..FI

Enhydra_lutris .......... .......S.. .....I.... .FA....... .......VV. ..K..V...T .I........ .......... ..V....M.. ....M..FI

Felis_catus .......... .......S.. .....I.... .FA....... .V.....VV. ..K......T .I........ .......... ..V....M.. .......FI

Halichoerus_grypus .......... .......S.. .....I.... .FA....... .......VV. ..K......T .I........ .......... ..V....M.. .......LI

Lontra_canadensis .......... .......S.. .....I.... .FA....... .......VV. ..K..V...T .I........ .......... ..V....M.. .......FI

Mirounga_leonina .......... .......S.. .....I.... .FA....... .......VV. ..K......T .I........ .......... ..V....M.. .......LI

Mustela_erminea .......... .......S.. .....I.... .FA....... .......VV. ..K..V...T .I........ .......... ..V....M.. .......FI

Neomonachus_schauinslandi .......... .......S.. .....I.... .FA....... .......VV. ..K......T .I........ .......... ..V....M.. .......LI

Neovison_vison .......... .......S.. .....I.... .FA....... .......VV. ..K..V...T .I........ .......... ..V....M.. .......FI

Odobenus_rosmarus .......... .......S.. .....I.... .FA....... .......VV. ..K......T .I........ .......... .......M.. .......FI

Panthera_leo .......... .......S.. .....I.... .FA....... .V.....VV. ..K......T .I........ .......... ..V....M.. .......FI

Panthera_pardus .......... .......S.. .....I.... .FA....... .V.....VV. ..K......T .I........ .......... ..V....M.. .......FI

Phoca_vitulina .......... .......S.. .....I.... .FA..M.... .......VV. ..K......T .I........ .......... ..V....M.. .......LI

Suricata_suricatta .......... .......S.. .....I.... .FA....... .V.....VV. ..K......T .I........ .......... ..V....M.. .......FI

Ursus_thibetanus .......... .......S.. .....I.... .FA....... .......VV. ..K......T .I........ .......... ..V....M.. .......FI

Zalophus_californianus .....H.... .......S.. .....I.... .FA....... .......VV. ..K......T .I........ .......... .......M.. .......FI

Artibeus_jamaicensis .......... .......S.. .....I.... .FA....... .V.....VV. ..K......T .I........ .......... .......M.. ....L..FI

Molossus_molossus .......... .......S.. .....I.... .FA....... .V.....VV. ..K......T .I........ .......... .......M.. .......F.

Myotis_myotis .......... .......S.. .....I.... .FA....... .V.....VV. ..K......T .I........ .......... .......M.. .......FI

Phyllostomus_discolor .......... .......S.. .....I.... .FA....... .V.....VV. ..K......T .I........ .......... .......M.. .......FI

Pipistrellus_kuhlii ......R... .......S.. .....I.... .FAV...... .V.....VV. ..K......T .I........ .......... .......M.. ....A..FI

Pteropus_vampyrus .......... .......S.. .....I.... .FA....... .......VV. ..K......T .I........ .......... .......M.. .......FI

Rhinolophus_ferrumequinum .......... .......S.. .....I.... .FA....... .L.....VV. ..K......T .I........ .......... .......M.. .......FI

Rousettus_aegyptiacus .......... .......S.. .....I.... .FA....... .......VV. ..K......T .I........ .......... .......M.. .......FI

Sturnira_hondurensis .......... .......S.. .....I.... .VA....... .V.....VV. ..K......T .I........ .......... .......... ....M..FI

Equus_asinus .......... .......S.. .....I.... .FA....... .V.....VV. ..K......T .I........ .......... .I.....I.. .......FI

Equus_caballus .......... .......S.. .....I.... .FA....... .V.....VV. ..K......T .I........ .......... .I.....I.. .......FI

Manis_pentadactyla .....H.... .......S.. .I...I.... .FA..M.... .V.....VV. ..K......T .I.....R.. .......... .......M.. ....M..FI

Prolemur_simus .......... .......S.. .....I.... .FA....... .V.....VV. ..K......T .I........ .......... .......M.. .......FI

Cercocebus_atys .......... .......S.. .....I.... .FA....... .V.....VV. ..K......T .I........ .......... .......M.. .......FI

Chlorocebus_sabaeus .......... .......S.. .....I.... .FA....... .V.....VV. ..K......T .I........ .......... .......M.. .......FI

Colobus_angolensis .......... .......S.. .....I.... .FA....... .V.....VV. ..K......T .I........ .......... .......M.. .......FI

Gorilla_gorilla .......... .......S.. .....I.... .FA....... .V.....VV. ..K......T .I........ .......... .......M.. .......FI

Homo_sapiens .......... .......S.. .....I.... .FA....... .V.....VV. ..K......T .I........ .......... .......M.. .......FI

Hylobates_moloch .......... .......S.. .....I.... .FA....... .V.....VV. ..K......T .I........ .......... .......M.. ....A..FI

Macaca_fascicularis .......... .......S.. .....I.... .FA....... .V.....VV. ..K......T .I........ .......... .......M.. .......FI

Macaca_mulatta .......... .......S.. .....I.... .FA....... .V.....VV. ..K......T .I........ .......... .......M.. .......FI

Macaca_nemestrina .......... .......S.. .....I.... .FA....... .V.....VV. ..K......T .I........ .......... .......M.. .......FI

Mandrillus_leucophaeus .......... .......S.. .....I.... .FA....... .V.....VV. ..K......T .I........ .......... .......M.. .......FI

Microcebus_murinus ..V....... .......S.. .....I.... .FA....... .V.....VV. ..K......T .I........ .......... .......M.. .......FI

Nomascus_leucogenys .......... .......S.. .....I.... .FA....... .V.....VV. ..K......T .I........ .......... .......M.. ....A..FI

Otolemur_garnettii ......F... .......S.. .....I.... .FA....... .V.....VV. ..K......T .I........ .......... .......M.. .......FI

Pan_paniscus .......... .......S.. .....I.... .FA....... .V.....VV. ..K......T .I........ .......... .......M.. .......FI

Pan_troglodytes .......... .......S.. .....I.... .FA....... .V.....VV. ..K......T .I........ .......... .......M.. .......FI

Papio_anubis .......... .......S.. .....I.... .FA....... .V.....VV. ..K......T .I........ .......... .......M.. .......FI

Piliocolobus_tephrosceles .......... .......S.. .....I.... .FA....... .V.....VV. ..K......T .I........ .......... .......M.. .......FI

Pongo_abelii .......... .......S.. .....I.... .FA....... .V.....VV. ..K......T .I........ .......... .......M.. .......FI

Propithecus_coquereli .......... .......S.. .....I.... .FA....... .V.....VV. ..K......T .I........ .......... .......M.. .......FI

Rhinopithecus_bieti .......... .......S.. .....I.... .FA....... .V.....VV. ..K......T .I........ .......... .......M.. .......FI

Rhinopithecus_roxellana .......... .......S.. .....I.... .FA....... .V.....VV. ..K......T .I........ .......... .......M.. .......FI

Sapajus_apella .......... .......S.. .....I.... .FA....... .V.....VVA ..K......T .I........ .......... .......M.. .......FI

Theropithecus_gelada .......... .......S.. .....I.... .FA....... .V.....VV. ..K......T .I........ .......... .......M.. .......FI

Trachypithecus_francoisi .......... .......S.. .....I.... .FA....... .V.....VV. ..K......T .I........ .......... .......M.. .......FI

Oryctolagus_cuniculus .......... .......S.. .....I.... .FA....... .......VV. ..K......T .I........ .......... .......M.. .......F.

Arvicanthis_niloticus .......... .......S.. .....I.... .FA....... .......VV. ..K......T .I........ .......... .......M.. .......F.

Arvicola_amphibius ..V....... .......S.. .....I.... .FA....... .......VV. ..K......T .I........ .......... .......M.. .......FI

Castor_canadensis .......... .......S.. .....I.... .FA....... .......VV. ..K......T .I........ .......... .......M.. .......FI

Chinchilla_lanigera .......... .......S.. .....I.... .FA....... .......VV. .........T .I........ .......... .......M.. .......FI

Cricetulus_griseus ..V.R..... .......S.. .....I.... .FA......V .......VV. ..K......T .I........ .......... .......M.. .......FI

Grammomys_surdaster ..V....... .......S.. .....I.... .FA....... .......VV. ..K......T .I........ .......... .......M.. .......F.

Heterocephalus_glaber .......... .......S.. .....I.... .FAV...... .......VV. ..K......T .I........ .......... .......M.. .......FI

Jaculus_jaculus ..V....... .......S.. .....I.... .FA.A..... .......VV. ..K......T .V........ .......... .......M.. .......F.

Marmota_flaviventris .......... .......S.. .....I.... .FA.....F. .V.....VV. ..K......T .I........ .......... .......M.. .......FI

Mastomys_coucha .......... .......S.. .....I.... .FA....... .......VV. ..K......T .I........ .......... .......M.. .......F.

Meriones_unguiculatus .......... .......S.. .....I.... .FA....... .......VV. ..K......T .I........ .......... .......M.. .......F.

Mesocricetus_auratus ..V....... .......S.. .....I.... .FA....... .......VV. ..K......T .I........ .......... .......M.. .......FI

Microtus_ochrogaster ..V....... .......S.. .....I.... .FA....... .......VV. ..K......T .I........ .......... .......M.. .......FI

Mus_caroli .......... .......S.. .....I.... .FA....... .......VV. ..K......T .I........ .......... .......M.. .......FI

Mus_musculus .......... .......S.. .....I.... .FA....... .......VV. ..K......T .I........ .......... .......M.. .......FI

Mus_pahari .......... .......S.. .....I.... .FA....... .......VV. ..K......T .I........ .......... .......M.. .......F.

Mus_spicilegus .......... .......S.. .....I.... .FA....... .......VV. ..K......T .I........ .......... .......M.. .......FI

Nannospalax_galili .......... .......S.. .....I.... .FA....... .......VV. ..R......T .I........ .......... .......M.. .......FI

Onychomys_torridus ..V....... .......S.. .....I.... .FA....... .......VV. ..K.....RT .I........ .......... .......M.. .......FI

Peromyscus_leucopus .......... .......S.. .....I.... .FA....... .......VV. ..K......T .I........ .......... .......M.. .......FI

Rattus_norvegicus .......... .......S.. .....I.... .FA......V .......V.A ..K......T .I........ .......... .......M.. .......F.

Rattus_rattus .......... .......S.. .....I.... .FA....... .......V.A ..K......T .I........ .......... .......M.. .......F.

Sciurus_vulgaris .......... .......S.. .....I.... .FA.....F. .......VV. ..K......T .I........ .......... .......M.. .......FI

Urocitellus_parryii .......... .......S.. .....I.... .FA.....F. .V.....VV. ..K......T .I........ .......... .......M.. .......FI

7.50 8.50

>>> TM7 **$** <<< **$**

P PP PP G GG

Ornithorhynchus_anatinus KLFAELSITS FQGL-MVAIL YCFINNEVQA EFRKSWARWR LERSSIERDS SMKPTQCPAG SLSSAGS--- --VYAATCQA SCS

Monodelphis_domestica ..VYQ.FF.. ....-..... .........L ....T.E... ..HLYTQ..C ....LK...S ....G.T-VG SSL....... T..

Phascolarctos_cinereus ...C.I.FA. ....-..... ...V....HL ..Q.T.E... ..HLYTQ..C ....LK...S ....G.T-VG SSL....S.. T..

Sarcophilus_harrisii ...S.I.LA. ....-..... ...V.....L ..Q.T.E... ..HLYTQ..C ....LK.T.S ....G.T-LG SSL....... T..

Trichosurus_vulpecula ...C.I.FA. ....-..... ...V.....L ..Q.T.E... ..HFYTQ..C ....LKY..S ....G.T-VG SSL....S.. T..

Choloepus_didactylus ...T...F.. ....-..... ...V.S...M ......E..Q ...LH.Q... ....LK..TS ....GAM-VG SS..T.S... ...

Elephantulus_edwardii ...T...F.. ....-..... ...V.S...M ......E... ...LY.Q... ....LK..TN ....G.T-VG SS........ ...

Orycteropus_afer ...T...F.. ....-..... ...V.S...M ......E... ..HLH.Q... ....LK..TN ...TG.T-TG SS......R. ...

Balaenoptera_musculus ...T...F.. ....-..... ...V.....M ......E... ..HLH.Q... ....FK..TS ...CE.T-VG SS....S... ...

Delphinapterus_leucas ...T...F.. ....-..... ...V.....M ....T.E... ..HLH.Q.E. ....FK..TS ...CE.T-VG SS....S... ...

Globicephala_melas ...T..FF.. ....-..... ...V.....M ....T.E... ..HLH.Q.E. ....FK..TS ...CE.T-VG SS....S... ...

Lagenorhynchus_obliquidens ...T...F.. ....-..... ...V.....M ....T.E... ..HLH.Q.E. ....FK..TS ...CE.T-VG SS....S... ...

Lipotes_vexillifer ...T...F.. ....-..... ...V.....M ....T.E... ..HLH.Q.E. .R..FK..TS ...CE.T-VG SS....S..V ...

Monodon_monoceros ...T...F.. ....-..... ...V.....M ....T.E... ..HLH.Q.E. ....FK..TS ...CE.T-VG SS....S... ...

Orcinus_orca ...T...F.. ....-..... ...V.....M ....T.E... ..HLH.Q.E. ....FK..TS ...CE.T-VG SS....S... ...

Phocoena_sinus ...T...F.. ....-..... ...V.....M ....T.E... ..HLH.Q.E. ....FK..TS ...CE.T-VG SS....S... ...

Physeter_catodon ...T...F.. ....-..... ...V.....M ....T.E... ..HLH.Q... ....FK..TS ...CE.T-VG SS....S... ...

Bos_taurus ...T...F.. ....-..... ...V.....M ......E..Q ..HLHVQ.EN ....FK..TS ...CGAT-AG SS....S..T ...

Bubalus_bubalis ...T...F.. ....-..... ...V.....M ......E... ..HLHVQ.EN ....FK..TS ...CGAT-AG SS....S..T ...

Camelus_ferus ...T...F.. ....-..... ...V.....M ......E... ..HLHTQ... ....FK..TS ...CG.T-VG SS....S... ...

Capra_hircus ...T...F.. ....-..... ...V.....M ......E... ..HLHVQ.EN ....FK..TS ...CGAT-AG SS....S..T ...

Catagonus_wagneri ...T...F.. ....-..... ...V.....M ......E... ..HLH.Q... ....FK..TS ...CGAT-VG SS..T.S... ...

Cervus_hanglu ...T...F.. ....-..... ...V.....M ......E... ..HLHVQ.EN ....FK..TS ...CGPT-AG SSM.------ ---

Moschus_moschiferus ...T...F.. ....-..... ...V.....M ......E... ..HLHVQ.EN ....FK..SS ...CGAT-AG SSM...S..T ...

Sus_scrofa ...T...F.. ....-..... ...V.....M ......E... ..HLH.Q... ....FK..TS ...CGAT-VG SS..S.S... ...

Ailuropoda_melanoleuca ...T...F.. ....-..... ...V.....M ...R..E... .KHLH.Q... ....LK..TS ....GAT-AG SS........ ...

Callorhinus_ursinus ...T...F.. ....-..... ...V.....M ...R..E... .KHLH.Q... ....LK..TS ....G.T-VG SS........ ...

Canis_lupus ...T...F.. ....-L.... ...V.....M ...R..E... ..HLHVQ... ..R.LK..TS ....G.T-VG SS....S... ...

Enhydra_lutris ...T...F.. ....-..... ...V.....L ...R..E... .KHLHTQG.. ..R.LK..TS ....G.T-VG SSI....... ...

Felis_catus ...T...F.. ....-..... ...V.....M ...R..E... .KHLH.Q... ....LK..TS ..T.G.T-VG SS....S... ...

Halichoerus_grypus ...T...F.. ....-..... ...V.....M ...R..E... .KHLH.Q... ....LK..TS ..N.G.T-VG SS........ ...

Lontra_canadensis ...T...F.. ....-..... ...V.....L ...R..E... .KHLHTQG.. ..R.LK..TS ....G.T-VG SS........ ...

Mirounga_leonina ...T...F.. ....-..... ...V.....M ...R..E... .KHLH.Q... ....LK..TS ....G.T-VG SS........ ...

Mustela_erminea ...T...F.. ....-..... ...V.....L ...R..E... .KHLHTQG.. ..R.LK..TS ....G.T-MG SS........ ...

Neomonachus_schauinslandi ...T...F.. ....-..... ...V.....M ...R..E... .KHLH.Q... ....LK..TS ....G.T-VG SS..T..... ...

Neovison_vison ...T...F.. ....-..... ...V.....L ...R..E... .KHLHTQG.. ..R.LK..TS ....G.T-VG SS........ ...

Odobenus_rosmarus ...T...F.. ....-..... ...V.....M ...R..E... .KHLH.Q... ....LK..TS ....G.T-VG SS.....S.. ...

Panthera_leo ...T...F.. ....-..... ...V.....M ...R..E... .KHLH.Q... ....LK..TS ..T.G.T-VG SS........ ...

Panthera_pardus ...T...F.. ....-..... ...V.....M ...R..E... .KHLH.Q... ....LK..TS ..T.G.T-VG SS........ ...

Phoca_vitulina ...T...F.. ....-..... ...V.....M ...R..E... .KHLH.Q... ....LK..SS ....G.T-VG SS........ ...

Suricata_suricatta ...T...F.. ....-..... ...V.....M ...R..E... .KHLH.Q... ....LK..TS ....G.T-VG SS........ .F.

Ursus_thibetanus ...T...F.. ....-..... ...V.....M ...R..E..Q .KHLH.Q... ....LK..TS ....GAT-VG SS........ ...

Zalophus_californianus ...T...F.. ....-..... ...V.....M ...R..E... .KHLH.Q.G. ....LK..TS ....G.T-VG SSI....... ...

Artibeus_jamaicensis ..LT...F.. ....-V.... ...V.S...M ......E... ..HLHMQ... ....LK..TS ....G.T-VG SSM....... ...

Molossus_molossus ..LT...F.. ....-V.... ...V.....M ...R..E..Q ...LHMD.G. ....LK..TS ....G.M-VG SSM....... .Y.

Myotis_myotis ..LT...F.. ....-..... ...V.....L ....R.E... ...LHMQ... ....L...TS ....G.T-VG SS..S..... ...

Phyllostomus_discolor ..LT...F.. ....-...V. ...V.S...M ......E... ..HLHMQ... ....LK..TS ....G.T-VG SSM.T..... ...

Pipistrellus_kuhlii ..LT...F.. ....-..... ...V.....L ....R.E... ...LHMQ... ....L..ATS ....G.A-AG SS..T....V ...

Pteropus_vampyrus ..LT...F.. ....-..... ...V.S...M ......E... ..HLH.Q... ....LK..TS ....G.M-VG SS........ ...

Rhinolophus_ferrumequinum ..LT...F.. ....-..... ...V.....M ......E... ..HLH.Q... ....LK..TS ....G.M-VG SS........ ...

Rousettus_aegyptiacus ..LT...F.. ....-..... ...V.S...M ......E... ..HLH.Q... ....LK..TS ....G.T-VG SS.......V ...

Sturnira_hondurensis ..LT...F.. ....-..... ...V.S...M ......E... ..HLHVQ... ....LK..TS ....G.T-VG SSM....... ...

Equus_asinus ...T...F.. ....-..... ...V.....M ......E... ..HLH.Q... ....LK..TS ....G.T-VG SG.......T ...

Equus_caballus ...T...F.. ....-..... ...V.....M ......E... ..HLH.Q... ....LK..TS ....G.T-VG SG.......T ...

Manis_pentadactyla ...T...F.. ....-...V. ...V.....T ...RG.E... .KHLH.Q... ....LK..TS ....G.P-VG SS.......I ...

Prolemur_simus ...T...F.. ....-..... ...V.....M ......E... ..HLH.Q... ...ALK..NS ....GAT-VS SS..S.P... ...

Cercocebus_atys ...T...F.. ....-..... ...V.....L ......E... ..HLH.Q... ....LK..TS ....GAT-AG SSM.T..... ...

Chlorocebus_sabaeus ...T...F.. ....-..... ...V.....L ......E... ..HLH.Q... ....LK..TS ....GAT-AG SSM.T..... ...

Colobus_angolensis ...T...F.. ....-..... ...V.....L ......E... ..HLH.Q... ....LK..TS ....GAT-AG SSM.T..... ...

Gorilla_gorilla ...T...F.. ....-..... ...V.....L ......E... ..HLH.Q... ....LK..TS ....GAT-AG SSM.T..... ...

Homo_sapiens ...T...F.. ....-..... ...V.....L ......E... ..HLH.Q... ....LK..TS ....GAT-AG SSM.T..... ...

Hylobates_moloch ...T...F.. ....-..... ...V.....L ......E... ..HLH.Q... ....LK..TS ....GAT-AG SS..T..... ...

Macaca_fascicularis ...T...F.. ....-..... ...V.....L ......E... ..HLH.Q... ....LK..TS ....GAT-AG SSM.T..... ...

Macaca_mulatta ...T...F.. ....-..... ...V.....L ......E... ..HLH.Q... ....LK..TS ....GAT-AG SSM.T..... ...

Macaca_nemestrina ...T...F.. ....-..... ...V.....L ......E... ..HLH.Q... ....LK..TS ....GAT-AG SSM.T..... ...

Mandrillus_leucophaeus ...T...F.. ....-..... ...V.....L ......E... ..HLH.Q... ....LK..TS ....GAT-AG SSM.T..... ...

Microcebus_murinus ...T...F.. ....-..... ...V.....M ......E... ..HLH.Q... ....LK..TS ....GAT-VS SS..S..... ...

Nomascus_leucogenys ...T...F.. ....-..... ...V.....L ......E... ..HLH.Q... ....LK..TS ....GAT-AG SS..T..... ...

Otolemur_garnettii ...T...F.. ....-..... ...V.....M ......E... ..HLH.Q... ....LK..TS ....GAT-VG SS........ ...

Pan_paniscus ...T...F.. ....-..... ...V.....L ......E... ..HLH.Q... ....LK..TS ....GAT-AG SSM.T..... ...

Pan_troglodytes ...T...F.. ....-..... ...V.....L ......E... ..HLH.Q... ....LK..TS ....GAT-AG SSM.T..... ...

Papio_anubis ...T...F.. ....-..... ...V.....L ......E... ..HLH.Q... ....LK..TS ....GAT-AG SSM.T..... ...

Piliocolobus_tephrosceles ...T...F.. ....-..... ...V.....L ......E... ..HLH.Q... ....LK..TS ....GAT-AG SSM.T..... ...

Pongo_abelii ...T...F.. ....-..... ...V.....L ......E... ..HLH.Q... ....LK..TS ....GAT-AG SS..T..... ...

Propithecus_coquereli ...T...F.. ....-...V. ...V.S...M ......E... ..HLHVQ... ....LK.ATS .....AT-VS SS..S..... ...

Rhinopithecus_bieti ...T...F.. ....-..... ...V.....L ......E... ..HLH.Q... ....LK..TS ....GAT-AG SSM.T..... ...

Rhinopithecus_roxellana ...T...F.. ....-..... ...V.....L ......E... ..HLH.Q... ....LK..TS ....GAT-AG SSM.T..... ...

Sapajus_apella ...T...F.. ....-..... .........L ....T.E... ..HLH.Q... ....LK..TS ....GAT-VG SS..T....I ...

Theropithecus_gelada ...T...F.. ....-..... ...V.....L ......E... ..HLH.Q... ....LK..TS ....GAT-AG SSM.T..... ...

Trachypithecus_francoisi ...T...F.. ....-..... ...V.....L ......E... ..HLH.Q... ....LK..TS ....GAT-AG SSM.T..... ...

Oryctolagus_cuniculus ...T...F.. ....-..... ...V.....M ......E... ..HLRVQ... ....LK..TS ....GAT-GG SS.....S.. -..

Arvicanthis_niloticus ...T...F.. ....-..... ...V.....M ......E... ..HLN.Q... ....LK..TS .V..GAT-VG SS.......T ...

Arvicola_amphibius ...T...F.. ....-..... ...V.....M ......E... ..HLN.Q... ....LK..TS .V..GAT-VG SS.......T ...

Castor_canadensis ...T...F.. ....-..... ...V.....M ......E... ..HLH.Q... ....LK..TS ....G.T-VG SS........ ...

Chinchilla_lanigera ...T...F.. ....-..... ...V.....M ......E..Q ..HLH.Q... ....LK..TS ....GAT-VG SS........ T..

Cricetulus_griseus ...T...F.. ....-..... ...V.....M ......E... ..HLN.Q... ....LK..NS .V..GAT-VG SS..T....G ...

Grammomys_surdaster ...T...F.. ....-..... ...V.....M ......E... ..HLN.Q... ....LK..TS .V..GAT-VG SS.......T ...

Heterocephalus_glaber ...T...F.. ....-..... ...V.....M .....CE... ..HLH.Q... ....LK...S ....G.T-VG SS........ ...

Jaculus_jaculus ...T...F.. ....-..... ...V.S...M ......E... ..HLRVQ... ....LK..TN ....GAT-VG SS........ .S.

Marmota_flaviventris ...T...F.. ....-..... ...V.S...M ......E... ..HLH.Q... ....LK..TS ....GAT-VG SS........ ...

Mastomys_coucha ...T...F.. ....-..... ...V.....M ......E... ..HLN.Q..N ....LK..TS .V..GATVVG SS......HT ...

Meriones_unguiculatus ...T...F.. ....-..... .........M ......E... ..HLN.Q... ....LK..TS .V..GAT-VG SS.......T ...

Mesocricetus_auratus ...T...F.. ....-..... ...V.....L ......E... ..HLNVQ... ....LK..TS .V..GVT-VG SS........ ...

Microtus_ochrogaster ...T...F.. ....-..... ...V.....M ......E... ..HLN.Q... ....LK..TS .V..GAT-VG SS.......T ...

Mus_caroli ...T...F.. ....-..... ...V.....M ....C.E... ..HLN.Q... ....LK..TS .V..GAT-VG SSL......T .Y.

Mus_musculus ...T...F.. ....-..... ...V.....M ....C.E... ..HLN.Q..C ....LK..TS .V..GAT-VG SS.......S .Y.

Mus_pahari ...T...F.. ....-..... ...V.....M ....C.E... ..HLN.Q... ....LK..TS .V..GAT-VG SS.......T .Y.

Mus_spicilegus ...T...F.. ....-..... ...V.....M ....C.E... ..HLN.Q..C ....LK..TS .V..GAT-VG SSL......S .Y.

Nannospalax_galili ...T...F.. ....-..... ...V.....M ......E... ..HLR.Q... ....LK..TS ....GAT-VG SS........ ...

Onychomys_torridus ...T...F.. ....-..... ...V.....M ......E... ..HLN.Q... ....LK..TS .V..GAT-VG SS........ ...

Peromyscus_leucopus ...T...F.. ....-..... ...V.....M ......E... ..HWN.Q... ....LK..TS .I..GAT-VG SS........ ...

Rattus_norvegicus ...T...F.. ...F-...V. ...V.....M ......E... ...LN.Q... ....LK..TS .V..GAT-VG SS.......N ...

Rattus_rattus ...T...F.. ...F-...V. ...V.....M ......E... ...LN.Q... ....LK..TS .V..GAW-WQ QR.-S....N ...

Sciurus_vulgaris ...T...F.. ....-..... ...V.S...M ......E... ..HLH.Q... ....LK..TS ....GAT-VG SS........ ...

Urocitellus_parryii ...T...F.. ..WV?.M.F. PLLFPRK..M ......E... ..HLH.Q... ....LK..TS ....GAT-VG SS........ ...

**Supplementary Figure 7. Alignment of mammalian glucagon-like peptide-1 (GLP-1) receptor (Glpr1) protein sequences**.

Predicted glucagon-like peptide-1 receptor (Glp1r) amino acid sequences from 105 mammals are based on the MAFFT [51] aligned coding sequences. Sequences are shown in single letter amino acid code, with identical residues indicated by a period (.) and gaps by dashes (-). The positions of the signal peptide and transmembrane domains (TM1 – TM7) in the human (*Homo sapiens*) sequence are indicated above the sequences, with <<< and >>> indications the extent of these domains. Amino acid sites involved in peptide ligand binding and G-protein binding are indicate by “P” and “G”, respectively, above the sequences and are from the GPCRdb [63,64]. The anchor points for the Wootten numbering system [73] are indicated by $ with the corresponding numbers shown above.
