# Supplementary material for: Variation in the Evolution and Sequences of Proglucagon and the Receptors for Proglucagon-Derived Peptides in Mammals
Source: Front Endocrinol (Lausanne). 2021 Jul 12;12:700066. doi: 10.3389/fendo.2021.700066 (PMC8312260; doi:10.3389/fendo.2021.700066)
Supplement: Supplementary File 1 — Fasta formatted proglucagon (Gcg) coding sequences. [file DataSheet_1.zip › Supplement/Suplementary Figures/Fig S1 Gcg tree.pdf]

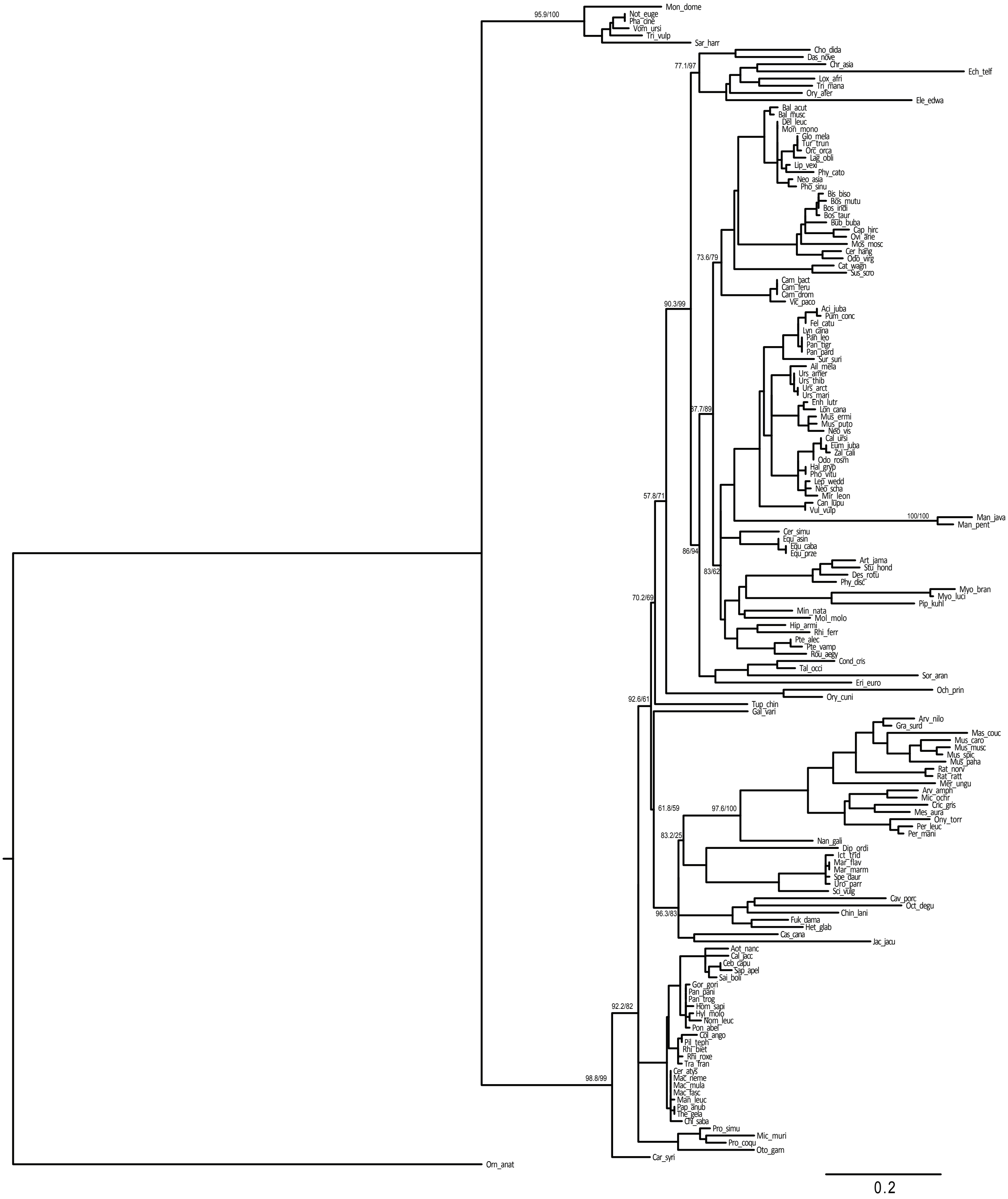

Supplementary Figure 1. Phylogeny of mammalian proglucagon (*Gcg*) coding sequences.
Maximum likelihood phylogenetic tree of *Gcg* coding sequences generated by IQ-tree (version 1.6.12) [54] using a codon-based DNA alignment with the model of sequence evolution MGK+F1X4+G4) selected using ModelFinder [55]. The phylogeny was rooted with a sequence from *Ornithorhynchus anatinus* (Orn_anat; a member of order Monotremata). Numbers at the nodes indicate the Shimodaira–Hasegawa approximate likelihood ratio test (SH-aLRT support) (%) / ultrafast bootstrap support (%) values, each based on 1000 replicates. Support values are only shown for early diverging lineages. Branch lengths are proportional to the amount of inferred sequence change, with the scale bar shown at the bottom. Species names are abbreviated, with full names listed in **Supplementary Table 1**.
